# A Genomic Survey of Angiotensin-Converting Enzymes Provides Novel Insights into Their Molecular Evolution in Vertebrates

**DOI:** 10.3390/molecules23112923

**Published:** 2018-11-09

**Authors:** Yunyun Lv, Yanping Li, Yunhai Yi, Lijun Zhang, Qiong Shi, Jian Yang

**Affiliations:** 1 School of Applied Chemistry and Biotechnology, Shenzhen Polytechnic, Shenzhen 518055, China; lvyunyun@genomics.cn (Y.L.); c7zlj@szpt.edu.cn (L.Z.); 2 BGI Education Center, University of Chinese Academy of Sciences, Shenzhen 518083, China; yiyunhai@genomics.cn; 3 Shenzhen Key Lab of Marine Genomics, Guangdong Provincial Key Lab of Molecular Breeding in Marine Economic Animals, BGI Academy of Marine Sciences, BGI Marine, BGI, Shenzhen 518083, China; liyanping@genomics.cn

**Keywords:** angiotensin-converting enzyme, phylogeny, genomic comparison, evolution

## Abstract

Angiotensin-converting enzymes, ACE and ACE2, are two main elements in the renin–angiotensin system, with a crucial role in the regulation of blood pressure in vertebrates. Previous studies paid much attention to their physiological functions in model organisms, whereas the studies on other animals and related evolution have been sparse. Our present study performed a comprehensive genomic investigation on *ace* and *ace2* genes in vertebrates. We successfully extracted the nucleotide sequences of *ace* and *ace2* genes from high-quality genome assemblies of 36 representative vertebrates. After construction of their evolutionary tree, we observed that most of the phylogenetic positions are consistent with the species tree; however, certain differences appear in coelacanths and frogs, which may suggest a very slow evolutionary rate in the initial evolution of *ace* and *ace2* in vertebrates. We further compared evolutionary rates within the entire sequences of *ace* and *ace2*, and determined that *ace2* evolved slightly faster than *ace*. Meanwhile, we counted that the exon numbers of *ace* and *ace2* in vertebrates are usually 25 and 18 respectively, while certain species may occur exon fusion or disruption to decrease or increase their exon numbers. Interestingly, we found three homologous regions between *ace* and *ace2*, suggesting existence of gene duplication during their evolutionary process. In summary, this report provides novel insights into vertebrate *ace* and *ace2* genes through a series of genomic and molecular comparisons.

## 1. Introduction

Serving as a principal volume-regulatory effector, the renin–angiotensin system (RAS) is one of the most important volume regulators in vertebrates. In human, it is a major regulator of blood pressure within the body. Therefore, the RAS could be a target biological system for the treatment of hypertension and other cardiovascular diseases [1]. As we know, the initial effective precursor molecule in the RAS is angiotensinogen (AGT), which has been generated mainly in the liver [2]. Angiotensinogen is cleaved to a 10-amino acid (aa) peptide named as angiotensin (Ang I) by a unique aspartyl protease termed renin, which is processing in the kidney [2,3]. Subsequently, angiotensin-converting enzyme (ACE) cleaves Ang I to a shorter-length (8 aa) while high-active peptide, angiotensin II (Ang II), plays a crucial role in causing vasoconstriction [4]. Meanwhile, Ang I and Ang II can alternatively be cleaved to other products such as Ang (1–9) and Ang (1–7), which displayed inactivity for vasoconstriction [5]. These two cleavage processes are regulated by the recently discovered angiotensin-converting enzyme-2 (ACE2) [5].

**Figure 1:**
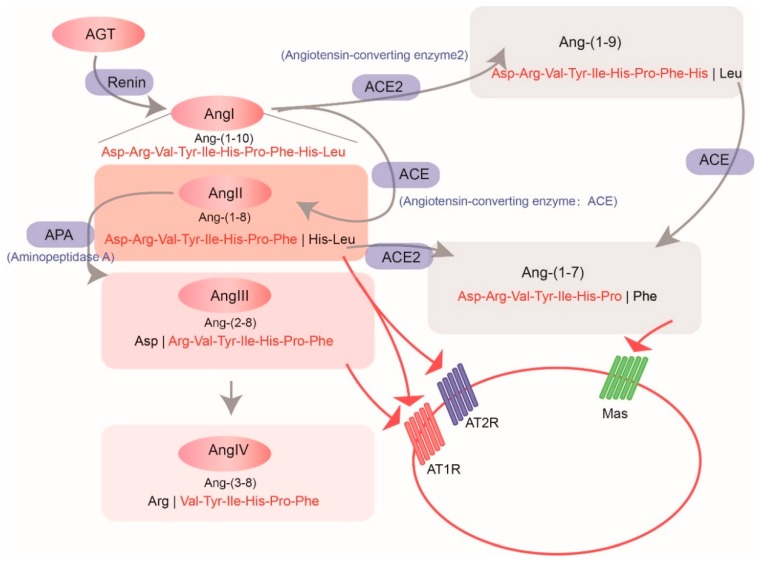
The classical pathway of vertebrate renin–angiotensin system (RAS). Grey arrows emphasize the enzymes that catalyze related reactions. Red words stand for the products, and the “|” between red and black words indicate the cleavage sites.

Contrary to the slow Ang I, Ang II is fast with signaling through two receptors, angiotensin II receptor type 1 (AT1R) and type 2 (AT2R); however, Ang (1–7) can signal through a unique G protein-coupled receptor, encoded by the MAS1 proto-oncogene (*Mas*) [6] (see more details in Figure 1). Thus, ACE plays an important role in the elevation of blood pressure, but ACE2 serves as an ACE-inhibitor to ease the function of ACE and regulate cardiac functions. Therefore, ACE2 can also be a potential target for treatment of hypertension and cardiovascular diseases.

The N and C domains in the ACE proteins are similar in sequence (Figure S1). Both of them contain a catalytically active site, characterized by a consensus zinc-binding motif (HEXXH in the single-letter aa code, where X is any other aa) and a glutamine near the carboxyl terminus that also binds zinc [7]. It is interesting to determine that ACE2 serves as an ACE-inhibitor and displays a homologous feature in its encoding regions with a single zinc-binding catalytic domain, and it is a carboxypeptidase with preference for carboxy-terminal hydrophobic or basic residues [8]. Gene structural comparisons indicated that *ace* and *ace2* arose by duplication from a common ancestor [8]; *ace* and *ace2* are identified in vertebrates, but also exist in primitive chordates and tunicates, suggesting an early origin of the RAS [9].

However, all major components of the RAS, with an exception of the Mas receptor, are presented at the divergence of bony fish [9]. In previous reports, ACE and ACE2 have been studied with comparative physiological techniques as their important functions in the regulation of blood pressure [10,11,12]. A comprehensive and systematic investigation of their evolution in vertebrates, however, has been absent, although a few reports had examined *ace* and *ace2* in limited vertebrate species.

In our present study, we aimed to provide a comprehensive investigation on *ace* and *ace2* genes in vertebrates. Recently published genomes of representative vertebrates with high quality were downloaded for screening. Through gene extractions, we successfully collected encoding nucleotide sequences of *ace* and *ace2* from target species. Subsequently, we performed extensive comparisons between *ace* and the homologous *ace2*, including phylogenetic construction, gene structural comparison, investigation of exon-length distribution, evolutionary rate comparison and sequence identity within each homologous exon. For this project, we would like to answer the following questions: (1) Are the phylogenetic relationships of vertebrate *ace* and *ace2* consistent with the species tree? (2) Do the *ace* and *ace2* gene structures in certain vertebrates present some variations? (3) Are the evolutionary rates of vertebrate *ace* and *ace2* similar? (4) Are exons in *ace* and *ace2* homologous or not?

## 2. Results

### 2.1. Collection of ace and ace2 Sequences

We successfully collected 35 entire *ace* and *ace2* coding sequences (CDS) from their genomes or corresponding predicted gene sets (Table 1). Combing with the verified sequences of human *ace* and *ace2*, we performed multiple sequence alignment of these 36 pairs of *ace* and *ace2* CDS and generated a total of 4107 and 2850 aligned sites for *ace* (Figure S2) and *ace2* (Figure S3), respectively. Meanwhile, *ace* and *ace2* CDS from a cavefish (*Sinocyclocheilus anshuiensis*; Sa) and amphibious blue-spotted mudskipper (*Boleophthalmus pectinirostris*; Bp) were well supported by corresponding transcriptome assemblies, which were published in our previous papers [13,14]. Thus, our data suggest a good reliability of these extracted CDS.

### 2.2. Phylogenetic Topologies of ace and ace2 Genes

**Figure 2:**
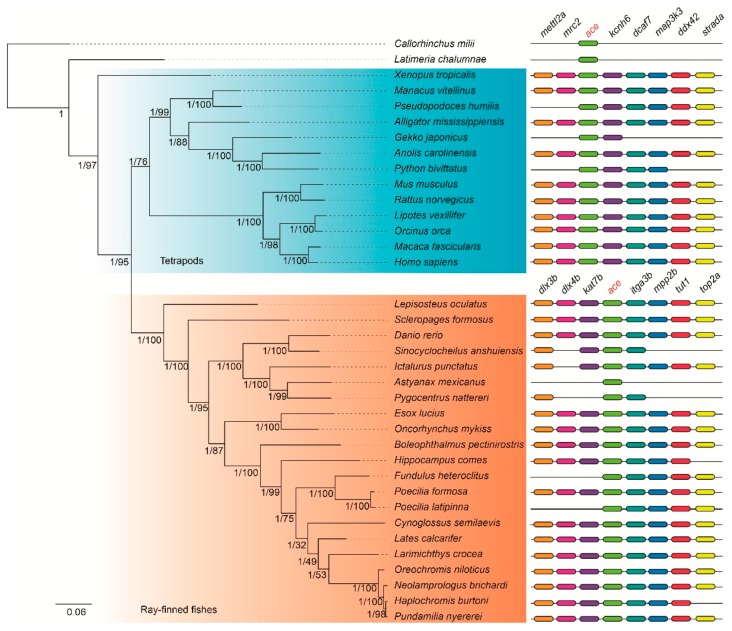
A phylogenetic tree based on 36 *ace* synteny sequences. The left and the right numbers in each node indicate the Bayesian posterior probability (BPP = 1) and the node supports (generated by the ML method), respectively. The scale bar represents nucleotide substitutions per site.

**Figure 3:**
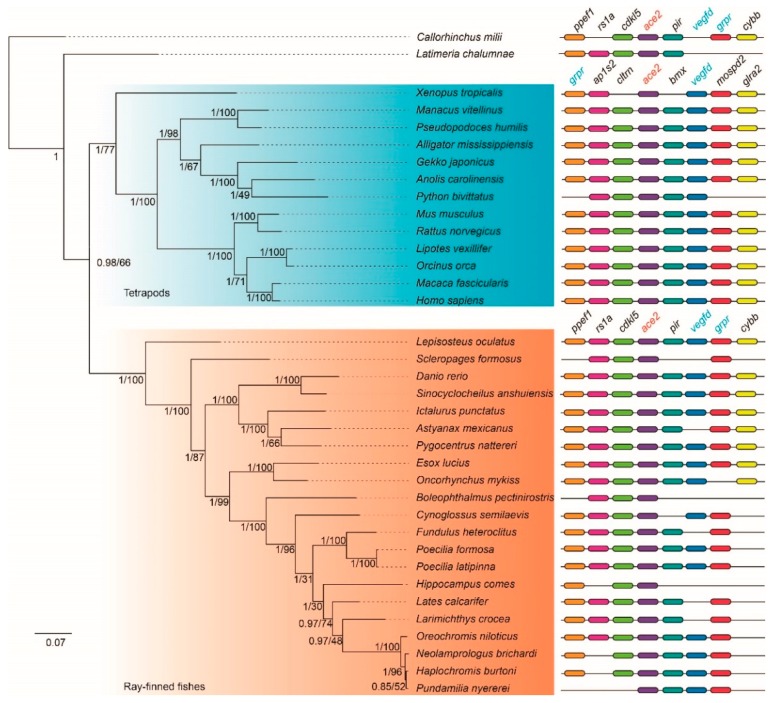
A phylogenetic tree based on 36 *ace2* synteny sequences.

Phylogenetic topologies of *ace* and *ace2* genes, predicted by the Bayesian inference (BI) method, were robust and highly supported as most of the branches displayed high Bayesian posterior probabilities (BPP = 1; see corresponding nodes in Figure 2 and Figure 3). These topologies were also confirmed by the maximum likelihood (ML) method (Figures S4 and S5). It seems that these topologies of *ace* and *ace2* were mostly similar, while some differences between them were also presented.

For the *ace2* genes, two main groups of vertebrates were divided, including tetrapods and ray-finned fishes (Figure 3); while for the *ace* genes, the tropical clawed frog (*Xenopus tropicalis*) located at the outer position of the common ancestor of tetrapods and ray-finned fishes (Figure 2). In addition, coelacanth (*Latimeria chalumnae*) should cluster with tetrapods according to their species tree [15,16]; however, for both *ace* and *ace2*, coelacanth located at the outer of the main clades (Figure 2 and Figure 3). This displayed a discordance between the gene trees of *ace* & *ace2* and their corresponding species trees, which may suggest slow evolutionary rates of *ace* and *ace2* during the early speciation of vertebrates.

Meanwhile, the phylogenetic positions of the tiger seahorse (*Hippocampus comes*) between *ace* and *ace2* are distinct. In the *ace* gene topology, the tiger seahorse located at the closest position with the representative mudskipper Bp, but it is not consistent with the *ace2* gene topology. According to their species tree [17], however, the topologies of *ace* and *ace2* should keep in agreement. This difference may be caused by a special evolutionary episode, which led to an exceptional appearance of the branch for the tiger seahorse *ace2*.

### 2.3. Conserved Synteny of ace and ace2 Genes

In searching the conserved synteny of *ace*, we found no overlaps of *ace* adjacent genes among tetrapods, ray-finned fishes, the elephant shark (*Callorhinchus milii*) and the coelacanth (Figure 2). Thus, we treated tetrapods and ray-finned fishes differently, and then selected eight adjacent genes (*mettl2a*, *mrc2*, *ace*, *kcnh6*, *dcaf7*, *map3k3*, *ddx42* and *strada*) for tetrapods and another eight adjacent genes (*dlx3b*, *dlx4b*, *kat7b*, *ace*, *itga3b*, *mpp2b*, *tut1* and *top2a*) for ray-finned fishes.

In their conserved synteny, most of the *ace* adjacent genes were identified in the 36 vertebrate genomes, but with few failures that could be generated by incomplete assembling of these regions (Figure 3). For the *ace2*, however, we found that two adjacent genes (*vegfd* and *grpr*) are overlapped between tetrapods and ray-finned fishes; while five adjacent genes including *ap1s2*, *cltrn*, *mbx*, *mospd2*, and *glra2* are specific for tetrapods and another five adjacent genes (*ppef1*, *rs1a*, *cdkl5*, *pir* and *cybb*) are specific for the genomes of ray-finned fishes (Figure 2). Interestingly, we observed that the elephant shark or coelacanth shared more *ace2* adjacent genes with ray-finned fishes (Figure 3) than tetrapods. In summary, the differences of conserved synteny in both *ace* and *ace2* genes between tetrapods and ray-finned fishes strongly support rearrangements of their adjacent regions.

### 2.4. Substitution Rate Variations and Gene Structural Changes

**Figure 4:**
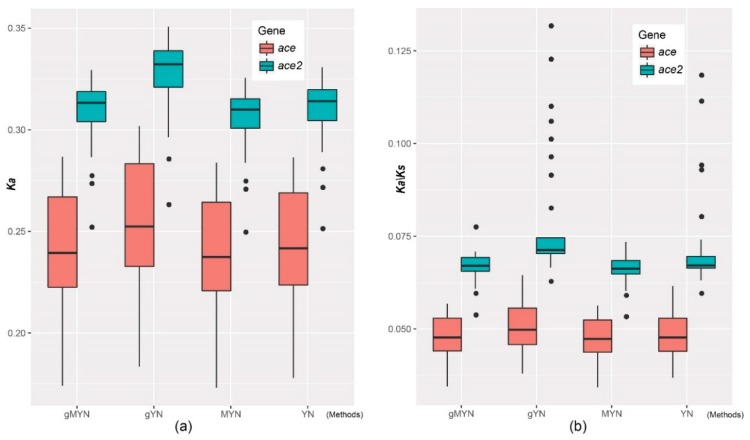
*Ka* and *Ka/Ks* values for *ace* and *ace2* coding sequences. The average values of *Ka* (**a**) and *Ka/Ks* (**b**) in *ace2* using any of the four methods are greater than those of *ace*.

Through comparing nonsynonymous substitution (*Ka*) and the ratio of non-synonymous to synonymous substitutions (*Ka/Ks*) based on four methods, including gMYN (Gamma-MYN) [18], gYN (Gamma-YN) [19], MYN (Modified YN) [20] and YN (Yang Z. and Nielsen R. 2000) [21], we found a conserved substitution rate in both *ace* and *ace2*, in which most of their *Ka/Ks* values are less than 0.1. However, *ace* displayed a lower *Ka/Ks* average value than *ace2* (Figure 4), suggesting that the evolution of *ace* in vertebrates could be more conservative than *ace2*.

**Figure 5:**
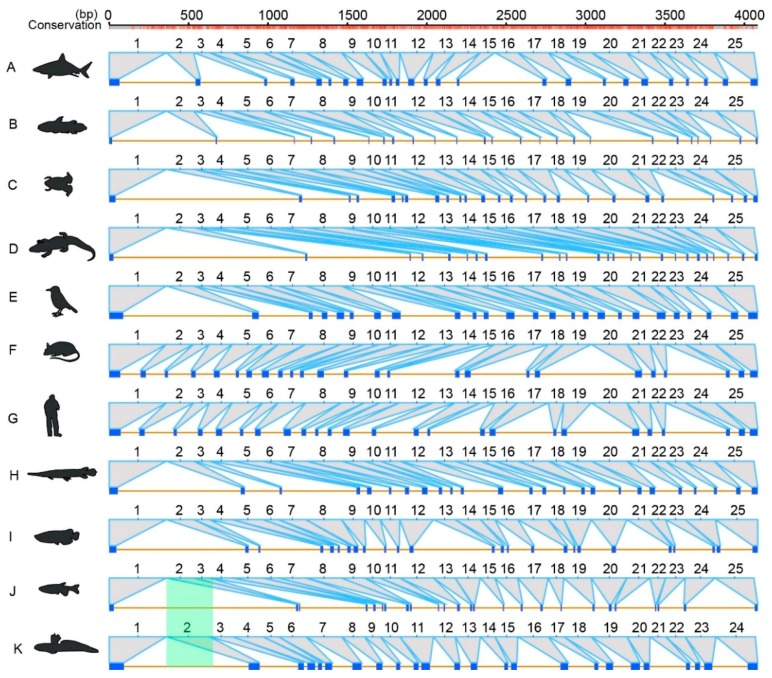
Sequence alignments of the *ace* gene structures in 11 representative vertebrates. Numbers in the top stand for the length of nucleotide base pairs (bp). Red bars in the top indicate the conservation status of the *ace* alignments. The green color shaded region indicates the species-special variations. The representatives include elephant shark (**A**), coelacanth (**B**), tropical clawed frog (**C**), American alligator (**D**), Tibetan ground-tit (**E**), house mouse (**F**), human (**G**), spotted gar (**H**), Asian arowana (**I**), zebrafish (**J**) and the representative mudskipper Bp (**K**).

**Figure 6:**
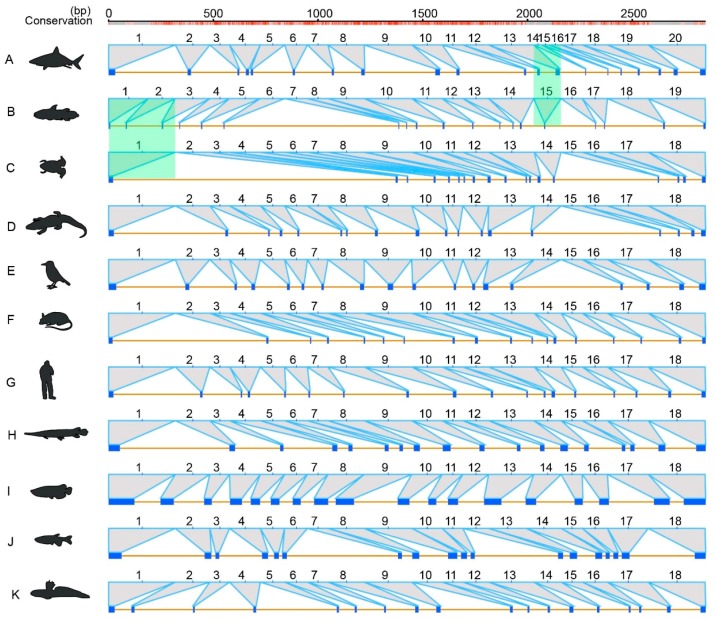
Sequence alignments of the *ace2* gene structures in 11 representative vertebrates. The green color shaded regions indicate the species-special variations. Find corresponding species names (**A**–**K**) in the legend of Figure 5.

For the gene structures in 11 representatives, including elephant shark, coelacanth, tropical clawed frog, American alligator (*Alligator mississippiensis*), Tibetan ground-tit (*Pseudopodoces humilis*), mouse (*Mus musculus*), human (*Homo sapies*), spotted gar (*Lepisosteus oculatus*), Asian arowana (*Scleropages formosus*), zebrafish (*Danio rerio*) and the mudskipper Bp, we predicted 25 exons for *ace* and 18 exons for *ace2* (Figure 5 and Figure 6). These exons are mostly shared, but with certain species-special structural variations, such as 24 exons for *ace* in Bp (Figure 5), 20 exons for *ace2* in the elephant shark, and 19 exons for *ace2* in the coelacanth (Figure 6). Through further exon alignments and mapping onto corresponding genome assemblies, we determined that the disappearance of one exon in Bp was due to fusion of the exons 2 and 3 (Figure 5); the appearance of two additional exons in the elephant shark was due to disruption of the exon 14; and one additional exon in coelacanth was caused by disruption of the exon 2 (Figure 6).

### 2.5. Exon Comparisons and Homologous Region Variations

**Figure 7:**
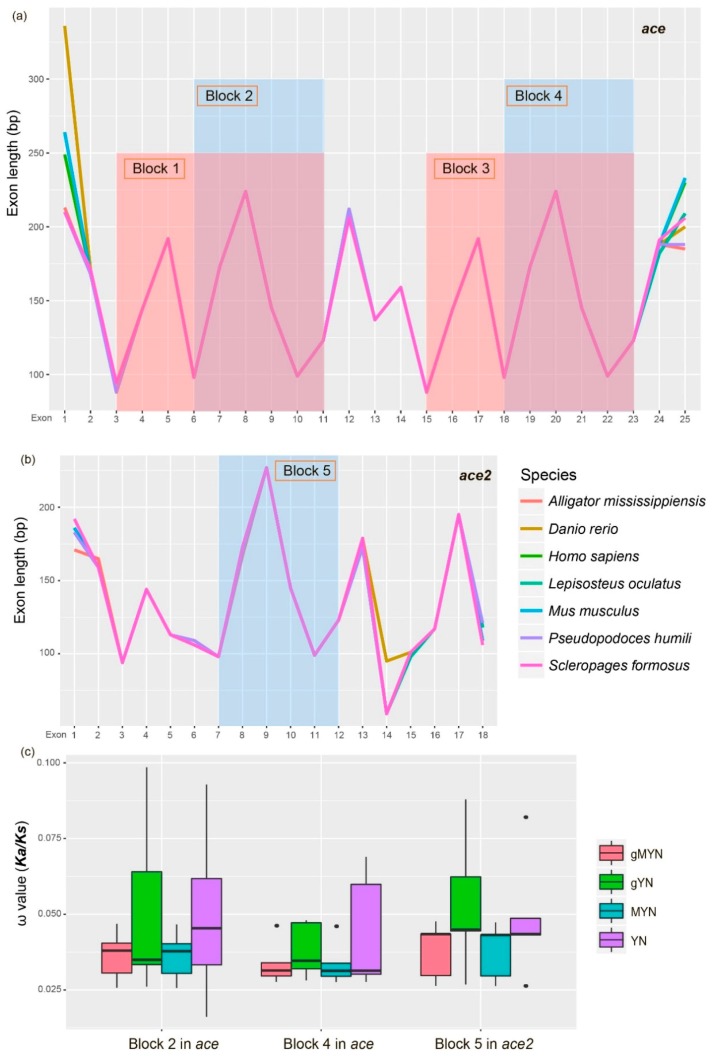
Exon-length distribution in seven representative vertebrates. (**a**) *ace* genes. (**b**) *ace2* genes. (**c**) Comparisons of average *Ka/Ks* values among the three homologous blocks using four methods. The red color shaded regions indicate the two homologous blocks (1 and 3) in *ace*, and the light-blue color shaded regions indicate the three homologous blocks (2, 4 and 5) among *ace* and *ace2*.

Seven species, including the American alligator, zebrafish, human, spotted gar, house mouse, Tibetan ground-tit and Asian arowana, with similar gene structures were selected for comparison of their exon-length in *ace* and *ace2*. We found that the length distribution of exons 3–11 (Block 1) in *ace* was similar to its exons 15–23 (Block 3; Figure 7a). The length distribution of exons 6–11 (Block 5 in Figure 7b) in *ace2* was also similar to the exons 6–11 (Block 2 in Figure 7a) and exons 18–23 (Block 4 in Figure 7a) in *ace*.

Therefore, we inferred two possible homologous blocks (Blocks 1 and 3) inside the *ace* genes and three possible homologous blocks (Blocks 2, 4 and 5) that resided throughout *ace* and *ace2*. Alignments of protein sequences of the corresponding codons in Blocks 2, 4 and 5 indicated a high similarity among these regions (Figure S6). Interestingly, 13 special amino acid sites were consistent in two conserved blocks (Blocks 2 and 4) of *ace*, but they were variable in *ace2* (see more details in Figure S6).

These data suggest a functional divergence in the initial evolution of *ace* and *ace2*. However, the calculation of average *Ka/Ks* of those corresponding codons based on the four methods (gMYN, gYN, MYN and YN) presented similar levels (Figure 7c), suggesting no apparent difference of evolutionary rates among the three homologous regions (Blocks 2, 4 and 5).

We also examined the sequence identity within each exon among Blocks 2, 4 and 5 in the seven representatives. In general, the Blocks 2 and 4 in *ace* displayed higher sequence identity values (ranged from 0.6 to 0.8 in Figure S7a) than others (ranged from 0.4 to 0.6; Figure S7b,c). The uneven identity of these homologous regions between *ace* and *ace2* may imply the asynchronous appearance of their ancestors.

## 3. Discussion

Cardiovascular diseases have been the leading reasons for death in humans worldwide [1,4,11]. The RAS acts as a critical systemic circle to regulate blood pressure, thus maintaining a whole stable blood circulation in our body [2,4]. However, long-time high blood pressure would lead to hypertension to cause related cardiovascular diseases. ACE and ACE2 are pairs of antagonists, in which ACE activates the generation of Ang II, but ACE2 inactivates AngII to reduce the blood pressure; thus ACE2 serves as an ACE inhibitor [1,4] and could be a potential target for neurogenic hypertension [22]. To accelerate the understanding of ACE and ACE2, we investigated *ace* and *ace2* genes from genome assemblies of 36 representative vertebrates, covering cartilaginous fish, ray-finned fishes, amphibians, reptiles, birds and mammals. This study is the first comprehensive investigation of *ace* and *ace2* evolution in vertebrates.

### 3.1. Early Evolution of ace and ace2 Genes in Vertebrates

Homologs of *ace* are extensively distributed in animals [7], including vertebrates (such as chimpanzee, cow, rabbit, mouse, chicken, goldfish and electric eel) and invertebrates (such as house fly, mosquito, horn fly, silk worm, fruit fly, *Caenorhabditis elegans* and bacteria). Similarly, *ace2* homologs have also been identified in both vertebrates [23] and invertebrates [9]. This coincidence indicates a very early origin of *ace* and *ace2* in the biological world.

In vertebrates, we proved a conserved evolution of *ace* and *ace2*, since their phylogenetic tree is mostly consistent with the species tree, with average *Ka/Ks* values far less than 1. However, some evolutionary episodes occurred during the evolution of *ace* and *ace2* in vertebrates, mainly reflected by the conflicts of the phylogenetic positions for frog *ace* and sea horse *ace2*. In addition, we also determined uneven evolving rates of *ace* and *ace2*, with a slightly greater average *Ka/Ks* values in the former. The gene structures of *ace* and *ace2* are also conserved in vertebrates, and most of them have 25 and 18 exons respectively. However, we also found some special structural variations in the mudskipper Bp, elephant shark and coelacanth, with existence of exon fusion or disruption to decrease or increase the exon numbers (Figure 5 and Figure 6). These interesting findings therefore improve our understanding of *ace* and *ace2* in vertebrates.

### 3.2. Rearrangements of ace and ace2 Adjacent Regions

In vertebrates, as we know, two rounds of whole-genome duplication (WGD) occurred in the common ancestor [24,25]. More specifically, the two rounds of WGD happened before the agnatha–gnatostoma and chrondrichthyes–osteichthyes split, respectively. In ray-finned fishes, the teleost experienced additional special whole-genome duplication (TSGD), which was regarded as the third round of WGD in vertebrates [26,27]. In our analysis, all examined species should have experienced the first and second rounds of WGD, and the teleost experienced the third one (TSGD).

In general, WGD duplications can generate pseudogenization, subfunctionalization or neofunctionalization [28]. In our present study, we found only one orthologous *ace* and *ace2* in these representative species as evidenced by our synteny analysis. Thus, we assume that the fate of originally duplicated *ace* and *ace2* genes should have experienced fast loss after the genome duplications. In addition, we identified rearrangements of *ace* and *ace2* adjacent regions in tetrapods and ray-finned fishes (Figure 2 and Figure 3). It seems that the rearrangement around *ace* was more serious than *ace2*, as there is no overlapped adjacent gene around *ace*, while sharing two genes in the *ace2* adjacent regions.

### 3.3. Evolution of Homologous Regions between ace and ace2

Based on the similarity of their protein sequences, *ace* and *ace2* are identified as homologous. ACE2 contains only a single 600-aa peptidase domain, whereas ACE orthologs have two such domains [29]. We confirmed that these domains correspond to the conserved exons such as Blocks 2, 4 and 5 (Figure 7a,b). Thus, we deduce that these regions also displayed coincident gene structures.

It is interesting to know the evolutionary changes among these conserved blocks in *ace* and *ace2*. However, we found no significant difference among the homologous blocks based on different methods, although a slight difference of average *Ka/Ks* values was determined between the entire *ace* and *ace2*. This indicates the natural selection between entire *ace* and *ace2* and their conserved blocks was slightly different.

In addition, we distinguished a closer similarity between Blocks 2 and 4, which both came from *ace*. It suggests that the ancestor of *ace* and *ace2* may appear at different times. Through the protein sequence alignments of the conserved regions (Figure S6), we observed some special sites in ACE2, which are different from those in ACE. These data suggest functional divergence of *ace* and *ace2* during their evolution.

## 4. Materials and Methods

### 4.1. Gene Extraction, Collection and Confirmation

Firstly, the coding DNA sequences (CDS) of human *ace* (NM_000789.3) and *ace2* (NM_021804.2) were downloaded from the National Center for Biotechnology Information (NCBI). Secondly, genome assemblies of 36 representative vertebrates with high-quality (scaffold N50 values over 1 Mb) were selected to realize gene extraction (Table 1). These species are chosen from chondrichthyes, ray-finned fishes, amphibians, reptiles, birds and mammals. Meanwhile, the corresponding whole-genome CDS datasets for the selected vertebrates were download from NCBI to construct a local database. The *ace* and *ace2* CDS from each species were extracted based on the best hits using BLAST (version 2.2.28, NCBI, Bethesda, MD, USA) [30], with the human *ace* and *ace2* CDS as the queries. We also provided further confirmation of the extracted nucleotide sequences by transcriptome data from the mudskipper Bp and cavefish Sa, which were generated previously by our lab [13,14].

### 4.2. Conserved Synteny Identification and Phylogenetic Inference

To evaluate the conservation of *ace* and *ace2* genes, we investigated several genes residing in the upstream and the downstream sequences within tetrapod and teleost genomes, respectively. Through the initial search, we found that the adjacent genes of *ace* and *ace2* in tetrapods and teleost were distinct. Thus, we selected different genes in tetrapods and teleosts to perform further synteny analysis respectively. The sequences served as queries for our synteny analysis were from human and zebrafish.

Meanwhile, all collected nucleotide sequences of *ace* and *ace2* were processed for alignments based on a codon-based mode, which was implemented in MEGA (version 7.0 [31], Temple university, Philadelphia, PA, USA) with the Muscle module. The outcome of alignments was subsequently adjusted manually. The final aligned nucleotide sequences were employed to predict their best nucleotide substitution model under the Akaike Infromation Criterion (AIC) by Jmodeltest (version 2.0 [32], University of Vigo, Vigo, Spain). The parameters within the best nucleotide substitution models (TIM2 + I + G for *ace* and GTR + I + G for *ace2*) were applied using BI in MrBayes (version 3.2.2 [33], Swedish Museum of Natural History, Stockholm, Sweden). We performed two parallel runs for 2-M generations (four chains per run), sampling every 500 generations. The initial 25% runs were discarded for unreliability. Finally, the maximum clade credibility tree from the remaining topologies was identified by using TreeAnnotator (version 1.7.5 [34], University of Auckland, Auckland, New Zealand). Meanwhile, we also employed the maximum likelihood (ML) method to confirm the topology generated by BI. ML was conducted with RAxML 8.0.17 [35] using a GTR + I + G model for our final likelihood search, and switched to the per-site rate category model during fast bootstrapping with 1000 replicates. To avoid the effect of the third site from each aligned codon, we performed the ML analysis again with the protein sequences that were translated from *ace* and *ace2* nucleotide sequences. Their best amino acid substitution models (WAG + I + G for ACE and JTT + I + G for ACE2) were determined under the Akaike Information Criterion (AIC) [36], which were implemented in ProtTest (version 3.4.2 [37], University of Vigo, Vigo, Spain). The ML analysis and 1000 bootstraps were replicated to infer their node supports in PhyML (version 3.1, University of Montpellier, Montpelier, France [38]).

### 4.3. Substitution Rate Estimation and Comparison

Considering the *Ka/Ks* values may differ between *ace* and *ace2* over the evolutionary process, we performed comparisons of the average *Ka* and *Ka/Ks* values between the two genes. The pairwise *Ka* and *Ka/Ks* values were calculated between each pair with codon-based alignments of *ace* and *ace2* under four methods (gMYN [18], gYN [19], MYN [20] and YN [21]) in KaKs_Calculator (version 2.0, Chinese Academy of Sciences, Beijing, China [39]). The average values of *Ka* and *Ka/Ks* were obtained in R language [40] to represent evolutionary rate differences between *ace* and *ace2*.

### 4.4. Examination of Gene Structural Variations

To detect the possible gene structural variations of *ace* and *ace2* in vertebrates, we aligned the collected *ace* and *ace2* CDS onto their corresponding genome assemblies. We selected 11 species to represent the main groups of vertebrates, including the elephant shark, coelacanth, tropical clawed frog, American alligator, Tibetan ground-tit, house mouse, human, spotted gar, Asian arowana, zebrafish and the representative mudskipper Bp. The alignment was performed on NCBI Splign [41] (https://www.ncbi.nlm.nih.gov/sutils/splign/). The generated results of gene structures were integrated and presented on their genome locations. Meanwhile, each coding exon was compared with multiple sequence alignment by a local Perl script. We further chose those *ace* and *ace2* genes without structural variations to compare exon-length distribution. In the homologous regions between *ace* and *ace2*, we extracted the codons to compare their average *Ka/Ks* values as mentioned above. Finally, we compared sequence identity among the exons within homologous regions, which was implemented by a local Perl script and displayed by R language [40].

## 5. Conclusions

This is the first comprehensive investigation and systematic comparisons of *ace* and *ace2* genes in vertebrates. In our present study, extraction of *ace* and *ace2* genes from 36 vertebrate genomes were realized. In their phylogenetic topology, we observed most consistence with the species tree; however, certain differences appear in coelacanths and frogs, which may suggest a very slow evolutionary rate in the initial evolution of *ace* and *ace2* in vertebrates. We further compared the evolutionary rates within the entire *ace* and *ace2*, and found that *ace2* evolved slightly faster than *ace*.

Meanwhile, we counted that exon numbers of *ace* and *ace2* in vertebrates are usually 25 and 18 respectively, whereas certain species exon fusion or disruption may occur to decrease or increase their exon numbers. Interestingly, we determined three homologous regions between *ace* and *ace2*, suggesting their origination from gene duplication. However, their uneven sequence identity may suggest that they evolved at different times. In summary, this report provided novel insights into *ace* and *ace2* genes in vertebrates through a series of genomic and molecular comparisons.

## Figures and Tables

**Table 1:** Selected 36 vertebrates and corresponding Genebank ID for their sequenced genomes.

Class	Common Name	Species Name	GenBank ID
**Chondrichthyes**	Elephant shark	*Callorhynchus milii*	GCA_000165045.2
**Sarcopterygii**	Coelacanth	*Latimeria chalumnae*	GCF_000225785.1
**Amphibians**	Tropical clawed frog	*Xenopus tropicalis*	GCA_000004195.3
**Aves**	Golden-collared manakin	*Manacus vitellinus*	GCA_001715985.2
	Tibetan ground-tit	*Pseudopodoces humilis*	GCA_000331425.1
**Reptiles**	American alligator	*Alligator mississippiensis*	GCF_000281125.3
	Gekko	*Gekko japonicus*	GCA_001447785.1
	Green anole	*Anolis carolinensis*	GCA_000090745.2
	Python bivittatus	*Python bivittatus*	GCA_000186305.2
**Mammals**	House mouse	*Mus musculus*	GCF_000001635.25
	Norway rat	*Rattus norvegicus*	GCF_000001895.5
	Yangtze River dolphin	*Lipotes vexillifer*	GCA_000442215.1
	Killer whale	*Orcinus orca*	GCA_000331955.2
	Crab eating macaque	*Macaca fascicularis*	GCF_000364345.1
	Human	*Homo sapiens*	GCF_000001405.37
**Actinopterygii**	Spotter gar	*Lepisosteus oculatus*	GCF_000242695.1
	Asian arowana	*Scleropages formosus*	GCF_001624265.1
	Zebrafish	*Danio rerio*	GCF_000002035.4
	Chinese golden-line fish (Sa)	*Sinocyclocheilus anshuiensis*	GCF_001515605.1
	Channel catfish	*Ictalurus punctatus*	GCF_001660625.1
	Mexican tetra	*Astyanax mexicanus*	GCF_000372685.1
	Red-bellied piranha	*Pygocentrus nattereri*	GCF_001682695.1
	Northern pike	*Esox lucius*	GCA_000721915.3
	Rainbow trout	*Oncorhynchus mykiss*	GCF_002163495.1
	Blue-spotted mudskipper (Bp)	*Boleophthalmus pectinirostris*	GCF_000788275.1
	Tiger seahorse	*Hippocampus comes*	GCF_001891065.1
	Mummichog	*Fundulus heteroclitus*	GCA_000826765.1
	Amazon molly	*Poecilia formosa*	GCA_000485575.1
	Sailfin molly	*Poecilia latipinna*	GCA_001443285.1
	Tongue sole	*Cynoglossus semilaevis*	GCF_000523025.1
	Barramundi perch	*Lates calcarifer*	GCF_001640805.1
	Large yellow croaker	*Larimichthys crocea*	GCF_000972845.1
	Tilapia	*Oreochromis niloticus*	GCF_001858045.1
	African cichlid	*Neolamprologus brichardi*	GCF_000239395.1
	Burton mouth brooder	*Haplochromis burtoni*	GCF_000239415.1
	Nyerrrei cichlid	*Pundamilia nyererei*	GCF_000239375.1

## Supplementary Materials

The following supplementary materials are available online. Figure S1: Alignment of human *ace* and *ace2* sequences. Figure S2: Alignment of *ace* sequences from human and other 35 vertebrates. Figure S3: Alignment of *ace2* sequences from human and other 35 vertebrates. Figure S4: A phylogenetic tree based on the translated 36 protein sequences of *ace* encoding regions. Number in each node represents the node support generated by the maximum likelihood (ML) method, and the scale bar stands for amino acid substitutions per site. Figure S5: A phylogenetic tree based on the translated 36 protein sequences of *ace2* encoding regions. Figure S6: Protein sequence alignments of the three conserved blocks (2, 4 and 5) in Figure 7 for *ace* and *ace2*. Dots with different hot colors indicate the conservation of each site. The red-line frames and arrows indicate the 13 special sites that displayed consistence in the two conserved blocks (2 and 4) in *ace*, but variable in ace2. Figure S7: Identity comparisons between blocks 1 and 3 & 2 and 4 in *ace* respectively (a), between Block 2 of *ace* and Block 5 of *ace2* (b), or between Block 4 of *ace* and Block 5 of *ace2* (c).
