# Supplementary material for: A Genomic Survey of Angiotensin-Converting Enzymes Provides Novel Insights into Their Molecular Evolution in Vertebrates
Source: Molecules. 2018 Nov 9;23(11):2923. doi: 10.3390/molecules23112923 (PMC6278350; doi:10.3390/molecules23112923)
Supplement: Supplementary file 1 [file molecules-23-02923-s001.zip › Figure S2.pdf]

```
conservation
Callorhinchus milii            ATG.............................................................................   3
Alligator mississippiensis     ATG.............................................................................   3
Anolis carolinensis            ATG.........................................................AGATCTGCGCCTCTATATAA  23
Astyanax mexicanus             ATG.............................................................................   3
Boleophthalmus pectinirostris  ATG.............................................................................   3
Cynoglossus semilaevis         ATG.............................................................................   3
Danio rerio                    ATGAACAGAGGGAAAAGGGAGAGCAGGACACCGATAGGAAAGCAGGAGCATCCAGCAGTGAACGTGTCCAGACGGCTCCT  80
Esox lucius                    ATG.............................................................................   3
Fundulus heteroclitus          ATG.............................................................................   3
Gekko japonicus                ATG.............................................................................   3
Haplochromis burtoni           ATG.............................................................................   3
Hippocampus comes              ATG.............................................................................   3
Ictalurus punctatus            ATG.............................................................................   3
Larimichthys crocea            ATG.............................................................................   3
Lates calcarifer               ATG.............................................................................   3
Latimeria chalumnae            ATG.............................................................................   3
Lepisosteus oculatus           ATG.............................................................................   3
Macaca fascicularis            ATG.............................................................................   3
Manacus vitellinus             ATG.............................................................................   3
Mus musculus                   ATG.............................................................................   3
Neolamprologus brichardi       ATG.............................................................................   3
Oncorhynchus mykiss            ATG.............................................................................   3
Oreochromis niloticus          ATG.............................................................................   3
Poecilia formosa               ATG.............................................................................   3
Poecilia latipinna             ATG.............................................................................   3
Pseudopodoces humilis          ATG.............................................................................   3
Pundamilia nyererei            ATG.............................................................................   3
Pygocentrus nattereri          ATG.............................................................................   3
Python bivittatus              ATG.............................................................................   3
Rattus norvegicus              ATG.............................................................................   3
Scleropages formosus           ATG.............................................................................   3
Sinocyclocheilus anshuiensis   ATG.............................................................................   3
Xenopus tropicalis             ATG.............................................................................   3
Lipotes vexillifer             ATG.............................................................................   3
Orcinus orca                   ATG.............................................................................   3
Homo sapiens                   ATG.............................................................................   3
```

```
                                                                                  10        20        30        40
conservation
Callorhinchus milii            ........................................CAAGTCAAAATGCCTTGGTTTCTGCCACTGCTGCTTATTG  43
Alligator mississippiensis     ...................CCTCCT..............................CTTTTAAGGCTCCTGCTGCTGTTCC  34
Anolis carolinensis            GCCGGCGCCAAGTCTGCGCCCGCCTTTTCGGACGGCGAAGATGCCGCAGCTGTTGGCGTTGGCGCTGCTGCTGCTGTGCT 103
Astyanax mexicanus             .................................................CAGTGGGCCGTTCTGCTGCTGGCCCTCACGG  34
Boleophthalmus pectinirostris  ...................ATGGCCACCAGCGGAGACAGGTCCTTGCGC...CCTCTGCTGTTGCTCCTGCCAGTCTTGG  61
Cynoglossus semilaevis         ......................GCCACTGGGCTGCACAGGTTTCAGCTGCTGCTGCTGCTGCTGCTGCCAGCCTTTC  61
Danio rerio                    CCGGAAACTTTTAAGCCAGCACTCGGCGTCTCAGCTGCGGTCCATTATGCTGCGTGCGGTGGTGCTGCTGACTCTCATCA 160
Esox lucius                    ..................................GCCAGAATGCTTTTGGCAGCGATGGGGCTGATACTGCCAATATCAG  49
Fundulus heteroclitus          .............GGAGCCATGGCTCCTGGGACGCACAGGTTGGTAAGAGGAGCGCTGCTGCTGCTGCTGCCAGCCCTGC  70
Gekko japonicus                ...................CCGCCGCTCCGC.....................GCCTCGCTGCTGCTCCTGCTGCCGCTGC  43
Haplochromis burtoni           ......................GGTACTGGTTTGGACAGGTTTCTGTGG...TCAGTACTGCTGCTGCTGCCAGTCTTTG  58
Hippocampus comes              ..................................GACAAGTTTGTTTGG...TCAGTGGCGCTGGTGCTGCAAGCGGTGC  46
Ictalurus punctatus            .....................................GAACCCATGATGCACTGGGCCGTGTTGCTGCTGTCCCTAGTCG  46
Larimichthys crocea            ......................GGCACCGGTGTGGACAGGTTTCTGTGG...ACAGTCCTGCTGCTGTTGCCAGTCTTGG  58
Lates calcarifer               ......................GGCACTGGTGTGGACAGGTTTCTGTGG...TCTGTGCTGCTGCTGCTGCCAGTCCTGG  58
Latimeria chalumnae            ........................................ACATACCAGATCTTTTGGTTACTGGTGTTTCCACTGCTAT  43
Lepisosteus oculatus           .................................................CTTTGGGTTATGTGGGTTGTGCCCCTCTTTG  34
Macaca fascicularis            .............GGGGCCGCCTCGGGCCGCCAGGGGCCGGGGCCGCTGCTGCAGCCGCTGCTACTGTTGCTGTTGCTA.  69
Manacus vitellinus             ....................................................CCCCCAGCGCTGGGGCTGCTGCTCGGGC  31
Mus musculus                   .............GGGGCCGCGTCCGGCCAGCGGGGGCGGTGGCCGTTGTCGCCTCCGCTCTTGATGCTGTCGCTGCTGG  70
Neolamprologus brichardi       ......................GGTACTGGTTTGGACAGGTTTCTGTGG...TCAGTACTGCTGCTGCTGCCAGTCTTTG  58
Oncorhynchus mykiss            ..................................GCCAGAATGCTTTGGGGAGCGGTGGTGGTGCTACTGCCACTCTTGG  49
Oreochromis niloticus          ......................GGTACTGGTTTGGACAGGTTTCTGTGG...TCAGTACTGCTGCTGCTGCCAGTCTTTG  58
Poecilia formosa               .............GGAGCCATGGCTCCCGAGGCGCGCAGGCTGGTGAGA...GCGGCGCTGCTGCTGCTGCCAGCCCTGC  67
Poecilia latipinna             .............GGAGCCATGGCTCCCGAGGCGCGCAGGCTGGTGAGA...GCGGCGCTGCTGCTGCTGCCAGCCCTGC  67
Pseudopodoces humilis          ....................................................CCCGCGGCGCTGGCGCTGCTGCTCGGGC  31
Pundamilia nyererei            ......................GGTACTGGTTTGGACAGGTTTCTGTGG...TCAGTACTGCTGCTGCTGCCAGTCTTTG  58
Pygocentrus nattereri          .................................................CATTGGGCTGTTTTGCTGCTGGCCCTGATAG  34
Python bivittatus              ...................CCTCCG...........................GTGGCGGGATTTTTCCTCTCGGTCTGCT  37
Rattus norvegicus              .............GGGGCCGCGTCCGGCCAGCGGGGGCGGTGGCCGTTGTCACCGCCGCTCTTGATGCTGTCGCTGCTGC  70
Scleropages formosus           ..............................................CTCTGGGCTGCACTGCTAGTGGCATTGCTGTGTG  37
Sinocyclocheilus anshuiensis   .................................................TACCGGGCAGTGGTGCTGCTGACTCTCATCA  34
Xenopus tropicalis             .................................................AGGGTGCTTCTCCTGGGGCTCCTCCTGCTGG  34
Lipotes vexillifer             .............GGGGCCGCGTCGGGCCGCCGGTGGCCGCGGCCGCTGCTGCTGCCGTTGCTGCTGCTGCTGCTGCTGC  70
Orcinus orca                   .............GGGGCCGCGTCGGGCCGCCGGTGGCCGCGGCCGCCGCTGCTGTCGTTGCTGCTGCTGCTGCTGCTG.  69
Homo sapiens                   .............GGGGCCGCCTCGGGCCGCCGGGGGCCGGGG...CTGCTGCTGCCGCTGCCGCTGCTGTTGCTGCTG.  66
```

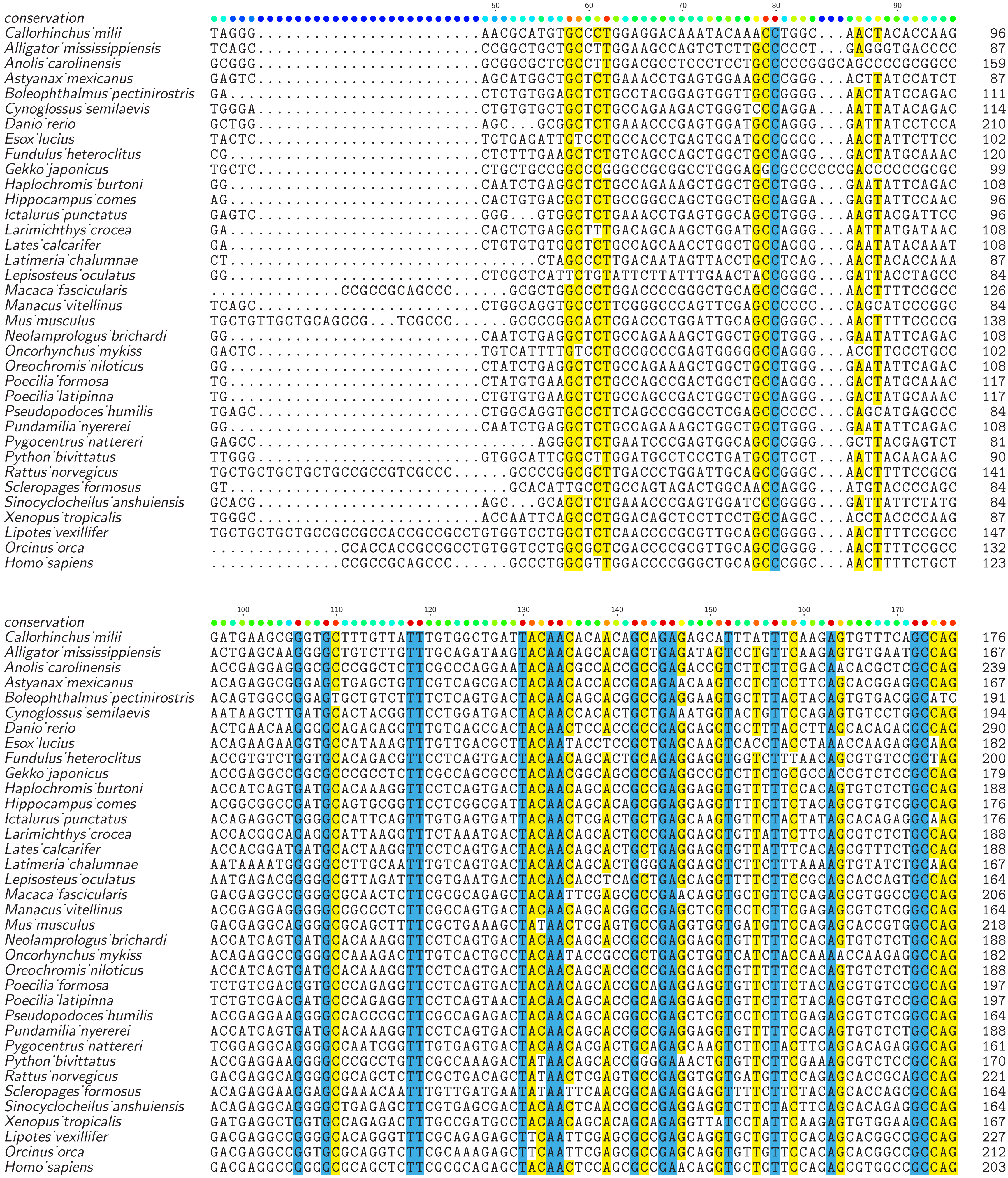
50 60 70 80 90
conservation
Callorhinchus milii TAGGG.........................AACGCATGTGCCCTGGAGGACAAATACAAACCTGGC...AACTACACCAAG 96
Alligator mississippiensis TCAGC.........................CCGGCTGCTGCCTTGGAAGCCAGTCTCTTGCCCCCT...GAGGGTGACCCC 87
Anolis carolinensis GCGGG.........................GCGGCGCTCGCCTTGGACGCCTCCCTCCTGCCCCCGGGCAGCCCCGCGGCC 159
Astyanax mexicanus GAGTC.........................AGCATGGCTGCTCTGAAACCTGAGTGGAAGCCCGGG...ACTTATCCATCT 87
Boleophthalmus pectinirostris GA............................CTCTGTGGAGCTCTGCCTACGGAGTGGTTGCCGGGG...AACTATCCAGAC 111
Cynoglossus semilaevis TGGGA.........................CTGTGTGCTGCTCTGCCAGAAGACTGGGTCCCAGGA...AATTATACAGAC 114
Danio rerio GCTGG.........................AGC...GCGGCTCTGAAACCCGAGTGGATGCCAGGG...GATTATCCTCCA 210
Esox lucius TACTC.........................TGTGAGATTGTCCTGCCACCTGAGTGGATGCCAGGG...AACTATTCTTCC 102
Fundulus heteroclitus CG............................CTCTTTGAAGCTCTGTCAGCCAGCTGGCTGCCAGGG...GACTATGCAAAC 120
Gekko japonicus TGCTC.........................CTGCTGCCGGCCCGGGCCGCGGCCTGGGAGGCGCCCCCCGACCCCCCGCGC 99
Haplochromis burtoni GG............................CAATCTGAGGCTCTGCCAGAAAGCTGGCTGCCTGGG...GAATATTCAGAC 108
Hippocampus comes AG............................CACTGTGACGCTCTGCCGGCCAGCTGGCTGCCAGGA...GAGTATTCCAAC 96
Ictalurus punctatus GAGTC.........................GGG...GTGGCTCTGAAACCTGAGTGGCAGCCTGGG...AAGTACGATTCC 96
Larimichthys crocea GA............................CACTCTGAGGCTTTGACAGCAAGCTGGATGCCAGGG...AATTATGATAAC 108
Lates calcarifer GA............................CTGTGTGTGGCTCTGCCAGCAACCTGGCTGCCAGGG...GAATATACAAAT 108
Latimeria chalumnae CT...............................CTAGCCCTTGACAATAGTTACCTGCCTCAG...AACTACACCAAA 87
Lepisosteus oculatus GG............................CTCGCTCATTCTGTATTCTTATTTGAACTACCGGGG...GATTACCTAGCC 84
Macaca fascicularis ..............CCGCCGCAGCCC......GCGCTGGCCCTGGACCCCGGGCTGCAGCCCGGC...AACTTTTCCGCC 126
Manacus vitellinus TCAGC.........................CTGGCAGGTGCCCTTCGGGCCCAGTTCGAGCCCCCC...CAGCATCCCGGC 84
Mus musculus TGCTGTTGCTGCAGCCG...TCGCCC......GCCCCGGCACTCGACCCTGGATTGCAGCCGGGC...AACTTTTCCCCG 138
Neolamprologus brichardi GG............................CAATCTGAGGCTCTGCCAGAAAGCTGGCTGCCTGGG...GAATATTCAGAC 108
Oncorhynchus mykiss GACTC.........................TGTCATTTTGTCCTGCCGCCCGAGTGGGGGCCAGGG...ACCTTCCCTGCC 102
Oreochromis niloticus GG............................CTATCTGAGGCTCTGCCAGAAAGCTGGCTGCCTGGG...GAATATTCAGAC 108
Poecilia formosa TG............................CTATGTGAAGCTCTGCCAGCCGACTGGCTGCCAGGG...GACTATGCAAAC 117
Poecilia latipinna TG............................CTGTGTGAAGCTCTGCCAGCCGACTGGCTGCCAGGG...GACTATGCAAAC 117
Pseudopodoces humilis TGAGC.........................CTGGCAGGTGCCCTTCAGCCCGGCCTCGAGCCCCCC...CAGCATGAGCCC 84
Pundamilia nyererei GG............................CAATCTGAGGCTCTGCCAGAAAGCTGGCTGCCTGGG...GAATATTCAGAC 108
Pygocentrus nattereri GAGCC...............................AGGGCTCTGAATCCCGAGTGGCAGCCCGGG...GCTTACGAGTCT 81
Python bivittatus TTGGG.........................GTGGCATTCGCCTTGGATGCCTCCCTGATGCCTCCT...AATTACAACAAC 90
Rattus norvegicus TGCTGCTGCTGCTGCCGCCGTCGCCC......GCCCCGGCGCTTGACCCTGGATTGCAGCCGGGC...AACTTTTCCGCG 141
Scleropages formosus GT..............................GCACATTGCCTGCCAGTAGACTGGCAACCAGGG...ATGTACCCCAGC 84
Sinocyclocheilus anshuiensis GCACG.........................AGC...GCAGCTCTGAAACCCGAGTGGATCCCGGGG...GATTATTCTATG 84
Xenopus tropicalis TGGGC.........................ACCAATTCAGCCCTGGACAGCTCCTTCCTGCCAGGC...ACCTACCCCAAG 87
Lipotes vexillifer TGCTGCTGCTGCCGCCGCCACCGCCGCCTGTGGTCCTGGCTCTCAACCCCGCGTTGCAGCCGGGG...AACTTTTCCGCC 147
Orcinus orca ..............CCACCACCGCCGCCTGTGGTCCTGGCGCTCGACCCCGCGTTGCAGCCGGGG...AACTTTTCCGCC 132
Homo sapiens ..............CCGCCGCAGCCC......GCCCTGGCGTTGGACCCCGGGCTGCAGCCCGGC...AACTTTTCTGCT 123
100 110 120 130 140 150 160 170
conservation
Callorhinchus milii GATGAAGCGGGTGCTTTGTTATTTGTGGCTGATTACAACACAACAGCAGAGAGCATTTATTTCAAGAGTGTTTCAGCCAG 176
Alligator mississippiensis ACTGAGCAAGGGGCTGTCTTGTTTGCAGATAAGTACAACAGCACAGCTGAGATAGTCCTGTTCAAGAGTGTGAATGCCAG 167
Anolis carolinensis ACCGAGGAGGGCGCCCGGCTCTTCGCCCAGGAATACAACGCCACCGCCGAGACCGTCTTCTTCGACAACACGCTCGCCAG 239
Astyanax mexicanus ACAGAGGCGGGAGCTGAGCTGTTCGTCAGCGACTACAACACCACCGCCAGAACAAGTCCTCTCCTTCAGCACGGAGGCCAG 167
Boleophthalmus pectinirostris ACAGTGGCCGGAGTGCTGTCTTTCTCAGTGACTACAACAGCACGGCCGAGGAAGTGCTTTACTACAGTGTGACGGCATC 191
Cynoglossus semilaevis AATAAGCTTGATGCACTACGGTTCCTGGATGACTACAACCACACTGCTGAAATGGTACTGTTCCAGAGTGTCCTGGCCAG 194
Danio rerio ACTGAACAAGGGGCAGAGAGGTTTGTGAGCGACTACAACTCCACCGCCGAGGAGGTGCTTTACCTTAGCACAGAGGCCAG 290
Esox lucius ACAGAAGAAGGTGCCATAAAGTTTGTTGACGCTTACAATACCTCCGCTGAGCAAGTCACCTACCTAAACCAAGAGGCAAG 182
Fundulus heteroclitus ACCGTGTCTGGTGCACAGACGTTCCTCAGTGACTACAACAGCACTGCAGAGGAGGTGGTCTTTAACAGCGTGTCCGCTAG 200
Gekko japonicus ACCGAGGCCGGCGCCCCGCCTCTTCGCCAGCGCCTACAACGGCAGCGCCGAGGCCGTCTTCTGCGCCACCGTCTCCGCCAG 179
Haplochromis burtoni ACCATCAGTGATGCACAAAGGTTCCTCAGTGACTACAACAGCACCGCCGAGGAGGTGTTTTTCCACAGTGTCTCTGCCAG 188
Hippocampus comes ACGGCGGCCGATGCAGTGCGGTTCCTCGGCGATTACAACAGCACAGCGGAGGAGGTTTTCTTCTACAGCGTGTCGGCCAG 176
Ictalurus punctatus ACAGAGGCTGGGGCCATTCAGTTTGTGAGTGATTACAACTCGACTGCTGAGCAAGTGTTCTACTATAGCACAGAGGCAAG 176
Larimichthys crocea ACCACGGCAGAGGCATTAAGGTTTCTAAATGACTACAACAGCACTGCCGAGGAGGTGTTATTCTTCAGCGTCTCTGCCAG 188
Lates calcarifer ACCACGGATGATGCACTAAGGTTCCTCAGTGACTACAACAGCACTGCTGAGGAGGTGTTATTTCACAGCGTTTCTGCCAG 188
Latimeria chalumnae AATAAAATGGGGGCCTTGCAATTTGTCAGTGACTACAACAGCACTGGGGAGGAGGTCTTCTTTAAAAGTGTATCTGCAAG 167
Lepisosteus oculatus AATGAGACGGGGGCGTTAGATTTCGTGAATGACTACAACACCTCAGCTGAGCAGGTTTTCTTCCGCAGCACCAGTGCCAG 164
Macaca fascicularis GACGAGGCCGGGGCGCAACTCTTCGCGCAGAGCTACAATTCGAGCGCCGAACAGGTGCTGTTCCAGAGCGTGGCCGCCAG 206
Manacus vitellinus ACCGAGGAGGGGGCCGCCCTCTTCGCCAGTGACTACAACAGCACGGCCGAGCTCGTCCTCTTCGAGAGCGTCTCGGCCAG 164
Mus musculus GACGAGGCAGGGGCGCAGCTTTTCGCTGAAAGCTATAACTCGAGTGCCGAGGTGGTGATGTTCCAGAGCACCGTGGCCAG 218
Neolamprologus brichardi ACCATCAGTGATGCACAAAGGTTCCTCAGTGACTACAACAGCACCGCCGAGGAGGTGTTTTTCCACAGTGTCTCTGCCAG 188
Oncorhynchus mykiss ACAGAGGCCGGGGCCAAAGACTTTGTCACTGCCTACAATACCGCCGCTGAGCTGGTCATCTACCAAAACCAAGAGGCCAG 182
Oreochromis niloticus ACCATCAGTGATGCACAAAGGTTCCTCAGTGACTACAACAGCACCGCCGAGGAGGTGTTTTTCCACAGTGTCTCTGCCAG 188
Poecilia formosa TCTGTCGACGGTGCCCAGAGGTTCCTCAGTGACTACAACAGCACCGCAGAGGAGGTGTTCTTCTACAGCGTGTCCGCCAG 197
Poecilia latipinna TCTGTCGACGATGCCCAGAGGTTCCTCAGTAACTACAACAGCACCGCAGAGGAGGTGTTCTTCTACAGCGTGTCCGCCAG 197
Pseudopodoces humilis ACCGAGGAAGGGGCCACCCGCTTCGCCAGAGACTACAACAGCACGGCCGAGCTCGTCCTCTTCGAGAGCGTCTCGGCCAG 164
Pundamilia nyererei ACCATCAGTGATGCACAAAGGTTCCTCAGTGACTACAACAGCACCGCCGAGGAGGTGTTTTTCCACAGTGTCTCTGCCAG 188
Pygocentrus nattereri TCGGAGGCAGGGGCCAATCGGTTTGTGAGTGACTACAACACGACTGCAGAGCAAGTCTTCTACTTCAGCACAGAGGCCAG 161
Python bivittatus ACCGAGGAAGGGGCCCGCCTGTTCGCCAAAGACTATAACAGCACCGGGGAAACTGTGTTCTTCGAAAGCGTCTCCGCCAG 170
Rattus norvegicus GACGAGGCAGGGGCGCAGCTCTTCGCTGACAGCTATAACTCGAGTGCCGAGGTGGTGATGTTCCAGAGCACCGCAGCCAG 221
Scleropages formosus ACAGAGGAAGGAGCGAAACAATTTGTTGATGAATATAATTCAACGGCAGAGGAGGTTTCTTCTACAGCACCAGCGCCAG 164
Sinocyclocheilus anshuiensis ACAGAGGCAGGGGCTGAGAGCTTCGTGAGCGACTACAACTCAACCGCCGAGGAGGTATCTTCTACTTCAGCACAGAGGCCAG 164
Xenopus tropicalis GATGAGGCTGGTGCCAGAGACTTTGCCGATGCCTACAACAGCACAGCAGAGGTTATCCTATTCAAGAGTGTGGAAGCCAG 167
Lipotes vexillifer GACGAGGCCGGGGCACAGGGTTTCGCAGAGAGCTTCAATTCGAGCGCCGAGCAGGTGCTGTTCCACAGCACGGCCGCCAG 227
Orcinus orca GACGAGGCCGGTGCGCAGGTCTTCGCAAAGAGCTTCAATTCGAGCGCCGAGCAGGTGTTGTTCCACAGCACGGCCGCCAG 212
Homo sapiens GACGAGGCCGGGGCGCAGCTCTTCGCGCAGAGCTACAACTCCAGCGCCGAACAGGTGCTGTTCCAGAGCGTGGCCGCCAG 203

```
                                180           190           200           210           220           230           240           250
conservation
Callorhinchus milii             CTGGAATTACAACACCAATCTGACCAAATATAACCAGCAGAAACAGATCAATGCCTCGCTAGTTGAGCAAGAGTTTACGG   256
Alligator mississippiensis      CTGGAATTACAACACCAACCTGACCGACTTCAACTCCAACCTGCAGATCATGGCCTCGCTGGAGGAGCAGAATTTCACGG   247
Anolis carolinensis             CTGGGTTTACAACACCAACCTCACCGACTTCAACGCCCAGCTGCAGGTGGCAGCTTCTTTGATGGAGCAAGAATTCATAG   319
Astyanax mexicanus              CTGGAATACAACACCAACCTGACCAATGAAAACTCCCTGCAACAGATCCAGGCGTCTATGATAGAGCAAGAATTTACAG   247
Boleophthalmus pectinirostris   ATGGAACTACAACACCAACCTGACGGCGCACAACTCACAGCTGCAGGTCCAGGCCTCCATGGATGAGCAGGCCTTTAGTG   271
Cynoglossus semilaevis          CTGGAACTACAACACCAACATCACACAACACAACTCTCAGCTTCAGGTAATTGCATCTCTTGAAGAACAGGAATTCTCTG   274
Danio rerio                     CTGGAACTACAACACAAACCTGACCGATCACAATTCACAACTGCAGATAGAAGCATCTTTGGAGGAGCAGGCTTTTACAG   370
Esox lucius                     CTGGACTTACCAGACCAACATTACAACTCACAACTCGGATAAAAAGGTGGAATCTGACGGGCTGAAGCAAGCTTTCACAG   262
Fundulus heteroclitus           CTGGAACTACAACACCAACCTTACGGATCACAACTCCAAGCTGCAGGTAGCTGCAAGTCTCGAAGAACAAGCCTTTGATG   280
Gekko japonicus                 CTGGGCCTACAACACCAACCTCACCGCCGCCAACGCCCAGCAGCAGGTGAATTCCTCTTTGGACGAACAGGAGTTTATAC   259
Haplochromis burtoni            CTGGAACTACAACACCAACATAACAGACCACAACTCAAAGCTTCAGGTAAGTGCATCTCTTGAAGAACAGGCATTCGTGG   268
Hippocampus comes               CTGGAACTACAACACCAACTTGACAGAGTACAACTCTCAACTGCAGGTAAACGCATCCTTGGAGGAGCAAGCTTTCATCG   256
Ictalurus punctatus             CTGGGAGCATAAAACCAACCTGACGGAGTACAATTCCCAGCAAGAGGTGCTGGCAAGTCTAGAGAAACAAAATTTCACAG   256
Larimichthys crocea             CTGGAACTACAACACCAACATAACAAGCCACAACTCTGAGCTTCAGGTAAATGCATCTCTTGACGAGCAGGCCTTTGCAT   268
Lates calcarifer                CTGGAACTACAACACCAACATAACAGAGCACAACTCCATGCTTCAGGTAAATGCAGCCCTTGAAGAGCAAGCATTCTCAG   268
Latimeria chalumnae             TTGGATTATAACACAAATCTGACTACATACAACCAGGAAAAACAGATCAATGCATCTTTAGAGGAGCAAGAGTTCTCTG   247
Lepisosteus oculatus            CTGGACATACAACACCGACCTGAATGAACATAACCAGATAGCACAGGTTAACGCCTCAATGGAGGAGCAGGCCTTTGTGG   244
Macaca fascicularis             CTGGGCTCATGACACCAACATCACCGCCGAGAATGCACGGCGCCAGGAGGAAGCAGCCCTGCTCAGCCAGGAGTTTGCCG   286
Manacus vitellinus              CTGGAATTACAACACCAACCTGACGGCGGAAAACGCTGCTCTGCAGGTCCAGGCGTCGCTGGAGGAGCAGAACTTCACGG   244
Mus musculus                    TTGGGCGCACGACACCAACATCACGGAGGAGAACGCGCGACGCCAGGAGGAAGCGGCCCTGGTCAGCCAGGAGTTTGCAG   298
Neolamprologus brichardi        CTGGAACTACAACACCAACATAACAGACCACAACTCAAAGCTTCAGGTAAGTGCATTTCTTGAAGAACAGGCATTTGTGG   268
Oncorhynchus mykiss             CTGGACATACCAGACCAACATTACGCCCCACAACGCAGAGAAAAAGGTGGAATCTGATGGGATGAAGCAGGCCTTCACAG   262
Oreochromis niloticus           CTGGAACTACAACACCAACATAACAGACCACAACTCAAAGCTTCAGGTAAGTGCATCTCTTGAAGAACAGGCATTCGTGG   268
Poecilia formosa                CTGGAACTACAACACCAACCTGACGGAGCACAACTCCAAGCTGCAGGTACTTGCAAGTCTTGAAGAACAAGCATTTGTTG   277
Poecilia latipinna              CTGGAACTACAACACCAACCTGACGGAGCACAACTCCAAGCTGCAGGTACTTGCAAGTCTTGAAGAACAAGCCTTTGTTG   277
Pseudopodoces humilis           CTGGAATTACACCACCAACCTGACGGCGGAAAATGCTGCTCTGCAGGTCAAGGCATCACTGGAGGAGCAGAACTTCACAG   244
Pundamilia nyererei             CTGGAACTACAACACCAACATAACAGACCACAACTCAAAGCTTCAGGTAAGTGCATCTCTTGAAGAACAGGCATTCGTGG   268
Pygocentrus nattereri           CTGGACATACAACACCAACCTGACGGACTACAACTCCCAGCAACAGGTTCTGGCATCTTTGGAGGAGCAAGCATTTAGTG   241
Python bivittatus               CTGGAATTACAACACCAACCTGACAGACGAGAACGCTCAGCTGCAGGTAGCAGCTTCTTTGAAGGAACAAAACTTCACAG   250
Rattus norvegicus               CTGGGCGCACGACACCAACATCACGGAGGAGAATGCGCGGCTCCAGGAGGAAGCGGCCCTGATCAACCAGGAGTTTGCAG   301
Scleropages formosus            CTGGGCATACAACACCAACCTGACTGAAGAGAACTCCAAAATACAGGTTGAAGCCTCCTTAGAGGAACAGGCCTTCACAG   244
Sinocyclocheilus anshuiensis    CTGGAACTACAACACCAACCTGACAGATCACAATTCACAGCTGCAGATTGCTGCATCTTTGGAGGAGCAGGCTTTCACAG   244
Xenopus tropicalis              TTGGGCATATAACACCAACCTGACTGACTACAACTCCAAGCAGCAGATTCTAGCTTCCATGGAGGAGCAGGAGTTTAATG   247
Lipotes vexillifer              CTGGGCGCACGACACCGACATCAACGAGGAAAACGCGCGGCGCCAGGAGGAGGCAGCCCTGCTCAGCCAGGAGTTTTCAG   307
Orcinus orca                    CTGGGCGCACGACACCAACATCACCGAGGAAAACGCGCGGCGCCAGGAGGAGGCAGCCCTAATCAGCCAGGAGTTTTCAG   292
Homo sapiens                    CTGGGCGCACGACACCAACATCACCGCGGAGAATGCAAGGCGCCAGGAGGAAGCAGCCCTGCTCAGCCAGGAGTTTGCGG   283
```

```
                                260           270           280           290           300                310           320           330
conservation
Callorhinchus milii             AAGCCTGGGCCAAGAAAGCCAAGGAATTGTTTGACGACATAAGTAAG...AACTTCAATGATACAGAGCTGAAGAAAATA   333
Alligator mississippiensis      AGGCCTGGGGCAAGAAAGCCAAGCAACTCTATGGCCCCATCTTCAGA...AATTTCACAGACCTACAACTGAAGAAAATC   324
Anolis carolinensis             AGGTTTGGGGGAAAAAAACAAAAGAAATCTATGGCAACATTTGGCAA...AACTTCAGTGATCCTGAACTGAAGAAAATA   396
Astyanax mexicanus              AGGCTTGGGGAATGAAGGCTAAAGCCACCTTTCCTGACGCTCTTATGGAGACCTTCACTAATCCAGATCTCATAAAAATC   327
Boleophthalmus pectinirostris   AAGCCTGGGGTCACAAAGCCAAAACCACATTTTCTGCTGCAGTGCTGAATTCTCTTCCC...AAACAAGACAAAAAATTA   348
Cynoglossus semilaevis          AAGCCTGGGGTAAAAAGGCCAAAGTCACTTTTACAGACGAAACGCTCAACTCACTTTTA...CCTGATAAAAAGAAACTG   351
Danio rerio                     AGGCATGGGGTCACAAAGCAAAGGCTACCTTCAGTGAATCTCTAATGGACACCTTCACTAATCCAGACCTGAAGAAGATC   450
Esox lucius                     AAGCCTGGGGTAAGAAGGCCAAAGTGACCTTTAACCCTGAGCTGCTGGCGACCTTCAAC...ACTACCCTACAGAGGAGA   339
Fundulus heteroclitus           AAGCCTGGGGAATGAAGGCCAAGCAGGTCTTCTCTGAGGATGTACTGAACTCTCTTCCT...GCAAAAGACAAGAAGCTG   357
Gekko japonicus                 AAGCTTGGGGACAGAAAGCAAAGGACAGTTTTGGTGCTATATGGCAG...AACTTCAGTGATCCTCAGCTGAAGAAAATC   336
Haplochromis burtoni            AGGCATGGGGGATCAAGGCTAAGCAGGTCTTCTCTTCTGGAGTTCTCGACACCCTTCCTGACCCTAAAGACAAAAAATTG   348
Hippocampus comes               AGGCCTGGGGGCTGAAGGCCAAGAAGGTGTTCACCCGTGAGCTGCTTGACACCCTTGAC...CCCAAAGATCAGAAACTG   333
Ictalurus punctatus             AAGCATGGGGAAAACTGGCTAAGGAAACATTCCCTGACGCACTCCTGGACACCTTTACTGATCCAACACTGAAGAACCTC   336
Larimichthys crocea             ATGCATGGGGGCTAAAAGCCAGA...GCATTCCCTCAGTCCATTCTCGACACTCTTGAA...CCCAGTGACAAGAAACTG   342
Lates calcarifer                AAGCCTGGGGGTTAAAGGCCAAGCAGGTCTTCACACGTGAGCTTCTCAACTCTCTTTCC...AGCAGTGACAAGAAATTG   345
Latimeria chalumnae             CAGTTTGGGGTGCATCAGCCAAGACA...TTTGATGACATTTTGGAA...ACCTTCCCC...ACTTCGCTAAAGAGGCTC   318
Lepisosteus oculatus            AGGCCTGGGGAAAGAAAGCCAAGGACCTCTTCCCCACCTGGGTGACGGACACCTTTAACGACATTCAGCTCAGGACCCTC   324
Macaca fascicularis             AGGCCTGGGGCCAGAAGGCCAAGGAGCTGTATGAACCGATCTGGCAG...AACTTCACGGACCCGGAGCTGCGCAAGATC   363
Manacus vitellinus              AGCTGTGGGGGAAGAGAGCCAAGGAGCTCTATGGCAACATCTGGAGC...AACTTCACAGACCCACAGCTGAGGAAGATC   321
Mus musculus                    AGGTCTGGGGCAAGAAGGCCAAGGAGTTGTATGAGTCCATTTGGCAG...AACTTTACTGACTCAAAGCTGCGAAGGATC   375
Neolamprologus brichardi        AGGCATGGGGGATCAAGGCTAAGCAGGTCTTCTCTTCTGGAGTTCTCGACACCCTTCCTGACCCTAAAGACAAAAAACTG   348
Oncorhynchus mykiss             AAGCCTGGGGGAAGAAGGCCAAAGATAACTTCTCTCCTACTGTGTTGGCCACCTTCAAC...ACTACTCTGCAGAGGCGT   339
Oreochromis niloticus           AGGCATGGGGGATGAAGGCTAAGCAGGTCTTCTCTTCTGGAGTTCTCGACACCCTTCCTGACCCTAAAGACAAAAAACTG   348
Poecilia formosa                AGGCCTGGGGGGGGAAGGCCAAGAACATCTTCTCTCAGGATACCCTGAACAATCTTACT...ACAGCTGACAAGAAGCTG   354
Poecilia latipinna              AGGCCTGGGGGGGGAAGGCCAAGAACATCTTCTCTCAGGATACCCTGAACGATCTTACT...ACAGCTGACAAGAAGCTG   354
Pseudopodoces humilis           AGTTATGGGGGAAGAAAGCCAAGGAACTCTATGGTGAGAAGTGGAAT...AACTTCAGTGACCCCCAGCTGAGGAAAATC   321
Pundamilia nyererei             AGGCATGGGGGATCAAGGCTAAGCAGGTCTTCTCTTCTGGAGTTCTCAACACCCTTCCTGACCCTAAAGACAAAAAATTG   348
Pygocentrus nattereri           AGGCCTGGGGACAGAAGGCTAAGACGACCTTCAGTGATGCACTCATGGACACCTTCACCAATCAGGAACTGAAGACACTC   321
Python bivittatus               AACTCTGGGGGAAAAAAGCTAAGGAGAATTATGGCCACAACTGGCAG...AACTTCAAGGATCCTCAACTGAAGAAAATA   327
Rattus norvegicus               AGGTCTGGGGCAAGAAGGCCAAGGAGCTGTATGAGTCCATCTGGCAG...AACTTCACTGACCAAAAGCTGCGAAGGATC   378
Scleropages formosus            AAGCCTGGGGGAAGAAGGCGAAAGAAGTCTTTCCTGAGGCTGTGCTGGACACGTTCATAGACACGCAGCTTAAGAAACTC   324
Sinocyclocheilus anshuiensis    AAGCATGGGGTCAAAAAGCAAAGGCTACCTTCAGTGATTCTCTCATGGATACCTTCACTAATCCAGACCTGAAGAAAATC   324
Xenopus tropicalis              AGGCATGGGGTATGAAGGCTAAGGAACTTTTCAATGATGTGTGGGAA...AACTTTACAGATCCATTACTCAAGAAAATT   324
Lipotes vexillifer              AGGTCTGGGGCCAGAAGGCCAAGGCTCTGTTCGACCCAATCTGGCAG...AACTTCACCGACCCCACGCTGCGACGCATC   384
Orcinus orca                    AGGTCTGGGGCCAGAAGGCCAAGGCTCTGTTCGACCCAATCTGGCAG...CACTTCACCGACCCCACGCTGCGACGCATC   369
Homo sapiens                    AGGCCTGGGGCCAGAAGGCCAAGGAGCTGTATGAACCGATCTGGCAG...AACTTCACGGACCCGCAGCTGCGCAGGATC   360
```

```
                                  340       350       360       370       380       390       400       410
conservation
Callorhinchus milii              CTGAGAGAGATTCGAATCCTAGGCCCTGCAAATCTTGACCCACAAAAGAGAGAGAAGTACAACACCATCCTGACTGAAAT   413
Alligator mississippiensis       ATTGGCTCCATCCAGACCTTGGGGCCTTCCAACCTGCCCATGAACAAGCGGGAGCAGTACAACACCATTTTGAGTGAGAT   404
Anolis carolinensis              ATCAGTAACATCCAGACATTAGGCCCCTCCAATTTGGATCTGGAAAAGCGGCAGCGGTATAACACTATAATGAGTGAGAT   476
Astyanax mexicanus               ATAAAGAAGATCAATGTGCTCGGAGCCGCCAACCTCCCAACTGCAGAGAGAGAGAGGTACAACACAATTCTGAGTCAGAT   407
Boleophthalmus pectinirostris    TATGACAAGATCGTTGTCCTGGGACCTGCCAACCTGCCCACCGCCGAAAGAGAGGAGTACAACACCATCCTCAGCACCAT   428
Cynoglossus semilaevis           ATGGAAAAAATCAAGGTACTGGGAGCCGCCAACCTTGGCCCAGACGAAAGGAAAGAGTACAACACCATCCTCAGCACCAT   431
Danio rerio                      ATCAAAAAGATCAATGTCCTGGAAGCCGCCAATCTCGCAATCACAGACAGAGAGCTGTACAACACTATTCTGAGCCAGAT   530
Esox lucius                      ATCCACAAGATCAACATTCTGGGTCCAGCTAACCTTCCTGCAGCAGAAAGAAATGAGTACAATCGCATCCTTAGTGAAAT   419
Fundulus heteroclitus            ATTAAAAAGATCACGTCACTGGGAGTTGCCAACCTTCCACTTGCTGAACGAGAGGAGTATAACACCATCCTAAGCACCAT   437
Gekko japonicus                  ATCCGCTCCGTCCAAACATTGGGGCCTTCTAATTTACCCATGGATAAACGACAACTGTACAACACCATCTTGAGTGAGAT   416
Haplochromis burtoni             ATGGAAAAGATCAAGCTACTCGGTGCTGCCAACCTTCTCCCGGAAGAAAGAGAGGAGTATAACACCATTCTGAGCACCAT   428
Hippocampus comes                ATGAGCAACGTCATGATCCTCGGAGCCGCCAACCTGCCTCCGGAAGAAAGAGGAGAGTACAACACCATTCTCAGCACCAT   413
Ictalurus punctatus              ATCAAAAAGATTGATGTCCTAGGACCTGCCAACCTCCCTACCACAGAGCGAGAGAGGTACAATTTGATTCTGAGTCAGAT   416
Larimichthys crocea              ATGAAAAAGATCACGCTTCTGGGAGCTGCCAACCTGGCTCCAAAAGAAAGAGAGGAGTACAACACAATTCTGAGCACCAT   422
Lates calcarifer                 ATGGAAAAGATCATGATGCTAGGAGCTGCCAACCTGCTCCAGAAAGAAAGAGAGGAGTACAACACCATTCTCAGCACTAT   425
Latimeria chalumnae              ATTAGACAGATTAGAGTCCTAGGAGTGGCTAACTTAAAAGTAGAAGAAAGACAAAAGTACAACACTTTATTGAGCCAAAT   398
Lepisosteus oculatus             ATAAAGAAGATCAGTGTCCTGGGAGCAGCTAACCTCAACACCACGGAGCGAAAGAACTACAACACCATCTTGAGTGACAT   404
Macaca fascicularis              ATCGGAGCTGTGGGCACCCTGGGCTCTGCCAACCTGCCCCTGGCTAAGCGGCAGCAGTACAATGCCCTGCTAAGCAACAT   443
Manacus vitellinus               ATCGGCTCCATCCAGACCCTGGGACCCTCCAACCTGCCCCTGGACAAGAGAGAGCAGTACAACACCATCCTGAGCGACAT   401
Mus musculus                     ATCGGATCTATTCGGACCCTAGGACCTGCCAATCTGCCCCTGGCCCAGCGGCAGCAGTACAACTCTCTGCTAAGCAACAT   455
Neolamprologus brichardi         ATGGAAAAGATCAAGCACCTTGGTGCTGCCAACCTTCTACAGGAAGAAAGAGAGGAGTATAACACCATTCTGAGCACCAT   428
Oncorhynchus mykiss              CTCAAGAAGATCAACATCCTGGGAGCGGCTAACCTACCTGCAGGAGAAAGAAATGAGTACAACGTCATACTAAGCAAGAT   419
Oreochromis niloticus            ATGGAAAAGATCAAGCTACTCGGTGCTGCCAACCTTCTCCCGGAAGAAAGAGAGAAGTATAACACCATTCTGAGCACCAT   428
Poecilia formosa                 ATTGAAAAGATCAAGTCACTGGGAGCTGCCAACCTTCCACAGGCTGAACGAGAGGAGTATAACACCATCCTGAGCACCAT   434
Poecilia latipinna               ATTGAAAAGATCAAGTCACTGGGAGCTGCCAACCTTCCACAGGCTGAACGAGAGGAGTATAACACCATCCTGAGCACCAT   434
Pseudopodoces humilis            ATTGGATCCATCCAGACCTTGGGACCCTCCAACCTGCCCCTGGAGATGAGAGAGCAGTATAACACCATCCTGAGTGACAT   401
Pundamilia nyererei              ATGGAAAAGATCATGCTACTCGGTGCTGCCAACCTTCTCCCGGAAGAAAGAGAGGAGTATAACACCATTCTGAGCACCAT   428
Pygocentrus nattereri            ATCAAAAAGATCAATGTGCTGGGACCTGCCAACCTCGTTCAAACGGAAAGAGAAAGGTACAATACCATTTTGAGTCAGAT   401
Python bivittatus                ATCAGCTCCATTCAGACCCTGGGCCCTTCCAATTTACCAATGAACAAGCGACACGAGTATAATACTGTTCTGAGTGAGAT   407
Rattus norvegicus                ATCGGATCCGTACAGACCCTAGGACCTGCCAACCTGCCCCTGACCCAGCGGCTGCAGTACAACTCTCTGCTAAGCAACAT   458
Scleropages formosus             ATAAAGGGTATCAGTATCCTCGGCCCTGCTAATCTAGCTCCAGCAGAGAGAGAGAAGTACAACAGCATTCTGAGTAAGAT   404
Sinocyclocheilus anshuiensis     ATCAATAATATCAATGTTCTGGGACCCGCCAATCTCCCAACTGCTGAGAGAGAGAAGTACAATACCATTCTGAGCCAGAT   404
Xenopus tropicalis               ATCAGTTCTATAAGGACCCTCGGAGCATCAAACTTGAACTTGACTGCAAGAGAGGAGTATAACACCATTCTAAGCGAGAT   404
Lipotes vexillifer               ATCAGTGCCGTGCGCACCCTGGGCCCCGCCAACCTGCCCCTGGAGGAGCGGCAGCAGTACAACTCTCTGCTAAGCAACAT   464
Orcinus orca                     ATCAGTGCCGTGCGCACCCTGGGCCCTGCCAACCTGCCCCTGGAGGAGCGGCAGCAGTACAACTCTCTGCTAAGCAACAT   449
Homo sapiens                     ATCGGAGCTGTGCGCACCCTGGGCTCTGCCAACCTGCCCCTGGCTAAGCGGCAGCAGTACAACGCCCTGCTAAGCAACAT   440

                                      420       430       440       450       460       470       480       490
conservation
Callorhinchus milii              GGATAATATCTATTCCACAGCGAAGGTGTGTCCCCCAAGCCAAGAGAATATCACTAAGTGCTGGTCGCTTGAACCGGATC   493
Alligator mississippiensis       GGACAATATCTATTCCACTGCCAAGGTGTGTCCTCCACACCAGATG......TCCAACTGCTGGTCATTGGAACCAGAGG   478
Anolis carolinensis              GGACAACATCTATTCCACTTCCAAGGTGTGTCTACCAAACCAGACC......TCCAATTGCTGGGCTCTAGAACCAGATA   550
Astyanax mexicanus               GGACAACATCTACTCCACGGCAAAAGTGTGTCCT......AAACCA......GAGGAGTGCTGGGCTTTGGAACCTGAGC   475
Boleophthalmus pectinirostris    GGACAACATTTACTCCACAGCCAAAGTCCACCCA......CAAGAA......AATGTTAGCTGGAGTCTGGAGCCAGAAC   496
Cynoglossus semilaevis           GGACAGTATCTATTCAACAGCAAAGGTGCATCCA......TCCCCA......AATGTCAGCTGGAGCCTAGAACCTGAAC   499
Danio rerio                      GGACAGCATCTACTCCACTGCAAAGGTGTGTCCC......TCTCCA......GAGGAATGCTGGTCTTTGGAGCCTGAGC   598
Esox lucius                      GTCTTCAATATACTCCACTGCCAAAGTGTGTCCA......AAGCCT......GAGGAATGCTGGTCTCTCGAGCCAGAGC   487
Fundulus heteroclitus            GGACAGTATTTATTCAACAGCTAAGGTGTACCCA......CAGCCA......AACATTAGCTGGAGCCTGGATCCTGAAC   505
Gekko japonicus                  GGACAACATCTACTCAACTGCAAAAGTGTGCCCACCAAACCAGAAC......TCCAGCTGTTGGGCCCTTGATCCAGAGA   490
Haplochromis burtoni             GGACAATATTTATTCGACATCTAAGGTGCACCCA......CAGCCA......AACATCAGCTGGAGTCTGGAACCTGAGC   496
Hippocampus comes                GGACAGTATTTATTCAACCGCTAAAGTGCATCCG......CAGCCC......AACGTCAGCTGGAGCCTGGAACCTCATC   481
Ictalurus punctatus              GGGCAGCATCTACTCCACGGCAAAAGTATGTCCC......AAACCA......GCGGAGTGCTGGTCTTTGGAGCCTGAGT   484
Larimichthys crocea              GGACAATATTTATTCAACAGCCAAAGTGCATCCA......CAGCCA......AACATAAGCTGGAGCCTGGAACCTGAAC   490
Lates calcarifer                 GGACAATATTTATTCAACATCCAAGGTGCATCCA......GAGCCA......AACGTAAGCTGGAGCTTGGAACCCCATC   493
Latimeria chalumnae              GGACAATATTTACTCCACTGCTAAAGTGTGTCCACCAAATCAAAGT......ACTGGCTGCTGGTCCTTGGAACCTGATC   472
Lepisosteus oculatus             GGACAGTATTTATTCTACTGCTAATGTGTGTCCACCTGGAGAGAAT......ATTAAGTGCTGGTCCCTTGAGCCCGACA   478
Macaca fascicularis              GAGCAGGATCTACTCCACCGCCAAGGTCTGCCTCCCCAACAAGACT......GCCACCTGCTGGTCCCTGGACCCAGACC   517
Manacus vitellinus               GGACAAGATCTACTCCACGGCCAAGGTGTGCCTGCCCAAC............GGCACCTGCTGGGATCTGGAGCCAGACC   469
Mus musculus                     GAGCAGAATCTACTCCACTGGCAAGGTCTGCTTCCCCAACAAGACT......GCCACCTGCTGGTCCCTGGACCCAGAGC   529
Neolamprologus brichardi         GGACAATATTTATTCGACATCTAAGGTGCACCCA......CAGCCA......AACATCAGCTGGAGTCTGGAACCTGAGC   496
Oncorhynchus mykiss              GTCTGAAATATACTCTACCGCCAAAGTGTGTCCA......AAGACT......AATGAATGCTGGTCTATTGAGCCAGAGC   487
Oreochromis niloticus            GGACAATATTTATTCGACATCTAAGGTGCACCCA......CAGCCA......AACATCAGCTGGAGTCTGGAACCTGAGC   496
Poecilia formosa                 GGACAGCATTTATTCAACAGCTAAGGTGTACCCA......CAGCCA......GACATAAGCTGGAGCCTGGAACCTGAAC   502
Poecilia latipinna               GGACAGCATTTATTCAACAGCTAAGGTGTACCCA......CAGCCA......GACATAAGCTGGAGCCTGGAACCTGAAC   502
Pseudopodoces humilis            GGACAAAATCTACTCCACGGCCAAGGTGTGCCTGCCCAAT............GGCACCTGCTGGGATCTGGAGCCAGACC   469
Pundamilia nyererei              GGACAATATTTATTCGACATCTAAGGTGCACCCA......CAGCCA......AACATCAGCTGGAGTCTGGAACCTGAGC   496
Pygocentrus nattereri            GGACAACATCTACTCCACTGCAAAACTGTGTCCT......AAAGAA......GGAGAGTGCTGGTCTTTGGAACCTGAGT   469
Python bivittatus                GGACAACATCTACTCCACTTCCAAGGTTTGTCCACCAAACCAGAGC......TCCAGCTGCTGGGCCTTAGAGCCAGATA   481
Rattus norvegicus                GAGCAGAATCTACTCCACCGGCAAGGTCTGCTTCCCCAACAAGACT......GCCACCTGCTGGTCCCTGGACCCAGAGC   532
Scleropages formosus             GGACAACATATACTCCACAGCAAAAGTGTGCCCCCCAGGACAGGAG......ATAAACTGCTGGTCATTGGAGCCCGAGC   478
Sinocyclocheilus anshuiensis     GGACAGCATCTACTCAACTGCAAAGGTGTGTCCT......TCA.........GAGGAATGCTGGTCTTTGGAGCCTGAGC   469
Xenopus tropicalis               GGACAGCATCTATTCAACAGCCAAAGTTTGCCCTCCCAATGCAACT......GCGAAATGCTGGTCTTTGGAACCCGAAA   478
Lipotes vexillifer               GTCCAGGATTTACTCCACGGCCAAGGTCTGCTTCCCCAACAAGACT......GCCACCTGCTGGTCCCTGGACCCAGAGC   538
Orcinus orca                     GTCCAGGATTTACTCCACGGCCAAGGTCTGCTTCCCCAACAAGACT......GCCACCTGCTGGTCCCTGGACCCAGAGC   523
Homo sapiens                     GAGCAGGATCTACTCCACCGCCAAGGTCTGCCTCCCCAACAAGACT......GCCACCTGCTGGTCCCTGGACCCAGATC   514
```

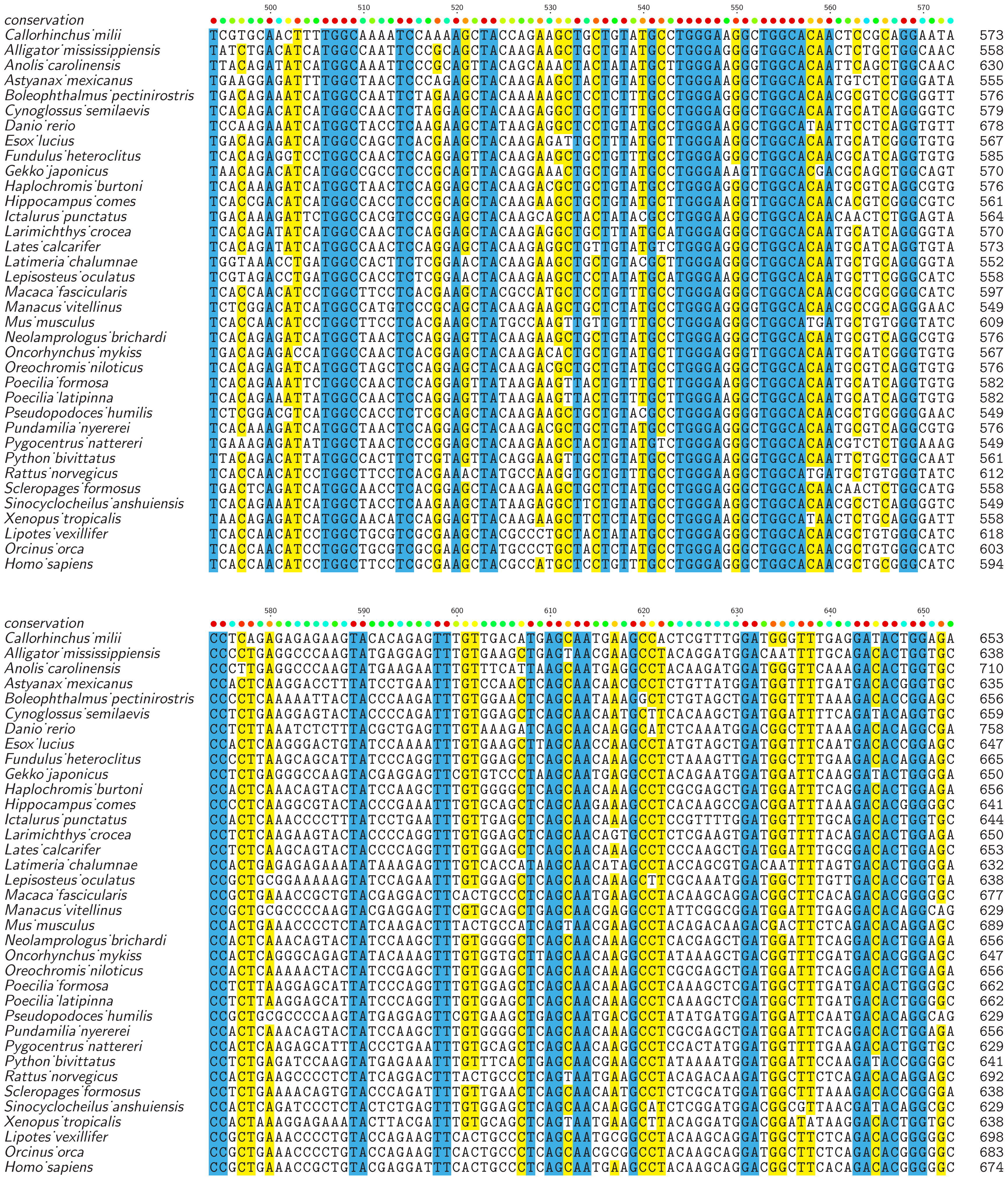

500 510 520 530 540 550 560 570
conservation
Callorhinchus milii TCGTGCAACTTTTGGCAAAATCCAAAAGCTACCAGAAGCTGCTGTATGCCTGGGAAGGCTGGCACAACTCCGCAGGAATA 573
Alligator mississippiensis TATCTGACATCATGGCCAATTCCCGCAGCTACAAGAAGCTGCTGTATGCCTGGGAGGGGTGGCACAACTCTGCTGGCAAC 558
Anolis carolinensis TTACAGATATCATGGCAAATTCCCGCAGTTACAGCAAACTACTATATGCTTGGGAGGGTGGCACAATTCAGCTGGCAAC 630
Astyanax mexicanus TGAAGGAGATTTTGGCTAACTCCCAGAGCTACAAGAAGCTACTGTATGCCTGGGAAGGCTGGCACAATGTCTCTGGGATA 555
Boleophthalmus pectinirostris TGACAGAAATCATGGCCAATTCTAGAAGCTACAAAAAGCTCCTCTTTGCCTGGGAGGGCTGGCACAACGCGTCCGGGGTT 576
Cynoglossus semilaevis TCACAGACATCATGGCCAACTCTAGGAGCTACAAGAGGCTGCTGTTTGCCTGGGAGGGCTGGCACAATGCATCAGGGGTC 579
Danio rerio TCCAAGAAATCATGGCTACCTCAAGAAGCTATAAGAGGCTCCTGTATGCCTGGGAAGGCTGGCATAATTCCTCAGGTGTT 678
Esox lucius TGACAGAGATCATGGCCAGCTCACGAAGCTACAAGAGATTGCTTTATGCTTGGGAAGGCTGGCACAATGCATCGGGTGTG 567
Fundulus heteroclitus TCACAGAGGTCCTGGCCAACTCCAGGAGCTACAAGAAGCTGCTGTTTGCCTGGGAGGGCTGGCACAACGCATCAGGTGTG 585
Gekko japonicus TAACAGACATCATGGCCGCCTCCCGCAGTTACAGGAAACTGCTGTATGCCTGGGAAAGTTGGCACGACGCAGCTGGCAGT 570
Haplochromis burtoni TCACAAAGATCATGGCTAACTCCAGGAGCTACAAGACGCTGCTGTATGCCTGGGAGGGCTGGCACAATGCGTCAGGCGTG 576
Hippocampus comes TCACCGACATCATGGCCACCTCCCGCAGCTACAAGAAGCTGCTGTATGCTTGGGAAGGTTGGCACAACACGTCGGGCGTC 561
Ictalurus punctatus TGACAAAGATTCTGGCCACGTCCCGGAGCTACAAGCAGCTACTATACGCCTGGGAAGGCTGGCACAACAACTCTGGAGTA 564
Larimichthys crocea TCACAGATATCATGGCCAACTCCAGGAGCTACAAGAGGCTGCTTTATGCATGGGAGGGCTGGCACAATGCATCAGGGGTA 570
Lates calcarifer TCACAGATATCATGGCCAACTCCAGGAGCTACAAGAGGCTGTTGTATGTCTGGGAGGGCTGGCACAATGCATCAGGTGTA 573
Latimeria chalumnae TGGTAAACCTGATGGCCACTTCTCGGAACTACAAGAAGCTGCTGTACGCTTGGGAGGGCTGGCACAATGCTGCAGGGGTA 552
Lepisosteus oculatus TCGTAGACCTGATGGCCACCTCTCGGAACTACAAGAAGCTCCTATATGCATGGGAAGGCTGGCACAATGCTTCGGGCATC 558
Macaca fascicularis TCACCAACATCCTGGCTTCCTCACGAAGCTACGCCATGCTCCTGTTTGCCTGGGAGGGCTGGCACAACGCCGCGGGCATC 597
Manacus vitellinus TCTCGGACATCATGGCCATGTCCCGCAGCTACAAGAAGCTGCTCTATGCCTGGGAGGGGTGGCACAACGCCGCAGGGAAC 549
Mus musculus TCACCAACATCCTGGCTTCCTCACGAAGCTATGCCAAGTTGTTGTTTGCCTGGGAGGGCTGGCATGATGCTGTGGGTATC 609
Neolamprologus brichardi TCACAGAGATCATGGCTAACTCCAGGAGTTACAAGAAGCTGCTGTATGCCTGGGAGGGCTGGCACAATGCGTCAGGCGTG 576
Oncorhynchus mykiss TGACAGAGACCATGGCCAACTCACGGAGCTACAAGACACTGCTGTATGCTTGGGAGGGTTGGCACAATGCATCGGGTGTG 567
Oreochromis niloticus TCACAGAGATCATGGCTAGCTCCAGGAGCTACAAGACGCTGCTGTATGCCTGGGAGGGCTGGCACAATGCGTCAGGTGTG 576
Poecilia formosa TCACAGAAATTCTGGCCAACTCCAGGAGTTATAAGAAGTTACTGTTTGCTTGGGAAGGCTGGCACAATGCATCAGGTGTG 582
Poecilia latipinna TCACAGAAATTATGGCCAACTCCAGGAGTTATAAGAAGTTACTGTTTGCTTGGGAAGGCTGGCACAATGCATCAGGTGTG 582
Pseudopodoces humilis TCTCGGACGTCATGGCCACCTCTCGCAGCTACAAGAAGCTGCTGTACGCCTGGGAGGGGTGGCACAACGCTGCGGGGAAC 549
Pundamilia nyererei TCACAAAGATCATGGCTAACTCCAGGAGCTACAAGACGCTGCTGTATGCCTGGGAGGGCTGGCACAATGCGTCAGGCGTG 576
Pygocentrus nattereri TGAAAGAGATATTGGCTAACTCCCGGAGCTACAAGAAGCTACTGTATGTCTGGGAGGGCTGGCACAACGTCTCTGGAAAG 549
Python bivittatus TTACAGACATTATGGCCACTTCTCGTAGTTACAGGAAGTTGCTGTATGCCTGGGAAGGGTGGCACAATTCTGCTGGCAAT 561
Rattus norvegicus TCACCAACATCCTGGCTTCCTCACGAAACTATGCCAAGGTGCTGTTTGCCTGGGAAGGCTGGCATGATGCTGTGGGTATC 612
Scleropages formosus TGACTCAGATCATGGCAACCTCACGGAGCTACAAGAAGCTGCTCTATGCCTGGGAGGGCTGGCACAACAACTCTGGCATG 558
Sinocyclocheilus anshuiensis TCACAGAAATCATGGCTACCTCAAGAAGCTATAAGAGGCTTCTGTATGCCTGGGAAGGCTGGCACAACGCCTCAGGGGTC 549
Xenopus tropicalis TAACAGAGATCATGGCAACATCCAGGAGTTACAAGAAGCTTCTCTATGCCTGGGAAGGCTGGCATAACTCTGCAGGGATT 558
Lipotes vexillifer TCACCAACATCCTGGCTGCGTCGCGAAGCTACGCCCTGCTACTATATGCCTGGGAGGGCTGGCACAACGCTGTGGGCATC 618
Orcinus orca TCACCAACATCCTGGCTGCGTCGCGAAGCTATGCCCTGCTACTCTATGCCTGGGAGGGCTGGCACAACGCTGTGGGCATC 603
Homo sapiens TCACCAACATCCTGGCTTCCTCGCGAAGCTACGCCATGCTCCTGTTTGCCTGGGAGGGCTGGCACAACGCTGCGGGCATC 594
580 590 600 610 620 630 640 650
conservation
Callorhinchus milii CCTCAGAGAGAGAAGTACACAGAGTTTGTTGACATGAGCAATGAAGCCACTCGTTTGGATGGGTTTGAGGATACTGGAGA 653
Alligator mississippiensis CCCCTGAGGCCCAAGTATGAGGAGTTTGTGAAGCTGAGTAACGAAGCCTACAGGATGGACAATTTTGCAGACACTGGTGC 638
Anolis carolinensis CCCTTGAGGCCCAAGTATGAAGAATTTGTTTCATTAAGCAATGAGGCCTACAAGATGGATGGGTTCAAAGACACTGGTGC 710
Astyanax mexicanus CCACTCAAGGACCTTTATCCTGAATTTGTCCAACTCAGCAACAACGCCTCTGTTATGGATGGTTTTGATGACACGGGTGC 635
Boleophthalmus pectinirostris CCCCTCAAAAATTACTACCCAAGATTTGTGGAACTCAGCAATAAAGGCTCTGTAGCTGATGGTTTTAAAGACACCGGAGC 656
Cynoglossus semilaevis CCTCTGAAGGAGTACTACCCCAGATTTGTGGAGCTCAGCAACAATGCTTCACAAGCTGATGGATTTTCAGATACAGGTGC 659
Danio rerio CCTCTTAAATCTCTTTACGCTGAGTTTGTAAAGATCAGCAACAAGGCATCTCAAATGGACGGCTTTAAAGACACAGGCGA 758
Esox lucius CCACTCAAGGGACTGTATCCAAAATTTGTGAAGCTTAGCAACCAAGCCTATGTAGCTGATGGTTTCAATGACACCGGAGC 647
Fundulus heteroclitus CCCCTTAAGCAGCATTATCCCAGGTTTGTGGAGCTCAGCAACAAAGCCTCTAAAGTTGATGGCTTTGAAGACACAGGAGC 665
Gekko japonicus CCTCTGAGGGCCAAGTACGAGGAGTTCGTGTCCCTAAGCAATGAGGCCTACAGAATGGATGGATTCAAGGATACTGGGGA 650
Haplochromis burtoni CCACTCAAACAGTACTATCCAAGCTTTGTGGGGCTCAGCAACAAAGCCTCGCGAGCTGATGGATTTCAGGACACTGGAGA 656
Hippocampus comes CCCCTCAAGGCGTACTACCCGAAATTTGTGCAGCTCAGCAAGAAAGCCTCACAAGCCGACGGATTTAAAGACACGGGGGC 641
Ictalurus punctatus CCACTCAAACCCCTTTATCCTGAATTTGTTGAGCTCAGCAACAAAGCCTCCGTTTTGGATGGTTTTGCAGACACTGGTGC 644
Larimichthys crocea CCTCTCAAGAAGTACTACCCCAGGTTTGTGGAGCTCAGCAACAGTGCCTCTCGAAGTGATGGTTTTACAGACACTGGAGA 650
Lates calcarifer CCTCTCAAGCAGTACTACCCCAGGTTTGTGGAGCTCAGCAACAAAGCCTCCCAAGCTGATGGATTTGCGGACACTGGAGC 653
Latimeria chalumnae CCACTGAGAGAGAAATATAAAGAGTTTGTCACCATAAGCAACATAGCCTACCAGCGTGACAATTTTAGTGACACTGGGGA 632
Lepisosteus oculatus CCGCTGCGGAAAAAAGTATCCAGAATTTGTGGAGCTCAGCAACAAAGCTTCGCAAATGGATGGCTTTGTTGACACCGGTGA 638
Macaca fascicularis CCGCTGAAACCGCTGTACGAGGACTTCACTGCCCTCAGCAATGAAGCCTACAAGCAGGACGGCTTCACAGACACGGGGGC 677
Manacus vitellinus CCGCTGCGCCCCAAGTACGAGGAGTTCGTGCAGCTGAGCAACGAGGCCTATTCGGCGGATGGATTTGAGGACACAGGCAG 629
Mus musculus CCACTGAAACCCCTCTATCAAGACTTTACTGCCATCAGTAACGAAGCCTACAGACAAGACGACTTCTCAGACACAGGAGC 689
Neolamprologus brichardi CCACTCAAACAGTACTATCCAAGCTTTGTGGGGCTCAGCAACAAAGCCTCACGAGCTGATGGATTTCAGGACACTGGAGA 656
Oncorhynchus mykiss CCACTCAGGGCAGAGTATACAAAGTTTGTGGTGCTTAGCAACAAGGCCTATAAAGCTGACGGTTTCGATGACACGGGAGC 647
Oreochromis niloticus CCACTCAAAAACTACTATCCGAGCTTTGTGGAGCTCAGCAACAAAGCCTCGCGAGCTGATGGATTTCAGGACACTGGAGA 656
Poecilia formosa CCTCTTAAGGAGCATTATCCCAGGTTTGTGGAGCTCAGCAACAAAGCCTCAAAGCTCGATGGCTTTGATGACACTGGGGC 662
Poecilia latipinna CCTCTTAAGGAGCATTATCCCAGGTTTGTGGAGCTCAGCAACAAAGCCTCAAAGCTCGATGGCTTTGATGACACTGGGGC 662
Pseudopodoces humilis CCGCTGCGCCCCAAGTATGAGGAGTTCGTGAAGCTCAGCAATGACGCCTATATGATGGATGGATTCAATGACACAGGCAG 629
Pundamilia nyererei CCACTCAAACAGTACTATCCAAGCTTTGTGGGGCTCAGCAACAAAGCCTCGCGAGCTGATGGATTTCAGGACACTGGAGA 656
Pygocentrus nattereri CCACTCAAGAGCATTTACCCTGAATTTGTGCAGCTCAGCAACAAGGCCTCCACTATGGATGGTTTTGAAGACACTGGTGC 629
Python bivittatus CCTCTGAGATCCAAGTATGAGAAATTTGTTTCACTGAGCAACGAAGCCTATAAAATGGATGGATTCCAAGATACCGGGGC 641
Rattus norvegicus CCACTGAAGCCCCTCTATCAGGACTTTACTGCCCTCAGTAATGAAGCCTACAGACAAGATGGCTTCTCAGACACAGGAGC 692
Scleropages formosus CCTCTGAAAACAGTGTACCCAGATTTTGTTGAACTCAGCAACAATGCCTCTCGCATGGATGGCTTTAAAGACACCGGGGA 638
Sinocyclocheilus anshuiensis CCACTCAGATCCCTCTACTCTGAGTTTGTGGAGCTCAGCAACAAGGCATCTCGGATGGACGGCGTTAACGATACAGGCGC 629
Xenopus tropicalis CCACTAAAGGAGAAATACTTACGATTTGTGCAGCTCAGTAATGAAGCTTACAGGATGGACGGATATAAGGACACTGGTGC 638
Lipotes vexillifer CCGCTGAAACCCCTGTACCAGAAGTTCACTGCCCTCAGCAATGCGGCCTACAAGCAGGATGGCTTCTCAGACACGGGGGC 698
Orcinus orca CCGCTGAAACCCCTGTACCAGAAGTTCACTGCCCTCAGCAACGCGGCCTACAAGCAGGATGGCTTCTCAGACACGGGGGC 683
Homo sapiens CCGCTGAAACCGCTGTACGAGGATTTCACTGCCCTCAGCAATGAAGCCTACAAGCAGGACGGCTTCACAGACACGGGGGC 674

	660 670 680 690 700 710 720 730	
conservation		
Callorhinchus milii	GTACTGGAGATCATGGTATGAATCGCCAACATTTGAGGAAGATCTTGAATTCATCTACCACCAGTTGAAACCTCTCTACC	733
Alligator mississippiensis	TTACTGGCGCTCCTGGTATGACTCACCCACTTTGAGGATGATCTGGAACAACTCTACCATCAGCTGGAACCCCTCTACC	718
Anolis carolinensis	ATATTGGCGGTCCTCATATGACTCACCAACATTTGAGGATGATCTGGAAGAACTCTATCATCAGTTGGAGCCACTCTATC	790
Astyanax mexicanus	TTACTGGCGTTCCTGGTACGAGTCTCCTACCTTTGAGCAGGACTTAGAAAACCTGTTTAAACAACTGCAGCCGCTTTACC	715
Boleophthalmus pectinirostris	GTACTGGAGGTCTTGGTATGAAAGCCCAACCTTTCAAGAGGATTTGAGGAGTCTTTACCAAACAGTGGAACCCCTCTACC	736
Cynoglossus semilaevis	AGACTGGAGATCGTGGTACGAGACTGAGACTTTTGAAAAGGACTTGGAAGAACTTTACAGGACCATTGAGCCACTGTACA	739
Danio rerio	GTACTGGCGCTCTTGGTACGAGTCTCCTACCTTCAAGCAAGACCTTGAGAACCTGTTTAAACAGCTGGAACCTCTCTACC	838
Esox lucius	ATATTGGCGTTCGTGGTATGAGTCCAGTTCCTTTGAGAATGACCTGGAGGTTATTTACAAACAGGTGCAGCCACTCTACC	727
Fundulus heteroclitus	TTATTGGCGCTCCTGGTATGAGACTGAAACCTTTCAGGAGGATTTAGAGAAGCTCTACAAGACGATTGAACCGCTCTACC	745
Gekko japonicus	TTACTGGCGCTCCTGGTATGATTCACCCACTTTTGAGGATGATCTGGAGAAGCTTTATCATCAGCTGGAGGAGCTCTACC	730
Haplochromis burtoni	TGACTGGCGCTCTTGGTATGAGACTGAAACTTTTAGGCAGGACTTGGAAGAAATCTACAAGACCATTGAGCCGCTCTACC	736
Hippocampus comes	CTACTGGCGCTCTTGGTACGAGACGGACAACTTCCAGGAAGTCATCGAGCAGCTGTACAAAACAATCGAACCGCTCTACC	721
Ictalurus punctatus	TTATTGGCGTTCCTGGTACGAGTCTCCAACTTTCGAGCAGGACCTGGAAAACCTGTTCAAGCAGCTGCAGCCTCTCTACC	724
Larimichthys crocea	TGACTGGCGCTCTTGGTATGAGACGCAGACCTTTGAGGAAGATCTTGAGGAACTGTACAGGACCATTCAACCTCTGTACC	730
Lates calcarifer	TGACTGGCGGTCTTGGTATGAGACTGAGACCTTTGAGCAGGATTTAGAGAAACTCTACAGGACCATTGAGCCCCTCTACC	733
Latimeria chalumnae	TTTCTGGCGCTCATGGTATGAGTCTTCAGAATTTGAAAATGACCTTGAGAAGATTTATCAGCAACTGGAACCACTGTACC	712
Lepisosteus oculatus	TTACTGGCGCTCCTGGTATGAATCCCCCACCTTTCAAGAAGACCTGGAGACAATTTATCAGCAGCTACAGCCTCTGTACC	718
Macaca fascicularis	CTACTGGCGCTCCTGGTACAACTCCCCCACTTTTGAGGACGATCTGGAGCACCTCTACCAACAGCTAGAGCCCCTCTACC	757
Manacus vitellinus	CTACTGGCGCTCCTGGTACGACTCAGACACCTTTGAGGATGACCTGGAGAGTATCTACAACCAGCTGGAGCCGCTCTACC	709
Mus musculus	CTTCTGGCGCTCCTGGTATGAGTCCCCCTCCTTTGAGGAGAGTCTGGAACATATCTACCACCAACTAGAGCCCCTCTACC	769
Neolamprologus brichardi	TGACTGGCGCGCTTGGTATGAGACTGAAACTTTTAGGCAGGACTTGGAAGAAATCTACAAGACCATTGAGCCGCTCTACC	736
Oncorhynchus mykiss	ATACTGGCGTTCGTGGTACAAGTCAAAGACTTTTGCGAATGACCTGGAGGCTATTTACAAACAGGTGCAGCCGCTCTACC	727
Oreochromis niloticus	TGACTGGCGCTCTTGGTATGAGACTGAAACCTTTAGGCAGGACTTGGAAGAAATCTACAAGACCATTGAGCCCCTCTACC	736
Poecilia formosa	TGATTGGCGCTCTTGGTATGAGACGGAAACCTTTCAGGAGGATTTAGAAAATCTCTACAAGACAATTGAACCGCTCTACC	742
Poecilia latipinna	TGATTGGCGCTCTTGGTATGAGACGGAAACCTTTCAGGAGGATTTAGAAAATCTTTACAAGACAATTGAACCGCTCTACC	742
Pseudopodoces humilis	CTACTGGCGCTCCTGGTATGACTCAGACTCCTTTGAGAAGGACCTGGAGCGCATCTACACCCAGCTGGAGCCACTCTACC	709
Pundamilia nyererei	TGACTGGCGCTCTTGGTATGAGACTAAAACTTTTAGGCAGGACTTGGAAGAAATCTACAAGACCATTGAGCCGCTCTACC	736
Pygocentrus nattereri	TTACTGGCGTTCCTGGTATGAATCTCCCACATTTGAGCAGGACTTGGAGAGACTGTACAAACAACTGCAGCCACTTTACC	709
Python bivittatus	TTATTGGCGGTCCTGGTATGATTCACCAACATTTGAGGAAGATCTAGAGCAACTCTATCAACAGCTGGAACCTCTCTATC	721
Rattus norvegicus	CTACTGGCGCTCCTGGTATGAGTCCCCTCCTTTGAAGAGAGTTTGGAGCATCTCTACCACCAAGTCGAGCCCCTCTACC	772
Scleropages formosus	CTACTGGCGATCCTGGTATGACTCACCTAACTTTGAAGAAGACTTGGAGAGGATTTACAAGCAGTTGGAGCCACTGTACC	718
Sinocyclocheilus anshuiensis	ATACTGGCGTTCCTGGTACGAATCTCCAACCTTTGAACAAGACCTAGAGAATCTGTTTAAACAGCTGGAACCTCTCTACC	709
Xenopus tropicalis	CTACTGGCGCTCTTGGTATGCATCTCCCACATTTGAAGAGGACCTGGATGGCCTATACCAACAACTAGAACCTCTATACC	718
Lipotes vexillifer	CTACTGGCGCTCCTGGTACGACTCCCCCACCTTCACGGAGGATCTGGAGCACCTCTACCATCAGTTAGAGCCCCTGTACC	778
Orcinus orca	CTACTGGCGCTCCTGGTACGACTCCCCCACCTTCACGGAGGATCTGGAGCACCTGTACCATCAGTTAGAGCCCCTGTACC	763
Homo sapiens	CTACTGGCGCTCCTGGTACAACTCCCCCACCTTCGAGGACGATCTGGAACACCTCTACCAACAGCTAGAGCCCCTCTACC	754

	740 750 760 770 780 790 800 810	
conservation		
Callorhinchus milii	TCAACCTCCATGCTTATGTGAGAAGAAAGTTATATCAAAGATATGGAGCCAAATATATCAATCTGAGAGGACCTATTCCT	813
Alligator mississippiensis	TGAACCTGCACGCTTTCGTCAGGAGGAAGCTGTATGACCGCTATGGTGCCAAGTACATCAACCTCAAGGGACCCATCCCT	798
Anolis carolinensis	TAAACCTGCATGCTTTCGTCAGGAGGAAACTATATGACCGATATGGATCCAAATACATCAACTTGAAAGGACCTATTCCA	870
Astyanax mexicanus	AGAACCTGCACGCCTTCGTCCGACGCAAGCTCTACGATTTCTACGGTCCAAAGTATATCAATCTTAAAGGACCCATCCCT	795
Boleophthalmus pectinirostris	TGCATCTTCATGCATTTGTGCGTCGCCAGTTGTACAACCAGTATGGCCCCAAATATATCAACCTCAAAGGGCCCATTCCA	816
Cynoglossus semilaevis	AAAACCTACATGCCTTTGTTCGGCGGCAGCTCTACAACCAATATGGTCCCAAGTACATCAACCTAAAAGGACCAATTCCT	819
Danio rerio	AAAACCTGCACGCTTTTGTCCGCCGAAAGCTCTACGACTACTATGGTCCGAAATACATCAACCTTAAAGGTCCCATCCCT	918
Esox lucius	AGAATCTTCATGCCTTTGTACGTCGTAAGCTCTACAACCACTACGGCCCAAAGTACATAAATCTGAAAGGACCTATCCCT	807
Fundulus heteroclitus	TGAACCTGCATGCCTTTGTGCGACGCAAGCTCTACAACTTCTACGGTCCTAAATATATCAATCTGAAAGGGCCTATCCCT	825
Gekko japonicus	TAAATCTCCATGCTTTTGTCAGGAGGAAACTGTATGAGCGATATGGCCCGAGATACATCAATCTGCAAGGCCCCATTCCC	810
Haplochromis burtoni	TGAATCTGCACGCCTTTGTGCGCCGCCAGCTCTACAACCAGTATGGCCCCAAATACATCAACCTTAAAGGACCTATCCCT	816
Hippocampus comes	AGCACCTGCACGCCTTTGTCCGCCGGCAACTCTACAAGCAGTACGGCCCCAAGTACATCAACCTGCGAGGACCCATCCCC	801
Ictalurus punctatus	TGAACCTGCACGCCTTTGTCCGTCGCAAGCTGTATAACTACTATGGCCCCAAGTACATCAATCTAAAAGGCCCCATCCCT	804
Larimichthys crocea	AGCACCTGCATGCCTTTGTACGCCGCAAGCTCTACAACCAGTACGGTCCCAAGTACATCAACCTTAAAGGACCCATCCCT	810
Lates calcarifer	AGAACCTGCATGCCTTTGTGCGCCGCCAGCTCTACAACCAATATGGTCCTAAGTACATCAATCTTAAAGGACCTATCCCT	813
Latimeria chalumnae	TGAACCTGCATGCATTTGTAAGGAGGAAGCTATATCAGCGCTATGGTGCTAAGTACATTAACCTAAGGGGACCCATTCCT	792
Lepisosteus oculatus	TCCATTTGCACGCCTTTGTTCGCCGCAAACTCTACCAGCGCTATGGCCCCAAGTACATCAACCTCAAAGGACCCATTCCT	798
Macaca fascicularis	TGAACCTCCATGCCTTCGTCCGCCGTGCACTGCATCGCCGATATGGGGACAGATACATCAACCTCAGGGGACCCATCCCT	837
Manacus vitellinus	TCAACCTGCACGCCTTCGTCCGGAGGAAGCTCTATGACTACTACGGCCCCAAATACGTCAACCTGAGGGGTCCCATCCCT	789
Mus musculus	TGAACCTCCATGCCTACGTCCGCCGCGCACTGCACCGCCGCTATGGGGACAAATACGTCAATCTCAGGGGGCCTATTCCT	849
Neolamprologus brichardi	TGAATCTGCACGCCTTTGTGCGCCGCCAGCTCTACAACCAGTATGGCCCCAAATACATCAACCTTAAAGGACCTATCCCT	816
Oncorhynchus mykiss	AGAATCTTCACGCCTTTGTGCGCCGCAAGCTCTACGACCATTACGGACCGAAATACATCAATCTTAAAGGACCTATCCCT	807
Oreochromis niloticus	TGAATCTGCACGCCTTTGTTCGCCGCCAGCTCTACAACCAGTATGGCCCCAAGTACATCAACCTTAAAGGACCTATCCCT	816
Poecilia formosa	TGAACCTGCATGCCTTCGTGCGACGCAAGCTCTACAACTTCTATGGACCCAAATATATCAACCTAAAGGGACCCATCCCT	822
Poecilia latipinna	TGAACCTGCATGCCTTCGTGCGACGCAAGCTCTACAACTTCTATGGACCCAAATATATCAACCTAAAGGGACCCATCCCT	822
Pseudopodoces humilis	TCAACCTGCATGCCTTTGTCCGGAGGAAGCTGTATGATCACTATGGGCCCAAATACATCAACCTGAAGGGTCCCATCCCT	789
Pundamilia nyererei	TGAATCTGCACGCCTTTGTGCGCCGCCAGCTCTACAACCAGTATGGCCCCAAATATATCAACCTTAAAGGACCTATCCCT	816
Pygocentrus nattereri	TGAACCTGCACGCCTTTGTCCGGCGCAAACTCTACGACTTTTACGGTCCCAAGTATATCAACCTTAACGGACCAATACCT	789
Python bivittatus	TAAATCTGCATGCATTTGTCAGGAGGAAACTATATGAGCAATATGGTTCTAAATACATAAACCTGAAAGGCCCTATTCCA	801
Rattus norvegicus	TGAACCTCCATGCCTTTGTCCGTCGCGCACTGCACCGCCGCTATGGGGACAAATACATCAATCTCAGAGGTCCTATTCCC	852
Scleropages formosus	AGAATCTGCATGCATTCGTGCGCCGCAAACTGTATGAACGTTATGGCCCAAAGTACATCAACCTCAACGGACCCATCCCA	798
Sinocyclocheilus anshuiensis	AAAACCTGCATGCCTTTGTCCGCCGAAAGCTCTACAACTACTATGGTCCCAAATACATCAACCTTAAAGGTCCCATCCCC	789
Xenopus tropicalis	TCAATCTACATGCCTTTGTACGCAGGAAACTGTACGAGCGCTATGGGGCTAAATATATCAACTTGAAAGGACCAATTCCA	798
Lipotes vexillifer	TGAACCTCCACGCCTACGTCCGCCGCGCACTGCACCGCCGATACGGGGACAGATTCATCAACCTCAGGGGACCCATCCCT	858
Orcinus orca	TGAACCTCCACGCCTACGTCCGCCGCGCACTGCACCGCCGATACGGGGACAGATTCATCAACCTCAGGGGACCCATCCCT	843
Homo sapiens	TGAACCTCCATGCCTTCGTCCGCCGCGCACTGCATCGCCGATACGGAGACAGATACATCAACCTCAGGGGACCCATCCCT	834

Species	Alignment (820 · 830 · 840 · 850 · 860 · 870 · 880 · 890)	
conservation		
Callorhinchus milii	GCACATCTCCTGGGTAATATGTGGGCACAGCAGTGGAATAATATCTATGAAATGATGATCCCATTCCCAGACAAAGACAA	893
Alligator mississippiensis	GCTCATTTGCTGGGCAACATGTGGGCTCAGCAGTGGAACAACATCTATGACATGATGATCCCCTACCCAAACAAGCCCAA	878
Anolis carolinensis	GCTCATTTACTGGGTAACATGTGGGCCCAGCAATGGAATAATATTTATGATATGATGATCCCTTTCCCAAATAAACCTAA	950
Astyanax mexicanus	GCCCATCTTCTCGGTAATATGTGGTCACAAACCTGGAATAACATCTATAACATGATGATTCCTTTCCCTGACAAGCCTAA	875
Boleophthalmus pectinirostris	GCTCACCTCCTCGGAAACATGTGGTCTCAGACGTGGAACAACATCTATGGGATGATGATACCTTTTCCAAACAAACCTAA	896
Cynoglossus semilaevis	GCTCACCTGTTAGGAAACATGTGGGCCCAGACCTGGAACAACATTTATGGTTTGATGATTCCATTTCCAGAGAAACCCAA	899
Danio rerio	GCTCATCTATTGGGCAACATGTGGTCACAAACATGGAACAACATCTACAATATGATGATCCCTTTCCCTAACAGACCCAA	998
Esox lucius	GCTCACCTTCTAGGAAACATGTGGGCGCAAACCTGGAACAACATCTATGACATGATGATCCCCTTCCCCGGAAAACCTAA	887
Fundulus heteroclitus	GCTCACCTGTTGGGAAATATGTGGGCCCAGTCGTGGGATAACATTTATAGTATGATGATCCCATTTCCTGACAAACCCAA	905
Gekko japonicus	GCTCACTTACTGGGTAACATGTGGGCCCAGCAGTGGAACAACATTTACGATATGATGATTCCTTTCCCAGACAAGCCCAA	890
Haplochromis burtoni	GCTCACTTGCTAGGAAATATGTGGGCCCAGACCTGGAACAACATTTATGATATGATGATCCCATTTCCTGACAAACCTAA	896
Hippocampus comes	GCTCATCTGCTGGGAAACATGTGGGCCCAGACCTGGAACAACATCTACGGCTTGATGATACCGTTTCCTGACAAACCCAA	881
Ictalurus punctatus	GCCCATCTGCTTGGAAACATGTGGGCACAAAGCTGGAACAACATCTATGACTTGATGATCCCTTTCCCTAACAAGCCCAA	884
Larimichthys crocea	GCTCACCTGCTTGGAAATATGTGGGCCCAGACCTGGAACAATATTTATGGTCTAATGATCCCATTTCCTGAGAAACCCAA	890
Lates calcarifer	GCCCACCTGCTAGGAAATATGTGGGCCCAGACCTGGAATAATATTTATGGTATGATGATCCCATTTCCTGACAAACCTAA	893
Latimeria chalumnae	GCCCACTTATTAGGTAACATGTGGGCTCAGCAATGGAATAACATCTATGACATGATGATTCCATTCCCACAAAAAACAAA	872
Lepisosteus oculatus	GCACACTTACTTGGAAACATGTGGGCTCAACAGTGGAACAACATCTATGATATGATGGTACCCTTTCCTCACAAGCCCAA	878
Macaca fascicularis	GCTCATCTGCTGGGAGACATGTGGGCCCAGAGCTGGGAAAACATCTACGACATGGTGGTGCCTTTCCCAGACAAGCCCAA	917
Manacus vitellinus	GCTCACCTCCTGGGGAACATGTGGGCTCAGCAGTGGAACAACATCTACGACCTGATGGTCCCCTACCCTGAGAAGCCCAA	869
Mus musculus	GCCCATTTGCTGGGAGACATGTGGGCCCAGAGCTGGGAGAACATCTACGACATGGTAGTGCCTTTCCCAGACAAACCCAA	929
Neolamprologus brichardi	GCTCACTTGCTAGGAAATATGTGGGCCCAGACCTGGAACAACATTTATGATATGATGATCCCATTTCCTGACAAACCTAA	896
Oncorhynchus mykiss	GCTCACCTGCTGGGAAACATGTGGTCGCAAACCTGGAACAACATCTATGGCATGATGATCCCCTTCCCCGGCAAACCCAA	887
Oreochromis niloticus	GCTCACTTGCTAGGAAATATGTGGGCCCAGACCTGGAACAACATTTATGATATGATGATCCCATTTCCTGACAAACCTAA	896
Poecilia formosa	GCACACCTGTTGGGAAATATGTGGGCCCAGACATGGAATAACATTTATGATATGATGATCCCATTTCCTGACAAACCCAA	902
Poecilia latipinna	GCACACCTGTTGGGAAATATGTGGGCCCAGACATGGAATAACATTTATGATATGATGATCCCATTTCCTGACAAACCCAA	902
Pseudopodoces humilis	GCTCACCTCCTGGGGAATATGTGGGCTCAGCAGTGGAACAACATCTATGACCTGATGATCCCCTACCCTGAGAAGCCTAA	869
Pundamilia nyererei	GCTCACTTGCTAGGAAATATGTGGGCCCAGACCTGGAACAACATTTATGATATGATGATCCCATTTCCTGACAAACCTAA	896
Pygocentrus nattereri	GCCCATCTTCTTGGAAACATGTGGTCACAAACCTGGAATAACATCTACGACATGATGATTCCGTTCCCTGATAAGCCAAA	869
Python bivittatus	GCTCACTTATTGGGTAACATGTGGGCCCAACAATGGAACAACATTTACGACATGATGGTGCCTTTTGCAAATAAGCCAAA	881
Rattus norvegicus	GCTCATCTGCTGGGAGACATGTGGGCGCAGAGCTGGGAGAACATTTACGACATGGTAGTGCCTTTCCCGGACAAACCCAA	932
Scleropages formosus	GCACATCTCTTAGGAAACATGTGGGCACAAACTTGGAACAACATCTATGACATGATGATCCCCTTCCCAGGGAAGCCCAA	878
Sinocyclocheilus anshuiensis	GCCCACCTCTTGGGCAACATGTGGTCACAAACGTGGAACAACATCTACAACATGATGATTCCTTTTCCTGACAAGCCCAA	869
Xenopus tropicalis	GCTCATCTGTTTGGTAACATGTGGTCCCAGCAGTGGAATAATATCTACGACATGATGATTCCCTTCCCAGACAAGACCAA	878
Lipotes vexillifer	GCTCACCTGCTGGGGGACATGTGGGCCCAGAGCTGGGACAAAATCTACGACATGGTGGTGCCTTTTCTCTGCCAAGCCCAA	938
Orcinus orca	GCTCACCTGCTGGGGGACATGTGGGCCCAGAGCTGGGACAAAATCTACGACATGGTGGTGCCTTTTCTCTGACAAGCCCAA	923
Homo sapiens	GCTCATCTGCTGGGAGACATGTGGGCCCAGAGCTGGGAAAACATCTACGACATGGTGGTGCCTTTCCCAGACAAGCCCAA	914

Species	Alignment (900 · 910 · 920 · 930 · 940 · 950 · 960 · 970)	
conservation		
Callorhinchus milii	CATTGATGTTACTGATGCAATGGTTCAGCAAAATTGGAACGCCACCCGTATGTTCAGAGTTGCTGAGGATTTCTTCAAAT	973
Alligator mississippiensis	TCTGGATGTCACAAATGCCATGAATCAGAAAAACTGGAACGCCACGCACATGTTCCGGGTCTCGGAGGAGTTCTTCACTT	958
Anolis carolinensis	TGTGGACGTCACCAACACCATGCAGCAACAAAATTGGAACGTAACACGCATGTTCCGGGTGTCGGAGGAATTCTTCACCT	1030
Astyanax mexicanus	TGTGGATGTGACTAATGCCATGGTTGAGAAGAACTACAACGCCACACATATGTTCCGGGTAGCTGAAGAGTTCTTCACAT	955
Boleophthalmus pectinirostris	CCTAGATGTGACAGCTGAAATGGAGAGACAAGGCTACAATGCCACACACATGTTCAGAGTAGCAGAGGAGTTCTTCACCT	976
Cynoglossus semilaevis	CCTGGATGTGACTGAGGAGATGGTCAACCAGGGTTACAATGCTACACACATGTTCCGTGTGGCTGAGGAGTTCTTCACCT	979
Danio rerio	TGTAGATGTGACCAATACCATGATCGCTAAAGGTTATAATGCAACGCACATGTTCCGGGTAGCAGAGGAGTTTTTCACCT	1078
Esox lucius	TGTGGATGTGACCAAAGAAATGGAGGCCAATAAATGGAACGCCACTCACATGTTCCGTGTGGCTGAGGAGTTCTTCACAT	967
Fundulus heteroclitus	CCTGGATGTGACCGATGAGATGGTCAGACAAGGTTACAATGCCACACACATGTTTCGTTTATCTGAGGAGTTCTTCACTT	985
Gekko japonicus	TCTCGATGTCACCAATACAATGAAACAAAAAAATTGGAATGCAACACACATGTTCCGGGTGGCAGAGGAGTTCTTCACGT	970
Haplochromis burtoni	CATTGATGTGACTGATGAAATGGTCAGACAAGGCTATAACGCCACACACATGTTTCGTGTGGCCGAGGAGTTCTTCACCT	976
Hippocampus comes	CATGGACGTGACTGAAGAAATGCTCAACCAAGGCTACAACGCGACGGCCATGTTTCGCGTTGCGGAGGAGTTCTTCACAT	961
Ictalurus punctatus	TGTGGACGTGACTAACACCATGGTTGCCCAAGGCTACAATGCCACCCATATGTTTCGGGTGGCTGAAGAGTTCTTCACCT	964
Larimichthys crocea	CTTGGATGTGACTAATGAAATGATCAACCAACGCTATAATGCCTCGCACATGTTCCGTGTGTCTGAGGAGTTCTTCACTT	970
Lates calcarifer	CATGGATGTGACTGATGAAATGGTCAGACAAGGCTACAATGCCACACACATGTTTCGTGTGGCCGAGGAGTTCTTCACCT	973
Latimeria chalumnae	CGTAGATGTCACGGATGCTATGATTAAGAAGAACTGGAATGCTACCCATATGTTCAAAGTTTCTGAAGAGTTCTTCACCT	952
Lepisosteus oculatus	CCTTGACGTGACACAAACTATGGTGAATCTGAAGTGGAATGCGACCCACATGTTCCGAGTCTCCGAAGAGTTCTTCACTT	958
Macaca fascicularis	CCTCGACGTCACCAGTACTATGCTGCAGCAGGGCTGGAATGCCACGCACATGTTCCGGGTGGCAGAGGAGTTCTTCACCT	997
Manacus vitellinus	CCTTGATGTCACGAGTGCCATGGTGCAGCAGGGCTGGAATGCCACCCACATGTTCCGGGTCTCAGAGGAGTTCTTCACCT	949
Mus musculus	CCTCGATGTCACCAGTACAATGGTACAGAAGGGCTGGAACGCCACACACATGTTCCGGGTATCAGAGGAATTCTTCACCT	1009
Neolamprologus brichardi	CATTGATGTGACTGATGAAATGGGCAGACAAGGCTATAACGCCACACACATGTTTCGTGTGGCCGAGGAGTTCTTCACCT	976
Oncorhynchus mykiss	TGTGGACGTGACCGATGAAATGGTGGCCAAAAACTGGAATGTTACTCACATGTTCCGGGTGGCTGAGGAGTTCTTCACCT	967
Oreochromis niloticus	CATTGATGTGACTGATGAAATGGTCAGACAAGGCTATAACGCCACACACATGTTTCGTGTGGCCGAGGAGTTCTTCACCT	976
Poecilia formosa	CCTGGATGTGACCAGTGAGATGGTCAATCAGGGCTACAATGCTACACACATGTTCCGTGTAGCTGAGGAGTTCTTCACCT	982
Poecilia latipinna	CCTGGATGTGACAAGCGAGATGGTCAATCAGGGCTACAATGCTACACACATGTTCCGTGTGGCTGAGGAGTTCTTCACCT	982
Pseudopodoces humilis	CCTTGATGTCACGAGCAGCATGGTGCAGCAGGGTTGGAATGCCACCCACATGTTCCGAGTCTCGGAGGAGTTCTTCACCT	949
Pundamilia nyererei	CATTGATGTGACTGATGAAATGGTCAGACAAGGCTATAACGCCACACACATGTTTCGTGTGGCCGAGGAGTTCTTCACCT	976
Pygocentrus nattereri	TGTGGATGTGACTAGCAACATGGTTGCACAAGGCTACAATGCCACCACCATGTTCCGTGTGGCTGAGGAGTTCTTCACTT	949
Python bivittatus	TCTAGATGTTACCAACGTCATGAAGCAGAAAAACTGGAACGTAACAGTCATGTTCCGGGTGGCAGAGGAGTTCTTCACCT	961
Rattus norvegicus	CCTCGATGTCACCAGTACAATGGTACAGAAGGGCTGGAATGCCACGCACATGTTCCGGGTCGCAGAGGAATTCTTTACCT	1012
Scleropages formosus	TGTTGATGTCACGGATGCTATGATTGCACAAGACTGGACCACCACAAAAATGTTCAGAGTCTCAGATGAGTTCTTCACTT	958
Sinocyclocheilus anshuiensis	TGTAGATGTGACGGACACCATGGTTGCTAAAGGTTATAATGCCACTCACATGTTCCGGGTTGCCGAGGAGTTCTTCACCT	949
Xenopus tropicalis	TATAGATGTCACTAATACAATGAGGGAAAAGGGATGGAATGCTACCCATATGTTCAGAGTTTCAGAGGAGTTCTTCACCT	958
Lipotes vexillifer	TCTTGACGTTACCAGTACTATGGTGCAGAAGGGATGGAACGCTACACACATGTTCCGGGTGGCAGAGGAGTTCTTCACCT	1018
Orcinus orca	TCTTGACGTTACCAGTACTATGGTGCAGAAGGGATGGAACGCTACACACATGTTCCGGGTGGCAGAGGAGTTCTTCACCT	1003
Homo sapiens	CCTCGATGTCACCAGTACTATGCTGCAGCAGGGCTGGAACGCCACGCACATGTTCCGGGTGGCAGAGGAGTTCTTCACCT	994

```
                                     980       990       1000      1010      1020      1030      1040      1050
conservation
Callorhinchus milii                CATTGAATCTTACTGAGATGCCCAAGGAGTTCTGGGATGAATCAATGCTGGAAAAGCCAAATGATGGCCGCGAGGTGGTG   1053
Alligator mississippiensis         CCCTGGGGCTTCTGGGGATGCCCCCGGAGTTCTGGGCTGAGTCCATGCTGGAGAAGCCTACGGATGGGCGGGAGGTGGTG   1038
Anolis carolinensis                CGCTTGGCATGCTTGGAATGCCACCTGAATTCTGGGAGAAATCCATGCTGGAGAAGCCAACAGATGGGCGGGAGGTGGTG   1110
Astyanax mexicanus                 CCCTGGGGCTCAAGGAGATGCCACAGAAGTTCTGGGACAAATCAATGCTGGAGAAGCCTGATGATGGACGGGAGGTTGTG   1035
Boleophthalmus pectinirostris      CTCTGGGCCTGGAACCAATGCCACAGGAGTTCTGGGATGAGTCCATGTTGGTGAAGCCTGAAGGT...CGTGAGGTGGTG   1053
Cynoglossus semilaevis             CTCTGGGATTGGACCCAATGCCTCAGGAATTCTGGGATGAGTCCATGTTGGAGAAGCCTGAAGGT...CGAGAGGTGGTG   1056
Danio rerio                        CTTTGGGTTTGCTGGAAATGCCTCCTGAGTTTTGGGACAAGTCCATGCTGGAGAAACCCACTGATGGAAGAGAGGTGGTG   1158
Esox lucius                        CCCTGGGTCTCATCAAAATGCCTGATGAGTTCTGGAATAAGTCTATGTTGGAAAAACCAGACGAC...CGGGAGGTGGTG   1044
Fundulus heteroclitus              CTCTGGGATTAAAGGAGATGCCCATGGAATTCTGGGAAGAGTCCATGCTCGTTAAGCCTGATGAT...CGGGAGGTTGTG   1062
Gekko japonicus                    CACTTGGCATGCTGGAAATGCCACCTGAATTCTGGGAGAGCTCAATGTTGGAGAAGCCAACGGATGGGCGGGAAGTCGTC   1050
Haplochromis burtoni               CTCTGGGTTTGGAGAGGATGCCTGAGGAGTTCTGGGAGGGATCCATGCTGGTTAAGCCTCAGGGT...CGAGAGGTGGTG   1053
Hippocampus comes                  CTCTGGGTTTAGCCGAGATGCCTCCAGAATTCTGGCAGGAATCCATGCTGGAGAAGCCCGACGAT...CGAGAGGTGGTG   1038
Ictalurus punctatus                CTCTTGGTCTGAAGGAGATGCCTCCCAAGTTCTGGGAGAAGTCCATGCTGGAGAAGCCCACTGGTGACCAAGAGGTGGTC   1044
Larimichthys crocea                CTCTGGGATTAGAAAAGATGCCTGATGAATTCTGGGAGGGATCAATGCTGGTGAAGCCTGAAGGT...CGAGAGGTGGTG   1047
Lates calcarifer                   CTCTGGATTTAGAGCCGATGCCTCAGGAGTTCTGGGATGAGTCCATGCTGGTGAAGCCTGAAGGT...CGGGAGGTGGTG   1050
Latimeria chalumnae                CTCTTGGACTGCTGGGAATGCCACAGGAATTCTGGGATCACTCCATGCTAGAAAAGCCAAAAGATGGGAGAGAGGTGGTG   1032
Lepisosteus oculatus               CCCTAGGCCTGATTGCCATGCCACAGGAGTTCTGGGACCATTCCATGCTGGAGAAACCCACTGATGGCCGGCAGGTTGTG   1038
Macaca fascicularis                CCCTGGAGCTCTCCCCCATGCCTCCCGAGTTCTGGGAAGGGTCGATGCTGGAGAAGCCGGCCGACGGGCGGGAGGTGGTG   1077
Manacus vitellinus                 CCTTGGGGCTGCTGGAGATGCCCCCCGAGTTCTGGGAGAAGTCCATGCTGGAGAAGCCGACGGACGGGCGGGAGGTGGTG   1029
Mus musculus                       CGCTGGGGCTCTCACCCATGCCTCCTGAGTTCTGGGCGGAGTCAATGCTGGAGAAACCAACGGATGGACGGGAGGTGGTG   1089
Neolamprologus brichardi           CTCTGGGTTTGGAGAGGATGCCTGAGGAGTTCTGGGAGGGATCCATGCTGGTTAAGCCTCAGGGT...CGAGAGGTGGTG   1053
Oncorhynchus mykiss                CCCTCGGCCTCATCAAAATGCCCCAAGAGTTCTGGGACAAGTCTATGTTTGAGAAACCAGAAGGT...CGCGAGGTGGTG   1044
Oreochromis niloticus              CTCTGGGTTTGGAGAGGATGCCTGAGGAGTTCTGGGAGGGATCCATGCTGGTTAAGCCTCAGGGT...CGAGAGGTGGTG   1053
Poecilia formosa                   CTCTGGGTCTAAAGGAGATGCCTCCAGAGTTCTGGGAAGGGTCGATGCTTGTTAAGCCTGAAGGT...CGGGAGGTTGTG   1059
Poecilia latipinna                 CTCTGGGTCTAAAGGAGATGCCTCCAGAGTTCTGGGAAGGGTCGATGCTTGTTAAGCCTGAAGGT...CGGGAGGTTGTG   1059
Pseudopodoces humilis              CCCTGGGGCTTCTGGAGATGCCCACTGAATTCTGGAAGAAGTCCATGCTGGAAAAGCCGACAGATGGGCGGGAGGTGGTG   1029
Pundamilia nyererei                CTCTGGGTTTGGAGAGGATGCCTGAGGAGTTCTGGGAGGAATCCATGCTGGTTAAGCCTCAGGGT...CGAGAGGTGGTG   1053
Pygocentrus nattereri              CTCTGGGACTGAAGGAGATGCCCCAGAAGTTCTGGGACAAGTCCATGCTGGAGAAGCCTGATGAC...CGAGACGTGGTG   1026
Python bivittatus                  CGCTTGGCATGCTTGGAATGCCACCTGAATTCTGGGAGAAATCCATGCTGGAGAAACCAACAGATGGACGGGAGGTGGTG   1041
Rattus norvegicus                  CGCTGGGGCTCTCCCCCATGCCTCCAGAGTTCTGGGCGGAGTCGATGCTGGAGAAACCAGCTGATGGACGGGAGGTGGTG   1092
Scleropages formosus               CACTGGGTCTGGAAAAGATGCCAGATGAATTTTGGAACATGTCCATGCTAGAGAAGCCAAATGATGGACGTGAGGTGGTG   1038
Sinocyclocheilus anshuiensis       CTCTGGGTTTGCTCGAAATGCCTCCTGAGTTTTGGGACAAGGCCATGCTGGAGAAACCCACTGATGGAAGGGAGGTGCTC   1029
Xenopus tropicalis                 CCCTGGGTCTGCTGGAGATGCCCCCAGAGTTTTGGGAAAAGTCAATGTTGGAAAAACCAGCAGATGGACGTGAGGTGGTG   1038
Lipotes vexillifer                 CCCTAGGGCTCTTGCCCATGCCTCCTGAGTTCTGGGCAGAGTCCATGCTGGAGAAGCCAAGCGACGGGCGTGAGGTGGTA   1098
Orcinus orca                       CCCTAGGGCTCTTGCCCATGCCTCCTGAGTTCTGGGCAGAGTCCATGCTGGAGAAACCAAGCGACGGGCGTGAGGTGGTG   1083
Homo sapiens                       CCCTGGAGCTCTCCCCCATGCCTCCCGAGTTCTGGGAAGGGTCGATGCTGGAGAAGCCGGCCGACGGGCGGGAAGTGGTG   1074

                                         1060      1070      1080      1090      1100      1110      1120      1130
conservation
Callorhinchus milii                TGCCACGCTTCAGCCTGGGATTTTCTGAACCGCAAGGCTTTCAGAATCAAACAATGTACAACAGTCACAATGGAGCAACT   1133
Alligator mississippiensis         TGCCATGCCTCAGCCTGGGACTTCTACAACCGCAAGGATTTCAGGATAAAGCAGTGCACCACAGTCACCATGGAGCAGCT   1118
Anolis carolinensis                TGCCATGCATCGGCATGGGACTTCTACAATCGCAAAGACTTCAGGATAAAGCAGTGCACAACCATCACTATGGAGCAGCT   1190
Astyanax mexicanus                 TGTCACGCCTCTGCCTGGGACTTCTACAACCGTGAGGACTTTAGGATCAAGCAGTGCACTATAGTGACTATGGAGCAGCT   1115
Boleophthalmus pectinirostris      TGCCACGCTTCAGCCTGGGACTTCTACAACCGGAAAGACTTCAGGATCAAGCAGTGCACCACAGTGACCATGGAGCAGTT   1133
Cynoglossus semilaevis             TGTCACGCGTCTGCCTGGGACTTTTACAACCGCAAAGACTTCAGAATCAAGCAGTGCACCACAGTGACCATGGAGCAGCT   1136
Danio rerio                        TGTCATGCTTCTGCCTGGGACTTTTACAACCGCAAAGACTTCAGAATCAAGCAGTGCACGACTGTGACCATGGAACAGCT   1238
Esox lucius                        TGCCATGCATCTGCCTGGGACTTCTACAACAGAAAGGACTTCAGGATCAAACAGTGTACCACCGTCAACATGCAACAGCT   1124
Fundulus heteroclitus              TGCCATGCATCTGCCTGGGACTTTTACAACCGCAAAGACTTCAGGATTAAGCAGTGCACCACAGTGACGATGGAGCAGCT   1142
Gekko japonicus                    TGCCATGCATCAGCGTGGGATTTCTACAACCGCAAAGACTTCAGGATAAAGCAGTGCACAACAGTGACCATGGAGCAGCT   1130
Haplochromis burtoni               TGCCACGCGTCTGCCTGGGACTTTTACAATCGCAAGGACTTCAGAATTAAGCAGTGCACCACAGTCACGATGGAACAGCT   1133
Hippocampus comes                  TGTCACGCGTCCGCCTGGGACTTTTACAACCGCAAAGACTTCAGAATCAAGCAGTGCACCACAGTCACGATGGAACAGCT   1118
Ictalurus punctatus                TGCCATGCCTCTGCGTGGGACTTTTATAACCGGGAGGACTTTAGGATTAAGCAGTGTACCACAGTAACTATGGAGCAGCT   1124
Larimichthys crocea                TGCCATGCATCCGCCTGGGACTTTTACAACCGCAAAGACTTCAGGATCAAGCAGTGCACTACAGTAACAATGGAGCAGCT   1127
Lates calcarifer                   TGCCATGCATCTGCCTGGGACTTTTACAACCGCAAAGACTTCAGGATCAAGCAGTGCACCACAGTGACGATGGAGCAGCT   1130
Latimeria chalumnae                TGCCATGCTTCTGCTTGGGATTTCTATAACAGAAAAGACTTCAGAATAAAGCAATGTACCACTGTGACAATGGAGCAACT   1112
Lepisosteus oculatus               TGTCATGCCTCTGCTTGGGACTTCTACAACCGGAAAGACTTCAGGATTAAGCAGTGCACCACAGTGACCATGGAGCAGCT   1118
Macaca fascicularis                TGCCACGCCTCGGCTTGGGACTTCTACAACAGGAAAGACTTCAGGATCAAGCAATGCACACGGGTCACGATGGACCAGCT   1157
Manacus vitellinus                 TGTCACGCCTCGGCCTGGGACTTCTACAACCGCAAGGACTTCAGGATCAAGCAGTGCACGTCGGTGACCATGGAGCAGCT   1109
Mus musculus                       TGCCACGCCTCTGCCTGGGACTTCTACAACCGGAAGGACTTCCGGATTAAGCAATGCACACGGGTCACGATGGAACAGCT   1169
Neolamprologus brichardi           TGCCACGCGTCTGCCTGGGACTTTTACAATCGCAAGGACTTCAGAATTAAGCAGTGCACCACAGTCACGATGGAACAGCT   1133
Oncorhynchus mykiss                TGTCATGCATCTGCCTGGGACTTCTACAATAGAAAGGACTTCAGGATCAAACAGTGCACCACAGTCAACATGCAGCAGCT   1124
Oreochromis niloticus              TGCCACGCGTCTGCCTGGGACTTTTACAATCGCAAGGACTTCAGAATTAAGCAGTGCACCACAGTCACGATGGAACAGCT   1133
Poecilia formosa                   TGCCATGCATCTGCCTGGGACTTTTACAACCGCAGAGACTTCAGGATTAAGCAGTGCACCACAGTGACCATGGAACAGCT   1139
Poecilia latipinna                 TGCCATGCATCTGCCTGGGACTTTTACAACCGCAGAGACTTCAGGATTAAGCAGTGCACCACAGTAACCATGGAACAGCT   1139
Pseudopodoces humilis              TGTCATGCCTCAGCCTGGGACTTCTACAACCGCAAGGACTTCAGGATCAAGCAATGCACGTCAGTGACCATGGAGCAGCT   1109
Pundamilia nyererei                TGCCACGCGTCTGCCTGGGACTTTTACAATCGCAAGGACTTCAGAATTAAGCAGTGCACCACAGTCACGATGGAACAGCT   1133
Pygocentrus nattereri              TGCCATGCCTCTGCCTGGGACTTCTATAACCGCGAGGACTTCAGGATCAAGCAGTGCACCATAGTGACTATGGAACAGCT   1106
Python bivittatus                  TGCCATGCATCGGCATGGGACTTCTTCAATCGCAAAGACTTCAGGATAAAGCAGTGTACAACAGTCACCATGGAGCAACT   1121
Rattus norvegicus                  TGCCATGCCTCTGCGTGGGACTTCTACAACAGGAAGGACTTCAGGATTAAGCAGTGCACGCGGGTCACGATGGACCAGCT   1172
Scleropages formosus               TGCCATGCCTCTGCCTGGGATTTCTACAACCGCAAGGACTTTAGAATCAAACAGTGCACCACAGTGACCATGGAACAGCT   1118
Sinocyclocheilus anshuiensis       TGCCATGCCTCTGCCTGGGACTTCTACAACCGCAAAGACTTCAGAATCAAACAGTGCACCACTGTGACTATGGAGCAGCT   1109
Xenopus tropicalis                 TGTCATGCATCTGCATGGGACTTCTACAACCGTAAGGACTTCCGGATAAAGCAATGCACTACAGTCACTATGGAGCAGCT   1118
Lipotes vexillifer                 TGCCACGCCTCCGCCTGGGATTTCTACAACAGGAAAGACTTCAGGATCAAGCAGTGCACACAGGTCACTATGGACCAGCT   1178
Orcinus orca                       TGCCACGCCTCCGCCTGGGATTTCTACAACAGGAAAGACTTCAGGATCAAGCAGTGCACGCAGGTCACTATGGACCAGCT   1163
Homo sapiens                       TGCCACGCCTCGGCTTGGGACTTCTACAACAGGAAAGACTTCAGGATCAAGCAGTGCACACGGGTCACGATGGACCAGCT   1154
```

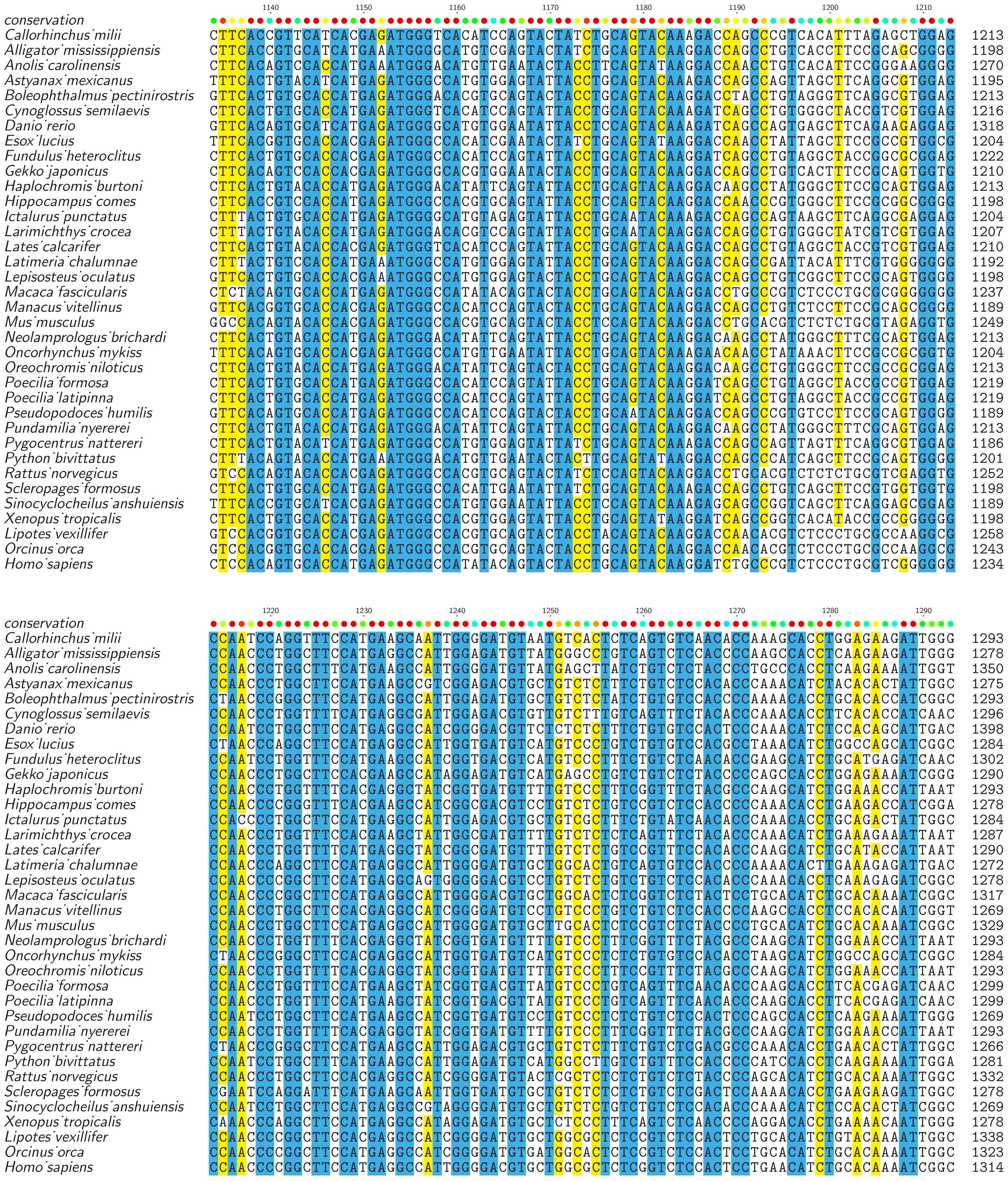
1140
1150
1160
1170
1180
1190
1200
1210
conservation
Callorhinchus milii 1213
Alligator mississippiensis 1198
Anolis carolinensis 1270
Astyanax mexicanus 1195
Boleophthalmus pectinirostris 1213
Cynoglossus semilaevis 1216
Danio rerio 1318
Esox lucius 1204
Fundulus heteroclitus 1222
Gekko japonicus 1210
Haplochromis burtoni 1213
Hippocampus comes 1198
Ictalurus punctatus 1204
Larimichthys crocea 1207
Lates calcarifer 1210
Latimeria chalumnae 1192
Lepisosteus oculatus 1198
Macaca fascicularis 1237
Manacus vitellinus 1189
Mus musculus 1249
Neolamprologus brichardi 1213
Oncorhynchus mykiss 1204
Oreochromis niloticus 1213
Poecilia formosa 1219
Poecilia latipinna 1219
Pseudopodoces humilis 1189
Pundamilia nyererei 1213
Pygocentrus nattereri 1186
Python bivittatus 1201
Rattus norvegicus 1252
Scleropages formosus 1198
Sinocyclocheilus anshuiensis 1189
Xenopus tropicalis 1198
Lipotes vexillifer 1258
Orcinus orca 1243
Homo sapiens 1234
1220
1230
1240
1250
1260
1270
1280
1290
conservation
Callorhinchus milii 1293
Alligator mississippiensis 1278
Anolis carolinensis 1350
Astyanax mexicanus 1275
Boleophthalmus pectinirostris 1293
Cynoglossus semilaevis 1296
Danio rerio 1398
Esox lucius 1284
Fundulus heteroclitus 1302
Gekko japonicus 1290
Haplochromis burtoni 1293
Hippocampus comes 1278
Ictalurus punctatus 1284
Larimichthys crocea 1287
Lates calcarifer 1290
Latimeria chalumnae 1272
Lepisosteus oculatus 1278
Macaca fascicularis 1317
Manacus vitellinus 1269
Mus musculus 1329
Neolamprologus brichardi 1293
Oncorhynchus mykiss 1284
Oreochromis niloticus 1293
Poecilia formosa 1299
Poecilia latipinna 1299
Pseudopodoces humilis 1269
Pundamilia nyererei 1293
Pygocentrus nattereri 1266
Python bivittatus 1281
Rattus norvegicus 1332
Scleropages formosus 1278
Sinocyclocheilus anshuiensis 1269
Xenopus tropicalis 1278
Lipotes vexillifer 1338
Orcinus orca 1323
Homo sapiens 1314

```
                                      1300           1310           1320           1330           1340           1350           1360           1370
conservation
Callorhinchus milii               CTGCTGCAAACAGTCTCAGACGGACACGAGGCTGAAATCAATTATCTTCTGAAGATGGCTCTAGAAAAAATTGCCTTCCT 1373
Alligator mississippiensis        CTCCTTGACCAGGTCGTCCAGGATACAGAGAATGACATCAATTATCTGCTAAAAATGGCCCTGGAGAAGATTGCCTTCCT 1358
Anolis carolinensis               TTGCTCAGTGAAGTCACTGAAGATAAAGAGAGTGATATCAATTACCTGTTGAAAATGGCCCTGCAAAAGATTGCATTTAT 1430
Astyanax mexicanus                CTTCTGGACACTCTGCCTAATGATAATGAGACAGACATCAATTACCTATTGAAAATGGCTTTGGAGAAGATAGCTTTCCT 1355
Boleophthalmus pectinirostris     CTGCTGGGCTCCAGTGCATCTGACACAGAAACCGACATTAACTATTTGTTGAAGATGGCTTTGGAGAAAATAGCTTTCCT 1373
Cynoglossus semilaevis            CTGCTAGATGATCCCTCTTCCAATAATGAAACTGACCTGAACTACCTGTTAAAGATGGCTTTGGAGAAGATCGCCTTCCT 1376
Danio rerio                       CTCCTGGACCAACTAACCGATGACGCTGAGAGTGACATCAATTACCTGTTGAAGATGGCCTTGGAAAAGATCGCCTTCCT 1478
Esox lucius                       CTTTTGCCCAATGCGACCAATGATCCTGAGAGCGACATCAACTACCTACTGAAGATGGCTCTGGAGAAGATTGCCTTCCT 1364
Fundulus heteroclitus             CTGCTGGAATCTGTGACCTCTGACGTTGAAACTGACCTGAACTTCCTGCTGAAGACTGCTCTGGATAAGATAGCCTTCCT 1382
Gekko japonicus                   CTGCTCGACCAGGTCACTGAGGATGCAGACAGTGATATCAACTATCTCCTGAAAATGGCCCTGGAGAAGATTGCCTTCCT 1370
Haplochromis burtoni              CTTCTGGAATCTGTGACATCTGACATCGAAACTGACACCAACTACCTGCTAAAAATGGCTCTGGAGAAGATAGCCTTCCT 1373
Hippocampus comes                 CTGCTGAAGTCGGCGACGTCCGACCCTGAATCCGACATCAACTACCTGTTAAAGATGGCACTGGAGAAGATAGCCTTCCT 1358
Ictalurus punctatus               CTGTTGGACACACTAACTGATGACACTGAAACTGACACAAATTACCTGTTAAAGATGGCTTTAGAGAAGATAGCCTTCCT 1364
Larimichthys crocea               CTGCTGGAATCTGCGACATCTGATATTCAAACTGATACCAACTACCTGTTAAAGATGGCTTTGGAGAAGATAGCCTTCCT 1367
Lates calcarifer                  CTACTGGAATCTGTGACCTCTGATGCTGAAACCGATATCAACTACCTGTTGAAGATGGCTCTGGAGAAGATTGCCTTCTT 1370
Latimeria chalumnae               TTACTGTCACAGTTCACTAATGATACGGAAAGTGACATCAATTATCTTCTGAAGATGGCTCTTGAGAAAATAGCCTTCCT 1352
Lepisosteus oculatus              CTACTGAAATCCTTTGTCCCGGACAATGAAAGTGACATTAACTACCTGCTGAAAATGGCCCTGGAGAAGATTGCCTTCCT 1358
Macaca fascicularis               CTGCTGGACCGTGTCACCAATGACACGGAAAGTGACATCAATTACTTGCTAAAGATGGCACTGGAAAAAATTGCCTTCCT 1397
Manacus vitellinus                CTCCTCAGCAATGCCACTGAGGATGAAGAGAGCAACATCAACTACCTGCTGAAGATGGCCCTGGAGAAGATTGCCTTCCT 1349
Mus musculus                      CTACTGGACCATGTTACCAATGACATAGAGAGTGACATCAATTACCTGCTAAAGATGGCCCTAGAGAAAATCGCCTTCTT 1409
Neolamprologus brichardi          CTTCTGGAATCTGTGACATCTGACATCGAAACTGACACCAACTACCTGTTAAAAATGGCTCTGGAGAAGATAGCCTTCCT 1373
Oncorhynchus mykiss               CTATTGTCTAACGCGACCAATGACAACGAGAGTGACATCAACTACCTGCTGAAGATGGCTCTGGACAAGATGGCCTTCCT 1364
Oreochromis niloticus             CTTCTGGAATCTGTGACATCTGACATCGAAACTGACACCAACTACCTGCTAAAAATGGCTCTGGAGAAGATAGCCTTCCT 1373
Poecilia formosa                  CTTCTAGAGTCTGTGGCCACTGACACAGAAACTGATCTGAACTTCCTGTTGAAGACTGCTCTGGAGAAGATAGCCTTCCT 1379
Poecilia latipinna                CTTCTAGAGTCTGTGGCCACCGACACAGAAACTGATCTGAACTTCCTGTTGAAGACCGCTCTGGAGAAGATAGCCTTCCT 1379
Pseudopodoces humilis             CTCCTCAGCAGTGCTGCTGAGGATGAAGAGAGCAATATCAACTACCTGCTGAAGATGGCCCTGGAGAAGATTGCCTTCCT 1349
Pundamilia nyererei               CTTCTGGAATCTGTGACATCTGACATCGAAACTGACACCAACTACCTGCTAAAAATGGCTCTGGAGAAGATAGCCTTCCT 1373
Pygocentrus nattereri             CTTCTGGAGACGCTGACTGATGATAATGAGACCGACATCAATTACTTATTAAAGATGGCTTTGGAGAAGATAGCGTTCCT 1346
Python bivittatus                 CTGCTCACCCATGCCAGTGAAGATAAAGAGAGTGACATCAATTATCTTCTGAAAATGGCCCTGGAAAAGATTGCCTTCCT 1361
Rattus norvegicus                 CTGCTAGACCGTGTTGCCAATGACATAGAAAGTGACATCAATTACTTGCTAAAGATGGCCCTAGAGAAAATTGCCTTCTT 1412
Scleropages formosus              CTGCTGGAGAACCTGGTTGAAGATCATGAGAGTGATCTTAACTACCTATTGAAGATGGCTCTGGAGAAGATAGCCTTCCT 1358
Sinocyclocheilus anshuiensis      ATCCTGGAACAACGGACCGATGATGCTGAGAGTGACATCAACTACCTGTTGAAGATGGCTTTGGAAAAGATCGCCTTCCT 1349
Xenopus tropicalis                CTTCTTGAAACTGTCACTAATGATAAAGAAAGTGATATCAATTATCTGTTGAAAATGGCTCTGGAGAAAATTGCCTTCCT 1358
Lipotes vexillifer                CTGCTGGACCATGTCACCAACGACACGGAAACCGACATCAATTACTTGTTAAAGATGGCACTGGAAAAAATTGCCTTCCT 1418
Orcinus orca                      CTGCTGGACCATGTCACCAACGACAAGGAAAGCGACATCAATTACTTGTTAAAGATGGCACTGGAAAAAATTGCCTTCCT 1403
Homo sapiens                      CTGCTGGACCGTGTCACCAATGACACGGAAAGTGACATCAATTACTTGCTAAAAATGGCACTGGAAAAAATTGCCTTCCT 1394

                                      1380           1390           1400           1410           1420           1430           1440           1450
conservation
Callorhinchus milii               TCCCTTTGGCTATTTGATAGACCAGTGGAGGTGGGAGTGTTCAGTGGACGCATCTCTCCATTGCATTACAACTACGAAT 1453
Alligator mississippiensis        GCCTTTTGGGTACCTTATTGACCAGTGGCGCTGGAACGTCTTCAGTGGGCACACCTCACCAAACCGCTACAACTACGACT 1438
Anolis carolinensis               TCCCTTTGGCTACCTCATAGACCAGTGGCGATGGAACGTTTTCAGTGGCCGCACCACTCCCAGCCGCTACAATTATGACT 1510
Astyanax mexicanus                TCCGTTTGGATACCTCATTGATCAGTGGCGTTGGGTGTGTTCAGTGGACGTACGCCACCTGAGAGATACAATGCAGAGT 1435
Boleophthalmus pectinirostris     TCCCTTTGGATACCTCATTGACCAGTGGAGATGGAACGTTTTCAGTGGAGATACAGCAGCAGATCGTTACAACTATGACT 1453
Cynoglossus semilaevis            TCCTTTTGGTTACCTCATTGACCAGTGGAGGTGGGGTGTGTTCAGTGGACAAACTCCTCCAGAGAAATACAACTCAGAAT 1456
Danio rerio                       TCCTTTTGGGTACCTTATTGACCAGTGGCGATGGAGTGTGTTCAGTGGAGAGACACCGCCTGACCGCTACAATGCAGATT 1558
Esox lucius                       TCCCTTTGGCTATCTTATAGACCAATGGCGATGGAACGTGTTTAGCGGTCACACTCCACCAGAGCGCTACAATGCAGACT 1444
Fundulus heteroclitus             CCCCTTCGGCTACCTCATTGACCTCTGGAGATGGGCGTGTTTAGTGGAAGCACCCCTCCGGAGAAGTACAACTCGGACT 1462
Gekko japonicus                   TCCCTTTGGCTACCTGATAGATCAGTGGCGATGGAACGTCTTCAGTGGGCGCACCCCTCCCAACCGTTACAATTACGACT 1450
Haplochromis burtoni              TCCCTTTGGCTACCTTATTGACCTCTGGAGATGGGATGTCTTTAGTGGAAAGACCCCTCCGGAGCGTTACAATGCTGACT 1453
Hippocampus comes                 TCCTTTTGGCTACCTGATCGACCAATGGAGATGGGCGTATTTGGGGGCAACATCCCCCCGGAGCGATACAACGCAGAAT 1438
Ictalurus punctatus               TCCTTTTGGATACCTCATTGATCAGTGGCGCTGGGTGTGTTCAGTGGACATACACCACCTGAACGCTACAACTCAGAGT 1444
Larimichthys crocea               TCCCTTTGGTTACCTCATCGACCAGTGGAGATGGAGTGTGTTCAGTGGACGCACACCTCCAGAGCGGTACAATGCTGACT 1447
Lates calcarifer                  GCCCTTTGGCTATCTCATTGACCAGTGGAGATGGGCGTCTTCAGTGGAAGCACTCCTAAGGAGCGGTACAACTCTGACT 1450
Latimeria chalumnae               ACCTTTTGGTTACCTGATTGACCAATGGCGTTGGGGGTGTTTAGTGGACGCATCACCCCTCAAGACTACAATTATGAGT 1432
Lepisosteus oculatus              GCCCTTCGGATACCTCATCGACCAGTGGAGGTGGGGGTGTTTAGCGGACGCACCCCACCTGAGCGCTACAATGCAGACT 1438
Macaca fascicularis               GCCCTTTGGCTACTTGGTGGACCAGTGGCGCTGGGGGTCTTTAATGGGCGTACTCCTCCTTCCCACTACAACTTCGACT 1477
Manacus vitellinus                ACCCTTTGGCTACCTCATCGACCAGTGGCGCTGGAACGTGTTCAGTGGCCGCACGCCCCCGAGCCGCTACAACTCCGACT 1429
Mus musculus                      GCCCTTTGGCTACCTGGTGGACCAGTGGCGTTGGGGGTCTTCAGTGGACGGACCCCACCCTCTCGCTACAACTTCGACT 1489
Neolamprologus brichardi          TCCCTTTGGCTACCTTATTGACCTCTGGAGATGGGATGTCTTTAGTGGAAAGACCCCTCAGGAGCGTTACAATGCTGACT 1453
Oncorhynchus mykiss               TCCCTTTGGCTATCTCATTGACCAGTGGAGATGGAGTGTGTTTAGTGGGCGCACCCCCCCAGAGCGATACAACGCAGACT 1444
Oreochromis niloticus             TCCCTTTGGCTACCTTATCGACCTCTGGAGATGGGATGTGTTTAGTGGAAAGACCCCTCCGGAGCGTTACAATGCTGACT 1453
Poecilia formosa                  TCCCTTTGGCTACCTCATTGACCTCTGGAGATGGGCGTGTTTAGTGGAAGCATTCCTCCAGAGAAGTACAACTCGGAAT 1459
Poecilia latipinna                TCCCTTTGGCTACCTCATTGACCTCTGGAGATGGGCGTGTTTAGTGGAAGCATTCCTCCAGAGAAGTACAACTCAGAAT 1459
Pseudopodoces humilis             GCCCTTTGGCTACCTCATCGACCAGTGGCGCTGGAATGTGTTCAGTGGCCACACGCCGCCAAGCCGTTACAACTACGACT 1429
Pundamilia nyererei               TCCCTTTGGCTACCTTATTGACCTCTGGAGATGGGATGTCTTTAGTGGAAAGACCCCTCCGGAGCGTTACAATGCTGACT 1453
Pygocentrus nattereri             TCCTTTTGGATACCTCATTGATCAGTGGCGCTGGGTGTGTTCAGTGGATATACACCGCCTGAACGCTACAATGCAGAGT 1426
Python bivittatus                 ACCCTTTGGCTACCTAATAGACCAGTGGCGGTGGAATGTCTTCAGTGGGCACACCCCTCCAAGCCGTTACAATTATGATT 1441
Rattus norvegicus                 GCCCTTTGGTTACCTGGTGGACCAGTGGCGCTGGGGGTCTTCAGTGGACGTACCCCACCCTCTCGCTACAACTACGACT 1492
Scleropages formosus              GCCTTTTGGGTTCCTCATTGACCAGTGGAGATGGGAGTATTCAGTGGCAAGACCCCCCCTGAGCGCTACAATGCAGATT 1438
Sinocyclocheilus anshuiensis      TCCTTTTGGATATCTTATTGATCAGTGGCGCTGGGTGTGTTTAGTGGACGACACCGCCAGAACGTTACAATGCAGAAT 1429
Xenopus tropicalis                GCCATTTGGCTTCCTGATTGATCAGTGGAGATGGAATGTCTTCAGTGGCCGCACCACTCCCACCAGATACAACTACGACT 1438
Lipotes vexillifer                GCCCTTTGGCTACTTGGTGGACCAGTGGCGTTGGGGGTCTTTAGTGGGCGTACCCCCCCTTCCCTCTACAACTATGACT 1498
Orcinus orca                      GCCCTTTGGCTACTTGGTGGACCAGTGGCGTTGGGGGTCTTTAGTGGGCGTACCCCCCCTTCCCTCTACAACTATGACT 1483
Homo sapiens                      GCCCTTTGGCTACTTGGTGGACCAGTGGCGCTGGGGGTCTTTAGTGGGCGTACCCCCCCTTCCCGCTACAACTTCGACT 1474
```

```
                                1460      1470      1480      1490      1500      1510      1520      1530
conservation
Callorhinchus milii             GGTGGTACCTGAGGACAAATACCAAGGAATATGCCCACCAATTTCTAGGAATGAAACACATTTTGATGCTGGTGCCAAG 1533
Alligator mississippiensis      GGTGGTACCTGAGAACAAAATATCAGGGAATATGTCCTCCTATTGCAAGGAATGAAACCAACTTTGATCCTGGTGCAAAG 1518
Anolis carolinensis             GGTGGTACCTGAGAACAAAGTACCAGGGGATCTGTCCTCCTATTGCAAGAAATGAAACAAACTTTGATCCTGGTGCTAAA 1590
Astyanax mexicanus              GGTGGTATCTCAGGACAAAATACCAAGGAATCTGTCCACCAGTGAGACGCACAGAAGAACACTTTGACCCCGGAGCAAAA 1515
Boleophthalmus pectinirostris   GGTGGTACCTCAGAACTAAATACCAAGGCATCTGCCCACCCACCAGACGCACAGAGGAGCACTTTGATGCAGGCGCAAAA 1533
Cynoglossus semilaevis          GGTGGTACCTCAGGACCAAGTATCAGGGAATCTGCCCCCCCACTGCACGTACTGAGGAGCACTTTGATCCTGGGGCCAAA 1536
Danio rerio                     GGTGGTACCTCAGAACAAAATACCAAGGCATCTGTCCACCAACCAGACGTACAGAAGAGCATTTTGATGCTGGAGGGAAA 1638
Esox lucius                     GGTGGCACCTCAGGACAAAATACCAGGGTATCTGTCCTCCCACTAAACGTACAGAGGAACACATGGACGCCGGAGCAAAA 1524
Fundulus heteroclitus           GGTGGTACCTAAGAACAAAATACCAAGGAATCTGCCCACCTACCAGGCGGACAGAGGATCACTTTGATCCTGGGGCAAAG 1542
Gekko japonicus                 GGTGGTATCTCAGAACAAAATACCAGGGGATTTGTCCTCCAGTAGCAAGAAATGAGACACATTTCGACCCTGGGGCCAAA 1530
Haplochromis burtoni            GGTGGTATCTCAGAACAAAATATCAAGGCATCTGCCCACCCACCAGGCGTACAGAGGAGCACTTTGATGCTGGGGCAAAA 1533
Hippocampus comes               GGTGGTACCTCAGGACAAAATATCAAGGCATCAGCCCACCCACCAAGCGCACCGAAGAGCACTTTGACGCCGGCGCTAAA 1518
Ictalurus punctatus             GGTGGTACCTCAGAACAAAATACCAAGGTATCTGTCCACCAGTAAGACGGACAGAGGAGCAGTTTGATGCTGGGGCAAAA 1524
Larimichthys crocea             GGTGGCACCTCAGGACAAAATATCAAGGTATCTGCCCACCGACCAGGCGTACAGAGGAGCACTTTGATCCTGGGGCAAAA 1527
Lates calcarifer                GGTGGTACCTCAGGACAAAATATCAAGGTATTTGCCCACCCACCAGACGTACAGAGGAGCACTTTGATGCTGGGGCAAAA 1530
Latimeria chalumnae             GGTGGTACCTCAGGACAAAATACCAGGGAATCTGTCCCCCAGTTGCACGCAGTGCAATCCACTTTGACCCCGGTGCAAAA 1512
Lepisosteus oculatus            GGTGGTACCTGAGAACGAAATACCAGGGGATCTGCCCACCAATCGAACGCAGTGAGGAGCACTTTGATGCTGGGGCCAAA 1518
Macaca fascicularis             GGTGGTATCTTCGAACCAAGTATCAGGGGATCTGTCCTCCTGTTACCCGAAACGAAACCCACTTTGATGCTGGAGCTAAG 1557
Manacus vitellinus              GGTGGTACCTGAGAACCAAATACCAGGGTATCTGTGCCCCTGTCACGAGGAATGAGAGCAACTTTGACCCTGGAGCAAAG 1509
Mus musculus                    GGTGGTATCTTCGAACCAAGTATCAGGGGATCTGCCCACCAGTTGCCCGGAATGAAACCCATTTTGATGCTGGAGCCAAG 1569
Neolamprologus brichardi        GGTGGTATCTCAGGACAAAATATCAAGGCATCTGCCCACCCACCAGGCGTACAGAGGAGCACTTTGATGCTGGGGCAAAA 1533
Oncorhynchus mykiss             GGTGGCACCTCAGGACAAAATACCAGGGTATATGTCCACCCACCAAACGCACAGAGGAGCACATGGACGCTGGAGCCAAA 1524
Oreochromis niloticus           GGTGGTATCTCAGAACAAAATATCAAGGCATCTGCCCACCCACCAGGCGTACAGAGGAGCACTTTGATGCTGGGGCAAAA 1533
Poecilia formosa                GGTGGTACCTCAGAACAAAGTACCAAGGAATTTGCCCACCTACCAGCCGCACAGAGGAGCACTTTGATCCCGGAGCAAAG 1539
Poecilia latipinna              GGTGGTACCTCAGAACAAAGTACCAAGGAATTTGCCCACCTACCAGCCGCACAGAGGAGCACTTTGATCCCGGAGCAAAG 1539
Pseudopodoces humilis           GGTGGTATCTGAGAACCAAATACCAGGGTATCTGTGCTCCAGTTCCAAGGAATGAAAGCAACTTTGACCCTGGAGCAAAG 1509
Pundamilia nyererei             GGTGGTATCTCAGAACAAAATATCAAGGCATCTGCCCACCCACCAGGCGTACAGAGGAGCACTTTGATGCTGGGGCAAAA 1533
Pygocentrus nattereri           GGTGGTACCTCAGGACAAAATACCAAGGCATCTGTCCACCAGTAAGACGGACAGAGGAGTTCTTTGACCCTGGGGCAAAA 1506
Python bivittatus               GGTGGTACCTAAGAACAAAATACCAGGGAATTTGTCCTCCTATTGCAAGAAATGAAACAAACTTTGATCCCGGTGCTAAA 1521
Rattus norvegicus               GGTGGTATCTTCGAACCAAGTATCAGGGGATCTGCCCACCAGTTGCTCGGAATGAAACCCATTTTGACGCTGGGGCCAAG 1572
Scleropages formosus            GGTGGTATCTGAGAACTAAATACCAAGGAATCTGCCCTCCAACAAAGCGCACAGAGGAACATTTTGATGCTGCTGCCAAA 1518
Sinocyclocheilus anshuiensis    GGTGGTACCTCAGGACAAAATACCAAGGCATCTGTCCACCAACCAGACGTACTGAAGAGCATTTTGATGCTGGAGCAAAA 1509
Xenopus tropicalis              GGTGGAACCTTCGAACAAAATATCAAGGTATCTGCCCACCAATCTCAAGAGATGATAACCAATTTGATGCTGGGGCAAAG 1518
Lipotes vexillifer              GGTGGTATCTTAGAACCAAGTATCAGGGGATCTGTCCTCCAGTTGTCCGAAATGAAACCCACTTTGACGCTGGAGCCAAG 1578
Orcinus orca                    GGTGGTATCTTCGAACCAAGTATCAGGGGATCTGTCCTCCAGTTGTCCGAAACGAAACCCACTTTGACGCTGGAGCCAAG 1563
Homo sapiens                    GGTGGTATCTTCGAACCAAGTATCAGGGGATCTGTCCTCCTGTTACCCGAAACGAAACCCACTTTGATGCTGGAGCTAAG 1554
```

```
                                1540      1550      1560      1570      1580      1590      1600      1610
conservation
Callorhinchus milii             TATCACGTCCAGGAAACACACCTTACATCAGATATTTGTGAGTTGTATTCTGCAATTCCAGTTTCATAAAGCTCTGTG 1613
Alligator mississippiensis      TATCATATCCCTGCCAACACACCCTACATCAGATACTTTGTGAGCTTCATCCTGCAGTTCCAGTTCCACAAGGCCCTGTG 1598
Anolis carolinensis             TATCACATTCCTGGCAATACACCTTATATCAGGTATTTTGTTAGTTACATTGTGCAGTTCCAGTTTCATAAGGCTCTTTG 1670
Astyanax mexicanus              TACCACATCCCAGGAAATACTCCTTACATCAGGTACTTTGTGAGCTTCATTCTTCAGTTCCAGTTTCATCAGAAGCTTTG 1595
Boleophthalmus pectinirostris   TACCACGTCCCCGGAAACACACCATACATCAGGTATTTTGTCAGCTTCATTTTGCAGTTCCAATTCCATGAAAAACTTTG 1613
Cynoglossus semilaevis          TACCACATCCCTGGAAACACGCCTTACATCAGATATTTCGTCAGCTTCATCCTTCAGTTCCAGTTTCATGAAAAACTGTG 1616
Danio rerio                     TATCACATCCCTGGAAATACACCGTACATAAGATACTTTGTGAGCTTCATTCTGCAGTTCCAGTTCCATGAGAAATTATG 1718
Esox lucius                     TACCACATTCCAGGAAATACTCCTTACATCAGATACTTTGTGAGTTTCATTCTGCAGTTCCAGTTCCACAAGAAGCTGTG 1604
Fundulus heteroclitus           TATCATATCCCCGGAAACACACCATATATCAGGTACTTTGTTAGCTTTATCCTCCAGTTCCAGTTTCATGAAAAACTCTG 1622
Gekko japonicus                 TACCACATCCCTGGAAACACGCCTTACATCAGGTACTTTGTCAGTTTCATCCTGCAGTTCCAGTTCCACAAGGCCCTCTG 1610
Haplochromis burtoni            TACCACATCCCTGGGAACACGCCGTACATCAGATATTTTGTCAGTTTCATCCTTCAGTTCCAGTTTCATGAAAAACTTTG 1613
Hippocampus comes               TTCCACATTCCTGGAAACACACCGTACATCAGGTATTTTGTGAGCTTCATCCTGCAGTTTCAGTTCCACGAAAAACTATG 1598
Ictalurus punctatus             TATCATATTCCAGGAAATACTCCATACATCAGATACTTTGTGAGCTTCATCCTGCAGTTCCAGTTTCACCAGAAGCTTTG 1604
Larimichthys crocea             TACCACATCCCTGGAAACACCCCATACATTAGGTATTTTGTCAGCTTCATCCTGCAGTTCCAGCTTCATGAAAAACTGTG 1607
Lates calcarifer                TACCACATCCCTGGAAACACACCATATATCAGGTATTTTGTCAGCTTCATCCTGCAGTTCCAGTTTCATGAAAAACTATG 1610
Latimeria chalumnae             TATCACATCCCTGCCAACACTCCGTACATCAGATATTTTGTGAGTTTCATTCTTCAGTTCCAGTTTCACAAGGCACTATG 1592
Lepisosteus oculatus            TTCCACATTCCAGGAAATACTCCATATATTAGGTATTTTGTCAGCTTCATCCTTCAGTTCCAGTTCCATGAGAAACTGTG 1598
Macaca fascicularis             TTTCATGTTCCAAATGTGACACCATACATCAGGTACTTTGTGAGTTTCGTCCTGCAGTTCCAGTTCCATGAAGCCCTGTG 1637
Manacus vitellinus              TACCACATCCCTGGGAACACCCCTTACATCAGGTACTTTGTGAGCTTCATCCTCCAGTTCCAGTTCCACAAGGCGCTGTG 1589
Mus musculus                    TTTCACATCCCAAACGTGACACCGTACATCAGGTACTTCGTGAGCTTTGTGCTGCAGTTCCAGTTCCATCAAGCACTGTG 1649
Neolamprologus brichardi        TACCACATCCCTGGGAACACGCCGTACATCAGATATTTTGTCAGTTTCATCCTTCAGTTCCAGTTTCATGAAAAACTTTG 1613
Oncorhynchus mykiss             TATCACATTCCAGGAAACACCCCATACATCAGGTACTTTGTGAGCTTCATTCTGCAGTTCCAGTTCCACAAGAAGCTGTG 1604
Oreochromis niloticus           TACCACATCCCTGGGAACACGCCGTACATCAGATATTTTGTCAGCTTCATCCTTCAGTTCCAGTTTCATGAAAAACTTTG 1613
Poecilia formosa                TATCATATTCCTGGAAACACGCCATACATCAGGTACTTTGTCAGCTTCATCCTCCAGTTCCAGTTTCATGAGAAACTCTG 1619
Poecilia latipinna              TATCATATTCCTGGAAACACGCCATACATCAGGTACTTTGTCAGCTTCATCCTCCAGTTCCAGTTTCATGAGAAACTCTG 1619
Pseudopodoces humilis           TACCACATCCCGGGGAACACTCCTTACATCAGGTACTTTGTGAGCTTCATCCTCCAGTTCCAGTTTCACAAGGCACTGTG 1589
Pundamilia nyererei             TACCACATCCCTGGGAACACGCCGTACATCAGATATTTTGTCAGTTTCATCCTTCAGTTCCAGTTTCATGAAAAACTTTG 1613
Pygocentrus nattereri           TATCATATCCCAGGAAATACTCCCTACATCAGGTATTTTGTGAGCTTCATCCTTCAGTTCCAGTTTCATCAGAAACTTTG 1586
Python bivittatus               TACCACATCCCTGGCAACACACCTTACATCAGGTACTTTGCCAGTTTTATTCTACAATTCCAGTTCCACAAGGCCCTTTG 1601
Rattus norvegicus               TTTCACATCCCAAGCGTGACACCATACATCAGGTACTTTGTGAGTTTCGTGCTACAGTTCCAGTTCCATCAAGCGCTGTG 1652
Scleropages formosus            TACCATATCCCTGGAAACACACCATATATCAGATATTTTGTGAGCTTTATTCTGCAGTTCCAGTTCCATGAGAGGTTGTG 1598
Sinocyclocheilus anshuiensis    TATCACATTCCTGGAAACACACCGTACATCAGATACTTTGTGAGCTTCATTCTGCAGTTCCAGTTCCATGAGAAATTATG 1589
Xenopus tropicalis              TATCATATTCCAGGCAACACACCTTACATCAGGTACTTTGTGAGTTTTGTGCTGCAGTTCCAGTTCCATAAGGCTCTCTG 1598
Lipotes vexillifer              TTTCATGTCCCAAATGTGACACCATACATCAGGTACTTTGTGAGCTTCGTCCTGCAGTTCCAGTTCCACCAAGCCCTGTG 1658
Orcinus orca                    TTTCATGTCCCAAATGTGACACCATACATCAGGTACTTCGTGAGTTTTGTCCTGCAGTTCCAGCTCCATCAAGCCCTGTG 1643
Homo sapiens                    TTTCATGTTCCAAATGTGACACCATACATCAGGTACTTTGTGAGTTTTGTCCTGCAGTTCCAGTTCCATGAAGCCCTGTG 1634
```

```
                                1620      1630      1640      1650      1660      1670      1680      1690
conservation
Callorhinchus milii            CAATGCTTCAAACCATACTGGAGCTCTACATACATGTGATATCTATAAATCCACGGTCGCTGGTGAGAAGCTCAGACAAG  1693
Alligator mississippiensis     CCAAGCTGCCAATCATACAGGCCCCCTGCACACCTGTGACATCTACCAGTCCAAAGAAGCTGGAACAAAACTCAGTGAGA  1678
Anolis carolinensis            CCAAGCTGCCAACCACACCGGCCTCTTCATACCTGTGATATCTACCAGTCCAAAGAAGCTGGAAGGCTGCTCAGCGCAG  1750
Astyanax mexicanus             TCAGGAGGCTGGACACACTGGACCACTGCACACCTGTGATATCTACAGATCTACCCAGGCTGGAGCAGTTCTAGAGAAAG  1675
Boleophthalmus pectinirostris  TGAAGCCGCTAAACACACTGGACCACTGCACACATGTGACATCTACCGCTCAAAGGAAGCTGGAGCCATTTTGAAGAAGG  1693
Cynoglossus semilaevis         TAAGGCTGCCAAACACACAGGTCCTCTGCATACATGTGACATCTACAAGTCCAAAGAGGCTGGAGCCATTCTAAAGAAGG  1696
Danio rerio                    CAAAGAGGCTGGACATACTGGACCTCTGCACAAATGTGACATCTACAAATCACGTGAAGCTGGAGCTGTTCTAGAGAAAG  1798
Esox lucius                    TGATGCGGCAGGACACCGTGGGCCCCTGCACACATGTGACATTTATCAGTCCAAAGAGGCAGGAAAGATTTTAGAGACGG  1684
Fundulus heteroclitus          TGATGCAGCAAATCACAAGGGGCCTCTGCACACATGCGACATCTACCGTTCTAAAGAAGCTGGAGACATCCTACGAACGG  1702
Gekko japonicus                CCAGGCTGCCAACCACACCGGCCCTCTCCACACCTGTGACATCTACCAGTCCAGAGAAGCTGGAAGGAAACTCAGCGAGG  1690
Haplochromis burtoni           CGAAGCAGCCAAGCACACAGGTCCCTTGCACACATGTGACATCTACCGGTCTGCGGAGGCGGGAGCCATTTTAAAGAAAG  1693
Hippocampus comes              CCAAGCGGCCAAACACACTGGACCCTTGCACACATGCGACATCTACCGCTCTGCAGAAGCGGGGACCATTTTAAAAAAAG  1678
Ictalurus punctatus            CCAGGAAGCTGGACACACTGGACCGCTGCATAAGTGTGACATCTATAGATCAGCCAAGGCTGGGGCTGTTTTAGAAAAAA  1684
Larimichthys crocea            CGCCGCAGCCAATCAAACCGGCCCCTTGCACACATGTGACATATACCGTTCCGCAGCGGCAGGGGCCATTTTAAAGAAAA  1687
Lates calcarifer               CGAAGCAGCGAAACACACTGGTCCTTTGCACACATGTGACATCTACCGCTCCAAAGAAGCAGGCGCCATCTTAAAGAAAG  1690
Latimeria chalumnae            TAAAGCGGCAGGTCATACAGGCCCATTACACACCTGTGACATCTACAAGTCCAAGATAGCAGGAGCTGAGCTCAGTAAGG  1672
Lepisosteus oculatus           CGAAGCAGCCAAACATGTGGGACCATTGCACCAGTGTGACATCTATCGTTCCAAGGAAGCAGGAGCTATCCTACAAAAGG  1678
Macaca fascicularis            CAAGGAGGCAGGCTATGAGGGCCCACTGCACCAATGTGACATCTACCAGTCCACCAAGGCAGGGGCCAAGCTCCGGAAGG  1717
Manacus vitellinus             CGAGGCAGCCGGACACAGCGGCCCCCTGCACACCTGTGACATCTACAGGTCCAGAGAGGCTGGAGACAAGCTCAGGGAAG  1669
Mus musculus                   CAAGGAGGCAGGCCACCAGGGCCCACTACACCAGTGTGACATCTACCAGTCCGCCCAGGCGGGGGCCAAGCTCAAGCAGG  1729
Neolamprologus brichardi       CGAAGCAGCCAAGCACACAGGTCCCTTGCACACATGTGACATCTACCGGTCTGCAGAGGCGGGAGCCATTTTAAAGAAAG  1693
Oncorhynchus mykiss            TCAGGCAGCCCATCAGACAGGACCCCTGCACACATGTGACATCTACCAGTCCAAGGAGGCTGGAAAAATTTTAGAGACAG  1684
Oreochromis niloticus          CGAAGCAGCCAGGCACACAGGTCCCTTGCACACATGTGACATCTACCGGTCTGCAGAGGCGGGAGCCATTTTAAAGAAAG  1693
Poecilia formosa               TGAGGCAGCCAAGCACACTGGACCTCTACACACCTGCGACATCTACCGTTCCGCAGAGGCTGGAGAGATTCTACGAAAAG  1699
Poecilia latipinna             TGAGGCAGCCAAGCACACCGGGCCTCTGCACACCTGCGACATCTACCGTTCCACAGAGGCTGGAGAGATTCTACGAAAAG  1699
Pseudopodoces humilis          CAAGGCAGCCAACCACAACGGTTCCTTGCACACCTGTGACATCTACAGGTCCAAAGAGGCTGGAGCCAAACTCAGGGAAG  1669
Pundamilia nyererei            CGAAGCAGCCAAGCACACAGGTCCCTTGCACACATGTGACATCTACCGGTCTGCGGAGGCGGGAGCCATTTTAAAGAAAG  1693
Pygocentrus nattereri          CCAGGTGGCTGGGCACACTGGACCCCTGCACAAGTGTGACATCTACAGATCAACCCAGGCTGGAGCTGTTTTGGAGAAAG  1666
Python bivittatus              CCAAGCTGCCAACCATACTGGCGATCTTCATACCTGCGATATCTATAACTCCAAAGAAGCTGGAAAGAAACTCAGTGAGG  1681
Rattus norvegicus              CAAGGAGGCAGGCCACCAGGGTCCACTACACCAGTGTGACATCTACCAGTCCACCAAGGCAGGGGCCAAGCTCCAACAGG  1732
Scleropages formosus           CCAGGCAGCCAACCACACAGGGCCATTGCACCAGTGCGACATTTTCCAATCCACTGAAGCAGGCAAAATCCTGGAAACGG  1678
Sinocyclocheilus anshuiensis   TCAGGAGGCTGGACATACTGGAACTCTGCACAAATGCGATATCTACAGATCCAAAGAGGCTGGAGCTGTTTTAGAGAAAG  1669
Xenopus tropicalis             TACAGCAGCCAAACATACAGGTCCCCTGCATACATGTGATATTTACAGATCCCAGGAGGCTGGAAAAATTCTTGGAGATG  1678
Lipotes vexillifer             CAAGGAGGCGGGCCACCAGGGCCCCCTGCACCAGTGTGACATCTACCAGTCCACCCAGGCAGGGGCCAAGCTCCGGGCAT  1738
Orcinus orca                   CAAGGAGGCGGGCCACCAGGGCCCGCTGCACCAGTGCGACATCTACCAGTCCACCCAGGCGGGGGCCAAGCTCCGGGCAC  1723
Homo sapiens                   CAAGGAGGCAGGCTATGAGGGCCCACTGCACCAGTGTGACATCTACCGGTCCACCAAGGCAGGGGCCAAGCTCCGGAAGG  1714
```

```
                                1700      1710      1720      1730      1740      1750      1760      1770
conservation
Callorhinchus milii            CACTGAGGGCTGGCTCTTCCAGATCTTGGAGGAGATTCTTAAGGAAGTGACAGGTGATGGGAAGATGGATGCCTCTGCT  1773
Alligator mississippiensis     TGCTGAAAGTTGGTTCTTCCAAGCCATGGCAAGAAATCCTGTTCACTATGACTGGCACCAGAAAGATGGATGCTGGGGCA  1758
Anolis carolinensis            CATTGAGGCCTGGATCTTCCCAGCCCTGGCAGGATATTCTTCAACAAATCACTGGCACAAATAAGATGGATGCTGCACCA  1830
Astyanax mexicanus             TCCTGCAGGCTGGCTCCTCTAAGCCCTGGACTGAAGTGCTGCAAGAAGCTTTGGGCACAAATAAGATGGACGCCGCCGCC  1755
Boleophthalmus pectinirostris  TTCTTGAGGCGGGATCTTCCCAGGCTTGGGAGGAGGTTCTTCTGAATGCGTTAGGGACTAGTAAGATGGATTCTTCATCA  1773
Cynoglossus semilaevis         TACTTGTAGCAGGTTCCTCTAAACCTTGGCCTGAGGTTCTGCAGGAGGCTATAGGAACCAACAAGATGGACGCCACCTCC  1776
Danio rerio                    TCTTGAAGGCTGGCTCTTCAGAACCTTGGACTCAGGTGCTACAAGAGGCGCTGGGCACTGACAAAATGGACGCCACCCCC  1878
Esox lucius                    TCCTTAAATCAGGAGAATCCAAACCTTGGCAAAATGTGCTCCAGGAAGCCATTGGTACAGACAAGATGAGCGCCAGTGCA  1764
Fundulus heteroclitus          TTCTAGAGGCAGGTTCTTCAAACCTTGGCCCGAGGTCCTTCAAGAGGCCATCGGCACGAATCAAATGAATGCTACCTCT  1782
Gekko japonicus                CGCTGAGGGCTGGGTCTTCCGAGCGGTGGCAGGATATCCTCCAGCAACTCACCGGCACAGACCAGATGGATGCGACGGCA  1770
Haplochromis burtoni           TCCTTCAGGCCGGCTCATCTGAAAGCTGGCCAGTTGTGCTCCAGGATGCTATAGGCACTAATAAATTAAATGCCAGCTCA  1773
Hippocampus comes              TTCTTCAGGCGGGTTCCTCCAAACCTTGGCCTGAAGTGCTTCAAGAGGCCATCGGGACGAACAAGATGGACGCAGGCGCA  1758
Ictalurus punctatus            TCATGCAGTTCGGTTCCTCCAAGCCCTGGACTGAGGTGCTACAAGAGACTTTGGGCACAAACAAAATGGACGCCAGTGCC  1764
Larimichthys crocea            TTCTTCAGGCTGGCTCCTCTAAGCCATGGCCTGACGTGCTCCAGGAAGCTATAGGCACCAGGAAGTTGGATGCTAACTCA  1767
Lates calcarifer               TTCTCCAGGCTGGCTCTTCCAAACCTTGGCCTGATGTGCTTCAGGAGGCAATAGGGACCAACAAAATGGATGCTAGCTCA  1770
Latimeria chalumnae            TTCTACAGGCTGGTTCCTCTAAACCATGGCAAGAAATCCTAGAGGGTCTAACAGGGGAACGTCACATGAATGCTGGTCCC  1752
Lepisosteus oculatus           TCCTCAGCGCTGGCTCCTCGAAGCCCTGGCAGGATATCCTGCAAGAAGCCTTGGGAACAAACCGAATGGATGCTGGACCT  1758
Macaca fascicularis            TGCTGCAGGCTGGCTCCTCCAGGCCCTGGCAGGAGGTGCTGAAGGACATGGTCGGCTCAGACGCCCTGGACGCCCAGCCA  1797
Manacus vitellinus             TGTTGAAAGCTGGGTCTTCCAAGTCGTGGCAGGAAGTCCTGTTCAACTTCACTGGCACGGATAAGATGGATGCTGGGCCC  1749
Mus musculus                   TGTTGCAGGCTGGCTGCTCCAGGCCCTGGCAGGAGGTACTGAAGGACCTGGTAGGCTCAGATGCCCTGGATGCCAAGGCA  1809
Neolamprologus brichardi       TCCTTCAGGCCGGCTCATCTGAAAGCTGGACAGTTGTGCTCCAGGATGCTATAGGCACTAATAAATTAAATGCCAGCTCA  1773
Oncorhynchus mykiss            TCCTTAAATCCGGAGAATCCAAGCCCTGGGAACAGGTGCTCCAGGAAGCAGTCGGGACAAACAAGATTGACGCCAGCTCC  1764
Oreochromis niloticus          TCCTTCAGGCCGGCTCATCTGAAAGCTGGCCAGTTGTGCTCCAGGATGCTATAGGCACTGATAAATTAAACGCCAGCTCC  1773
Poecilia formosa               TTCTTGAAGCAGGTTCCTCTAAGCCCTGGCCTGAAGTCCTCCAAGAAGCTATAGGCACAAATAAGATGAATGCCACCTCC  1779
Poecilia latipinna             TTCTTGAAGCAGGTTCCTCTAAGCCCTGGCCTGAAGTTCTCCAAGAAGCTATAGGCACAAATAAGATGAACGCCACCTCC  1779
Pseudopodoces humilis          TGTTGAAAGCTGGGTCCTCGAAGCCATGGCAGGATATCCTCTTGAATCTCACTGGCATGGGTCAGATGGACGCTGCTCCC  1749
Pundamilia nyererei            TCCTTCAGGCCGGCTCATCTGAAAGCTGGCCAGTTGTGCTCCAGGATGCTATAGGCACTAATAAATTAAATGCCAGCTCA  1773
Pygocentrus nattereri          TCTTGCGTGCCGGCTCATCTAGGCCCTGGACGGAAGTGCTCCAAGATGCTTTGGGCACAAACAAAATGGATGCCAGTGCC  1746
Python bivittatus              CACTGAAGGCTGGATCTTCCCGGCCTTGGCAAGATATTCTCCAACAACTCACTGGTACAAATAAGATGGATGCTTCGGCA  1761
Rattus norvegicus              TGCTGCAGGCTGGCTGCTCCAGGCCCTGGCAGGAGGTGCTGAAGGACCTGGTGGGTTCAGATGCGCTGGATGCCAGTGCG  1812
Scleropages formosus           TCCTGAAAGCAGGTTCATCCAGACCATGGAGAGAAGTTCTGATGGAAGCTGTGGGGACTGGTGAGATGGATGCCAGTGCC  1758
Sinocyclocheilus anshuiensis   TCTTGAAGGCTGGCTCTTCAAAGCCTTGGACAGAGGTGCTACAAGAGGCTCTTGGCACTAACAGAATGGACGCCAGCCCC  1749
Xenopus tropicalis             TTCTCCGGTCAGGATCCTCCAAGTCTTGGCAGGAGGTTCTGAAGGACATGACAGGTAGTGAAAAGATGGATGTTGGGCCC  1758
Lipotes vexillifer             TGCTGCAGGCAGGCTCCTCGCGGCCCTGGCAGGAGGTGCTGAAGGACATGGTCGGCTCAGATTCCCTGGATGCTCAGCCG  1818
Orcinus orca                   TGCTGCAGGCAGGCTCCTCACGGCCCTGGCAGGAGGTGCTGAAGGACATGGTCGGCTCAGATTCCCTGGACGCTCAGCCG  1803
Homo sapiens                   TGCTGCAGGCTGGCTCCTCCAGGCCCTGGCAGGAGGTGCTGAAGGACATGGTCGGCTTAGATGCCCTGGATGCCCAGCCG  1794
```

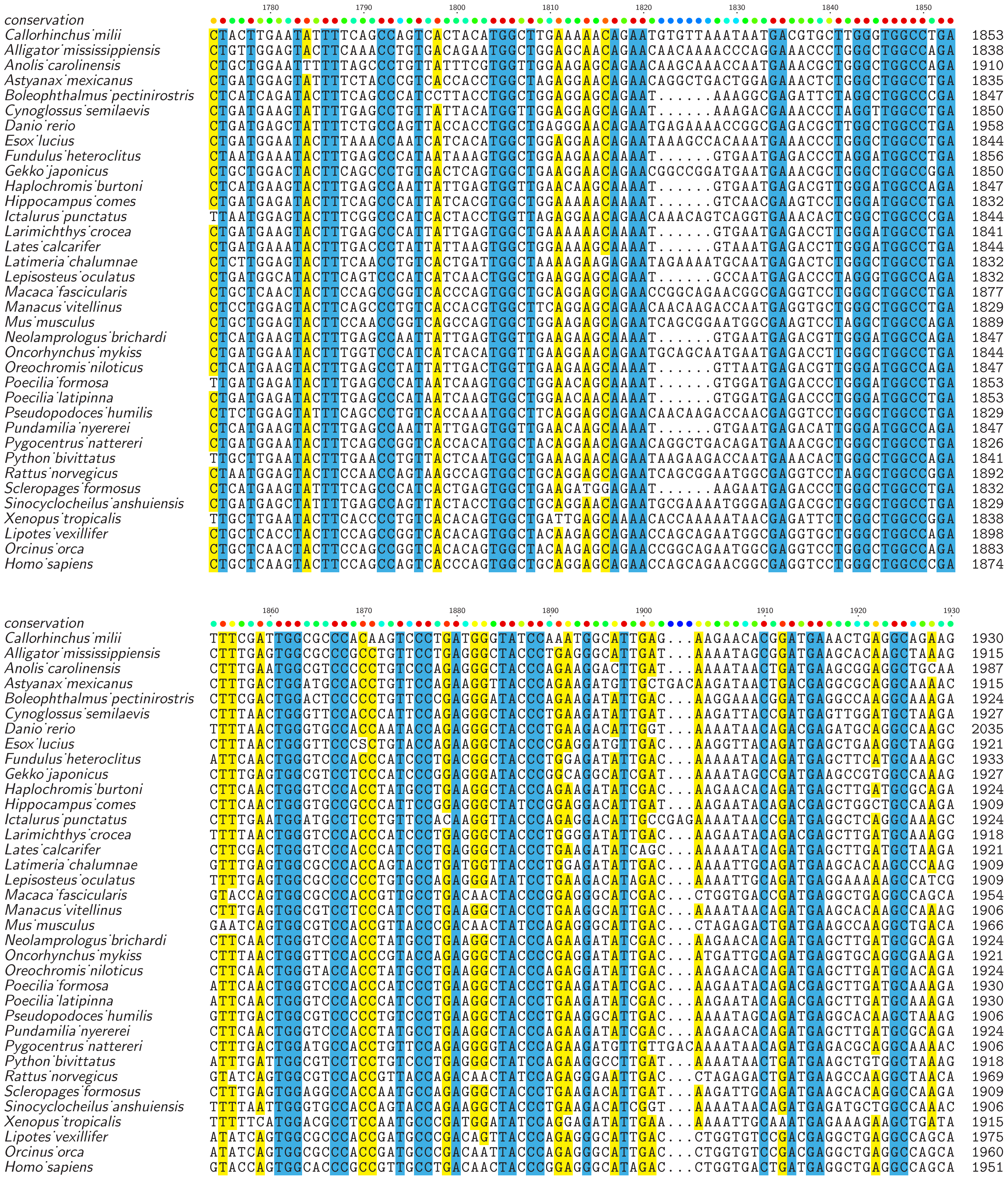
conservation
Callorhinchus milii
Alligator mississippiensis
Anolis carolinensis
Astyanax mexicanus
Boleophthalmus pectinirostris
Cynoglossus semilaevis
Danio rerio
Esox lucius
Fundulus heteroclitus
Gekko japonicus
Haplochromis burtoni
Hippocampus comes
Ictalurus punctatus
Larimichthys crocea
Lates calcarifer
Latimeria chalumnae
Lepisosteus oculatus
Macaca fascicularis
Manacus vitellinus
Mus musculus
Neolamprologus brichardi
Oncorhynchus mykiss
Oreochromis niloticus
Poecilia formosa
Poecilia latipinna
Pseudopodoces humilis
Pundamilia nyererei
Pygocentrus nattereri
Python bivittatus
Rattus norvegicus
Scleropages formosus
Sinocyclocheilus anshuiensis
Xenopus tropicalis
Lipotes vexillifer
Orcinus orca
Homo sapiens

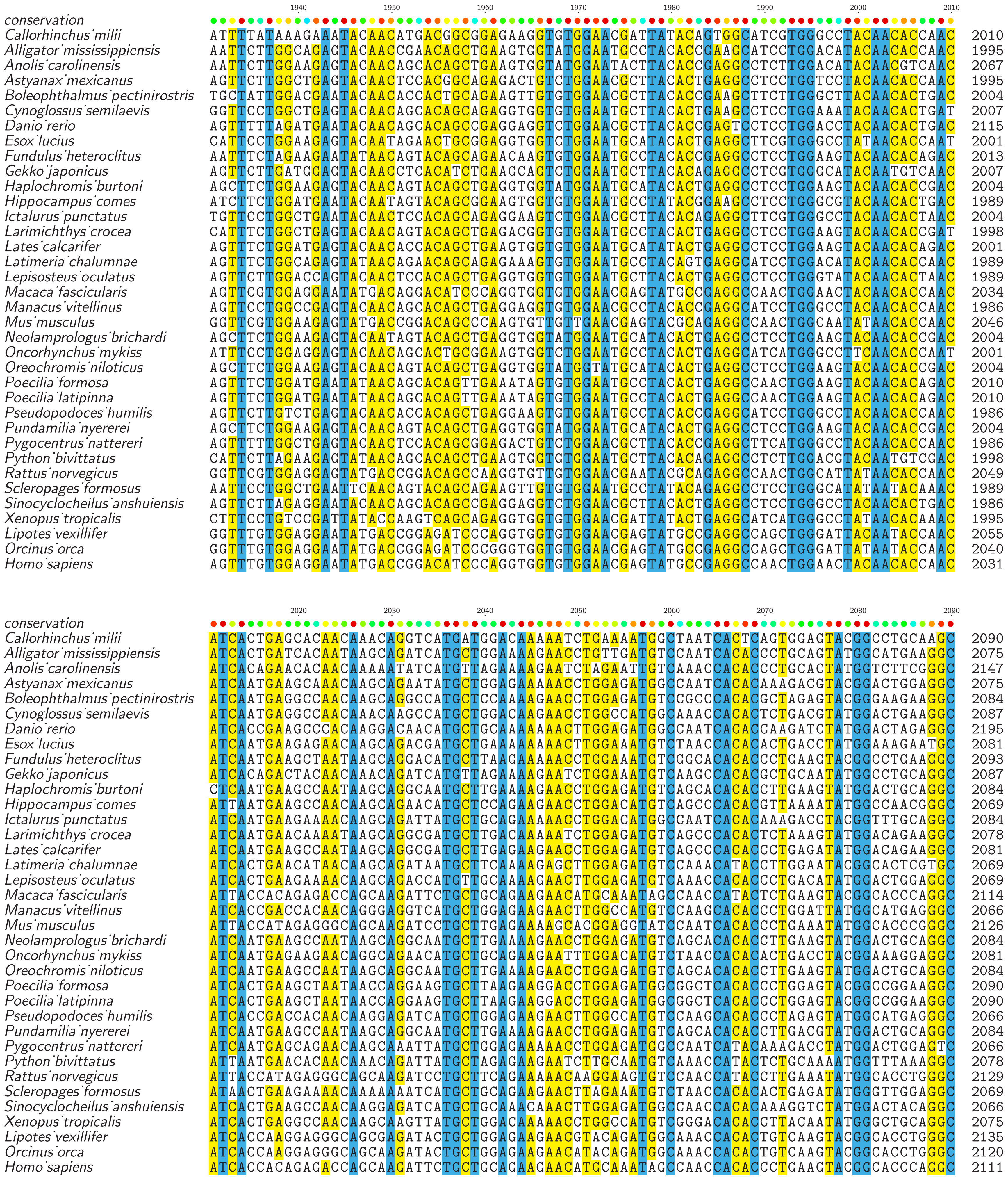

conservation
Callorhinchus milii 2010
Alligator mississippiensis 1995
Anolis carolinensis 2067
Astyanax mexicanus 1995
Boleophthalmus pectinirostris 2004
Cynoglossus semilaevis 2007
Danio rerio 2115
Esox lucius 2001
Fundulus heteroclitus 2013
Gekko japonicus 2007
Haplochromis burtoni 2004
Hippocampus comes 1989
Ictalurus punctatus 2004
Larimichthys crocea 1998
Lates calcarifer 2001
Latimeria chalumnae 1989
Lepisosteus oculatus 1989
Macaca fascicularis 2034
Manacus vitellinus 1986
Mus musculus 2046
Neolamprologus brichardi 2004
Oncorhynchus mykiss 2001
Oreochromis niloticus 2004
Poecilia formosa 2010
Poecilia latipinna 2010
Pseudopodoces humilis 1986
Pundamilia nyererei 2004
Pygocentrus nattereri 1986
Python bivittatus 1998
Rattus norvegicus 2049
Scleropages formosus 1989
Sinocyclocheilus anshuiensis 1986
Xenopus tropicalis 1995
Lipotes vexillifer 2055
Orcinus orca 2040
Homo sapiens 2031
conservation
Callorhinchus milii 2090
Alligator mississippiensis 2075
Anolis carolinensis 2147
Astyanax mexicanus 2075
Boleophthalmus pectinirostris 2084
Cynoglossus semilaevis 2087
Danio rerio 2195
Esox lucius 2081
Fundulus heteroclitus 2093
Gekko japonicus 2087
Haplochromis burtoni 2084
Hippocampus comes 2069
Ictalurus punctatus 2084
Larimichthys crocea 2078
Lates calcarifer 2081
Latimeria chalumnae 2069
Lepisosteus oculatus 2069
Macaca fascicularis 2114
Manacus vitellinus 2066
Mus musculus 2126
Neolamprologus brichardi 2084
Oncorhynchus mykiss 2081
Oreochromis niloticus 2084
Poecilia formosa 2090
Poecilia latipinna 2090
Pseudopodoces humilis 2066
Pundamilia nyererei 2084
Pygocentrus nattereri 2066
Python bivittatus 2078
Rattus norvegicus 2129
Scleropages formosus 2069
Sinocyclocheilus anshuiensis 2066
Xenopus tropicalis 2075
Lipotes vexillifer 2135
Orcinus orca 2120
Homo sapiens 2111

```
                                 2100      2110      2120      2130      2140      2150      2160      2170
conservation
Callorhinchus milii              TCGCCAGTTTGACTCCACAAATTTCCAGAATGAAGCAACTAAACGGATCCTGAACAAGCTGAGCGACATTCAGCAGGCAG  2170
Alligator mississippiensis       CCGGCAGTTTGACTACTCCGACTTTCAGGACCAGAGCATGAAGCGCATCCTTAAGAAGCTGAGCGACATTGAGAGGGCTG  2155
Anolis carolinensis              TCGGACATTTGATCCCACTGATTTTAAAGACCCCAGCATAAAGCGTATTCTGAAGAAACTGAGTGATATCGAGCGGGCAG  2227
Astyanax mexicanus               CCGCAAGTACGACACCTCTGACTTCCAGGACTCTTCAGTGAAGAGGATTCTGAAGAAACTGAGTGACCTAGAGAGAGCTG  2155
Boleophthalmus pectinirostris    GCGTCAGTTTGACACCACGGACTTCCAGGACGCGCAGGTCAAACGTATCATCAAGAAACTCAGTGACATCGAAAGAGCTG  2164
Cynoglossus semilaevis           TCGCCAGTTCGACACCACTGACTTCCAGGACCCCTCAGTAAAACGAATTATAGAGAAACTGAGTGATATTGAACGAGCAG  2167
Danio rerio                      CCGTAAATACGACACCAGTGACTTCCAGGACGAGTCTGTGAAGAGGATCCTGAGCAAACTGAGTGATCTCGAGAGGGCTG  2275
Esox lucius                      ACGCAAGTACGATACCACAGACTTCCAGGATAATTCCGTGAAGCGTATCCTCAAGAAACTCAGTGACATTGAAAGGGCTG  2161
Fundulus heteroclitus            TCGTGATTATGACATCACTGACTTCCAGGATCCTTCAGTCAAACGCATCATCCAAAAACTCAGCGACATTGACAGGGCTG  2173
Gekko japonicus                  TCGGCAGTTTGATCCGACTGACTTCCAGGACCCCAGCATCAAGCGCATTTTAAAGAAGCTGAGTGACATTGAGCGGGCAG  2167
Haplochromis burtoni             CCGTCAATACGACACCACTGACTTCCAAGATGGCTCGGTCAAACGCATCATTAAAAAACTCAGTGACATCGAGAGGGCTG  2164
Hippocampus comes                ACGGCAGTTCGACACGGCGGACTTCCAGGAAGACGCAGTCAAACGCATCATCAAAAAACTCAGCGACATCGAGAGGGCCG  2149
Ictalurus punctatus              CCGCAAGTATGACACTACTGATTTCCAGGACTACTCAGTGAAAAGGATCCTAAAAAAACTCAGTGATCTGGAGAGGGCTG  2164
Larimichthys crocea              CCGTCTGTACGACACCACTGACTTCCAGGATGCCTCGACCAAACGCATCATCAAAAAACTCAGCGACATCGAGAGGGCCG  2158
Lates calcarifer                 CCGTCAGTACGACACCACTGACTTCCAGGACCCCTCAGTCAAACGCATCATCAAAAAACTCAGCGACATTGAGCGGGCTG  2161
Latimeria chalumnae              TCACAAGTTTGACTACACTGACTTCTTGGATGGTAGTAGTAAACGTATTCTGAAGAAACTGAGTGAACTAGACAAGGCAG  2149
Lepisosteus oculatus             ACGGAAGTTTGATTACACTGATTTCCAGGATGAATCGGTGAAGCGCATTCTGAAGAAACTGAGTGAGATTGAGCGTGCGG  2149
Macaca fascicularis              CAGGAGGTTTGATGTGAACCACTTCCAGAACACCACGATCAAGCGGATCATAAAGAAGGTTCAGGACCTAGAACGGGCAG  2194
Manacus vitellinus               CAGGCAGTTCGACACCTCCGACTTCCAGGACGAAAGTGTCACCCGCATCCTCAAGAAGCTGAGCGTCATCGAGAGGGCAG  2146
Mus musculus                     CAAGACATTTGATGTGAGCAACTTTCAAAACTCTTCCATCAAGCGGATCATAAAGAAGCTTCAGAACCTGGACCGGGCAG  2206
Neolamprologus brichardi         CCGTCAATACGACACCACTGACTTCCAAGATGGCTCGGTCAAACGCATCATTAAAAAACTCAGTGACATCGAGAGGGCTG  2164
Oncorhynchus mykiss              CCGCAAGTATGACACCACAGACTTCCAGGATGGGGCCTTGAAGCGTATCTTGAATAAACTCAGTGACATTGAAAGGGCCG  2161
Oreochromis niloticus            CCGTCAGTACGACACCACTGACTTCCAAGATGGCTCGGTCAAACGCATCATTAAAAAACTCAGTGACATCGAGAGGGCTG  2164
Poecilia formosa                 TCGCCAGTACGACACCTCCGACTTCCAGGACCCCTCGGTCAAACGCATCATCAAAAAACTCAGTGACCTTGAGAAGGCAG  2170
Poecilia latipinna               TCGCCAGTACGACACCTCCGACTTCCAGGACCCCTCGGTCAAACGCATCATCAAAAAACTCAGTGACCTTGAGAAGGCAG  2170
Pseudopodoces humilis            CAGGCAGTTTGACACCGCAGATTTCCAGGACCAAAGTGTCACCCGCATCCTCAAGAAGCTGAGTGTCATTGAGAGGGCAG  2146
Pundamilia nyererei              CCGTCAATACGACACCACTGACTTCCAAGATGGCTCGGTCAAACGCATCATTAAAAAACTCAGTGACATCGAGAGGGCTG  2164
Pygocentrus nattereri            CCGCAAATATGACACTACTGACTTCCAGGACCCTTCAGTAAAAAGGATTCTTAAGAAACTTAGTGATCTGGAAAGGGCTG  2146
Python bivittatus                TCGGCAGTTTGATTCCACTGATTTCAAAGATCCCAGCATAAAACGTATTCTGAAGAAATTAAGTGATATTGAGCGGGCAG  2158
Rattus norvegicus                CAAGACATTTGACGTGAGCAACTTCCAGAACTCTACCATCAAGCGGATCATAAAGAAGGTTCAGAACGTGGACCGGGCAG  2209
Scleropages formosus             ACAGAAGTTTGACACAACAGACTTTCAGGATCCATCTGTGAAACGAATCCTGAAAAAGCTTAGCGACATTGAAAGGGCAA  2149
Sinocyclocheilus anshuiensis     CCGTAAGATCGACACCAGTGACTTCCAGGATGAGTCAGTGAAGAGAATCTTGACGAAACTGAGTGACCTTGAGAGAGCTG  2146
Xenopus tropicalis               AAGAAACTACGATTACAGTGATTTCCAGGACCCTGAGACACAGCGCATTCTGAGGAAACTCAGTGAGATCGATAAAGCTG  2155
Lipotes vexillifer               CAGGAAGTTTGACGTGGCCAACTTCCAGAACGCCACTATGAAGCGGATGATAAAGAAGATTCAGGATCTAGAGCGGGCGG  2215
Orcinus orca                     CAGGAAGTTTGACGTGACCAACTTCCAGAACGCCACCATGAAGCGGATGATAAAGAAGATTCAGGATCTAGAGCGGGCGG  2200
Homo sapiens                     CAGGAAGTTTGATGTGAACCAGTTGCAGAACACCACTATCAAGCGGATCATAAAGAAGGTTCAGGACCTAGAACGGGCAG  2191
```

```
                                 2180      2190      2200      2210      2220      2230      2240      2250
conservation
Callorhinchus milii              CTCTTCCAGAACCAGAATTGATAGAGTATAACAATATTCTATCTGACATGGAAACCACCTACAGTGTTGCCAAAGTGTGT  2250
Alligator mississippiensis       CATTGCCCAAGGATGAGCTGGAGGAGTACAACAAACTTCTCTCGGACATGGAGACCACATACAGCATAGCCAAGGTCTGC  2235
Anolis carolinensis              CCCTATCTGAACAGGAGCTGAAGGAGTATAATCAGCTCCTCTCAGATATGGAAACAACTTACAGCGTAGCCAAAGTCTGT  2307
Astyanax mexicanus               TGCTCTCTGATGATGACCTGAAGGAGTACAACAACCTGTTGGCCTCCATGGAGACGACTTACAGTGTAGCTAAAGTCTGT  2235
Boleophthalmus pectinirostris    CGTTGTCTTCTGGAGAGCTGGAGGAGTACAACAATCTGCTGTCCAACATGGAGACCAAGTACAGTGTGGCAGAGGTGTGC  2244
Cynoglossus semilaevis           CACTGAGCCCTGCTGAGCTGGAAGATTACAATACTCTCCTGTCTGACATGGAGACCAAGTACAGTGTGGCTGAGGTCTGC  2247
Danio rerio                      CGCTTTCTGCAGAAGATCTCGTGGAGTACAATAATTTACTAGCATCCATGGAGACCCTGTACAGTGTAGCGACGGTCTGC  2355
Esox lucius                      GACTTCCAGACAATGAACTTGTAGAGTACAACAATCTGCTGGCTAATATGGAGACCAAGTACAGTGTGGCTAATGTGTGC  2241
Fundulus heteroclitus            CACTGTCCACTCCAGAGCTAGAAGAGTTCAACACTCTTCTGTCTAACATGGAGACCAAATACAGCGTTGCTGAGGTTTGC  2253
Gekko japonicus                  CCCTGTCTGAGGAGGAGCTGAAGGAGTATAATCAGCTCCTCTCAGACATGGAGACGACTTACAGTGTAGCCAAAGTCTGC  2247
Haplochromis burtoni             CGCTCCCCTCTGCAGAACTGGAAGAGTACAATACGCTCCTGTCCAACATGGAGACCAAGTACAGCGTGGCTGAGGTCTGC  2244
Hippocampus comes                CGCTGCCGTCGGCGCAGCTTGAGGAGTACAACAATCTCCTGTCCAACATGGAGACCAAATATAGCGTAGCAGAAGTGTGC  2229
Ictalurus punctatus              CGCTCCCTGACGCTCAGCTGAGAGAGTACAATGACTTGTTGGCCTCCATGGAGACCACATACAGTGTAGCTAAAGTCTGT  2244
Larimichthys crocea              CACTATCCTCAGAAGAACTGAAAGAGTACAACAATCTCCTATCCAACATGGAGACCGAGTACAGTGTGGCTCAGGTCTGC  2238
Lates calcarifer                 CACTGTCCCCGGCAGAGCTGGAAGAGTACAATACTCTGCTGTCCAACATGGAGACCAAGTACAGTGTGGCTGAGGTCTGC  2241
Latimeria chalumnae              CACTGCCTGAGGAGGAATTAAAAGAGTACAATAATATCCTGTCACAAATGGAAACAACATACAGTGTTGCCAAAGTTTGC  2229
Lepisosteus oculatus             GACTTCCACAGAATGAGCTCAAAGATTACAATGAGCTACTCTCTAGAATGGAGACCACTTACAGCGTTGCTACGGTGTGC  2229
Macaca fascicularis              CGCTGCCTGCCCAGGAGCTGGAGGAGTACAACAAGATCCTGTTGGACATGGAAACCACCTACAGCGTGGCCACTGTGTGC  2274
Manacus vitellinus               CCCTGCCTGAGGATGAGCTGATGGAGTATAACACCCTCCTCTCAGATATGGAGACCACGTACAGCCTGGCCAAGGTCTGC  2226
Mus musculus                     TGCTGCCTCCCAAGGAATTAGAAGAGTACAACCAGATCCTGCTAGACATGGAGACAACTTACAGCTTATCCAACATTTGC  2286
Neolamprologus brichardi         CGCTCCCCTCTGCAGAACTGGAAGAGTACAATACGCTCCTGTCCAACATGGAGACCAAGTACAGCGTGGCTGAGGTCTGC  2244
Oncorhynchus mykiss              GACTTCCCGACGAGGAACTGAAAGAGTACAACAATTTGCTGGCCAACATGGACACCAAGTACAGTGTGGCTGAGGTGTGC  2241
Oreochromis niloticus            CGCTCCCCTCAGCAGAACTGGAAGAGTACAATACACTCCTGTCCAACATGGAGACCAAGTACAGTGTGGCTGAGGTCTGC  2244
Poecilia formosa                 CGCTGCCTACTGAAGAACTGGAAGAGTTGAATAATATTATGTCTAGCATGGAAACCAAATATAGTATGGCAGAAGTTTGT  2250
Poecilia latipinna               CGCTGCCTACTGAAGAACTGGAAGAGTTAAATAATATTATGTCAAGCATGGAAACCAAATATAGTGTGGCAGAAGTTTGT  2250
Pseudopodoces humilis            CCCTACCTGAGAATGAGCTGAAGGAGTATAACACCCTTCTCTCAGATATGGAGACCACATACAGCGTGGCCAAGGTCTGC  2226
Pundamilia nyererei              CGCTCCCCTCTGCAGAACTGGAAGAGTACAATACGCTCCTGTCCAACATGGAGACCAAGTACAGCGTGGCTGAGGTCTGC  2244
Pygocentrus nattereri            TGCTGCCTGATGCTGAACTCAAGGAGTACAATAACTTGTTAGCCTCTATGGAGACTACATACAGCGTAGCTAAAGTCTGT  2226
Python bivittatus                CCTTACCTGAAGCAGAGCTGAAGGAGTATAATCAACTGCTGTCAGAAATGGAGACAATTTATAGTGTAGCCAAAGTCTGC  2238
Rattus norvegicus                TGCTGCCTCCCAACGAGTTAGAAGAGTACAACCAGATCCTGCTAGACATGGAGACGACTTACAGTGTAGCCAATGTTTGC  2289
Scleropages formosus             GACTTCCAGAGGAGGAATTAAAGGAGTACAACAATCTACTCGCCACCATGGAGACAAAATACAGTGTAGCACAAGCTTGC  2229
Sinocyclocheilus anshuiensis     CGCTTCCTGAAGAAGATCTCATAGAGTACAATAATTTGCTAGCATCCATGGAGACCCTGTACAGCGTAGCCACAGTCTGC  2226
Xenopus tropicalis               CTCTTTCAGAGGAGGAACAGAAAGAGTACAATCAAATCACATCCGACATGGAGACTATTTATAGTGTGGCCAAAGTTTGC  2235
Lipotes vexillifer               CGCTGCCTGTCAGGGAGCTGGAGCAGTACAACCAGATCCTGCTGGACATGGAGACCACTTACAGCGTGGCCTCTGTGTGC  2295
Orcinus orca                     CGCTGCCTGTCAGGGAGCTGGAGCAGTACAACCAGATCCTGCTGGACATGGAGACCACTTACAGCGTGGCCTCTGTGTGC  2280
Homo sapiens                     CACTGCCTGCCCAGGAGCTGGAGGAGTACAACAAGATCCTGTTGGATATGGAAACCACCTACAGCGTGGCCACTGTGTGC  2271
```

conservation	2260 · 2270 · 2280 · 2290 · 2300 · 2310 · 2320	
Callorhinchus milii	CGAGCC...AATGGGACATGTCACCCTCTGGACCCTGATCTGACGGAAATAATGGCATCCTCATCTAACTACAGTGAACT	2327
Alligator mississippiensis	AAGGAA...AACGACAAGTGCTATCCACTGGATCCTGACCTTACCAACATCCTCGCCACCTCAAAGGATTATGATGAGCT	2312
Anolis carolinensis	ACAGGA...GAGAAAGTCTGCAAGTCACTTGATCCTGATCTTACAAAAACCATGGCCCAATCAAGGGACTATGATGAACT	2384
Astyanax mexicanus	AAAGAT...GATGGAACCTGTCTACCTCTTGATCCAGACCTGAATAAGATCATGGCTGAGTCCAGAGACTATGATGAGCT	2312
Boleophthalmus pectinirostris	AGAGCC...AATAATACCTGCCATCCTTTGGACCCAGATCTTCAGAAGATCATGGCCGAGTCCAGGGACTACGATGAGCT	2321
Cynoglossus semilaevis	AGAGCC...AACGGCACCTGTCACCCACTGGATCCAGACCTACAGAAGATCATGGCGGAGTCCAGAGATTATGACGAGCT	2324
Danio rerio	AAGGAC...AAATCAAACTGTCTGCCTCTGGATCCAGATCTGAATAAGATCATGGCTGAGTCGCGGGATTATGACGAGCT	2432
Esox lucius	AGAGAC...AATGGGACCTGCCATCCACTGGACCCAGACCTCCAGAAGATCATGGCTGAGTCAAGAGACTACAGTGAGCT	2318
Fundulus heteroclitus	AGGGAA...GATGGCACGTGTCATCCGCTGGATCCAGATCTCCAGAAAATCATGGCCGAGTCGAGGGATTATAATGAACT	2330
Gekko japonicus	AATGGA...GAAAAAGGCTGCAAGGCCCTGGATCCAGAACTTACAGACATCATGGCCCAGTCAAGAGACTACGACGAACT	2324
Haplochromis burtoni	AGAGAG...AATAATGAATGCCTCCCTCTGGATCCAGATCTCCAGAAGATCATGGCAGAGTCCAGGGATTACGATGAGCT	2321
Hippocampus comes	AGAGAT...AATGGCCAATGCCACCCACTGGATCCCGACCTTCAGAAGATCATGGCGGAGTCCAGAGATTATGATGAGCT	2306
Ictalurus punctatus	GAAGAT...CATGGTACCTGTCTACCTCTTGATCCAGACCTAAATAAGATCATGGCTGAGTCCAGGGACTACGACAGGCT	2321
Larimichthys crocea	AGACCT...GACAAGACGTGCCATCCACTGGATCCAGATCTCCAGAAGATCATGGCAGAGTCCAGGGATTATGATGAATT	2315
Lates calcarifer	AGAGAT...GATGGGACTTGCCACCCACTGGATCCAGATCTCCAGAAGATAATGGCAGAGTCCAGGGATTATGATGAACT	2318
Latimeria chalumnae	CGAGAT...GATGGAAAATGCCATCCATTAGATCCAGATCTAACTGAGATTATGGCCACTTCAAGGAATTACAGTGAGCT	2306
Lepisosteus oculatus	CAAGAG...GGTCGGCGATGCCTCCCCCTGGACCCTGATCTCACTGAGATTATGGCCAAGTCCCGAGACTACAAGGAACT	2306
Macaca fascicularis	CACACG...AATGGCAGCTGCCTGCAGCTCGAGCCAGATCTGACGAATGTGATGGCCACGTCCCGGAAATATGAAGAACT	2351
Manacus vitellinus	AGGGAT...GACAAAACCTGTCTCCCACTGGATCCTGACCTCACAGACATCATGGCCACCTCCCGGGACTATGACGAGCT	2303
Mus musculus	TACACA...AATGGCACTTGTATGCCCCTGGAACCTGATCTAACAAACATGATGGCCACATCCCGGAAATATGAAGAATT	2363
Neolamprologus brichardi	AGAGAG...AATAATGAATGCCTCCCTCTGGATCCAGATCTCCAGAAGATCATGGCAGAGTCCAGGGATTACGATGAGCT	2321
Oncorhynchus mykiss	AGAGCC...AATGGGACCTGCCATCCACTGGACCCAGACCTCCAGAAGATCATGGCTGAGTCCAGAGACTACGATGAGCT	2318
Oreochromis niloticus	AGAGAG...AATAATGAATGCCTCCCTCTGGATCCAGATCTCCAGAAGATCATGGCAGAGTCCAGGGATTACGATGAGCT	2321
Poecilia formosa	AGGGCA...GATGGCAAATGTCATCCACTGGATCCAGATCTTCAGAAAATCATGGCAGAGTCCAGGGATTATGATGAACT	2327
Poecilia latipinna	AGGGCA...GATGGCAAATGTCATCCACTGGATCCAGATCTTCAGAAAATCATGGCAGAGTCCAGGGATTATGATGAACT	2327
Pseudopodoces humilis	AGAGAG...AACAAAACCTGTCACCCATTGGATCCTGACCTCACAGACATCATGGCCACCTCTCGGGACTATGATGAGCT	2303
Pundamilia nyererei	AGAGAG...AATAATGAATGCCTCCCTCTGGATCCAGATCTCCAGAAGATCATGGCAGAGTCCAGGGATTACGATGAGCT	2321
Pygocentrus nattereri	AGAGAT...GAAAATACCTGTCTACCACTTGATCCAGACCTGAATAAGATCATGGCTGAGTCCAGAGACTATGATGAGTT	2303
Python bivittatus	AGAAAA...GACACAGACTGCAGAGCACTAGATCCCGACCTTACCGATACCATGGCTACATCAAGAGACTACGATGAGCT	2315
Rattus norvegicus	TACACA...AATGGCACTTGTCTGTCACTGGAGCCTGATCTGACAAATATAATGGCCACGTCCCGGAAATACGAAGAATT	2366
Scleropages formosus	AGGGAT...AACGGAGAGTGTCTCCCCTTGGAACCAGACCTCACCAAGATCATGGCTGAAATCAGAGACTACGATGAGCT	2306
Sinocyclocheilus anshuiensis	AAGGAT...GAATCAAAGTGTCTACCTTTAGATCCAAATTTGAATAAGATCATGGCCGAGTCCAGGGATTATGACGAGCT	2303
Xenopus tropicalis	AAGGATGGTAACACAAACTGCCATCCTCTTGATCCAGACCTAACAGAGATCTTGGCCAAGTCTCGGGACTATGATGAGCT	2315
Lipotes vexillifer	CATAGC...AATGGCACTTGTCTACAGCTCGAGCCCGATCTGACAAATCTGATGGCCACATCCCGGAACTATGGAGAACT	2372
Orcinus orca	CATAGC...AACGGCACTTGTCTGCAGCTCGAGCCTGATCTGACACATCTGATGGCCACATCCCGGAACTATGAAGAACT	2357
Homo sapiens	CACCCG...AATGGCAGCTGCCTGCAGCTCGAGCCAGATCTGACGAATGTGATGGCCACGTCCCGGAAATATGAAGACCT	2348

conservation	2330 · 2340 · 2350 · 2360 · 2370 · 2380 · 2390 · 2400	
Callorhinchus milii	GCTGTTTGCCTGGAATGGGTGGAGAAATGTTTCTGGAAGAAAATGAGGAACAACTTCAAGAGATACGTGGAACTCAGCA	2407
Alligator mississippiensis	CCTTTTTGCCTGGAAGGGCTGGCGGGATGCCTCTGGGAATCAAATCAGAACCAAATACAAGAGATATGTGGAGCTAAGCA	2392
Anolis carolinensis	CCTAGAAGCATGGAAAGGCTGGCGGGATGTATCAGGCAAGAAAATCCGGACCCCTTACAAGAGATATGTGCAACTTAGCA	2464
Astyanax mexicanus	GCTGTTTGCCTGGCAGGGCTGGCGCAACGCTTCAGGAAGAGAACTGCGCCAAAGCTATGTGAGATACGTGGAGCTCGCCA	2392
Boleophthalmus pectinirostris	GCTGTTTGCTTGGAAAGGATGGAGAGATGCTGCAGGCAAAGTCCTCCGTCAGGATTACAAGAGATATGTGGAGCTGGCCA	2401
Cynoglossus semilaevis	GTTATTTGCCTGGAAAGGATGGAGAGACGCTGCTGGGAAAGTCCTTCGTCAGGATTATAAGAGATATGTGGAACTGGCCA	2404
Danio rerio	GCTGTTTGCCTGGCAGGGTTGGCGAAACGCCTCTGGCAGAGAAATCCGAGACAGCTACAAGAGATATGTGGAGCTCGCCA	2512
Esox lucius	TCTATTTGCCTGGCAGGGCTGGAGAAATGCCTCTGGCAGAGAACTTCGCCAGGACTACAAGAGATATGTGCAACTGGCCA	2398
Fundulus heteroclitus	TTTGTTTGCCTGGAAAGGATGGAGAGACGCTGCAGGCAAAGTGCTTCGTGAGGATTATAAGAGATATGTTGAACTGGCCA	2410
Gekko japonicus	CCTTAACGCCTGGAAAGACTGGCGGGACGTGTCAGGGAAAAAAATCCGCAGTCAGTACAAGAGATATGTCAGCCTGAGCA	2404
Haplochromis burtoni	GCTATTTGCCTGGAAAGGATGGCGAGATGCTGCTGGCAAAGTACTCCGTCAGGATTATAAGAGATATGTTGAACTGGCCA	2401
Hippocampus comes	GTTATTTGCCTGGAAGGGCTGGCGAGATGCAGCCGGCAAAGTACTTCGCTCGGATTACGAGCGATATGTGGAACTGGCCA	2386
Ictalurus punctatus	GCTGTTTGCCTGGCAGGGCTGGCGAAATGCTTCAGGAAGAGAACTGCGCCAAAGCTACAAGAGATATGTAGAGCTTGCCA	2401
Larimichthys crocea	GTTGTTTGCCTGGAAAGGATGGAGAGATGCTGCTGGCAAAGTGATTCGCCAAGATTACAAGAGATATGTTGAACTGGCCA	2395
Lates calcarifer	GTTATTTGCGTGGAAGGGATGGAGAGATTCTGCTGGAAAAGTACTTCGTCAGGATTACAAGAGATATGTTGAACTGGCCA	2398
Latimeria chalumnae	GCTCTTTGCTTGGAAAGGTTGGCATGATGCTTCAGGCAAGAAAATCCGTAAAGACTATGAGAAATATGTGGAATTTAGCA	2386
Lepisosteus oculatus	CCTTTTCGCCTGGCAGGGATGGCGTGATGCCTCTGGGAAAAAGATTCGCCAGGATTACAAGACCTACGTGGAGCTCAGCA	2386
Macaca fascicularis	GTTGTGGGCATGGGAGGGCTGGCGAGACAAGGCTGGGAGAGCCATCCTCCAGTTTTACCCAAAATACGTGGAACTGATCA	2431
Manacus vitellinus	CCTCTTTGCCTGGAAGGGCTGGCGGGATGCTTCTGGGAAGAAGATGAGGAACGACTACAAGCGATACGTGGAACTGAGCA	2383
Mus musculus	GCTATGGGCATGGAAGAGCTGGAGAGACAAGGTGGGGAGAGCCATCCTTCCTTTTTTCCCAAAGTATGTGGAGTTCTCCA	2443
Neolamprologus brichardi	GCTATTTGCCTGGAAAGGATGGCGAGATGCTGCTGGCAAAGTACTCCGTCAGGATTATAAGAGATATGTTGAACTGGCCA	2401
Oncorhynchus mykiss	GTTGTTCGCCTGGCAGGGCTGGAGAAATGCCTCTGGCAGAGAACTGCGACAGGACTACAAGAGATATGTGCAACTGGCCA	2398
Oreochromis niloticus	GCTATTTGCCTGGAAAGGATGGAGAGATGCTGCTGGCAAAGTACTCCGTCAGGATTATAAGAGATATGTTGAACTGGCCA	2401
Poecilia formosa	GTTGTTTGCCTGGAAAGGATGGAGAGATGCTGCTGGCAAAGTAATTCGTGACGATTATAAGAGATATGTTGAACTGGTCA	2407
Poecilia latipinna	GTTGTTTGCCTGGAAAGGATGGAGAGACGCTGCTGGCAAAGTAATTCGTGACGATTATAAGAGATATGTTGAACTGGTCA	2407
Pseudopodoces humilis	CCTCTTTGCCTGGAAGGGCTGGCGGGATGCTTCTGGGAAGAAGATGAGGAACAACTACAAGCGATACGTGGAACTGAGCA	2383
Pundamilia nyererei	GCTATTTGCCTGGAAAGGATGGCGAGATGCTGCTGGCAAAGTACTCCGTCAGGATTATAAGAGATATGTTGAACTGGCCA	2401
Pygocentrus nattereri	ACTGTTTGCCTGGCAAGGCTGGCGCAATGCTTCAGGAAGAGAACTGCGCCAGAGCTATGAGAGATATGTAGAGCTCGCCA	2383
Python bivittatus	TCTTAACACCTGGAAAGGCTGGCGGGATGCATCAGGGAAGAAAATCCGAAGCCACTACAAGAGATATGTAGAGCTAAGCA	2395
Rattus norvegicus	GCTTTGGGTGTGGAAGAGCTGGCGAGACAAGGTGGGGAGAGCCATCCTTCCCTTTTTCCCAAAGTACGTGGACTTCTCCA	2446
Scleropages formosus	CCTGTTTGCCTGGCAGGGCTGGCGCAATGCTTCTGGCAAGGGTCTTCGACAAGACTACAAGAGATACGTGGAGCTGGCCA	2386
Sinocyclocheilus anshuiensis	GCTGTTTGCCTGGCAGGGTTGGCGCAATGCCTCGGGCAGAGAGCTCCACAACAGCTACAAGAGATATGTGGAGCTTGCCA	2383
Xenopus tropicalis	TCTGTTTGCTTGGAAAGGGTGGAGAGATGCTTCAGGCAAACAAATCCGTGAAAAATACAAGAGATATGTACAATTGGCCA	2395
Lipotes vexillifer	GCTGTGGGCATGGAAGGGCTGGCGAGACAAGGTGGGGAGATCCATCCTCCCATATTTCCCCCAATATGTGGAGCTCACCA	2452
Orcinus orca	GCTGTGGGCATGGAAGGGCTGGCGAGACAAGGTGGGGAGATCCATCCTCCCATATTTCCCCCAATATGTGGAGCTCAGCA	2437
Homo sapiens	GTTATGGGCATGGGAGGGCTGGCGAGACAAGGCGGGGAGAGCCATCCTCCAGTTTTACCCGAAATACGTGGAACTCATCA	2428

conservation	2410 · 2420 · 2430 · 2440 · 2450 · 2460 · 2470 · 2480	
Callorhinchus milii	ACAAGGCGGCACGATTAAATAATCACGCAGATGCTGGAGCTTTCTGGAGGTCGCTGTATGATTCTGAAACATTTGAGGCA	2487
Alligator mississippiensis	ACAAGGCAGCCAGACTCAATGGTCATGCTGACAATGGTGCCTTCTGGAGATCCCTCTATGAAACACCCACCTTTGAAGAG	2472
Anolis carolinensis	ATAAGGCTGCCAGACTAAATGGTCATGCAGACAATGGTGCTTTCTGGAGATCACTATATGAAACACCAGCTTTTGAAGAA	2544
Astyanax mexicanus	ATTTGGCTGCCCGCAGTAACGGTCATGCTGACAATGGGGCTTTCTGGCGTTCCTTATATGAAACCCCCACCTTTGAGGAG	2472
Boleophthalmus pectinirostris	ACAAGGCTGCCACACTCAATGGTCACAGTGATAACGGGGCATTCTGGCGCTCTCTCTATGAAACTCCCACTTTTGAGCAA	2481
Cynoglossus semilaevis	ATAAAGCTGCCAAACTCAACGGTCACCCTGATAACGGAGCTTTCTGGCGTTCCTTGTATGAATCCCCAACCTTTGAGCAA	2484
Danio rerio	ACTCCGCTGCTAAAAGCAATGGTCACACTGATAACGGAGCATTCTGGCGCTCTCTGTATGAGACTCCCACCTTTGAACAG	2592
Esox lucius	ATAAAGCTGCCACACTTAATGGACACTCTGACAATGGAGCATTTTGGCGCTCCCTGTATGAGACCCCCACCTTTGAAGAG	2478
Fundulus heteroclitus	ATAAAGCTGCCACACTCAATGGTCATTCTGATAATGGGGCTTTCTGGCGGTCCCTGTACGAAACTCCTACCTTTGAGGAG	2490
Gekko japonicus	ATAAAGCTGCCACACTGAATGGGCATGCAGACAGCGGTGCTTTCTGGAGGTCTCTGTATGAATCACCCACATTTGAAGCG	2484
Haplochromis burtoni	ACAAAGCTGCCACACTAAATGGTCACTCTGATAATGGAGCTTTCTGGCGTTCCCTGTATGAAACGCCTACCTTTGAAGAA	2481
Hippocampus comes	ACCAAGCTGCCAGACTCAACGGTCACAGCGACAACGGTGCTTTCTGGCGCTCGCTGTACGAAACGCCGACCTTTGAGGAG	2466
Ictalurus punctatus	ACTTAGCTGCCAGGAGTAACGGTCACGCTGACAATGGCGCTTTCTGGCGCTCTTTGTACGAAACACCCACCTTCGAGCAG	2481
Larimichthys crocea	ACAAAGCTGCCACACTCAATGGACACTCTGACAATGGGGCTTTCTGGCGTTCCCTGTATGAAACCCCTACCTTTGAAGAA	2475
Lates calcarifer	ACAAAGCAGCAACACTCAATGGTCACCCTGATAATGGAGCTTTCTGGCGCTCCTTGTATGAGACCCCTACCTTTGAAGAA	2478
Latimeria chalumnae	ACAAGGCAGCAAGACTGAATGGCCATCCAGATAATGGAGCCTTTTGGCGATCCCTTTATGAGTCTCCAACTTTTGAAGCT	2466
Lepisosteus oculatus	ACAAAGCAGCCAGACTGAATGGTCACCCCGACAATGGGGCCTACTGGCGCTCGCTCTACGAGACGCCCACCTTCGAACAG	2466
Macaca fascicularis	ACCAGGCTGCCCGGCTCAATGGCTATGTAGATGCCGGGGACTCGTGGAGGTCTATGTACGAGACACCATCCCTGGAGCAA	2511
Manacus vitellinus	ACAAGGCAGCCGTGCTCAATGGTTACACAGACAATGGGGCCTACTGGAGATCCCTGTATGAGACATCCACCTTCGAGGAA	2463
Mus musculus	ACAAGATTGCCAAGCTCAATGGCTACACGGATGCAGGGGATTCATGGAGATCCTTATACGAGTCTGACAACCTGGAGCAA	2523
Neolamprologus brichardi	ACAAAGCTGCCACACTAAATGGTCACTCTGATAATGGAGCTTTCTGGCGTTCCCTGTATGAAATGCCTACCTTTGAAGAA	2481
Oncorhynchus mykiss	ACAAAGCTGCTGCGCTTAACGGACATTCTGACAATGGGGCGTTCTGGCGCTCCATGTACGAGACCCACTCCTTTGAAGAG	2478
Oreochromis niloticus	ACAAGGCTGCCACACTAAATGGTCACTCTGATAATGGAGCTTTCTGGCGTTCCCTGTATGAAACGCCTACCTTTGAAGAA	2481
Poecilia formosa	ATAAGGCTGCCACACTCAATGGGCATTCTGATAATGGGGCTTTCTGGCGGTCCCTGTATGAAACCCCAACCTTTGAAGAG	2487
Poecilia latipinna	ATAAGGCTGCCACACTCAATGGGCATTCTGATAATGGGGCTTTCTGGCGGTCCCTGTATGAAACCCCAACCTTTGAAGAG	2487
Pseudopodoces humilis	ACAAGGCAGCCATGCTCAATGGCTACAAAGACAATGGGGCCTACTGGAGATCCCTGTATGAGACATCCACCTTTGAGGAA	2463
Pundamilia nyererei	ACAAAGCTGCCACACTAAATGGTCACTCTGATAATGGAGCTTTCTGGCGTTCCCTGTATGAAACGCCTACCTTTGAAGAA	2481
Pygocentrus nattereri	ACTTGGCTGCCCGCAGTAACGGTCACATTGACAACGGGGCATTCTGGCGCTCCTTATACGAAACACCAACCTTTGAAAAG	2463
Python bivittatus	ATAAGGCTGCCAGATTAAATGGACATGCAGACAATGGTGCTTTCTGGAGATCCCTTTATGAAACTCCAACTTTCGAAACT	2475
Rattus norvegicus	ACAAGATCGCCAAGCTCAACGGCTACTCTGATGCAGGGGATTCCTGGAGATCCTCATATGAGTCCGACGACTTGGAGCAA	2526
Scleropages formosus	ACAAGGCTGCTAGACTTAATGGTCATGCTGACAATGGGGCATTCTGGCGCTCCCTGTATGAGACAACAACATTTGAAGAG	2466
Sinocyclocheilus anshuiensis	ACTTAGCTGCAAAGAGCAATGGTCACACGGATAATGGGGGATTTTGGCGTTCCCTGTATGAGACCCCCACCTTTGAAGAG	2463
Xenopus tropicalis	ACAAAGCTGCACAGCTCAATGGTTACAATGACAATGGAGCATACTGGAGATCTTGGTATGAGACCCCAACACTTGAATCA	2475
Lipotes vexillifer	ACAAGGCTGCCAGGCTCAATGGCTACGAGGACGGAGGGGACTCCTGGAGATCTATGTACGAGATGCCATTCCTGGAGTAT	2532
Orcinus orca	ACAAGGCTGCCAGGCTCAATGGCTACAAGGACGGAGGGGACTCCTGGAGATCTATGTACGAGATGCCATTCCTGGAGTAC	2517
Homo sapiens	ACCAGGCTGCCCGGCTCAATGGCTATGTAGATGCAGGGGACTCGTGGAGGTCTATGTACGAGACACCATCCCTGGAGCAA	2508

conservation	2490 · 2500 · 2510 · 2520 · 2530 · 2540 · 2550 · 2560	
Callorhinchus milii	GATTTGGAGAGGATTTACAACCAGCTCCAGCCTCTATACTTGAATCTGCATGCTTATGTCCGACGTGGATTGTATCAGAA	2567
Alligator mississippiensis	GACCTAGAGAAGCTCTACCTGCAACTGCAGCCCCTGTACCTCAACCTTCATGCCTATGTGCGCCGAGCCCTCTACAACAA	2552
Anolis carolinensis	GATCTAGAGAGGATCTGGCATCAGCTTCAGTCATTGTACTTCAATTTACATGCCTATGTACGTCGGGCTCTCTACAAAAA	2624
Astyanax mexicanus	GATCTGGAAGCATTATGGAAAGATCTACAGCCGCTCTACCTCAATCTTCACTCTTACGTCCGCCGTGCCCTCTTCAAAAA	2552
Boleophthalmus pectinirostris	GACCTGGAGGCGCTGTGGAAAGAGCTGGAGCCACTCTATCACAACGTGCACGCCTATGTCCGCCGCGCACTCTACAAGAA	2561
Cynoglossus semilaevis	GACCTGGAGACTCTGTGGTTGGAGCTGGAGCCACTCTACCAGAACGTCCACGCTTATGTCCGTAGAGCTTTGTACAAAAA	2564
Danio rerio	GATCTGGAGGCCTTGTGGAAAGATCTAGAACCGCTCTACCTCAGCGTTCATGCTTATGTGCGCAGGGCACTGTACAAGAA	2672
Esox lucius	GATCTTGAGTCTCTCTGGAAGGAGCTTGAACCCCTCTATCTCAATGTACACGCATATGTGCGCAGGTCCCTCTATAAGAA	2558
Fundulus heteroclitus	GACCTGGAGGCTCTGTGGAAGGAGCTGGAGCCACTCTACCTCAATGTGCATGCCTATGTTCGAAGGGCTCTTCACAAAAA	2570
Gekko japonicus	GACCTGGAGAGGATCTGGCAGCAACTTCAGCCGTTATACCTCAACTTGCACGCCTACGTGCGCAGGGCACTCCACAAGAC	2564
Haplochromis burtoni	GATCTGGAGACTCTGTGGAAAGAGCTGGAGCCACTCTATCTAAACTTGCATGCTTATGTTCGAAGGGGTCTATATAATAA	2561
Hippocampus comes	GACCTGGAGAGACTCTGGAAAGAACTTGAGCCGCTCTACCTGAACGTGCACTCCTATGTTCGCAGGGGTCTGTACAACAA	2546
Ictalurus punctatus	GATTTGGAGGCGTTATGGAAAGAGCTACAGCCGCTCTATCTCAACCTCCATGCGTATGTCCGGAGAGCACTCTTCAAAAA	2561
Larimichthys crocea	GACCTGGAGACTCTCTGGAAAGAGCTGGAGCCACTCTATCAGAATGTGCATGCATATGTTCGCAGAGCTTTGTACAAAAA	2555
Lates calcarifer	GACCTGGAGTCCCTGTGGAAGGAGCTGGAGCCGCTTTATCAGAACGTGCATGCTTATGTTCGTAGAGCTTTGTACAAAAA	2558
Latimeria chalumnae	GACCTGGAAAAACTCTATCATCAGCTACAGCCCCTGTACCTCAACCTCCATGCTTATGTCCGTCAGGCACTCCACAGAAA	2546
Lepisosteus oculatus	GACCTGGAGAGGCTGTACCAGGAGATGCAGCCCCTCTACCTCAACTTGCACGCCTATGTGCGCAAAGCCCTGTACAAGAA	2546
Macaca fascicularis	GACCTGGAGCGCCTCTTCCAGGAGCTGCAGCCGCTGTACCTGAACCTGCATGCCTATGTGCGCCGGGCCCTGCACCGCCA	2591
Manacus vitellinus	GATCTGGAGAGGCTGTACCAGCAGCTGCAGCCCCTGTACCTCAACCTGCACGCCTACGTGCGCCGGGCCCTCTACAAGAA	2543
Mus musculus	GACCTGGAAAAACTGTACCAGGAGCTGCAGCCACTCTACCTGAACCTGCATGCCTATGTGCGTCGTTCCCTGCACCGCCA	2603
Neolamprologus brichardi	GATCTGGAGACTCTGTGGAAGGAGCTGGAGCCACTCTATCTAAACTTGCATGCTTATGTTCGAAGGGGTCTATATAATAA	2561
Oncorhynchus mykiss	GATCTGGAGCATCTGTGGAAGGAGCTGGAGCCCCTCTATCTCAATGTGCACGCCTACGTGCGCAGGTCTCTTTACAGGAA	2558
Oreochromis niloticus	GATCTGGAGACTCTGTGGAAGGAGCTGGAGCCACTCTATCTGAACTTGCATGCTTATGTTCGAAGGGGTCTATATAATAA	2561
Poecilia formosa	GACCTGGAGGCTCTGTGGAAGGAACTGGAGCCGCTCTATCTCAACGTACACGCCTACGTTCGAAGGGCTCTTTACAAAAA	2567
Poecilia latipinna	GACCTGGAGGCTCTGTGGAAGGAACTGGAGCCGCTCTATCTCAACGTACACGCCTACGTTCGAAGGGCTCTTTACAAAAA	2567
Pseudopodoces humilis	GATCTGGAGAGGCTGTATCTGCAGCTGCAGCCCCTGTACCTCAACCTACATGCTTATGTACGCCGAGCCCTCTACAAAAA	2543
Pundamilia nyererei	GATCTGGAGACTCTGTGGAAGGAGCTGGAGCCACTCTATCTAAACTTGCATGCTTATGTTCGAAGGGGTCTATATAATAA	2561
Pygocentrus nattereri	GACCTGGAGGCGTTATGGAAAAATCTAGAGCCCCTCTACCTCAATCTGCACACTTATGTCCGCCGCGCTCTCTTTAAAAA	2543
Python bivittatus	GACCTGGAGAACATCTGGCATCAGCTTCAACCATTATACTTCAATCTGCATGCTTATGTACGTCGGGCTTTATACAAAAA	2555
Rattus norvegicus	GACCTGGAAAAACTATACCAGGAGCTGCAGCCGCTCTACCTGAACCTGCATGCCTATGTGCGCCGCTCCCTGCACCGCCA	2606
Scleropages formosus	GATCTTGAGAGACTGTGGAAGGAGCTTGAGCCACTCTATCTCAATGTGCATGCATATGTTCGCAGAGTGCTCTTTAAACA	2546
Sinocyclocheilus anshuiensis	GATCTAGAGGCCTTGTGGAAAGATCTAGAGCCGCTCTACCTCAACATTCATGCTTATGTGCGCAGGGCACTGTACAAGAA	2543
Xenopus tropicalis	GATGTTGAGAAACTTTATGACGAGTTGCAGCCTCTCTACCTCAATTTGCATGCATATGTGCGCAGGGCTCTTTACAAGAA	2555
Lipotes vexillifer	GACCTGGAGCAGCTCTTCCAGGAGTTGCAGCCGCTCTACCTGAACCTGCACGCCTACGTGCGCCGGGCCCTGTATCGCTT	2612
Orcinus orca	GAGCTGGAGCAGCTCTTCCAGGAGCTGCAGCCGCTCTACCTGAACCTGCACGCCTACGTGCGCCGGGCGCTGTATCGCTT	2597
Homo sapiens	GACCTGGAGCGGCTCTTCCAGGAGCTGCAGCCACTCTACCTCAACCTGCATGCCTACGTGCGCCGGGCCCTGCACCGTCA	2588

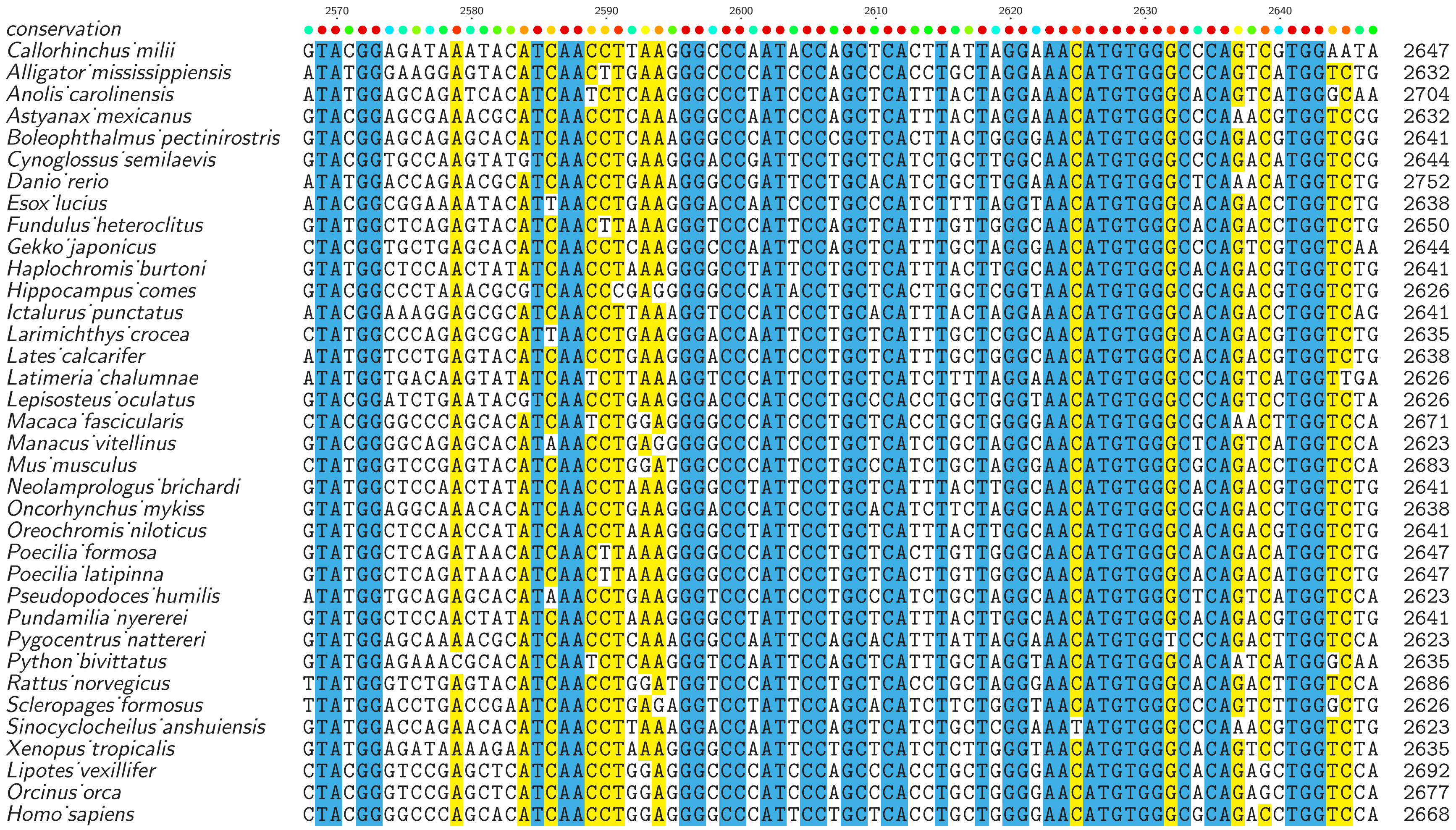

2570
2580
2590
2600
2610
2620
2630
2640
conservation
Callorhinchus milii 2647
Alligator mississippiensis 2632
Anolis carolinensis 2704
Astyanax mexicanus 2632
Boleophthalmus pectinirostris 2641
Cynoglossus semilaevis 2644
Danio rerio 2752
Esox lucius 2638
Fundulus heteroclitus 2650
Gekko japonicus 2644
Haplochromis burtoni 2641
Hippocampus comes 2626
Ictalurus punctatus 2641
Larimichthys crocea 2635
Lates calcarifer 2638
Latimeria chalumnae 2626
Lepisosteus oculatus 2626
Macaca fascicularis 2671
Manacus vitellinus 2623
Mus musculus 2683
Neolamprologus brichardi 2641
Oncorhynchus mykiss 2638
Oreochromis niloticus 2641
Poecilia formosa 2647
Poecilia latipinna 2647
Pseudopodoces humilis 2623
Pundamilia nyererei 2641
Pygocentrus nattereri 2623
Python bivittatus 2635
Rattus norvegicus 2686
Scleropages formosus 2626
Sinocyclocheilus anshuiensis 2623
Xenopus tropicalis 2635
Lipotes vexillifer 2692
Orcinus orca 2677
Homo sapiens 2668

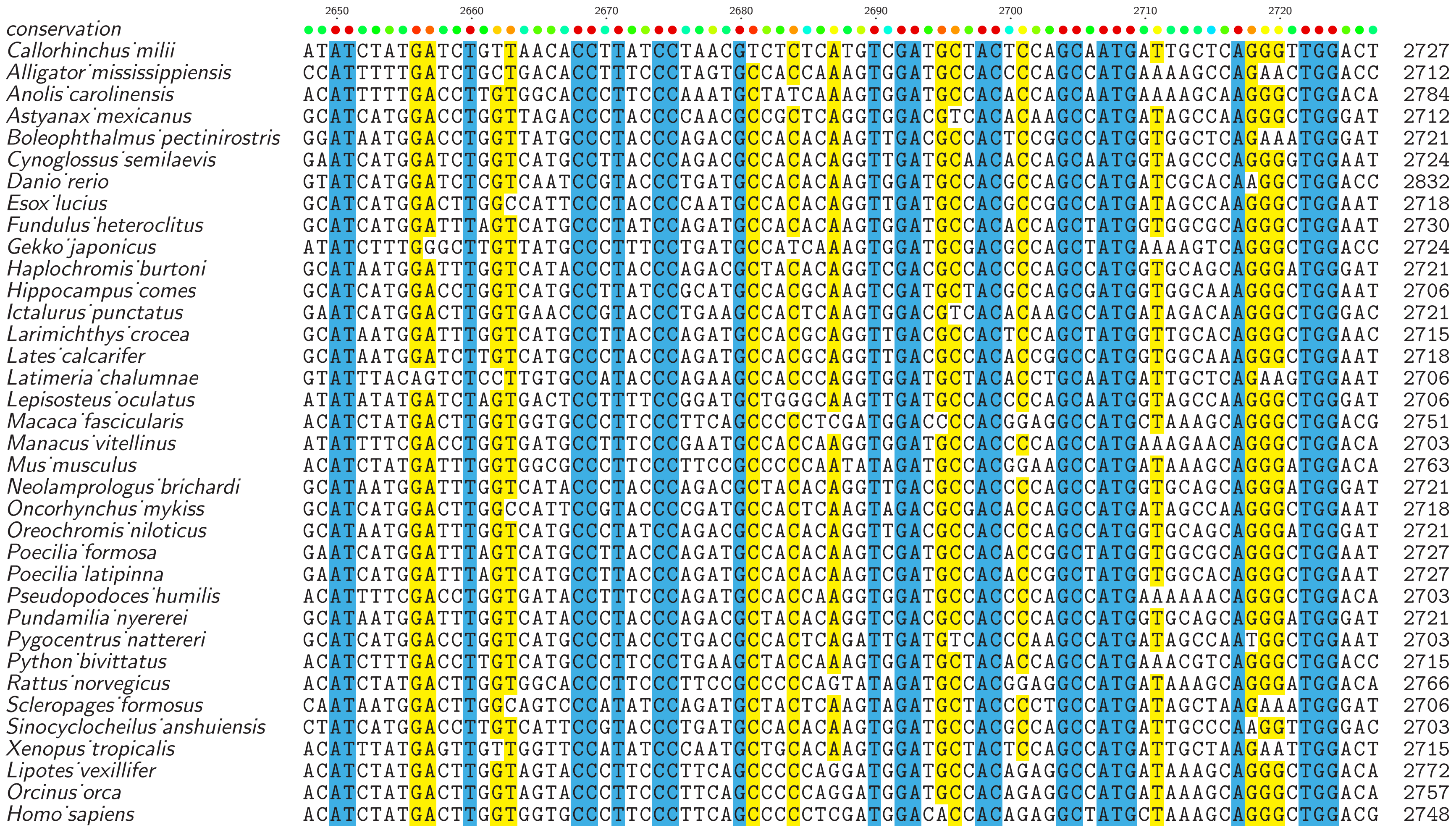

2650
2660
2670
2680
2690
2700
2710
2720
conservation
Callorhinchus milii 2727
Alligator mississippiensis 2712
Anolis carolinensis 2784
Astyanax mexicanus 2712
Boleophthalmus pectinirostris 2721
Cynoglossus semilaevis 2724
Danio rerio 2832
Esox lucius 2718
Fundulus heteroclitus 2730
Gekko japonicus 2724
Haplochromis burtoni 2721
Hippocampus comes 2706
Ictalurus punctatus 2721
Larimichthys crocea 2715
Lates calcarifer 2718
Latimeria chalumnae 2706
Lepisosteus oculatus 2706
Macaca fascicularis 2751
Manacus vitellinus 2703
Mus musculus 2763
Neolamprologus brichardi 2721
Oncorhynchus mykiss 2718
Oreochromis niloticus 2721
Poecilia formosa 2727
Poecilia latipinna 2727
Pseudopodoces humilis 2703
Pundamilia nyererei 2721
Pygocentrus nattereri 2703
Python bivittatus 2715
Rattus norvegicus 2766
Scleropages formosus 2706
Sinocyclocheilus anshuiensis 2703
Xenopus tropicalis 2715
Lipotes vexillifer 2772
Orcinus orca 2757
Homo sapiens 2748

```
                              2730      2740      2750      2760      2770      2780      2790      2800
conservation
Callorhinchus milii           CCCAAAAAGATGTTTGAAGAATCAGATAAGTTTTTTACATCTCTCGGGCTTATCCAAATGCCGAAGGAATTCTGGGATAA  2807
Alligator mississippiensis    CCCAAGAGGATGTTTCAGGAGTCAGACAATTTCTTCACCTCTCTGGACTTGATCCCTATGCCCCAGGAATTCTGGGACAA  2792
Anolis carolinensis           CCAAAGAAGATGTTTGAAGAGTCAGATAATTTTTTTAAATCACTGGGCCTCATCCCTATGCCTGATGAGTTCTGGAAATC  2864
Astyanax mexicanus            GCTAATCGAATGTTCAGGGAGTCTGAAAACTTCTTCACGTCTGTGGGACTGCTGCCCATGCCTGATGAGTTCTGGCAGAA  2792
Boleophthalmus pectinirostris GCTCATAGAATGTTTAAGGAATCAGAAAACTTCTTCACCTCTCTGGGCCTGATGGAGATGCCACCAGAGTTCTGGGACAA  2801
Cynoglossus semilaevis        GCTGTGAAAATGTTCAATGAGTCAGACCGGTTCTTTACTTCTCTTGGTTTGCTGCCAATGCCTCAAGAATTCTGGGATAA  2804
Danio rerio                   CCCAAGCGTATGTTTGAGGAATCTGACAGGTTTTTCACATCTCTGGGTCTCCTGCCCATGCCTCCTGAGTTCTGGAACAA  2912
Esox lucius                   GCCGTAAGGATGTTCAATGAGTCAGACCACTTCTTCACTTCTCTGGGGTTGCTGCCTATGCCTCCAGAGTTCTGGAGTAA  2798
Fundulus heteroclitus         GCCACCAGGATGTTTCAGGAATCAGACAGATTTTTCACCTCCCTGGGTCTGCTGCCAATGCCACAAGAGTTCTGGGACAA  2810
Gekko japonicus               CCGAAGAAGATGTTTGAGCAGTCGGACAGCTTCTTCCAATCCCTGGGCCTCATCCCTATGCCACCGGAGTTCTGGAACCG  2804
Haplochromis burtoni          GCCATCAGAATGTTTCAGGAATCTGACAAATTTTTCACCTCACTTGGTTTGGAGCCAATGCCGCCGGAATTCTGGAACAA  2801
Hippocampus comes             GCCAAAAGAATGTTCCAGGAATCAGATAACTTTTTCACCTCTCTGGGGCTGCTGCCAATGCCGAAAGAGTTCTGGGACAA  2786
Ictalurus punctatus           GCTCTGAAAATGTTCACTGAGTCGAACGACTTCTTCACGTCAGTGGGCCTGGAGCCTATGCCTTTGGAATTCTGGAACAA  2801
Larimichthys crocea           CCCATCAGAATGTTCCAGGAGTCAGACAGGTTTTTCACCTCTTTGGGTCTTCTGCCAATGCCACCAGAGTTTTGGAACAA  2795
Lates calcarifer              GCCACCAGGATGTTCCAGGAATCAGACAATTTTTTCACCTCTCTTGGTTTGCTACCAATGCCAAAAGAGTTTTGGGATAA  2798
Latimeria chalumnae           GCGGAAAGATGTTCAAGGAGTCTGACAACTTTTTACCTCCCTTGGACTCATCCCCAAGAATTCTGGGACAA  2786
Lepisosteus oculatus          GCACGGAAGATGTTTGAACAGTCAGACCACTTCTTCCAGTCCCTTGGATTGATTCCCATGCCCCCGGAGTTCTGGAGCAA  2786
Macaca fascicularis           CCCCGGAGGATGTTTAAGGAGGCTGATGATTTCTTCACCTCCCTGGGGCTGCTGCCCGTGCCTCCTGAGTTCTGGAACAA  2831
Manacus vitellinus            GCCAAGAAGATGTTTGAGGAGTCAGACCGTTTCTTTACCTCCCTGGGCCTCATCCCCATGCCCGAGGAGTTCTGGAACAA  2783
Mus musculus                  CCCAGAAGGATATTTAAGGAAGCTGACAATTTCTTTACCTCCCTGGGGCTGTTACCTGTGCCCCCTGAGTTCTGGAACAA  2843
Neolamprologus brichardi      GCCATCAGAATGTTTCAGGAATCTGACAAATTTTTCACCTCACTTGGTTTGGAGCCAATGCCGCCGGAATTCTGGAGCAA  2801
Oncorhynchus mykiss           GCCACCAGGATGTTCCAAGAGTCAGACAACTTCTTCACTTCTCTGGGGCTATTGCCCATGCCTCCAGAGTTCTGGGTCAA  2798
Oreochromis niloticus         GCCATCAGAATGTTTCAGGAATCTGACAAATTTTTCACCTCTCTGGGTTTGGAGCCAATGCCGCCGGAATTCTGGAGCAA  2801
Poecilia formosa              GCAACCAGGATGTTTCAGGAATCGGACAGGTTTTTCACCTCCCTGGGTCTACTGCCAATGCCACAAGAGTTCTGGGACAA  2807
Poecilia latipinna            GCAACCAGGATGTTTCAGGAATCGGACAGGTTTTTCACCTCCCTGGGTCTACTGCCAATGCCACAAGAGTTCTGGGACAA  2807
Pseudopodoces humilis         CCCAAAAAATGTTTGAAGAGTCAGACCGTTCTTCACCTCTCTGGGCCTCATCCCCATGCCACAGGAGTTCTGGGACAA  2783
Pundamilia nyererei           GCCATCAGAATGTTTCAGGAATCTGACAAATTTTTCACCTCACTTGGTTTGGAGCCAATGCCGCCGGAATTCTGGAACAA  2801
Pygocentrus nattereri         GCCCGGCGAATGTTCAATGAGTCTGAAAGATTCTTCACGTCTTTGGGCCTGATGGCCATGCCTGATGAGTTCTGGCAGAA  2783
Python bivittatus             CCAAAGAAGATGTTTGAACAGTCTGATAGTTTCTTCAGATCACTGGGACTTATCCCTATGCCACCTGAGTTCTGGAACAA  2795
Rattus norvegicus             CCCAGAAGGATATTTAAGGAAGCTGACAATTTTTTTACCTCCCTGGGGCTGTTACCTGTGCCCCCTGAGTTCTGGAACAA  2846
Scleropages formosus          CCCAGAAAGATGTTTAATGAGTCTGACAGATTCTTCACCTCTCTGGGTTTGATCCCTATGCCCCCTGAGTTCTGGGACAA  2786
Sinocyclocheilus anshuiensis  CCCAAACGGATGTTCGAGGAATCGGACCGGTTCTTCACTTCTCTGGGTCTTCTGCCCATGCCTCCTGAGTTCTGGAACAA  2783
Xenopus tropicalis            CCAAAAAGGATGTTTGAGGAATCTGACAACTTCTTCAAGTCTCTGGGGCTTCTCCCAATGCCACAGGAATTCTGGGATAA  2795
Lipotes vexillifer            CCCCAAAGGATGTTTAAGGAAGCAGACAATTTCTTCACCTCCCTGGGGCTGCTGCCCGTGCCCCCCGAGTTCTGGAGCAA  2852
Orcinus orca                  CCCCAAAGGATGTTTAAGGAAGCAGACAATTTCTTCATCTCCCTGGGGCTGCTGCCCGTGCCCCCCGAGTTCTGGAGCAA  2837
Homo sapiens                  CCCAGGAGGATGTTTAAGGAGGCTGATGATTTCTTCACCTCCCTGGGGCTGCTGCCCGTGCCTCCTGAGTTCTGGAACAA  2828
```

```
                              2810      2820                2830      2840      2850      2860      2870      2880
conservation
Callorhinchus milii           GTCGATGTTGATGAAACCT...GAAGGCAGGGAGGTTGTCTGTCATGCTTCTGCCTGGGACTTTTATAACAGGAAAGATT  2884
Alligator mississippiensis    GTCCATGATTGAGAAGCCATCGGATGGGCGGGAAGTGGTGTGCCATGCCTCAGCCTGGGACTTCTACAACCGCAAGGACT  2872
Anolis carolinensis           ATCCATGCTGGAAAAACCAAAAGATGGGAGGGAAGTGGTTTGCCATGCATCAGCATGGGACTTCTACAACCGCAAGGATT  2944
Astyanax mexicanus            GTCCATGCTGGAGAAACCCACAGACGGCCGAGAGGTGGTGTGCCATGCCTCTGCCTGGGACTTTTACAACCGCAAAGACT  2872
Boleophthalmus pectinirostris GTCAATGCTGGAGAAACCCTCCGAT...CGCAAAGTGGTGTGCCACGCATCAGCATGGGACTTCTACAACAGAAAGGACT  2878
Cynoglossus semilaevis        ATCCATGCTGGAAAAGCCTGAGGATGGGCGTAAGGTTGTGTGTCATGCCTCTGCCTGGGACTTCTACAACAGGAAAGACT  2884
Danio rerio                   GTCCATGCTGGAAAAGCCAACAGATGGCCGTGAAGTGGTGTGCCATGCCTCTGCCTGGGACTTCTACAACCGCAAAGACT  2992
Esox lucius                   ATCCATGCTGGAGAAACCCACGGATGGCCGCAATGTGGTGTGTCATGCCTCCGCCTGGGACTTTTACAACCGCAAAGACT  2878
Fundulus heteroclitus         GTCCATGCTGGAAAAGCCTTCTGACGGACGGCAGGTGGTGTGCCACGCGTCTGCATGGGATTTTTATAACCGAAAGGATT  2890
Gekko japonicus               GTCCATGCTGGAGAAGCCAGCGGATGGCAGGGAAGTGGTGTGCCACGCCTCAGCCTGGGACTTCTACAACCGCAAGGACT  2884
Haplochromis burtoni          ATCTATGCTGGAGAAGCCGTCTGATGGACGCCAGGTGGTGTGCCACGCCTCTGCGTGGGACTTCTATAACCGAAAAGACT  2881
Hippocampus comes             ATCCATGCTGGAGAAGCCCACCGACGGACGCCAAGTGGTCTGCCACGCTTCCGCATGGGACTTCTACAACAGAAAAGACT  2866
Ictalurus punctatus           GTCAATGCTGACGAAGCCTGCAGAC...CGTGAAGTGGTGTGTCATGCCTCAGCCTGGGACTTTTACAACCGCAAAGACT  2878
Larimichthys crocea           GTCGATGCTTGAGAAGCCGTCTGATGGGCGACAGGTGGTGTGCCACGCCTCTGCATGGGACTTCTATAACAGAAAAGACT  2875
Lates calcarifer              GTCCATGCTGGAGAAGCCGTCTGACGGGCGCCAGGTGGTGTGCCATGCCTCAGCATGGGACTTCTACAACCGAAAAGACT  2878
Latimeria chalumnae           ATCCATGATTGAGAAGCCAACAGATGGGAGGGAGGTTGTGTGCCATGCCTCTGCTTGGGACTTCTACAACAGGAAAGACT  2866
Lepisosteus oculatus          GTCCATGCTGGAGAAGCCCTCCGATGGGCGGTCGGTGGTATGCCACGCCTCGGCCTGGGACTTCTACAACCGCAAGGACT  2866
Macaca fascicularis           GTCGATGCTGGAGAAGCCAACTGACGGGCGAGAGGTGGTCTGCCACGCCTCGGCCTGGGACTTCTACAACGGCAAGGACT  2911
Manacus vitellinus            GTCCATGATCGAGAAGCCGTCGGACGGGCGGGAGGTGGTGTGTCACGCCTCGGCCTGGGACTTCTACAACCGCAAGGACT  2863
Mus musculus                  GTCGATGTTAGAGAAGCCCACCGATGGAAGGGAGGTGGTGTGCCATCCCTCAGCCTGGGACTTCTACAACGGCAAGGACT  2923
Neolamprologus brichardi      ATCTATGCTGGAGAAGCCGTCTGATGGACGCCAGGTGGTGTGCCACGCCTCTGCGTGGGACTTCTATAACCGAAAAGACT  2881
Oncorhynchus mykiss           GTCCATGCTAGAGAAACCCAATGATGGACGCAACGTGGTGTGCCATGCCTCCGCCTGGGACTTCTACAACCGCAAAGACG  2878
Oreochromis niloticus         ATCTATGCTGGAGAAGCCGTCTGATGGACGCCAGGTGGTGTGCCACGCCTCTGCGTGGGACTTCTATAACCGAAAAGACT  2881
Poecilia formosa              ATCCATGCTGGAGAAGCCGACTGACGGACGCCAGGTGGTGTGCCATGCCTCTGCGTGGGATTTCTTTAACCGAAAGGACT  2887
Poecilia latipinna            ATCCATGCTGGAGAAGCCGACTGACGGACGCCAGGTGGTGTGCCATGCCTCTGCGTGGGATTTCTTTAACCGAAAGGACT  2887
Pseudopodoces humilis         GTCCATGATTGAGAAGCCATCAGATGGGCGGGAGGTGGTGTGTCATGCCTCGGCCTGGGATTTCTACAATCGCAAGGACT  2863
Pundamilia nyererei           ATCTATGCTGGAGAAGCCGTCTGATGGACGCCAGGTGGTGTGCCACGCCTCTGCGTGGGACTTCTATAACCGAAAAGACT  2881
Pygocentrus nattereri         GTCCATGCTGGAGAAGCCCACAGAC...CGGGAGGTGGTATGTCATGCCTCGGCCTGGGACTTCTACAACCGCAAAGACT  2860
Python bivittatus             GTCTATGTTGGAAAAGCCATCAGATGGAGGGAAGTGGTCTGTCATGCTTCGGCCTGGGACTTCTACAACCGCAAGGACT  2875
Rattus norvegicus             GTCAATGTTAGAGAAGCCAACCGATGGAGGGAGGTGGTGTGCCATGCCTCAGCCTGGGACTTCTACAACGGCAAGGACT  2926
Scleropages formosus          ATCCATGTTGGAAAAGCCAACTGATGGCCGAAATGTGGTTTGCCATGCCTCCGCCTGGGACTTCTACAATCGTAAGGACT  2866
Sinocyclocheilus anshuiensis  GTCCATGCTGGAAAAGCCAACAGATGGACGTGAAGTGGTCTGTCACGCATCTGCTTGGGACTTCTACAACCGCAAAGACT  2863
Xenopus tropicalis            ATCCATGATCGAGAAGCCTAAAGAC...AGAGAGGTAGTGTGCCATGCATCTGCTTGGGACTTCTACAACCGCAAAGATT  2872
Lipotes vexillifer            GTCTATGCTGGAGAAGCCGGCCGACGGGCGGGAGGTGGTGTGCCACGCCTCCGCCTGGGACTTTTCAATGGCAAGGACT  2932
Orcinus orca                  GTCTATGCTGGAGAAGCCGACCGATGGGCGGGAGGTGGTGTGCCACGCCTCCGCCTGGGACTTCTTCAACGGCAAGGACT  2917
Homo sapiens                  GTCGATGCTGGAGAAGCCAACCGACGGGCGGGAGGTGGTCTGCCACGCCTCGGCCTGGGACTTCTACAACGGCAAGGACT  2908
```

	2890 2900 2910 2920 2930 2940 2950 2960	
conservation		
Callorhinchus milii	TTAGGATTAAGCAATGTACTGTGGTCAACATGGAAGATCTGGTCACTGTCCACCATGAAATGGGCCACGTTCAGTACTTC	2964
Alligator mississippiensis	TCAGGATTAAGCAGTGTACGGTGGTGAACATGGATGACCTGATCACTGTGCACCATGAGATGGGCCACGTCCAGTACTTC	2952
Anolis carolinensis	TCAGAATTAAGCAATGCACCGTTGTGAATATGGATGACTTAATAACAACTCACCATGAAATGGGGCATGTCCAGTACTTT	3024
Astyanax mexicanus	TCAGAATTAAGCAGTGCACTGTGATAAACATGGATGACCTCATCACCGTGCACCATGAGATGGGTCATGTTCAGTACTTC	2952
Boleophthalmus pectinirostris	TCAGAATCAAACAGTGCACAGTGATCACCATGGACGACCTGATCACAGTGCATCACGAGATGGGACATGTGCAATACTTT	2958
Cynoglossus semilaevis	TCAGGATCAAACAGTGCACAGTGATCACCATGGACGACCTGATCACCGTGCACCATGAGATGGGCCACGTCCAGTACTTC	2964
Danio rerio	TTAGGATCAAGCAGTGCACGGTGGTGACCATGGATGACCTCATTACTGTGCACCACGAGATGGGCCACGTTCAGTACTTC	3072
Esox lucius	TCAGGATCAAACAGTGCACTGTGGTAACTATGGATGATCTTATCACTGTGCACCATGAGATGGGTCACGTGCAGTATTTC	2958
Fundulus heteroclitus	TCAGGATAAAACAGTGCACTGTTGTGACCATGGATGACCTGATCACTGTGCACCATGAAATGGGTCACGTACAGTACTTC	2970
Gekko japonicus	TCAGGATTAAACAGTGCACTGTTGTGAATATGGATGATTTGATCACGGTCCACCATGAAATGGGCCACGTGCAGTACTTC	2964
Haplochromis burtoni	TCAGGATCAAGCAGTGCACTGTTGTCACAATGGATGACCTGATCACCGTGCACCACGAGATGGGCCACATTCAGTATTTC	2961
Hippocampus comes	TCAGAATCAAACAGTGCACCGTTGTGACCATGGACGACTTGATCACCGTCCACCACGAAATGGGCCACGTCCAGTACTTC	2946
Ictalurus punctatus	TCAGGATTAAGCAATGTACAGTGGTGGACATGGACGACCTGATCACAGTGCATCATGAAATGGGCCATGTGCAGTATTTC	2958
Larimichthys crocea	TCAGGATCAAACAGTGTACTGTTGTGACTATGGATGACCTGATCACAGTTCACCATGAGATGGGCCATGTGCAGTACTTC	2955
Lates calcarifer	TCAGGATCAAACAGTGCACTGTTGTGACTATGGATGACCTGATCACGGTGCACCACGAGATGGGCCACGTCCAGTACTTC	2958
Latimeria chalumnae	TCAGAATTAAGCAATGTACTGTCGTCAACATGGATGACCTCATCACTGTCCATCATGAGATGGGACATATTCAGTATTTC	2946
Lepisosteus oculatus	TCAGGATCAAGCAGTGCACAGTGGTGACGATGGATGACCTGATCACTGTACATCATGAGATGGGCCATGTGCAGTACTTC	2946
Macaca fascicularis	TCCGGATCAAGCAGTGCACCACTGTGAACTTGGAGGACCTGGTGGTGGCCCACCACGAAATGGGCCACATCCAGTATTTC	2991
Manacus vitellinus	TCAGGATCAAGCAGTGCACGGTGGTGAACATGGACGACCTGATCACGGTGCACCACGAGATGGGCCACGTCCAGTACTTC	2943
Mus musculus	TCAGGATCAAGCAGTGTACCTCTGTGAACATGGAGGACTTGGTGATAGCGCACCACGAAATGGGCCACATCCAGTATTTC	3003
Neolamprologus brichardi	TCAGGATCAAGCAGTGCACTGTTGTCACAATGGATGACCTGATCACCGTGCACCACGAGATGGGCCACATTCAGTATTTC	2961
Oncorhynchus mykiss	TCAGGATCAAACAGTGCACCGTGATAACCATGGATGACCTCATCACAGTGCACCATGAGATGGGTCATGTGCAGTACTTC	2958
Oreochromis niloticus	TCAGGATCAAGCAGTGCACTGTTGTCACTATGGATGACCTGATCACCGTGCACCACGAGATGGGCCACATTCAGTATTTC	2961
Poecilia formosa	TCAGGATAAAACAGTGCACCGTTGTGACTATGGATGACCTGATCACTGTGCACCATGAGATGGGCCATGTGCAGTACTTC	2967
Poecilia latipinna	TCAGGATAAAACAGTGCACTGTTGTGACTATGGATGACCTGATCACTGTGCACCATGAGATGGGCCATGTGCAGTACTTC	2967
Pseudopodoces humilis	TCAGGATCAAGCAGTGCACCGTGGTGAACATGGACGACCTCATCACAGTTCATCACGAGATGGGCCACGTGCAGTACTTC	2943
Pundamilia nyererei	TCAGGATCAAGCAGTGCACTGTTGTCACAATGGATGACCTGATCACCGTGCACCACGAGATGGGCCACATTCAGTATTTC	2961
Pygocentrus nattereri	TCAGGATCAAGCAGTGCACAGTGGTAAACATGGATGACCTCATCACAGTGCACCATGAGATGGGCCATGTGCAGTACTTC	2940
Python bivittatus	TTAGGATAAAACAATGCACTGTTGTGAATATGGATGATTTAATAACAGTTCACCATGAAATGGGACACGTCCAATACTTT	2955
Rattus norvegicus	TCAGGATCAAGCAGTGTACCTCTGTGAACATGGAGGAATTGGTGATAGCCCACCACGAAATGGGCCACATCCAGTATTTC	3006
Scleropages formosus	TCAGGGTCAAACAGTGCACAGTGGTGACCATGGATGATCTCATTACGGTTCACCACGAGATGGGTCATGTACAGTACTTC	2946
Sinocyclocheilus anshuiensis	TCAGGATCAAGCAGTGCACAGTGGTGACCATGGACGACCTCATCACTGTGCACCATGAGATGGGCCACGTTCAGTACTTC	2943
Xenopus tropicalis	TCAGGATTAAACAGTGCACAGTGGTAAACATGGACGACCTAATCACAGTCCACCATGAGATGGGGCACGTTCAATATTTC	2952
Lipotes vexillifer	TCAGGATCAAGCAGTGCACCACTGTGAACATGGAGGACCTGGTGGTGGCCCACCACGAAATGGGCCACATACAGTATTTC	3012
Orcinus orca	TCAGGATCAAGCAGTGCACCACTGTGAACATGGAGGACCTGGTGGTGGCCCACCACGAAATGGGCCACATACAGTATTTC	2997
Homo sapiens	TCCGGATCAAGCAGTGCACCACCGTGAACTTGGAGGACCTGGTGGTGGCCCACCACGAAATGGGCCACATCCAGTATTTC	2988

	2970 2980 2990 3000 3010 3020 3030 3040	
conservation		
Callorhinchus milii	CTTCAGTACAAAGATCAGCCGGTCTCATTCCGAGATGGAGCAAACCCAGGATTCCACGAGGCAATTGGCGATGTTATGGC	3044
Alligator mississippiensis	CTGCAGTACAAGGACCAGCCCATATCTTTCCGTGATGGAGCCAACCCTGGCTTCCATGAGGCCATTGGGGATGTCATGGC	3032
Anolis carolinensis	CTGCAGTACAAGGACCAACCCATTTCATTTCGTGATGGGGCCAACCCAGGATTTCATGAAGCCATTGGAGATGTCATGGC	3104
Astyanax mexicanus	CTGCAGTACAAGGACCAGCCCATCTCCTTCCGTGACGGTGCCAACCCAGGATTCCATGAGGCTATTGGGGACGTCTTGGC	3032
Boleophthalmus pectinirostris	CTGCAGTACAAGGACCAGCCGGTGTCCTTCCGGGATGGAGCTAACCCCGGGTTCCACGAGGCCATCGGGGATGTCTTGGC	3038
Cynoglossus semilaevis	TTGCAGTACAAAGAGCAGCCCGTGTCGTTCCGTGACGGGGCCAATCCTGGGTTCCATGAGGCCATTGGCGATGTACTGGC	3044
Danio rerio	CTGCAGTATAAGGATCAGCCCATCTCTTTCCGTGACGGCGCCAACCCTGGTTTCCACGAGGCCATCGGTGACGTACTAGC	3152
Esox lucius	CTGCAGTACAAAGATCAGCCCATCTCCTTCCGTGACGGTGCCAACCCCGGCTTCCATGAGGCCATCGGTGATGTGCTGGC	3038
Fundulus heteroclitus	CTGCAGTACAAAGACCGGCCCGTGTCGTTTCGCGACGGTGCCAATCCTGGCTTCCACGAAGCCATCGGAGATGTGCTCGC	3050
Gekko japonicus	CTGCAGTACAAAGACCAGCCCATATCCTTCCGCGATGGGGCCAATCCGGGGTTCCATGAAGCCATCGGAGACGTCATGGC	3044
Haplochromis burtoni	CTGCAGTACAAAAACCAGCCCGTGTCCTTCCGTGACGGAGCCAACCCCGGCTTTCACGAGGCCATTGGAGATGTACTTGC	3041
Hippocampus comes	CTGCAATATAAGGATCAGCCCGTGGCGTTCCGCGAGGGCGCCAACCCTGGCTTCCACGAGGCCATCGGCGATGTCCTGGC	3026
Ictalurus punctatus	CTCCAGTATAAGGACCAGCCCATCTCGTTCCGTGACGGCGCCAACCCGGGATTCCATGAGGCCATCGGGGATGTGCTGGC	3038
Larimichthys crocea	TTACAGTACAAAGACCAGCCTGTGTCCTTCCGCGATGGAGCCAACCCTGGCTTCCATGAGGCCATCGGGGATGTTTTAGC	3035
Lates calcarifer	CTGCAGTACAAAGACCAGCCTGTGTCCTTCCGTGATGGAGCCAACCCTGGATTCCACGAGGCAATCGGCGATGTACTAGC	3038
Latimeria chalumnae	CTGCAGTACAAAGACCAACCCATCTCCTTTCGAGATGGTGCAAACCCTGGTTTCCATGAGGCAATCGGTGACGTCCTAGC	3026
Lepisosteus oculatus	CTGCAGTACAAGGACCAGCCTGTCCCGTTTAGGGATGGAGCCAACCCTGGCTTCCATGAGGCCATCGGGGACGTTCTGGC	3026
Macaca fascicularis	ATGCAGTACAAAGACTTGCCTGTGGCCTTGAGGGAGGGCGCCAACCCCGGCTTCCATGAGGCCATTGGGGACGTGCTAGC	3071
Manacus vitellinus	CTGCAGTACAAGGACCAGCCCGTCTCCTTCCGGGATGGGGCCAATCCCGGCTTCCACGAGGCCGTCGGGGATGTCATGGC	3023
Mus musculus	ATGCAGTACAAAGACTTACCCGTGACTTTCCGGGAGGGTGCCAACCCTGGTTTTCATGAAGCTATTGGAGATATAATGGC	3083
Neolamprologus brichardi	CTGCAGTACAAAGACCAGCCCGTGTCCTTCCGTGACGGAGCCAACCCCGGCTTTCACGAGGCCATTGGAGATGTACTTGC	3041
Oncorhynchus mykiss	CTGCAGTACAAGGACCAACCTATTTCCTTCCGCGACGGCGCCAACCCAGGCTTCCACGAGGCCATTGGTGATGTGCTGGC	3038
Oreochromis niloticus	CTGCAGTACAAGGACCAGCCCGTGTCCTTCCGTGACGGAGCCAACCCCGGCTTTCACGAGGCCATCGGAGATGTGCTTGC	3041
Poecilia formosa	CTGCAGTACAAAGACCAGCCTGTTTCATTCCGCACTGGCGCTAACCCAGGCTTCCACGAAGCCATCGGAGATGTTCTTGC	3047
Poecilia latipinna	CTGCAGTACAAAGACCAGCCTGTTTCATTCCGCACTGGTGCTAACCCAGGCTTCCACGAAGCCATCGGAGATGTTCTTGC	3047
Pseudopodoces humilis	CTGCAGTACAAGGACCAGCCTGTCTCCTTCCGTGATGGGGCCAACCCTGGCTTCCATGAGGCTGTTGGGGATGTCATGGC	3023
Pundamilia nyererei	CTGCAGTACAAAAACCAGCCCGTGTCCTTCCGTGACGGAGCCAACCCCGGCTTTCATGAGGCCATTGGAGATGTACTTGC	3041
Pygocentrus nattereri	CTGCAGTACAAGGAGCAGCCCATTTCCTTCCGCGATGGCGCCAACCCAGGATTCCACGAGGCTATTGGAGACGTCCTAGC	3020
Python bivittatus	TTGCAGTACAAAGACCAGCCCATTTCATTTCGTGATGGGGCCAATCCAGGATTCCATGAAGCCATTGGCGATGTTATGGC	3035
Rattus norvegicus	ATGCAGTACAAAGACTTGCCTGTGACCTTTCGGGAGGGCGCCAACCCCGGTTTTCATGAGGCTATTGGAGATGTTTTGGC	3086
Scleropages formosus	CTGCAATATAAAGAACAGCCCATTTCCTTCAGAGAAGGAGCCAACCCAGGATTCCATGAAGCTATTGGTGATGTGATGGC	3026
Sinocyclocheilus anshuiensis	CTGCAGTATAAGGATCAACCCATCTCTTTCCGCGACGGCGCCAACCCTGGATTCCATGAGGCCATTGGTGATGTTTTGGC	3023
Xenopus tropicalis	CTTCAGTACAAGGATCAGCCTATCTTGTTCCGTGAGGGAGCCAACCCAGGTTTCCATGAGGCAATTGGGGATGTGCTTGC	3032
Lipotes vexillifer	ATGCAGTACAAGGACTTGCCGGTGACCTTCCGGGAGGGCGCCAACCCCGGTTTTCACGAAGCCATTGGGGATGTGCTGGC	3092
Orcinus orca	ATGCAGTACAAGGACTTGCCGGTGACCTTCCGGGAGGGTGCCAACCCTGGTTTTCACGAGGCCATTGGGGATGTGCTGGC	3077
Homo sapiens	ATGCAGTACAAAGACTTACCTGTGGCCTTGAGGGAGGGTGCCAACCCCGGCTTCCATGAGGCCATTGGGGACGTGCTAGC	3068

```
                              3050      3060      3070      3080      3090      3100      3110      3120
conservation
Callorhinchus milii           TCTTTCCGTTTCCACTCCAAAACATCTGCACACCATCGGCCTACTCAGTGAAGTAGAGGACAATATCGAAATGGATATCA  3124
Alligator mississippiensis    CCTGTCAGTCTCCACCCCTAAGCACCTGCACAGCATCAACCTTCTTCCTCAGGTTGAGGATAATATGGAGAGCGACATCA  3112
Anolis carolinensis           CCTATCGGTGTCTACTCCAAAACACTTATTCAGCATCAAGCTGCTAGATCAGGTGGAAGACAATCCTGAGAGTGACATTA  3184
Astyanax mexicanus            CCTGTCAGTGTCCACTCCAAAACATCTCCACACCATCGGCCTGCTGGACAAACTGGAGGAAAACGCAGAAAGCACCATTA  3112
Boleophthalmus pectinirostris TCTGTCTGTGTCCACTCCCAAACATCTACAGAGCATTGGTCTGCTGGACAAAGTGGAAAGCAACTCTGAGAGTGACATTA  3118
Cynoglossus semilaevis        ACTGTCAGTGTCAACACCCAAACATCTGCACAGCATTGGTCTCCTGGACAAAGTGGAGAGCAACCACGAGAGCGACATCA  3124
Danio rerio                   TCTGTCAGTAGCCACACCTAAACATCTTCAGAGCATCGGCCTGCTGGACAAGGTGGAGGACAATGCAGAGAGCACCATCA  3232
Esox lucius                   CCTGTCCGTTGCCACTCCCAGACATTTAAAGGAGATTGGTTTACTAGATGTGGTGGAAGCCAACAAGGAGAGTACAATCA  3118
Fundulus heteroclitus         ACTGTCAGTCTCCACGCCCAAACATCTCCAGAGCATCGGCCTCCTGGACAAAGTGGAGAGTAACCACGAGAGTGACATAA  3130
Gekko japonicus               CTTGTCAGTGTCCACCCCCAAACACCTGTACAGCATCAGTCTCCTGGATCGGGTAGAAGACAACCTGGAGGGTGACATTA  3124
Haplochromis burtoni          CTTGTCTGTGTCGACACCCAAACATCTGCAGAGCATCGGCCTCCTGGACAAGGTTGAGAACAACTATGAGAGTGACATCA  3121
Hippocampus comes             TTTGTCCGTGTCCACACCCAAGCACCTGAAGAGCATCGGCCTCCTGGACAAAGTGGAAAACAATCACGAGAGTGACATCA  3106
Ictalurus punctatus           GCTTTCAGTGTCCACACCCAAACACCTTCACACCATCGGCCTGCTTGATAAGGTCGAGGACAACCCGGAGAGCACCATAA  3118
Larimichthys crocea           CCTGTCTGTTTCTACGCCCAAACATCTGCAGAGCATCGGCCTCCTGGACAAAGTAGAAAGCAACCATGAGAGTGATATCA  3115
Lates calcarifer              CCTTTCTGTGTCTACACCCAAACATCTGCAGAGCATTGGCCTCTTGGACAAAGTTGAGAACAACCATGAGAGTGATATCA  3118
Latimeria chalumnae           ACTATCTGTCTCCACCCCAAAACACCTTCACAGTATTGGGCTACTTGACACAGTCAATGAAAATTATGAGAGTGACATTA  3106
Lepisosteus oculatus          CCTGTCGGTTTCCACACCCAAGCACCTGCACAGCATCGGACTGCTGGACAAAGTGGAGAACAACTATGACAGTGACATTA  3106
Macaca fascicularis           CCTCTCAGTGTCTACGCCCAAGCACCTGCACAGTCTGAACCTGCTGAGCAGTGAGGGTGGCAGCCATGAGCATGATATCA  3151
Manacus vitellinus            CTTGTCCGTCTCCACCCCCAAACACCTGCACAGCATCAATCTGCTGGATGAAGTCAAGGAAGACCAAGAGAGTGACATTA  3103
Mus musculus                  TCTCTCAGTGTCTACCCCCAAGCATCTATACAGTCTCAACCTGCTTAGCACTGAGGGCAGTGGCTACGAGTATGACATCA  3163
Neolamprologus brichardi      CTTGTCTGTGTCGACACCCAAACATCTGCAGAGCATCGGCCTCCTGGACAAGGTTGAGAACAACTATGAGAGTGACATCA  3121
Oncorhynchus mykiss           CCTGTCCGTCGCCACTCCCAAGCATCTAAAGGAGATTGGTTTACTGGATGTGGTGGAGGACAACCCAGAGAGTACGATCA  3118
Oreochromis niloticus         CTTGTCTGTGTCAACACCCAAACATCTGCAGAGCATCGGCCTCCTGGACAAGGTTGAGAACAACTATGAGAGTGACATCA  3121
Poecilia formosa              GCTGTCCGTCTCCACGCCCAAACATCTGCAGAGCATCGGGCTCCTGGACAAAGTGGAAAGTAACCACGAGAGTGACATCA  3127
Poecilia latipinna            GCTGTCCGTCTCCACGCCCAAACATCTGCAGAGCATCGGGCTCCTGGACAAAGTGGAGAGTAACCACGAGAGTGACATCA  3127
Pseudopodoces humilis         CTTGTCTGTCTCCACCCCCAAACACCTGCATAGCATCAATCTGCTGGACCAAGTCATGGAAAATGAAGAAAGTGATATTA  3103
Pundamilia nyererei           CTTGTCTGTGTCGACACCCAAACATCTGCAGAGCATCGGCCTCCTGGACAAGGTTGAGAACAACTATGAGAGTGACATCA  3121
Pygocentrus nattereri         CCTGTCAGTGTCCACTCCTAAACACCTCCACAGCATTGGCTTGCTGGACAAGGTGGAGGACAACCAAGAGAGCACCATTA  3100
Python bivittatus             CTTATCAGTATCAACCCCAAAGCACTTGTATAGCATCAAGCTGCTTGATCATATGGAAGACAACCTTGAGGGTGACATCA  3115
Rattus norvegicus             TCTGTCTGTGTCTACACCCAAGCATCTACACAGTCTCAACCTGCTCAGCAGTGAGGGCAGTGGCTACGAGCATGACATCA  3166
Scleropages formosus          TCTGTCTGTCTCCACACCAATGCATCTGCACAGCATTGGTCTTCTGGACAATGCAGAATCCTCATATGAGAGTGACATTA  3106
Sinocyclocheilus anshuiensis  CCTGTCAGTTTCCACACCTAAACATCTTAAGAGCATTGGCCTGCTGGACAAGGTGGAGGACAATAAAGAAAGCACCATTA  3103
Xenopus tropicalis            TCTTTCAGTCTCTACACCAAAACACTTGCAAAGCATTGGCCTTTTGGATAAGGTGGAGGACAACCCAGAAAGTGATATTA  3112
Lipotes vexillifer            CCTCTCAGTGTCTACCCCCAAGCACCTGCACAAGATCAACCTGCTGAGTAGTGGGGACGGCAGCTATGAGGAGGACATCA  3172
Orcinus orca                  CCTCTCAGTGTCTACCCCCAAGCACCTGCACAAGATCAACCTGCTGAGTAGTGGGGACGGCAGCTATGAGGAGGACATCA  3157
Homo sapiens                  CCTCTCAGTGTCTACGCCCAAGCACCTGCACAGTCTCAACCTGCTGAGCAGTGAGGGTGGCAGCGACGAGCATGACATCA  3148
```

```
                              3130      3140      3150      3160      3170      3180      3190      3200
conservation
Callorhinchus milii           ACTACCTGTTGAGCATTGCTCTGGAAAAAATTGCTTTCATTCCATTTGGATATCTGATGGATAAGTGGCGATGGAAGGTT  3204
Alligator mississippiensis    ATTACCTGATGAGCATTGCCTTGGACAAGATTGCCTTTGTGCCTTTGGCTACCTTATGGACCAGTGGAGGTGGAAGGTG  3192
Anolis carolinensis           ACTACTTGATGAGCATTGCATTGGATAAGATTGCTTTCCTGCCCTTTGGCTATCTGATGGACCAATGGAGATGGAAAGTT  3264
Astyanax mexicanus            ACTTCCTGATGAGCATTGCCTTGGACAAAATTGCATTCCTGCCGTTCGGCTACCTAATGGACCAGTGGAGATGGAAAGTG  3192
Boleophthalmus pectinirostris ACTTCCTGATGAGCATGGCCCTTGATAAGATTGCCTTCCTGCCTTTTGGTTACCTTATGGACCAGTGGAGATGGAAAGTA  3198
Cynoglossus semilaevis        ACTTCCTGATGAGCATGGCTCTGGACAAGATTGCATTCCTACCTTTCGGGTTCCTGATGGACCAGTGGAGATGGAAAGTG  3204
Danio rerio                   ACTTTCTGATGAGCATTGCATTGGATAAGATTGCCTTCTTGCCATTTGGATACCTGATGGACCAGTGGAGGTGGAAAGTG  3312
Esox lucius                   ATTACCTCATGAGTATTGCTCTGGACAAAATCGCCTTCCTGCCCTTCGGTTACCTGATGGACCAGTGGAGGTGGAAAGTG  3198
Fundulus heteroclitus         ACTTTTTAATGAGCATGGCGCTGGACAAAATTGCCTTCCTGCCTTTCGGTTACCTGATGGATCAGTGGAGGTGGAAGGTC  3210
Gekko japonicus               ACTACTTGATGAGCATTGCACTGGACAAGATTGCCTTTCTGCCCTTTGGCTACCTCATGGACCAGTGGAGGTGGAAAGTG  3204
Haplochromis burtoni          ACTTCCTGATGAGCATTGCACTTGACAAGATTGCCTTCCTACCTTTCGGCTACCTGATGGACCAATGGAGGTGGAAGGTG  3201
Hippocampus comes             ACTTTCTGATGAACATGGCGCTCGATAAAATCGCCTTCCTGCCGTTTGGCTACCTGATGGACCAGTGGAGATGGAAGGTG  3186
Ictalurus punctatus           ACTTCCTGATGAGCATCGCCCTGGATAAAATCGCCTTCCTGCCTTTCGGCTACCTGATGGACCAATGGAGGTGGAAGGTG  3198
Larimichthys crocea           ACTTCCTGATGAACATGGCGCTTGACAAGATTGCCTTCCTTCCTTTGGCTACTTGATGGATCAGTGGAGATGGAAGGTG  3195
Lates calcarifer              ACTTCCTGATGAGCATGGCACTTGACAAGATCGCCTTCCTACCTTTTGGCTACCTGATGGACCAGTGGAGATGGAAGGTA  3198
Latimeria chalumnae           ACTATCTAATGAGTATTGCCCTGGATAAGATTGCATTCCTGCCCTTTGGGTATCTCATGGATCAGTGGCGATGGAAGGTG  3186
Lepisosteus oculatus          ACTATCTGATGAGCATTGCTTTGGACAAAATTGCCTTCCTGCCCTTCGGCTACCTGATGGACCAGTGGCGCTGGAAGGTT  3186
Macaca fascicularis           ACTTTCTGATGAAGATGGCGCTTGACAAGATCGCCTTTATCCCCTTCAGCTATCTCGTTGATCAGTGGCGCTGGAGGGTG  3231
Manacus vitellinus            ACTACCTGATGAGCATTGCCCTGGACAAAATCGCCTTCCTGCCCTTCGGGTACCTCATGGACCAGTGGCGCTGGAAGGTG  3183
Mus musculus                  ACTTTCTAATGAAGATGGCCCTCGACAAGATCGCCTTTATCCCCTTCAGCTACCTCATCGACCAGTGGCGCTGGAGGGTC  3243
Neolamprologus brichardi      ACTTCCTGATGAGCATTGCACTTGACAAGATTGCCTTCCTACCTTTCGGCTACCTGATGGACCAATGGAGGTGGAAGGTG  3201
Oncorhynchus mykiss           ATTACCTCATGAGCATCGCCCTGGACAAAATTGCCTTCCTGCCCTTCGGCTACTTGATGGACCAGTGGAGGTGGAAAGTG  3198
Oreochromis niloticus         ACTTCCTGATGAGCATGGCACTTGACAAGATTGCCTTCCTACCTTTCGGCTACCTAATGGACCAGTGGAGGTGGAAGGTG  3201
Poecilia formosa              ACTTTTTGATGAGCATGGCGCTAGACAAAATTGCCTTCCTGCCTTTCGGCTACCTGATGGACCAGTGGAGGTGGAAGGTC  3207
Poecilia latipinna            ACTTTTTGATGAGCATGGCGCTAGACAAAATTGCCTTCCTGCCTTTTGGCTACCTGATGGACCAGTGGAGGTGGAAGGTC  3207
Pseudopodoces humilis         ACTACCTGATGAGCATCGCCCTGGATAAAATTGCCTTCCTGCCCTTTGGGTACCTCATGGACCAGTGGCGCTGGAAGGTG  3183
Pundamilia nyererei           ACTTCCTGATGAGCATTGCACTTGACAAGATTGCCTTCCTACCTTTCGGCTACCTGATGGACCAATGGAGGTGGAAGGTG  3201
Pygocentrus nattereri         ACTTCCTAATGAGCATTGCCTTGGACAAAATCGCCTTCCTGCCATTCGGCTACCTGATGGACCAGTGGCGGTGGAAGGTG  3180
Python bivittatus             ACTACCTGATGAGCATTGCACTGGATAAGATTGCTTTCCTGCCCTTTGGCTACCTGATGGACCAATGGAGGTGGAAAGTG  3195
Rattus norvegicus             ACTTTCTAATGAAGATGGCCCTTGACAAGATCGCCTTCATCCCCTTCAGCTACCTCATTGACCAGTGGCGCTGGAGGGTC  3246
Scleropages formosus          ATTACTTGATGAGCATTGCTCTGGACAAAATCGCCTTCCTGCCTTTCGGCTACTTGATGGACCAGTGGAGGTGGAAGGTG  3186
Sinocyclocheilus anshuiensis  ACTTCCTGATGAGCATGGCTTTGGATAAGATTGCCTTCCTGCCTTTGGCTACCTGATGGACCAGTGGAGGTGGAAGGTA  3183
Xenopus tropicalis            ATTATCTCATGAGTATTGCACTGGATAAAATTGCCTTCCTTCCATTTGGTTTCCTCATGGATCAGTGGCGCTGGAAGGTG  3192
Lipotes vexillifer            ACTTTCTGATGAAGATGGCGCTCGACAAGATCGCCTTTGTTCCCTTCAGCTATCTTGTGGACCAGTGGCGCTGGAGGGTA  3252
Orcinus orca                  ACTTTCTGATGAAGATGGCGCTCGACAAGATCGCCTTTGTTCCCTTCAGCTATCTTGTGGACCAGTGGCGCTGGAGGGTG  3237
Homo sapiens                  ACTTTCTGATGAAGATGGCCCTTGACAAGATCGCCTTTATCCCCTTCAGCTACCTCGTCGATCAGTGGCGCTGGAGGGTA  3228
```

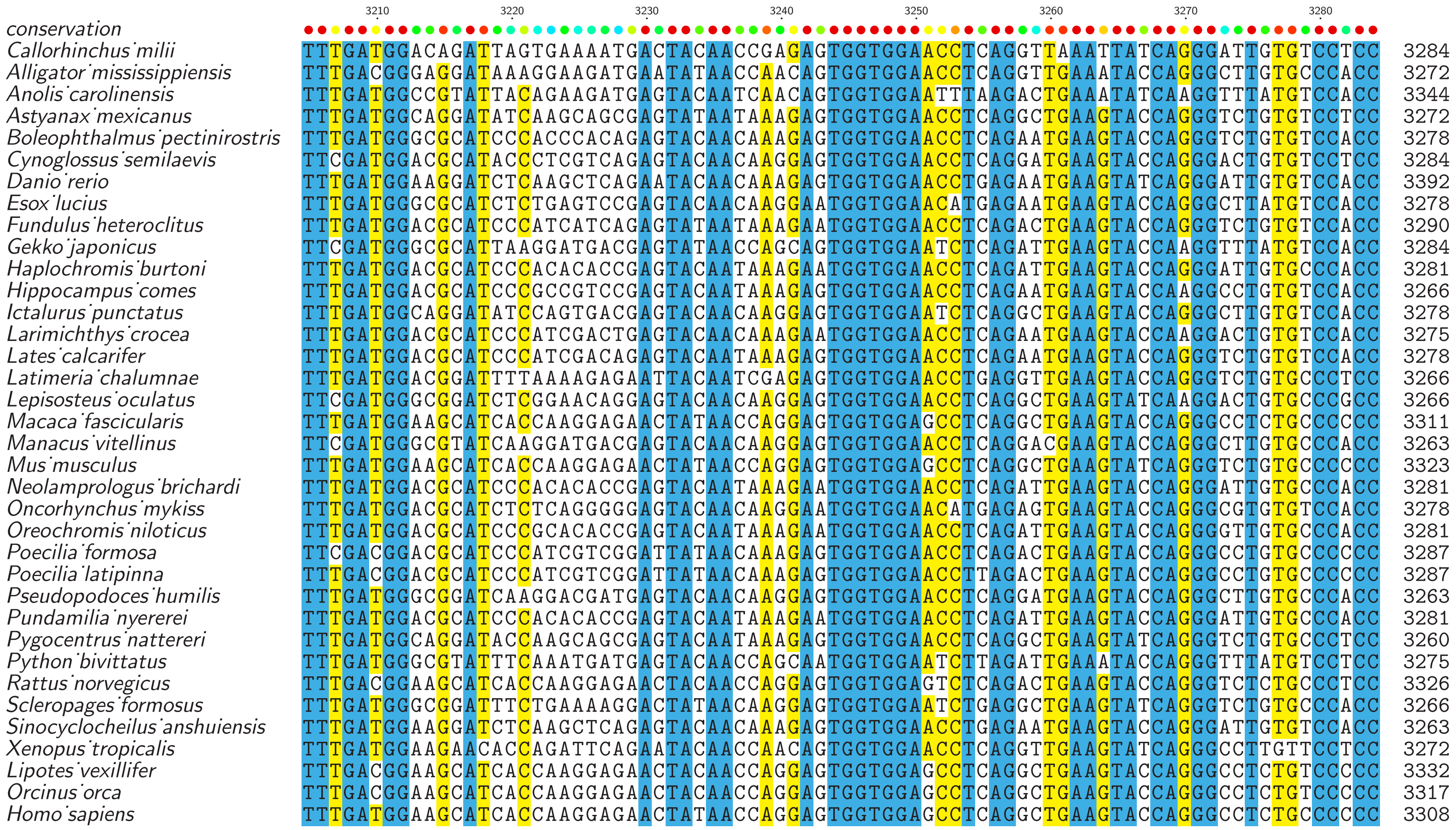
3210
3220
3230
3240
3250
3260
3270
3280
conservation
Callorhinchus milii 3284
Alligator mississippiensis 3272
Anolis carolinensis 3344
Astyanax mexicanus 3272
Boleophthalmus pectinirostris 3278
Cynoglossus semilaevis 3284
Danio rerio 3392
Esox lucius 3278
Fundulus heteroclitus 3290
Gekko japonicus 3284
Haplochromis burtoni 3281
Hippocampus comes 3266
Ictalurus punctatus 3278
Larimichthys crocea 3275
Lates calcarifer 3278
Latimeria chalumnae 3266
Lepisosteus oculatus 3266
Macaca fascicularis 3311
Manacus vitellinus 3263
Mus musculus 3323
Neolamprologus brichardi 3281
Oncorhynchus mykiss 3278
Oreochromis niloticus 3281
Poecilia formosa 3287
Poecilia latipinna 3287
Pseudopodoces humilis 3263
Pundamilia nyererei 3281
Pygocentrus nattereri 3260
Python bivittatus 3275
Rattus norvegicus 3326
Scleropages formosus 3266
Sinocyclocheilus anshuiensis 3263
Xenopus tropicalis 3272
Lipotes vexillifer 3332
Orcinus orca 3317
Homo sapiens 3308

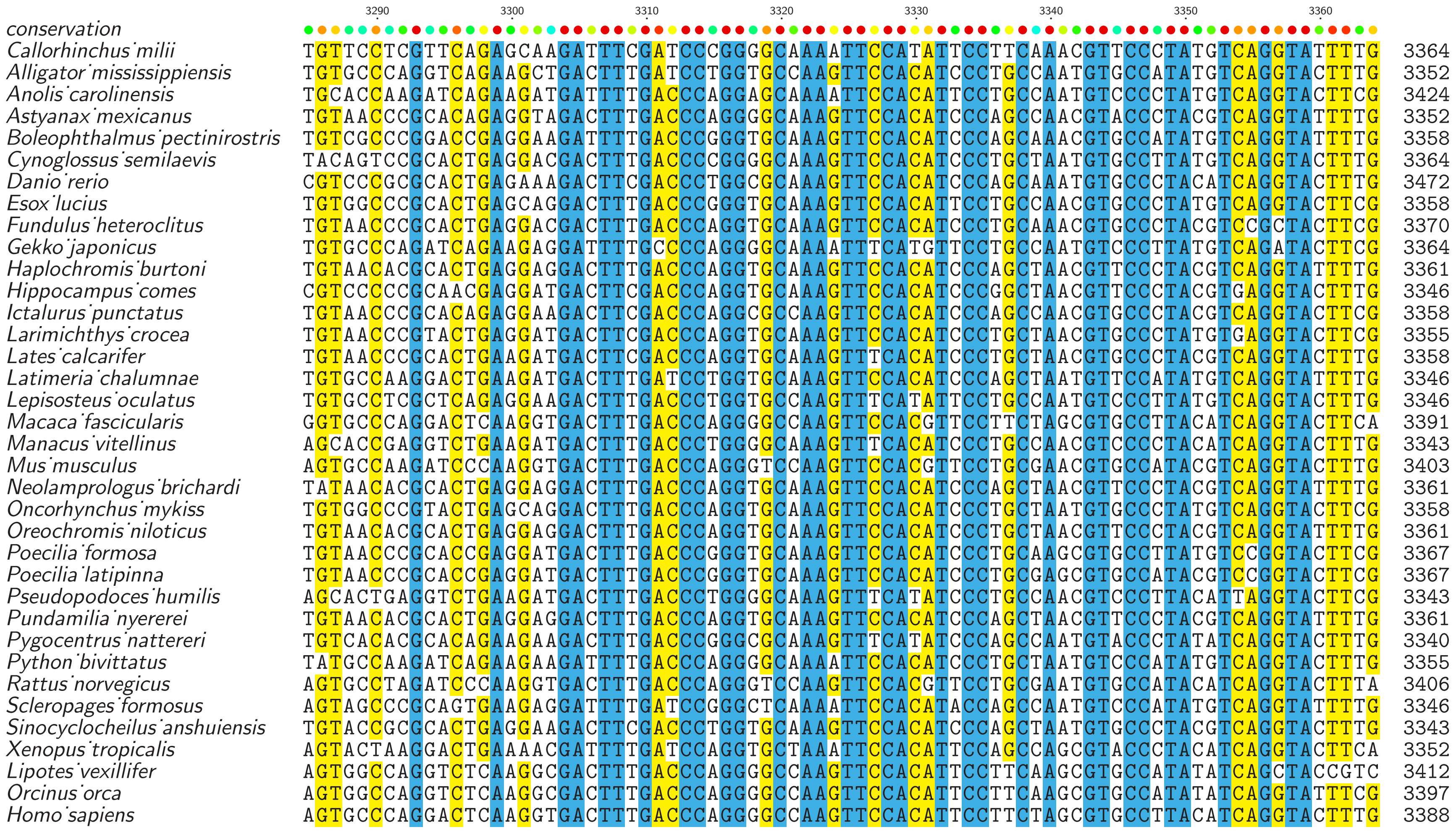
3290
3300
3310
3320
3330
3340
3350
3360
conservation
Callorhinchus milii 3364
Alligator mississippiensis 3352
Anolis carolinensis 3424
Astyanax mexicanus 3352
Boleophthalmus pectinirostris 3358
Cynoglossus semilaevis 3364
Danio rerio 3472
Esox lucius 3358
Fundulus heteroclitus 3370
Gekko japonicus 3364
Haplochromis burtoni 3361
Hippocampus comes 3346
Ictalurus punctatus 3358
Larimichthys crocea 3355
Lates calcarifer 3358
Latimeria chalumnae 3346
Lepisosteus oculatus 3346
Macaca fascicularis 3391
Manacus vitellinus 3343
Mus musculus 3403
Neolamprologus brichardi 3361
Oncorhynchus mykiss 3358
Oreochromis niloticus 3361
Poecilia formosa 3367
Poecilia latipinna 3367
Pseudopodoces humilis 3343
Pundamilia nyererei 3361
Pygocentrus nattereri 3340
Python bivittatus 3355
Rattus norvegicus 3406
Scleropages formosus 3346
Sinocyclocheilus anshuiensis 3343
Xenopus tropicalis 3352
Lipotes vexillifer 3412
Orcinus orca 3397
Homo sapiens 3388

	3370–3440	
conservation		
Callorhinchus milii	TGAGCTTCATCATCCAGTTCCAGTTCCATGAACGCCTTTGTGAAGTAGCAAACCAAACAGGTTCCCTGTTTAAGTGTGAC	3444
Alligator mississippiensis	TCAGCTTTGTGATTCAGTTCCAGTTCCACCAAGCCCTCTGCAAAGCAGCTGGACACACTGGCCCCTTGTACAAGTGTGAC	3432
Anolis carolinensis	TTAGTTTTGTTATCCAGTTCCAGTTCCACCAAGCTCTTTGTAAAGCTGCTGGCCACACAGGACCCTTGCACAAGTGTGAC	3504
Astyanax mexicanus	TCAGCTTTGTGGTTCAGTTCCAGTTCCACAAAGCTCTGTGTGACACTGCTGGCCACACTGGTCCTCTGCACAACTGTGAT	3432
Boleophthalmus pectinirostris	TCAGCTTCGTGATCCAGTTTCAGTTCCACAAAGCTCTGTGTAATGCCGCCAAACACACTGGTCCCCTTCATACATGTGAT	3438
Cynoglossus semilaevis	TCAGCTTCATCATCCAGTTCCAGTTCCACAAAGCTCTGTGTGAGGCTGCCAAACATGAGGGTCCTCTCCATACATGTGAC	3444
Danio rerio	TGAGTTTTGTAATCCAGTTCCAGTTCCACAAAGGCCTGTGTGATGCTGCGGGTCACAAAGGGCCGCTCCACAACTGTGAC	3552
Esox lucius	TGAGTTTCATCATTCAGTTTCAGTTCCACCAGGCTCTTTGTAAGGCTGCCAACCATAAAGGGCCTCTTCATGAATGCGAT	3438
Fundulus heteroclitus	TAAGCTTCATCATCCAGTTTCAGTTCCACAAAGCTCTGTGCGATGCTGCCAATCATGTTGGGCCTCTGCACACCTGTGAT	3450
Gekko japonicus	TCAGTTTCATCATCCAGTTCCAGTTCCACCAAGCTCTGTGCGAAGCTGCTGGCCAGGTGGGGCCCTTGCACAAGTGTGAC	3444
Haplochromis burtoni	TCAGCTTTGTCATCCAGTTTCAGTTCCACAAAGCTCTGTGTAATGCTGCCAAGCATAATGGACCTTTATACACTTGTGAC	3441
Hippocampus comes	TCAGCTTCATTATCCAGTTTCAGTTCCACAAGGCCCTGTGTAACGCTGCCAAGCACGTGGGGCCCCTGCACACGTGCGAC	3426
Ictalurus punctatus	TCAGCTTTGTGATCCAGTTCCAGTTCCATAAAGCTCTGTGTGATGCTGCGGGTCACACTGGACCTCTCCACAACTGTGAT	3438
Larimichthys crocea	TCAGTTTCATCATCCAATTTCAGTTCCACAAGGCTCTGTGTGAAGCTGCTAACCATGTGGGCCCTCTTCATACCTGCGAC	3435
Lates calcarifer	TCAGCTTCGTCATCCAGTTCCAGTTCCACAAAGCTCTGTGTGATGCTGCCAATCATGTTGGGCCTCTCCACACCTGCGAT	3438
Latimeria chalumnae	TTAGCTTCATAATCCAGTTCCAGTTCCATGAGGCTCTGTGTAAGGCAACCAAGCAAACGGGTCCTCTTCACAAATGTGAC	3426
Lepisosteus oculatus	TCAGCTTCGTGATCCAGTTCCAGTTCCATGAGGCTCTCTGCAAGGCAGCAAAACACACTGGCCCACTCCACATGTGTGAC	3426
Macaca fascicularis	TCAGCTTCATCATCCAGTTCCAGTTCCATGAAGCACTGTGCCAGGCAGCTGGCCACACGGGCCCCCTGCACAAGTGTGAC	3471
Manacus vitellinus	TCAGCTTCGTGATCCAGTTCCAGTTCCACCAGGCTCTCTGTAATGCAGCCGGGCACACGGGTGCCCTGCACACCTGTGAC	3423
Mus musculus	TCAGCTTCATCATCCAGTTCCAGTTCCACGAGGCGCTGTGTCGCGCAGCCGGGCACACGGGTCCCCTGCACAAGTGTGAC	3483
Neolamprologus brichardi	TCAGCTTCATCATCCAGTTTCAGTTCCACAAAGCTCTGTGTAATGCTGCCAAGCATAATGGACCTTTATACACCTGTGAC	3441
Oncorhynchus mykiss	TGAGCTTCATCATCCAGTTCCAGTTTCATCAGGCTCTGTGTAACACCGCCGGGCATGTAGGCCCTCTCCACCAATGTGAT	3438
Oreochromis niloticus	TCAGCTTCGTCATCCAGTTTCAGTTCCACAAAGCTCTGTGTAATGCTGCCAAGCATAATGGACCTTTATACACCTGTGAC	3441
Poecilia formosa	TCAGCTTCGTCATCCAGTTTCAGTTCCACAAAGCTCTGTGTGATGCAGCTAAGCATAATGGGCCTCTGCACACGTGTGAT	3447
Poecilia latipinna	TCAGCTTCGTCATCCAGTTTCAGTTCCACAAAGCTCTGTGTGACGCAGCTAAGCATAATGGGCCTCTGCACACGTGTGAT	3447
Pseudopodoces humilis	TCAGCTTTGTGATCCAGTTCCAGTTCCACCAGGCACTCTGTGATGCAGCTGGGCACAAGGGTCCCCTGCACACCTGTGAC	3423
Pundamilia nyererei	TCAGCTTCATCATCCAGTTTCAGTTCCACAAAGCTCTGTGTAATGCTGCCAAGCATAATGGACCTTTATACACTTGTGAC	3441
Pygocentrus nattereri	TCAGCTTTGTGATCCAGTTCCAGTTCCATAAGGCTCTGTGTGAAGCTGCTGGTCACACTGGCCCTTTGCACAACTGTGAC	3420
Python bivittatus	TCAGTTTTGTAATTCAGTTCCAGTTTCACCAAGCTCTGTGCAAAGCTTCCGGCCATACTGGACCTTTATACAAATGTGAC	3435
Rattus norvegicus	TCAGCTTCATCATCCAGTTCCAGTTCCACGAGGCACTATGTCGCGCAGCCGGGCACACCGGCCCCCTGTACAAGTGTGAT	3486
Scleropages formosus	TGAGCTTTGTCATCCAGTTCCAGTTCCACCAGGCCCTGTGCCAAGCGGCCAATCATGTTGGCCCACTCCACACTTGCGAC	3426
Sinocyclocheilus anshuiensis	TGAGTTTTGTAATCCAATTCCAGTTCCACAAAGCCCTGTGTGAAGCAGCCGGTCAGCCAGCTCCACTCCACAACTGTGAC	3423
Xenopus tropicalis	TCAGTTTTGTCATCCAGTTCCAGTTTCATGAAGCTCTTTGTAAAGCAGCAAATCAGGAAGGACCTCTGCACAAATGTGAC	3432
Lipotes vexillifer	AGGGC...GACCTGGGCACCCGGCTGCATGGCCCACTGTGT......CTGGGCCACTCA.....................	3462
Orcinus orca	TCAGCTTCGTCATCCAGTTCCAGTTTCACGAGGCGCTGTGTCAGGCGGCGGGCCACAAGGGCCCCCTGCACAAGTGCGAC	3477
Homo sapiens	TCAGCTTCATCATCCAGTTCCAGTTCCACGAGGCACTGTGCCAGGCAGCTGGCCACACGGGCCCCCTGCACAAGTGTGAC	3468

	3450–3520	
conservation		
Callorhinchus milii	ATCTATAAGTCCAAGGAAGCTGGAAAATTAATTGCGGATGTCATGAAGCTTGGTAGCAGCAAGCCCTGGTCTGAAGCCAT	3524
Alligator mississippiensis	ATCTACAAATCCAAGGAGGCAGGGACCCTCCTGGGGAATGCCCTGAAGCTGGGCTTTAGCAAACCCTGGCCAGAGGCCAT	3512
Anolis carolinensis	ATCTACAACTCTGAGGCAGCTGGAACAATCCTGGGGAATGCCTTGAAGCTGGGCTTCAGCAAACCATGGCCAGAAGTCAT	3584
Astyanax mexicanus	ATCTATCAGTCTAAAGAGGCAGGGAAACTGCTGGGTGATGTGATGAAGCTGGGCTTCAGTAAGCCCTGGCCTGAGGCTAT	3512
Boleophthalmus pectinirostris	ATTTACCAATCTAAAGAGGCTGGGAAGCTTATGGGTGACGTAATGAAGCTAGGATTCAGTAAACCCTGGCCTGAAGCTAT	3518
Cynoglossus semilaevis	ATCTACAAGTCTAAAGAAGCTGGGAAGCTACTGGGTGACGTGATGAAGCAGGGCTTCAGTAAACCCTGGCCTGAGGCCAT	3524
Danio rerio	ATCTACCAGTCCAAAGAAGCAGGAAAACTCCTGAGTGATGTGATGAAGATGGGCTTCAGTAAACCCTGGCCTGAAGCCAT	3632
Esox lucius	ATCTACCGCTCTAAAGAGGCAGGGAAACTTCTAGGTGATGTAATGAAGCTCGGTTTCAGTAAGCCCTGGCCTGAAGCAAT	3518
Fundulus heteroclitus	ATCTACCAATCCAAAGAAGCTGGGAAGCTGCTGGGGGATGTGATGAAGCTCGGGTACAGTAAGCCGTGGCCTGAAGCCAT	3530
Gekko japonicus	ATATATCAGTCAAAGCAGGCTGGAACAATTCTGGGGAATGCCATGAAACTGGGCTACAGCAAGCCATGGCCTGAAGCCAT	3524
Haplochromis burtoni	ATCTACCAATCCAAAGAAGCTGGAAAGCTCTTAGGTGATGTAATGAAGCTGGGCTTCAGCAAGCCCTGGCCTGAGGCTAT	3521
Hippocampus comes	ATTTACAAATCGAAGGAGGCTGGGAAGCTGCTAGGAGACGTGATGAAGCTGGGTTTCAGCAAGCCCTGGCCCGAGGCCAT	3506
Ictalurus punctatus	ATCTATAAGTCCACAAAGGCAGGACAACTCCTCGGTGATGTGATGAAGCTGGGCTTCAGTAAGCCCTGGCCCGAGGCGAT	3518
Larimichthys crocea	ATCTACAAATCTAAGGAAGCCGGGAAACTGCTGGGTGATGTGATGAAGCTTGGCTTCAGTAAGCCCTGGCCCGAGGCTAT	3515
Lates calcarifer	ATCTACAAATCTAAAGAAGCTGGGAAGCTGCTGGGTGACGTGATGAAGCTGGGCTTCAGTAAGCCCTGGCCTGAGGCTAT	3518
Latimeria chalumnae	ATCTACAAGTCAAATGCTGCAGGCAAGCTGCTCGGTGATGCTCTGAAGCTAGGGTTCAGCAAACCCTGGCCAGAGGCAAT	3506
Lepisosteus oculatus	ATCTACAACTCCAGGGAGGCTGGCAAGTTACTGGGGGATGTCATGCAGCTGGGGTTCAGTAAACCGTGGCCAGAGGCCAT	3506
Macaca fascicularis	ATCTACCAGTCCAAGGAGGCAGGGCAGCGCCTGGCGACGGCCATGAAGCTGGGCTTCAGTAGGCCGTGGCCAGAAGCCAT	3551
Manacus vitellinus	ATCTACCAGTCCACGGAGGCTGGGAAGATCCTGGGGGATGCCCTGAAGCTGGGTTTCAGCAAGCCATGGCCTGAGGCCAT	3503
Mus musculus	ATCTACCAATCCAAGGAAGCAGGGAAGCTCCTGGCGGATGCCATGAAGCTGGGCTACAGTAAGCCGTGGCCAGAGGCCAT	3563
Neolamprologus brichardi	ATCTACCAATCCAAAGAAGCTGGAAAGCTCTTAGGTGATGTAATGAAGCTGGGCTTCAGCAAGCCCTGGCCTGAGGCTAT	3521
Oncorhynchus mykiss	ATCTACCGCTCCAAAGAGGCAGGGAAACTTCTAGGTGACGTGATGAAGCTGGGTTTTAGTAAGCCCTGGCCTGAAGCGAT	3518
Oreochromis niloticus	ATCTACCAATCCAAAGAAGCTGGAAAGCTCTTAGGTGATGTAATGAAGCTGGGCTTCAGCAAGCCCTGGCCTGAGGCTAT	3521
Poecilia formosa	ATCTACCAATCTAAAGAAGCTGGGAAGCTGCTGGGGGATGTGATGCGGCTCGGTTACAGTAAGCCATGGCCGGAAGCCAT	3527
Poecilia latipinna	ATCTACCAATCTAAAGAAGCTGGGAAGCTGCTGGGGGATGTGATGCGGCTCGGTTACAGTAAGCCATGGCCGGAAGCCAT	3527
Pseudopodoces humilis	ATCTACCAGTCCCAGGAGGCTGGGAAGATCTTGGGGGATGCCCTGAAGCTGGGTTTCAGCAAGCCGTGGCCCGAAGCCAT	3503
Pundamilia nyererei	ATCTACCAATCCAAAGAAGCTGGAAAGCTCTTAGGTGATGTAATGAAGCTGGGCTTCAGCAAGCCCTGGCCTGAGGCTAT	3521
Pygocentrus nattereri	ATCTATGAGTCCAAAAAAGCAGGAAAACTGCTGGGTGATGTGATGAAGCTTGGCTATAGTAAGCCCTGGCCTGAGGCGAT	3500
Python bivittatus	ATTTACCAGTCTAAAGAAGCTGGGAAAATCCTAGCGAATGCCTTGAAGCTGGGCTTCAGCAGGCCGTGGCCTGAAGTCAT	3515
Rattus norvegicus	ATCTACCAATCCAAGGAAGCAGGGAAGCTGCTGGCAGATGCCATGAAGTTGGGCTACAGTAAGCAGTGGCCAGAAGCCAT	3566
Scleropages formosus	ATCTATCAATCAAAGGCCGCTGGGAAGCTACTGGGAGATGTGATGAAGCTTGGCTTCAGCAAGCCTTGGCCGGAAGCCAT	3506
Sinocyclocheilus anshuiensis	ATCTACCAGTCCAAAGAAGCAGGGAAACTCCTGGGTGATGTGATGAAGATGGGCTTCAGTAAACCCTGGCCCGAAGCCAT	3503
Xenopus tropicalis	ATCTACCAATCCACGCAGGCTGGAAAACTGCTCGGAGATGCCATGAAGTTGGGAAACAGCAAACCTTGGCCAGAAGCTAT	3512
Lipotes vexillifer	......CCTTCCCAGGAGGCCGGGAAGCGCCTGGCCGACGCCATGAAGCTGGGCTTCAGTCGGCCGTGGCCCGAAGCCAT	3536
Orcinus orca	ATCTACCAGTCCCAGGAGGCCGGGAAGCGCCTGGCCGACGCCATGAAGCTGGGCTTCAGTCGGCCGTGGCCCGAAGCCAT	3557
Homo sapiens	ATCTACCAGTCCAAGGAGGCCGGGCAGCGCCTGGCGACCGCCATGAAGCTGGGCTTCAGTAGGCCGTGGCCGGAAGCCAT	3548

```
                               3530      3540      3550      3560      3570      3580      3590      3600
conservation
Callorhinchus milii            GAAGTTAATAACAGGGAAGCCAGATATGTCTGCTGAGCCTCTACTGAAGTACTTCAAGCCACTGACAGACTGGCTTATTG  3604
Alligator mississippiensis     GCAGCTTATCACCGGGCAGCCCAACATGTCTGCAGATGCCCTGATGATGTATTTGAGCCGCTGATGACCTGGCTGGTGG  3592
Anolis carolinensis            GCAACTTATCACAGGGCAACCCAACATGTCGGCTGATGCTTTGTTGACTTATTTCCAACCACTGACAGACTGGCTCATCA  3664
Astyanax mexicanus             GGCTATGATCACTGGCCAGCCCCAAATGTCTGTGCAGCCTCTGATGGAGTATTTCCAGCCCCTCATCACCTGGCTCGAGC  3592
Boleophthalmus pectinirostris  GGCCATGATCACCGGTCAGCCCAAAATGAGCGCCCTGCCGCTCATGGAGTATTTCCAACCACTCATCACTTGGTTGGAGG  3598
Cynoglossus semilaevis         GACCATGATCACGGGCCAGCCCAAAATGACAGCCCAGCCTCTCATGGAGTACTTCCAACCCCTCATCCAGTGGCTGGAGT  3604
Danio rerio                    GAAGATCATCACAGGTCAGCCCAAAATGTCCGTCCAGCCTCTGATGGAGTATTTCAAACCGCTCATCGAGTGGCTGGAGA  3712
Esox lucius                    GGCCATGATCACTGGAGAGCCCGTCATGTCCGCCAAGCCACTGGTTGAGTACTTCAAACCCCTCACTGATTGGCTGGAGG  3598
Fundulus heteroclitus          GGCCATGATCACGGGTCAGTCCAAAATGACTGCCCAGCCGCTCATGCAGTACTTTGAACCTCTCATTAAATGGCTTGAGG  3610
Gekko japonicus                GCAGCTTATCACAGGACAGCCCAACATGTCGGCAGAAGCATTGCTGACCTATTTCCACCCACTAACAAACTGGCTAATCA  3604
Haplochromis burtoni           GGCTATGATCACTGGCGAGTCCAAAATGAGTGCCCAGCCACTCATGGAGTACTTCCAACCTCTCATTGAATGGCTTGAGA  3601
Hippocampus comes              GACCATGATCACAGGCGAGCCCACGATGAGCGCCAAGCCGCTCATGGAGTATTTCGAACCTCTCATTAAATGGCTGGAGC  3586
Ictalurus punctatus            GGCCTTGATCACAGGTAAGCCCGTGATGTCTGTACAGCCTCTGATGGAGTACTTTGAGCCCCTCATCACCTGGCTAGAGA  3598
Larimichthys crocea            GGCCATGATCACTGGCCAGCCCAAAATGACTGCCCAGCCACTCATGCAGTATTTCAAACCTCTCATTGACTGGCTGGAGA  3595
Lates calcarifer               GACCATGATCACTGGCCAGCCCAAAATGAGTGCCCAGCCACTCATGCAGTATTTCCAACCTCTCATTCAATGGCTGGAGG  3598
Latimeria chalumnae            GACTCTCATCACTGGACAGCCTAACATGACAGCTGAGGCTCTGATGAAGTATTTGAACCACTGACAAAGTGGCTTATTA  3586
Lepisosteus oculatus           GACGTTGATCACTGGGCAGCCCAACATGTCGGCCCAGGCTCTGCTGAAATACTTCGAGCCCCTGATTACCTGGCTAGAGA  3586
Macaca fascicularis            GCAGCTGATCACGGGCCAGCCCAATATGAGCGCCTCGGCCATGCTGAGCTACTTCAAGCCGCTGCTGGACTGGCTCCGCA  3631
Manacus vitellinus             GCAGCTCATCACGGGGCAGCCCAACATGTCAGCAGAGGCCCTGATGAGCTACTTTGAGCCGCTCATGACATGGCTGGAGA  3583
Mus musculus                   GAAGCTGATCACAGGCCAGCCTAACATGTCAGCCTCCGCCATGATGAATTACTTCAAGCCACTGACAGAATGGCTCGTCA  3643
Neolamprologus brichardi       GGCTATGATCACTGGCGAGTCCAAAATGAGTGCCCAGCCACTCATGGAGTACTTCCAGCCTCTCATTGAATGGCTTGAGA  3601
Oncorhynchus mykiss            GGCTATGATCACTGGAGAGCCTACCATGTCCGCCAAGCCACTGGTACAGTACTTCAAACCTCTCACTGATTGGCTGGAGG  3598
Oreochromis niloticus          GGCTATGATCACTGGCGAGTCCAAAATGAGTGCCCAGCCACTCATGGAGTACTTCCAGCCTCTTATTGAATGGCTCGAGA  3601
Poecilia formosa               GGCCATGATCACGGGTCAGTCCAAGATGAGCGCCCAGCCTCTCATGCAGTACTTCCAACCTCTCATCACATGGCTGGAGG  3607
Poecilia latipinna             GGCCATGATCACGGGTCAGTCCAAGATGAGCGCCCAGCCTCTCATGCAGTACTTCCAACCTCTCATCACATGGCTGGAGG  3607
Pseudopodoces humilis          GCAGCTCATCACAGGGCAGCCCAACATGTCAGCAGAGGCCCTTATGAGCTACTTCGAGCCACTCATGACGTGGCTGGAGA  3583
Pundamilia nyererei            GGCTATGATCACTGGCGAGTCCAAAATGAGTGCCCAGCCACTCATGGAGTACTTCCAACCTCTCATTGAATGGCTTGAGA  3601
Pygocentrus nattereri          GGCTATGATCACTGGCCAGCCTGAAATGTCTGTGCAGCCTCTCATGGAGTACTTCCAGCCCCTCATCACCTGGTTAGAGC  3580
Python bivittatus              GCAACTTATCACAGGACAATCCAACATGTCCGCTGAAGCTTTGCTGACCTATTTTGAACCACTGACAAACTGGCTTATTG  3595
Rattus norvegicus              GAAGATAATCACAGGCCAACCTAACATGTCAGCCTCTGCCATTATGAATTACTTCAAGCCACTGACTGAATGGCTCGTCA  3646
Scleropages formosus           GACTATGATCACTGGTCAGCCCAACATGTCTGCGCAGGCATTAATTGAATACTTCCAGCCTCTCATCAAGTGGCTGGAGA  3586
Sinocyclocheilus anshuiensis   GACTTTGATTACTGGCCAGCCCAAAATGTCAGTCCAGCCTCTGATGGAATACTTCCAACCGCTCATCGAGTGGCTGGTGG  3583
Xenopus tropicalis             GCAAACAATTACTGGACAAAGGAACATGTCTGCTCATGCCCTTCTCAAATACTTCCAGCCCCTGACTGACTGGCTTATCA  3592
Lipotes vexillifer             GCGGCTCATCACAGGCCAGCCCAACATGTCGGCCGCTGCCTTGATGACCTACTTCAAGCCGCTGCTGGACTGGCTCGTGA  3616
Orcinus orca                   GCGGCTCATCACGGGCCAGCCCAAGATGTCGGCCGCTGCCATGATGACCTACTTCAAGCCGCTGCTGGACTGGCTCGTGA  3637
Homo sapiens                   GCAGCTGATCACGGGCCAGCCCAACATGAGCGCCTCGGCCATGTTGAGCTACTTCAAGCCGCTGCTGGACTGGCTCCGCA  3628
```

```
                               3610      3620      3630      3640      3650      3660      3670      3680
conservation
Callorhinchus milii            AGAAGAACATCAAGAATGGAGAGACCCTGGGATGGCCTGAATACAATTGGACTCCAGCTCTAGGCTCAGACAATCTGCCA  3684
Alligator mississippiensis     ATGTGAACAAGAAGAATGGGGACATTCTGGGCTGGTCTGATTACGACTGGACGCCATATGCAGCTCGA............  3660
Anolis carolinensis            ACCAGAACTTGAAAAATCAAGAAACTCTAGGCTGGCCTGAATATACCTGGCTACCCTATGCGGGCCAA............  3732
Astyanax mexicanus             AGGAGAACGCAAAGAATGGAGATGTCCTCGGATGGCCTGAGTATAGCTGGATGCCAAACACAGCTGAGAGTAACTCT...  3669
Boleophthalmus pectinirostris  AGGAGAATGCCAAGAATGGAGATGTTCTTGGATGGCCCGAGTACGACTGGAAACCCTACGTGGTGTCAAAACAA......  3672
Cynoglossus semilaevis         TAGAGAATAACAAGAACAGAGATGTTCGTGGGTGGCCTGACTACGACTGGAGACCTGGGAGTGAA...CAGGAAAAA...  3678
Danio rerio                    AGGAGAACGAGAAGAATGGCGACGTGCTGGGATGGCCAGAGTACGACTGGACACCTTACAAACTCAGCACA.........  3783
Esox lucius                    CAGAGAACAACAAGAATGGCGAGGTCAGAGGTTGGCCTGAGTACGACTGGAAACCGCCAAGTCCTGGCTCCGTAACACTT  3678
Fundulus heteroclitus          AGGAGAACAACAAGAACAGCGAAGTCCGTGGATGGCCCGACTACGACTGGAAACCTTCAGATCCTGGCACTGAAGTC...  3687
Gekko japonicus                ATGAGAACATGAAAAACGGGGAAACCCTGGGCTGGCCTGAATACACCTGGACGCCCTACGCAGGCCAA............  3672
Haplochromis burtoni           AGGAGAACAGCAAGAACAATGATGTTCGCGGGTGGCCAGATTACGACTGGAAGCCTTTTAGT..................  3663
Hippocampus comes              GAGAGAACGATAAGAACGGGGACATCCGCGGGTGGCCTGATTACGACTGGAAGCCTTTGACGGGT......GACCCC...  3657
Ictalurus punctatus            TGGAGAACAAAAAGAACGGCGACGTCCTCGGCTGGCCCGACTACAACTGGATGCCAACCACGACTGCAGTT.........  3669
Larimichthys crocea            CAGAAAACAACAAGAACAATGAGGTCCGCGGGTGGCCCGAATACGACTGGAAACCTTTAAGCTCC...GATCCAGAT...  3669
Lates calcarifer               CAGAGAACAACAAGAACAACGATATCCGCGGGTGGCCTGAGTACAACTGGAAACCTAGTATGCCT...............  3663
Latimeria chalumnae            AACAGAATGAAAAGAATAGAGTGACCCTGGGCTGGCCCGAATACGATTGGACTCCA............GACAACCTT...  3651
Lepisosteus oculatus           AGGAGAACAAGAAAAATGGAGACATTTTGGGATGGCCCGAGTACGACTGGGTCCCAGTTTCAGGACCGGAGAAAGGG...  3663
Macaca fascicularis            CGGAGAATGAGCTGCACGGGGAGAAGCTGGGCTGGCCGCAGTACAACTGGACACCGAACTCCGCTCGCTCAGAAGGG...  3708
Manacus vitellinus             AGGAGAATGAGAAGAACGGGGAGGTCCTGGGCTGGCCCGAGTACAGCTGGACTCCTGACTCAGCCACTCTGGTC......  3657
Mus musculus                   CCGAGAACAGGAGACATGGAGAGACACTGGGCTGGCCGGAGTACAACTGGGCGCCAAACACCGCTCGCGCAGAAGGC...  3720
Neolamprologus brichardi       AGGAGAACAGCAAGAACAATGATGTTCGCGGATGGCCAGATTACGACTGGAAGCCTTTTAGTGAA...TCTCCG......  3672
Oncorhynchus mykiss            TAGAGAACAACAAGAATGGCGAGGTCAGAGGCTGGCCAGAATATGACTGGAAACCACCAAGTGACGGCTCAGAAACATCT  3678
Oreochromis niloticus          AGGAGAACAGCAAGAACAATGATGTTCGCGGATGGCCAGATTACGACTGGAAGCCTTTTAGTGAA...TCTTCA......  3672
Poecilia formosa               AGCAGAACAATAAGAACAACGAGGTCCGCGGGTGGCCCGACTACACCTGGAGACCTTCAGATATAGTTGAAGTTGCA...  3684
Poecilia latipinna             AGCAGAACAATAAGAACAACGAGGTCCGTGGGTGGCCCGACTACAACTGGAGACCTTCAGGTATTTTGAAGTTGCA...  3684
Pseudopodoces humilis          AAGAGAACAAGAAGAACGGGGAGGTCCTGGGCTGGCCCGAGTACAGCTGGACTCCTTACACAGCC......CAA......  3651
Pundamilia nyererei            AGGAGAACAGCAAGAACAATGATGTTCGCGGGTGGCCAGATTACGACTGGAAGCCTTTTAGTGAA...TCTCCA......  3672
Pygocentrus nattereri          AGGAAAACAAAAATAATGGCGATATCCTCGGATGGCCTGAATACAGCTGGATGCCGTCCACAACTGAGAGT.........  3651
Python bivittatus              AAAAGAACTTGCAAAATAATGAAACTCTGGGCTGGCCTGAATACACCTGGAAGCCATATACAGGT...............  3660
Rattus norvegicus              CAGAGAACAGGAGACATGGAGAGACACTGGGCTGGCCGGAGTACACCTGGACACCAAACACGGCTCGTGCAGAAGGC...  3723
Scleropages formosus           CAGAAAACAACAAAAACAATGATGTCCGTGGCTGGCCTGAGTATAACTGGAAACCATTCGCTCAAAGCACAGAA......  3660
Sinocyclocheilus anshuiensis   AGGAGAACAAAAAGAATGGTGATGTCCTGGGATGGCCAGAGTACGATTGGACGCCTTACAAAATCACTTCA.........  3654
Xenopus tropicalis             AGGAGAATACAAAGAATGGCGAAACCTTAGGATGGCCAGAATACAACTGGAGCCCAGTACAATCCGTTCCTCTGCCA...  3669
Lipotes vexillifer             CCGAGAACAGGCGGCACGGGGAGAAGCTGGGCTGGCCTCAGTACAACTGGATGCCAAACTCAGCTCGCTCGGAAGGC...  3693
Orcinus orca                   CCGAGAACACGCGGCACGGGGAGAAGCTGGGCTGGCCTCAGTACAACTGGATGCCAAACTCAGCTCGCTCGGAAGCC...  3714
Homo sapiens                   CGGAGAACGAGCTGCATGGGGAGAAGCTGGGCTGGCCGCAGTACAACTGGACGCCGAACTCCGCTCGCTCAGAAGGG...  3705
```

```
                                                   3690      3700      3710      3720      3730      3740
conservation
Callorhinchus milii               ATT...............CCAACTTCAACACCAGAAGAGGCGAAAGATAAGGTGGATTTTCTGGGATTAACATTGGATCC  3749
Alligator mississippiensis        .......................................ACAGAGTCTACAGTCAATTTTCTGGGGATGTCTCTGGATCG  3701
Anolis carolinensis               .................................GTGCAAGCAGACAACAAAGTCAATTTCCTTGGGATGTTGATGACCAG  3779
Astyanax mexicanus                .....................ACTGTAGTGGTAGTGGAGAAGGACAGCCCCGTGAACTTTCTGGGTTTGAGTGTGGATGA  3728
Boleophthalmus pectinirostris     .......................................GAAAAGAGTAAAGTGGACTTCCTCGGCATGAATCTGGACAG  3713
Cynoglossus semilaevis            .................................GTGGTTGAATCCAATAAAGTGGACTTCCTGGGTATGAGTGTGGATAA  3725
Danio rerio                       ...........................GTGGTGGAGGAGTCGCCCAAATCTGTGAATTTCCTGGGGTTGAGTGTGGATGC  3836
Esox lucius                       CCACTCACCACAGTCCCACATGATACCAGTCCAACAATTCCACCACATGCTGTGGACTTCCTGGGGATGAAACTGGACCA  3758
Fundulus heteroclitus             .................................ATT......GATAGTAAAGTTGAGTTCCTGGGACTGAAGGTGGACCA  3728
Gekko japonicus                   .................................GCGCAAGAGAGCAGCAGAGTCCAGTTCCTGGGCATGTCTTTGGACAG  3719
Haplochromis burtoni              .......................................GAAGCTAAAACAGTGGATTTCCTGGGTTTAAATGTGAACAG  3704
Hippocampus comes                 .................................GGGGTGGAGACCAGCACGGTGGACTTCCTGGGAATGAACGTGGACAG  3704
Ictalurus punctatus               ........................GTGGAGGAGGTCAAC...CCTACTACTGTGGATTTCTTAGGGTTAAGTGTGGATGA  3722
Larimichthys crocea               .................................CCAATTAAGGATAATAAAGTGGACTTCCTTGGCATGAATGTGGATGG  3716
Lates calcarifer                  ....................................GGTAAAGCTGGTAAAGTGGACTTCCTGGGTATGAATGTGGACAG  3707
Latimeria chalumnae               ..................CAGACAGCTTCTCCCAACAATAATGGTGACAAAGTGGATTTCCTTGGCATGACACTAAACAG  3713
Lepisosteus oculatus              ........................CCAGAGAAAGTGACCGGATCTGCGGAGGTGAGCTTCCTGGGCATGAACCTGACCAG  3719
Macaca fascicularis               ..............................CCCCTCCCAGACAGTGGCCGCGTCAGCTTCCTGGGCCTGGACCTAGATGC  3758
Manacus vitellinus                .................................CCAGATGGCTCCAGCAAAACTGATTTCCTGGGAATGTCCCTGACCAA  3704
Mus musculus                      ..............................TCCACCGCAGAGTCCAACCGCGTCAATTTCCTGGGCCTGTACCTGGAGCC  3770
Neolamprologus brichardi          .................................ACAGGTGAAGCTAAAACAGTGGATTTCCTGGGTTTAAATGTGAACAG  3719
Oncorhynchus mykiss               CCCCCAGCCCCAATCCCA...............GTTCTAGATAATGGTAAAGTTAACTTTCTGGGCATGAGTCTGGATGG  3743
Oreochromis niloticus             .................................ACAGGTGAAGCTAAAACTGTGGATTTCCTGGGTTTAAATGTGAACAG  3719
Poecilia formosa                  .................................ACGAAAGATGATGAGAAAGTTGAGTTCCTGGGACTGAAAGTGGACAA  3731
Poecilia latipinna                .................................ACGAATGATGATGAGAAAGTTGAGTTCCTGGGACTGAAAGTGGACAA  3731
Pseudopodoces humilis             .................................GATGACTCCAGC...AAAACTGATTTCCTGGGAATGTTCCTGACCAA  3695
Pundamilia nyererei               .................................ACAGGTGAAGCTAAAACAGTGGATTTCCTGGGTTTAAATGTGAACAG  3719
Pygocentrus nattereri             .....................ACTGCAGCGGTGGTGGAG...GACAGCTCAGTAGACTTTTTGGGCTTGAATATAGACCA  3707
Python bivittatus                 ..........................................GAATCAGATGTCAGTTTCCTTGGGATGTCGCTGAACCG  3698
Rattus norvegicus                 ..............................TCCCTCCCAGAGTCCAGTCGCGTCAACTTCCTGGGTATGTACCTGGAACC  3773
Scleropages formosus              ..........................................GAGAAAAAAGTGGACTTCCTTGGATTAAGTGTAGACAG  3698
Sinocyclocheilus anshuiensis      ...........................GTGATGGACGAGAAGCCAAAAGCAATCAGTTTCCTGGGTTTGAGTGTGGATGA  3707
Xenopus tropicalis                .................................CCGTCTGGAGATTCTAACTCTGACTTCTTGGGACTGGCGGTTAGCAA  3716
Lipotes vexillifer                ..............................TCCTTCCTGGGCTCCGGCCGTGTCAGCTTCCTGGGCCTGAACCTGGAGGA  3743
Orcinus orca                      ..............................TCCTTCCCGGGCTCCGGCCGTGTCAGCTTCCTGGGCCTGAACCTGGAGGA  3764
Homo sapiens                      ..............................CCCCTCCCAGACAGCGGCCGCGTCAGCTTCCTGGGCCTGGACCTGGATGC  3755
```

```
                                  3750      3760      3770      3780      3790      3800      3810      3820
conservation
Callorhinchus milii               CCAGCAGGCAAAAGCAGGACAGTGGATTCTGCTTTCAGTCGGTATTGCCCTAGTCTTTTTAGTTTTGTTTGTGGTAATGA  3829
Alligator mississippiensis        CAATCAAGCCACTGCTGGCCAGTGGGTCCTGCTTGTCCTGGGACTGGTCCTGCTTCTGTCCACCATTGTCCTTGGAGTCA  3781
Anolis carolinensis               CAGCGAAGCCGCAGCAGGACAGTGGATTTTGCTTGTCCTTGGACTCCTCCTTCTTATTGCAGCAATAGTATTGGGGGCCA  3859
Astyanax mexicanus                AGCTGCAGCTACAGCTGGTCAGTGGGTTCTGCTGGTGCTGGGGATCGTCCTCCTCCTCGCCACCATCTTCCTGGCCTACA  3808
Boleophthalmus pectinirostris     CTCTTCAGCAGTTGCCGGACAGTGGATTCTGCTTGTCGCTGGTCTGGTCCTCCTTGTGGCCACAATCCTTTTAGCGTATA  3793
Cynoglossus semilaevis            AGCAGCTGCTGTAGCTGGACAGTGGATCCTTCTAGTGCTTGGCCTGGTTCTTCTGGTCGCCACCATCCTGGTTGCCTACA  3805
Danio rerio                       AGCGGGAGCAGCAGCGGGACAGTGGATCCTGCTGGTCCTCAGCATCGTCTTCCTGCTTGCCGTCGTCTTCTTAGCCTACA  3916
Esox lucius                       TTCAGCGGCGTCTGCGGGCCAGTGGGTCCTTCTGGTCCTGGGCCTGGTGCTCTTAGTGGCCACCATTCTCCTGGGCTACA  3838
Fundulus heteroclitus             GGAGGCTGCCAAAGTTGGCCAGTGGCTGCTGCTGTCAATCAGCCTGGCTTTCTTGGTGGTCATCATCCAGCTCGCCTACA  3808
Gekko japonicus                   CAACGAAGCAGCAGTGGGCCTGTGGACCTTGCTGGGCCTTGGGCTGCTCCTCTTCATTGCCGCGCTAGTCTTGGGGGCCA  3799
Haplochromis burtoni              GTCGGCTGCCACAGTCGGCCAGTGGATCCTGTTGGCCCTCGGCATGCTGCTATTGGTGGTCACCATCCTTATGGCCTACA  3784
Hippocampus comes                 TGCGGCCGCCATTGCGGGCCAGTGGGTCCTGCTGGTGCTGGGCCTGGTCCTGTTGGTTGCCACCATCTTCCTGGCGTACA  3784
Ictalurus punctatus               AGCTGCAGCTGTAGCTGGGCAGTGGGTTCTCCTTGTGCTGGGGATTGTTCTCCTGGTCTCCACAGCCTTCTTGGCCTACA  3802
Larimichthys crocea               CTCAGCTGCCACAGCTGGCCAGTGGATACTGTTGGTCCTTGGCATAGTCCTCTTGGTGGCCACCATCCTATTGGGCTACA  3796
Lates calcarifer                  CGCAGCTGCCATAGCTGGCCAGTGGGTCCTATTGGTCATTGGCCTGGTCCTCTTGGTGGCCACCATCCTATTGGCCTACA  3787
Latimeria chalumnae               CAGTCAGGCTGCAGCTGGACAGTGGATCCTACTGGTCATTGGACTTGGCCTTGTCATTGCCACCATTGTCTTAGCAGTCA  3793
Lepisosteus oculatus              CGATCAGGCAGCGGCCGGCCAGTGGATTCTGCTGGTCCTGGGGCTGGGACTACTCATTGCCACCATCTGCCTGGCCTACA  3799
Macaca fascicularis               GCAGCAGGCCCGCGTGGGCCAGTGGCTGCTGCTCTTCCTGGGCATCGCCCTGCTGGTAGCCACCCTGGGCCTCAGCCAGC  3838
Manacus vitellinus                AAGCCAAGCCTCGGTAGGCGCCTGGGTCCTGCTCGCCCTGGCGCTCGTCTTCCTGATCACCACCATCTTCTTCAGCGTCA  3784
Mus musculus                      ACAGCAGGCCCGCGTGGGCCAGTGGGTGCTGCTCTTCCTGGGCGTCGCCCTGCTCGTGGCCACCGTGGGTCTCGCCCATC  3850
Neolamprologus brichardi          GTCGGCTGCCATAGCCGGCCAGTGGATCCTGTTGGCCCTCGGCGTGCTGCTATTGGTGGTCACCATCCTTATGGCCTACA  3799
Oncorhynchus mykiss               TGCAGCGGCTGCAGCAGGCCAGTGGGTCCTACTGGTCCTGGGCCTGGTGTTCCTAGTGGCCACCATCCTCCTGGGCTACA  3823
Oreochromis niloticus             GTCGGCTGCCATAGCCGGCCAGTGGATCCTGTTGGCCCTCGGCGTGCTGCTCTTGGTGGCCACCGTCCTTACGGCCTACA  3799
Poecilia formosa                  GGTCGCTGCCAAAGCTGGTCAGTGGTTACTGCTATCAATCAGCCTGGCTTTCCTGGTGGTCATCATCCAACTCGCCTACA  3811
Poecilia latipinna                GGACGCTGCCATAGCTGGTCAGTGGTTACTGCTATCACTCAGCGTGGCTTTCCTGGTGGTCATCATCCAACTCGCCTACA  3811
Pseudopodoces humilis             AAGTCAAGCCACAGCAGGTGGCTGGGTCCTGCTCGCCCTGGCACTCATCTTCCTGATCACCACCATCTTCTTGGGTGTCA  3775
Pundamilia nyererei               GTCGGCTGCCACAGTCGGCCAGTGGATCCTGTTGGCCCTCGGCATGCTGCTATTGGTGGTCACCATCCTTATGGCCTACA  3799
Pygocentrus nattereri             AGCTGCAGCTACAGCTGGCCAGTGGGTCCTCCTGGTACTCGGTATCGTTCTCCTGCTCGCTACCATCTTCTTGGCCTACA  3787
Python bivittatus                 CAGTCAAATAGAAGCAGGACAGTGGATCTTGCTTGCAATTGGGCTTATCCTTCTTATTACCACAATAGTCTTGGGTGTCA  3778
Rattus norvegicus                 ACAGCAGGCCCGTGTGGGCCAGTGGGTGCTGCTCTTCCTAGGCGTCGCCCTGCTGGTGGCCACCGTGGGTCTCGCCCACC  3853
Scleropages formosus              TGCTGCAGCTGTGGCAGGCCAGTGGGTCCTACTGGTGCTGGGATTGGTGTTGCTGCTTGCAACCATCTTTATGGGCTATA  3778
Sinocyclocheilus anshuiensis      GGGAGGCGCTGTGGCGGGACAGTGGATTCTCTTGGTTCTGAGCATCGTCTTCCTGCTTGGCATCATCTATTTAGTCTACA  3787
Xenopus tropicalis                TAGCCAAGCAGCTGCTGGACAATGGATTCTCCTGGCTTTGGGCATTGTCCTCATCATCACCACCATTGTCTTTGGTGTGC  3796
Lipotes vexillifer                GCAGCAGGCCCGCGTGGGCCAGTGGGTGCTGCTCTTCCTGGGCGTCGCCCTGCTGGTGGCCACCCTGGGCCTCGCCCACC  3823
Orcinus orca                      GCAGCAGGCCCGCGTGGGCCAGTGGGTGCTGCTTTTCCTGGGCGTCGCCTTGCTGGTGGCCACCCTGGGCCTCGCCTACC  3844
Homo sapiens                      GCAGCAGGCCCGCGTGGGCCAGTGGCTGCTGCTCTTCCTGGGCATCGCCCTGCTGGTAGCCACCCTGGGCCTCAGCCAGC  3835
```

```
                               3830          3840          3850          3860          3870                               3880
conservation
Callorhinchus milii            AATTTCTTTCAAACAAAAGAAGCCCAAAAAAGTCAACATCT........................GAACTCGAACTAAAA  3885
Alligator mississippiensis     AATGCTACTCTGCAAGGCGGAGGGCCTACAAATCCGGCTCA........................GAAATGGAACTGAAA  3837
Anolis carolinensis            AATGGCACCAGGCCAGGAGAAGAGCCACCAGATCAAGCATG........................GAAATGGAGTTGAAA  3915
Astyanax mexicanus             GTTACAGAAAATCCAGACGCCTCCAGAGCAAATCTATCTCT........................CAGATGGAGCTGAAA  3864
Boleophthalmus pectinirostris  AGTACAGGAAGTCAAAGAAA...CCTGAGAAGTCCATATCC........................ACTCTGGAGCTCAAG  3846
Cynoglossus semilaevis         AGTACAAGAAAGCAAAAAAG...TCCAGGAAGAGCTTGTCC........................TCCATGGAGCTCAAG  3858
Danio rerio                    GATACAGCAAGACTAAACGCCTACAGAACAAATCCATGTCT........................CAGATGGAGCTCAAG  3972
Esox lucius                    AGTACAAGAAATCCAAACGG...AGAAGTCTGTCCTCATCT........................GAGGTGGAGCTTAAG  3891
Fundulus heteroclitus          AGTACAGGAAGTCAAAG......CGTGGAAAGTCCCCGTCG........................ATGATGGAGCTCAAG  3858
Gekko japonicus                GATTCCTCTCAGACAGGAGCAAGATCTTCAGTTCTAACTCT........................GAAATGGAGTTGAAA  3855
Haplochromis burtoni           TGTACAGGAAGGCAAGAAAG...CCTGAAAAGTCCTTGTCT........................ACTATGGAGCTGAAG  3837
Hippocampus comes              GATACAGGAAGTCAAAGAGG...TCCAACATGTCGTTATCA........................ACGATAGAGCTCAAG  3837
Ictalurus punctatus            AATACCGGAAATCCAGACGCCTACACAAGAAGTCCATCTCT........................CAAATGGAGCTCAAA  3858
Larimichthys crocea            AGTACAGGAAGACAAAAAAG...CGTGAAAAGTCCTCATCC........................ATGATGGAGCTTAAG  3849
Lates calcarifer               AGTATAGGAAATCAAGGAAG...CCTCAAAAGTCCCTCTCC........................ACAATGGAGCTCAAG  3840
Latimeria chalumnae            AGTTCACCTCTATGAAACGACAAACCAAAAAGTCCTCTTCA........................GAACTGGAACTGAAA  3849
Lepisosteus oculatus           AATCCAAGTCCAAAAAGAGA......CAGAAATCCAACTCG........................GAGCTGGAGCTGAAA  3849
Macaca fascicularis            GGCTCTTCAGCATCCGC...CACCAAAGCCTCCACCAGCACCCCCAGGGGCCCCAGTTCGGCTCCGAGGTGGAGCTGAGA  3915
Manacus vitellinus             AGTTCTTCTCGTCCAGGAGAAAGGCCTTCAAATCCAGCTCA........................GAAATGGAACTGAAA  3840
Mus musculus                   GGCTCTACAACATCCGTAACCATCACAGCCTCCGCCGGCCCCACCGTGGGCCCCAGTTTGGGTCTGAGGTGGAGCTCAGA  3930
Neolamprologus brichardi       TGTACAGGAAGGCAAGAAAG...CCTGAAAAGTCCTTGTCT........................ACTATGGAGCTGAAG  3852
Oncorhynchus mykiss            AGTACAAGAAATCAAAACGA...AGAAATCTGTCCTCATCT........................GAGCTGGAGCTGAAG  3876
Oreochromis niloticus          TGTACAGGAAGGCAAGAAAG...CCGGAAAAGTCCCTGTCT........................ACTATGGAGCTGAAG  3852
Poecilia formosa               GGTACAGGAAGTCCAAGAAG...CGTAATAAGTCCTCATCC........................ATGATGGAGCTCAAG  3864
Poecilia latipinna             GGTACAGGAAGTCCAAGAAG...CGTAATAAGTCCTCATCC........................ATGATGGAGCTCAAG  3864
Pseudopodoces humilis          AGTTCTTCTCAGCCAGGAGAAAGGCCTTCAAATCCAGCTCA........................GAAATGGAACTGAAA  3831
Pundamilia nyererei            TGTACAGGAAGGCAAGAAAG...CCTGAAAAGTCCTTGTCT........................ACTATGGAGCTGAAG  3852
Pygocentrus nattereri          AATACAGAAGATCCAGACACCTACAGAACAAATCCATCTCT........................CAAGTGGAGCTCAAA  3843
Python bivittatus              AGTACTTCATAACAAGGAGGAAAGGCCTCAGATCTAACTCA........................GAAATGGAACTGAAA  3834
Rattus norvegicus              GACTCTACAACATCCATAACCATCACAGCCTCCGCCGGCCCCACCGTGGGCCCCAGTTTGGGTCCGAGGTGGAGCTCAGA  3933
Scleropages formosus           AGAACTATAAAGCAAAGAAG...TTGGCAAAGTCCTCCTCT........................ATAATTAAGCTAGAT  3831
Sinocyclocheilus anshuiensis   GATACAGGAAGACTAAACGCTTACAGGACAAATCAATATCT........................CAGATGGAGCTCAAG  3843
Xenopus tropicalis             TATACTCAAAAATGAAAAGCAGAGCTAAGATGTCCAGCTCT........................CAGATGGAGCTGAAA  3852
Lipotes vexillifer             GGCTCTTCAGCATCCGC...CACCACAGCCTCCACCAGCCCCACCGCGGGCCCCAGTTTGGCTCCGAGGTGGAGCTCAGA  3900
Orcinus orca                   GGCTCTTCAGCATCCGC...CACCACAGCTTCCACCACCCCACAGCGGGCCCCAGTTTGGCTCCGAGGTGGAGCTCAGA  3921
Homo sapiens                   GGCTCTTCAGCATCCGC...CACCGCAGCCTCCACCGGCACTCCCACGGGCCCCAGTTCGGCTCCGAGGTGGAGCTGAGA  3912
```

```
conservation
Callorhinchus milii            TAA  3888
Alligator mississippiensis     TAA  3840
Anolis carolinensis            TAA  3918
Astyanax mexicanus             TAA  3867
Boleophthalmus pectinirostris  TCC  3849
Cynoglossus semilaevis         TAA  3861
Danio rerio                    TAA  3975
Esox lucius                    TAA  3894
Fundulus heteroclitus          CAA  3861
Gekko japonicus                TAG  3858
Haplochromis burtoni           CAA  3840
Hippocampus comes              CAA  3840
Ictalurus punctatus            TAG  3861
Larimichthys crocea            TAA  3852
Lates calcarifer               CAA  3843
Latimeria chalumnae            TAA  3852
Lepisosteus oculatus           TAG  3852
Macaca fascicularis            CAC  3918
Manacus vitellinus             TGA  3843
Mus musculus                   CAC  3933
Neolamprologus brichardi       CAA  3855
Oncorhynchus mykiss            TAA  3879
Oreochromis niloticus          CAA  3855
Poecilia formosa               TAA  3867
Poecilia latipinna             TAA  3867
Pseudopodoces humilis          TAA  3834
Pundamilia nyererei            CAA  3855
Pygocentrus nattereri          TAG  3846
Python bivittatus              TGA  3837
Rattus norvegicus              CAC  3936
Scleropages formosus           TCC  3834
Sinocyclocheilus anshuiensis   TAA  3846
Xenopus tropicalis             TAG  3855
Lipotes vexillifer             CAC  3903
Orcinus orca                   CAC  3924
Homo sapiens                   CAC  3915
```

X non conserved
X $\geq$ 60% conserved
X $\geq$ 85% conserved
