# Supplementary material for: A Genomic Survey of Angiotensin-Converting Enzymes Provides Novel Insights into Their Molecular Evolution in Vertebrates
Source: Molecules. 2018 Nov 9;23(11):2923. doi: 10.3390/molecules23112923 (PMC6278350; doi:10.3390/molecules23112923)
Supplement: Supplementary file 1 [file molecules-23-02923-s001.zip › Figure S3.pdf]

| conservation | | |
|---|---|---|
| Callorhinchus milii | ATG................................................TTCCTCCAG.................... | 12 |
| Alligator mississippiensis | ATG..................................................................TTGGGCTACAT | 14 |
| Anolis carolinensis | ATG.......................................GCATCTCTTAGCTCAAGTGGAAGTTTGCTGTTCCAGCT | 41 |
| Astyanax mexicanus | ATG........................................................................TCAGT | 8 |
| Boleophthalmus pectinirostris | ATG..................................................................AACCACGCTCT | 14 |
| Cynoglossus semilaevis | ATG.....................................................................TCTGCACA | 11 |
| Danio rerio | ATG........................................................................TGTGC | 8 |
| Esox lucius | ATG..................................................................TCTATGAAG.. | 12 |
| Fundulus heteroclitus | ATG.....................................................................CCTGCCGG | 11 |
| Gekko japonicus | ATG..................................................................CGGAGGCATCT | 14 |
| Haplochromis burtoni | ATG.....................................................................TCTCCGAG | 11 |
| Hippocampus comes | ATG.....................................................................TCAGGAAA | 11 |
| Ictalurus punctatus | ATG........................................................................TCTGT | 8 |
| Larimichthys crocea | ATG.....................................................................TCTGTGAG | 11 |
| Lates calcarifer | ATG.....................................................................TCTGTTAG | 11 |
| Latimeria chalumnae | ATGCGCGTGACTCTGGACAAAAAAACAAAATCGTGGCGACAATGCGACTTCTACTTCTCAAATACTGGTGGGGCTAGGCC | 80 |
| Lepisosteus oculatus | ATG........................................................................TCTGT | 8 |
| Macaca fascicularis | ATG..................................................................TCAGGCTCTTC | 14 |
| Manacus vitellinus | ATG............................................................AATATGTTGGTCCACCT | 20 |
| Mus musculus | ATG..................................................................TCCAGCTCCTC | 14 |
| Neolamprologus brichardi | ATG.....................................................................TCTCCGAG | 11 |
| Oncorhynchus mykiss | ATG..................................................................TCTATGACCAG | 14 |
| Oreochromis niloticus | ATG.....................................................................TCTCCGAG | 11 |
| Poecilia formosa | ATG.....................................................................TCCGCCGG | 11 |
| Poecilia latipinna | ATG.....................................................................TCCGCCGG | 11 |
| Pseudopodoces humilis | ATG..................................................................TTGGTCCACTT | 14 |
| Pundamilia nyererei | ATG.....................................................................TCTCCGAG | 11 |
| Pygocentrus nattereri | ATG........................................................................TCTGG | 8 |
| Python bivittatus | ATG..................................................................TTACCCTGGCT | 14 |
| Rattus norvegicus | ATG..................................................................TCAAGCTCCTG | 14 |
| Scleropages formosus | ATG........................................................................CATTC | 8 |
| Sinocyclocheilus anshuiensis | ATG........................................................................CTTGC | 8 |
| Xenopus tropicalis | ATG..................................................................CCGGGTCTGCT | 14 |
| Lipotes vexillifer | ATG..................................................................TCAGGCTCTTT | 14 |
| Orcinus orca | ATG..................................................................TCAGGCTCTTT | 14 |
| Homo sapiens | ATG..................................................................TCAAGCTCTTC | 14 |

| conservation | 20 30 40 50 60 | |
|---|---|---|
| Callorhinchus milii | ....TGGCTTTTGCTGCTGAGCCTGGCAGCAGCTGCTCTTTCTCTGTCACCT............................ | 60 |
| Alligator mississippiensis | TTGGGTTTTTTGTGGT......CTGTTTGCAGTTGCTGTGCCACAG.................................. | 54 |
| Anolis carolinensis | CTATCTTATTTGGAAC......TTGGTTCTTCTGGCTGTAGCACAG.................................. | 81 |
| Astyanax mexicanus | TCGCTTTCTCCTGGTTTTGGCCTTGGCCTCCGGGGCCTGGGCTCAGACG............................... | 57 |
| Boleophthalmus pectinirostris | AGCTCTTACTCTCCTGCTGGGCCTCTGCTGCTCGGCCTGGGCCCAGACTGAG............................ | 66 |
| Cynoglossus semilaevis | GATTCTGTTAGCGTTGCTGGCTCTGTCCTGCTGCGTCTCTGCTCAA.................................. | 57 |
| Danio rerio | TCGCTGGCTCCTGCTTTTGGCCTTGGCCTCTGTGGCCTGCTGTCAGACT............................... | 57 |
| Esox lucius | .TTTCTTCTCGTGGGATGGACCGTTGTGGTCTGTGTGGTGGGACAGTCGGAA............................ | 63 |
| Fundulus heteroclitus | GACTCTCGTAAGGCTTCTGGCCGTGTTCTCGGCTGTTTCCCTGGTGGCTGCGGTG......................... | 66 |
| Gekko japonicus | CTGTCTTCTTTGGAAT......TTGGCTCTTCTGGCTGCAGCCCAG.................................. | 54 |
| Haplochromis burtoni | GATTCTTGTAACGCTCCTGGCCGTGTCCTGTGCTGTTTCTGCTCAGTCAGAC............................ | 63 |
| Hippocampus comes | GATTTTCCTTTGCGCGCTGGCTTTCTGTTTTTCTCTTTCTTCTCAGTCGCTA............................ | 63 |
| Ictalurus punctatus | TCTGTGCGTCTTGGTTTTGCTTACGGCTTCTGCGGCCTGGGCTCAGACA............................... | 57 |
| Larimichthys crocea | GATTCTTTTATTGTTGCTGGCAGTGTCCTCTGCTGTTTCTGCTCAGTCAAGC............................ | 63 |
| Lates calcarifer | GATTCTTGTAACATTGCTGGTTGTGTCCTGCGCTGTCTTTGCCCAGTCTGAC............................ | 63 |
| Latimeria chalumnae | TGCTTTCCAGATATTGCTGGAATATAACCCGGCGTCCCTGGGTGGTGTGACAACCCTTGATGAATTCTTTGTCTTAAGTG | 160 |
| Lepisosteus oculatus | CCATCTTCTGTTAGTGTTGATTGTGGCAGTTGCTGCTGCTGCCCAGTTAACT............................ | 60 |
| Macaca fascicularis | CTGGCTCCTTCTCAGC......CTTGTTGCTGTAACTGCTGCTCAG.................................. | 54 |
| Manacus vitellinus | CTGGCTTCTCTGTGGT......CTGATCACTGCTGTGACCCCTCAA.................................. | 60 |
| Mus musculus | CTGGCTCCTTCTCAGC......CTTGTTGCTGTTACTACTGCTCAG.................................. | 54 |
| Neolamprologus brichardi | GATTCTTGTAACGCTCCTGGCCGTGTCCTGTGCTGTTTCTGCTCAGTCAGAC............................ | 63 |
| Oncorhynchus mykiss | GCTTCTTCTAGTGGGGCTGATTGCTGTGGCCTGTGTGGCAGCACAGTCAGAC............................ | 66 |
| Oreochromis niloticus | GATTCTTGTAACGCTTCTGGCTGTGTCCTGTGCTGTTTCTGCTCAGTCAGAC............................ | 63 |
| Poecilia formosa | GATTCTTGTAGTGCTTCTGGCCGTGTCTTCGGCTGTTTCCCTCGTGACGGCC............................ | 63 |
| Poecilia latipinna | GATTCTTGTAGTGCTTCTGGCCGTGTCTTCGGCTGTTTCCCTCGTGACGGCC............................ | 63 |
| Pseudopodoces humilis | CTGGCTTCTCTGTGGC......CTGAGTGCTGTTGTGGCCCCTCAG.................................. | 54 |
| Pundamilia nyererei | GATCCTTGTAACGCTCCTGGCCGTGTCCTGTGCTGTTTCTGCTCAGTCAGAC............................ | 63 |
| Pygocentrus nattereri | ACGCTTTCTCTTGATTTTGGCCTTGGCCTCCACAGCCTGGGCTCAGACA............................... | 57 |
| Python bivittatus | CTGCCTTATTTGGAGC......CTGGTGGTTCTGGCTGTGGCCCAG.................................. | 54 |
| Rattus norvegicus | CTGGCTCCTTCTCAGC......CTTGTTGCTGTTGCTACTGCTCAG.................................. | 54 |
| Scleropages formosus | TAAGCTTCTTGCACTTCTAGCTTGGGCCTTTGTGGCCTCCGTGCTTAGTCAGTCC......................... | 63 |
| Sinocyclocheilus anshuiensis | TCGCTGGCTCCTGCTTTTGGCCATGGCCTCTGTGGACTGCTCTCAGACC............................... | 57 |
| Xenopus tropicalis | TTGGCTTTTTTGTATTGGGCTACTCCTTGCCACTGGCAGGAGCCAA.................................. | 60 |
| Lipotes vexillifer | CTGGCTCCTTCTCAGC......CTTGTTGCTGTAACGGCTGCTCAA.................................. | 54 |
| Orcinus orca | CTGGCTCCTTCTCAGC......CTTGTTGCTGTAACGGCTGCTCAA.................................. | 54 |
| Homo sapiens | CTGGCTCCTTCTCAGC......CTTGTTGCTGTAACTGCTGCTCAG.................................. | 54 |

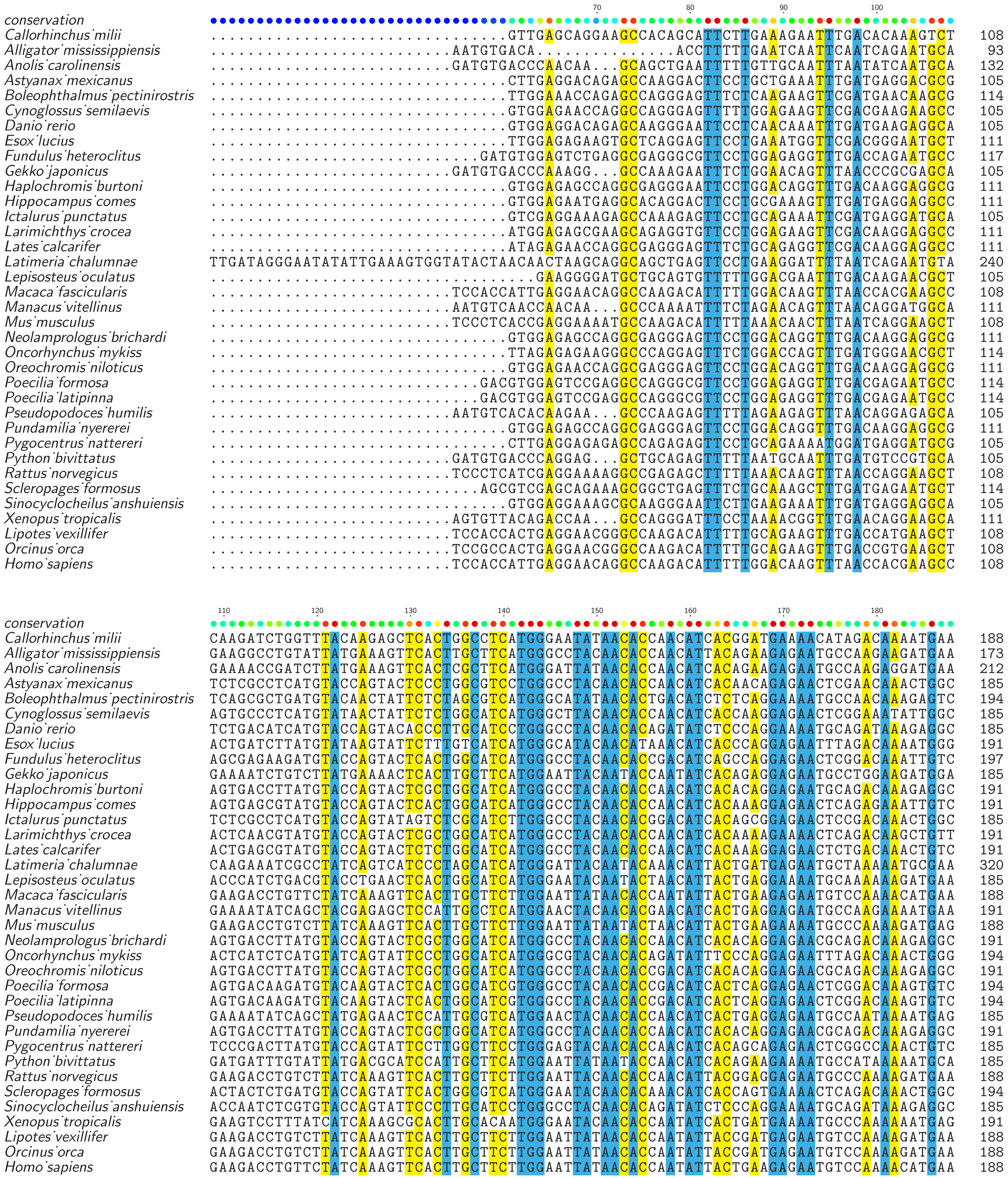

70
80
90
100
conservation
Callorhinchus milii 108
Alligator mississippiensis 93
Anolis carolinensis 132
Astyanax mexicanus 105
Boleophthalmus pectinirostris 114
Cynoglossus semilaevis 105
Danio rerio 105
Esox lucius 111
Fundulus heteroclitus 117
Gekko japonicus 105
Haplochromis burtoni 111
Hippocampus comes 111
Ictalurus punctatus 105
Larimichthys crocea 111
Lates calcarifer 111
Latimeria chalumnae 240
Lepisosteus oculatus 105
Macaca fascicularis 108
Manacus vitellinus 111
Mus musculus 108
Neolamprologus brichardi 111
Oncorhynchus mykiss 114
Oreochromis niloticus 111
Poecilia formosa 114
Poecilia latipinna 114
Pseudopodoces humilis 105
Pundamilia nyererei 111
Pygocentrus nattereri 105
Python bivittatus 105
Rattus norvegicus 108
Scleropages formosus 114
Sinocyclocheilus anshuiensis 105
Xenopus tropicalis 111
Lipotes vexillifer 108
Orcinus orca 108
Homo sapiens 108
110
120
130
140
150
160
170
180
conservation
Callorhinchus milii 188
Alligator mississippiensis 173
Anolis carolinensis 212
Astyanax mexicanus 185
Boleophthalmus pectinirostris 194
Cynoglossus semilaevis 185
Danio rerio 185
Esox lucius 191
Fundulus heteroclitus 197
Gekko japonicus 185
Haplochromis burtoni 191
Hippocampus comes 191
Ictalurus punctatus 185
Larimichthys crocea 191
Lates calcarifer 191
Latimeria chalumnae 320
Lepisosteus oculatus 185
Macaca fascicularis 188
Manacus vitellinus 191
Mus musculus 188
Neolamprologus brichardi 191
Oncorhynchus mykiss 194
Oreochromis niloticus 191
Poecilia formosa 194
Poecilia latipinna 194
Pseudopodoces humilis 185
Pundamilia nyererei 191
Pygocentrus nattereri 185
Python bivittatus 185
Rattus norvegicus 188
Scleropages formosus 194
Sinocyclocheilus anshuiensis 185
Xenopus tropicalis 191
Lipotes vexillifer 188
Orcinus orca 188
Homo sapiens 188

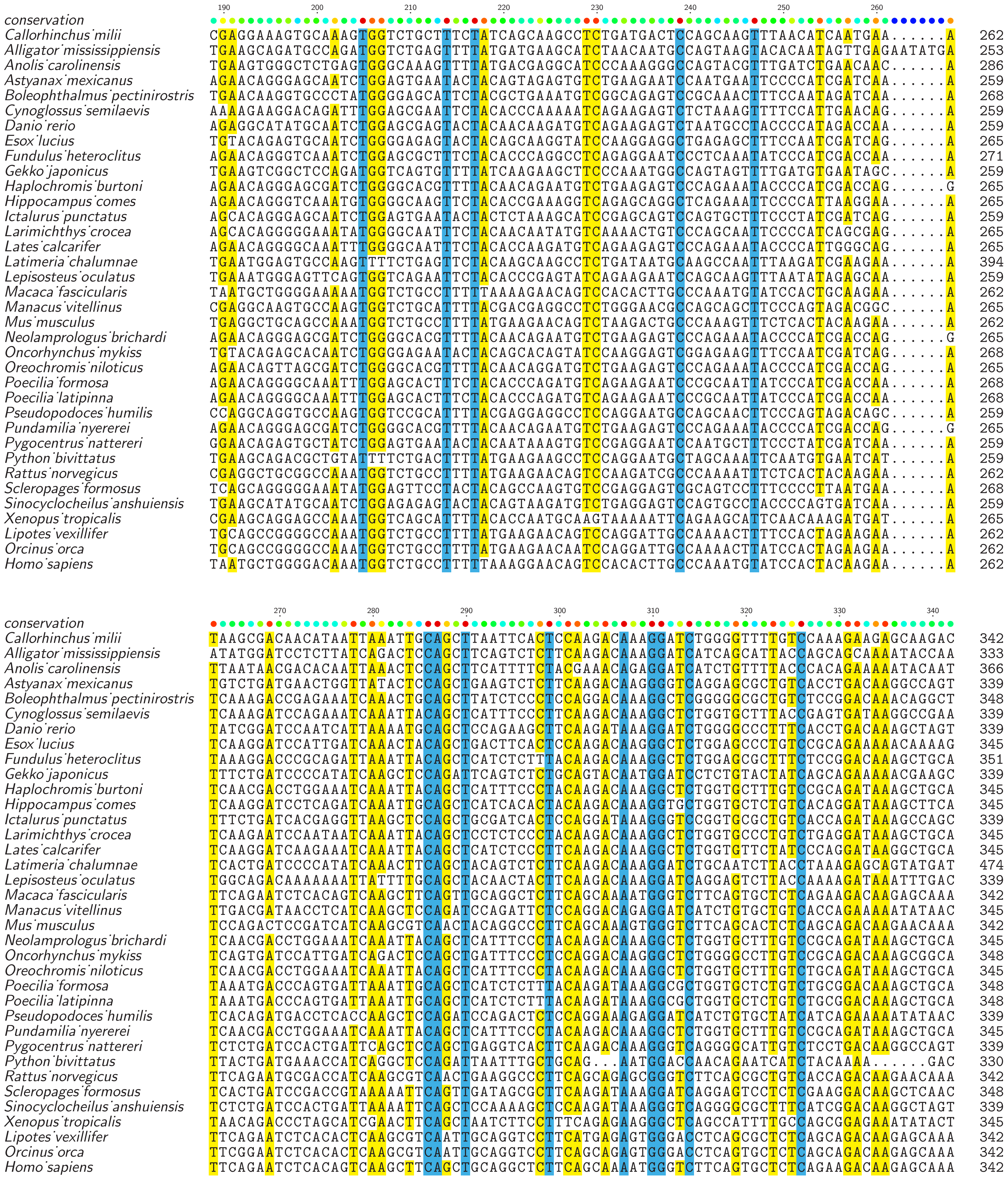
190
200
210
220
230
240
250
260
conservation
Callorhinchus milii 262
Alligator mississippiensis 253
Anolis carolinensis 286
Astyanax mexicanus 259
Boleophthalmus pectinirostris 268
Cynoglossus semilaevis 259
Danio rerio 259
Esox lucius 265
Fundulus heteroclitus 271
Gekko japonicus 259
Haplochromis burtoni 265
Hippocampus comes 265
Ictalurus punctatus 259
Larimichthys crocea 265
Lates calcarifer 265
Latimeria chalumnae 394
Lepisosteus oculatus 259
Macaca fascicularis 262
Manacus vitellinus 265
Mus musculus 262
Neolamprologus brichardi 265
Oncorhynchus mykiss 268
Oreochromis niloticus 265
Poecilia formosa 268
Poecilia latipinna 268
Pseudopodoces humilis 259
Pundamilia nyererei 265
Pygocentrus nattereri 259
Python bivittatus 259
Rattus norvegicus 262
Scleropages formosus 268
Sinocyclocheilus anshuiensis 259
Xenopus tropicalis 265
Lipotes vexillifer 262
Orcinus orca 262
Homo sapiens 262
270
280
290
300
310
320
330
340
conservation
Callorhinchus milii 342
Alligator mississippiensis 333
Anolis carolinensis 366
Astyanax mexicanus 339
Boleophthalmus pectinirostris 348
Cynoglossus semilaevis 339
Danio rerio 339
Esox lucius 345
Fundulus heteroclitus 351
Gekko japonicus 339
Haplochromis burtoni 345
Hippocampus comes 345
Ictalurus punctatus 339
Larimichthys crocea 345
Lates calcarifer 345
Latimeria chalumnae 474
Lepisosteus oculatus 339
Macaca fascicularis 342
Manacus vitellinus 345
Mus musculus 342
Neolamprologus brichardi 345
Oncorhynchus mykiss 348
Oreochromis niloticus 345
Poecilia formosa 348
Poecilia latipinna 348
Pseudopodoces humilis 339
Pundamilia nyererei 345
Pygocentrus nattereri 339
Python bivittatus 330
Rattus norvegicus 342
Scleropages formosus 348
Sinocyclocheilus anshuiensis 339
Xenopus tropicalis 345
Lipotes vexillifer 342
Orcinus orca 342
Homo sapiens 342

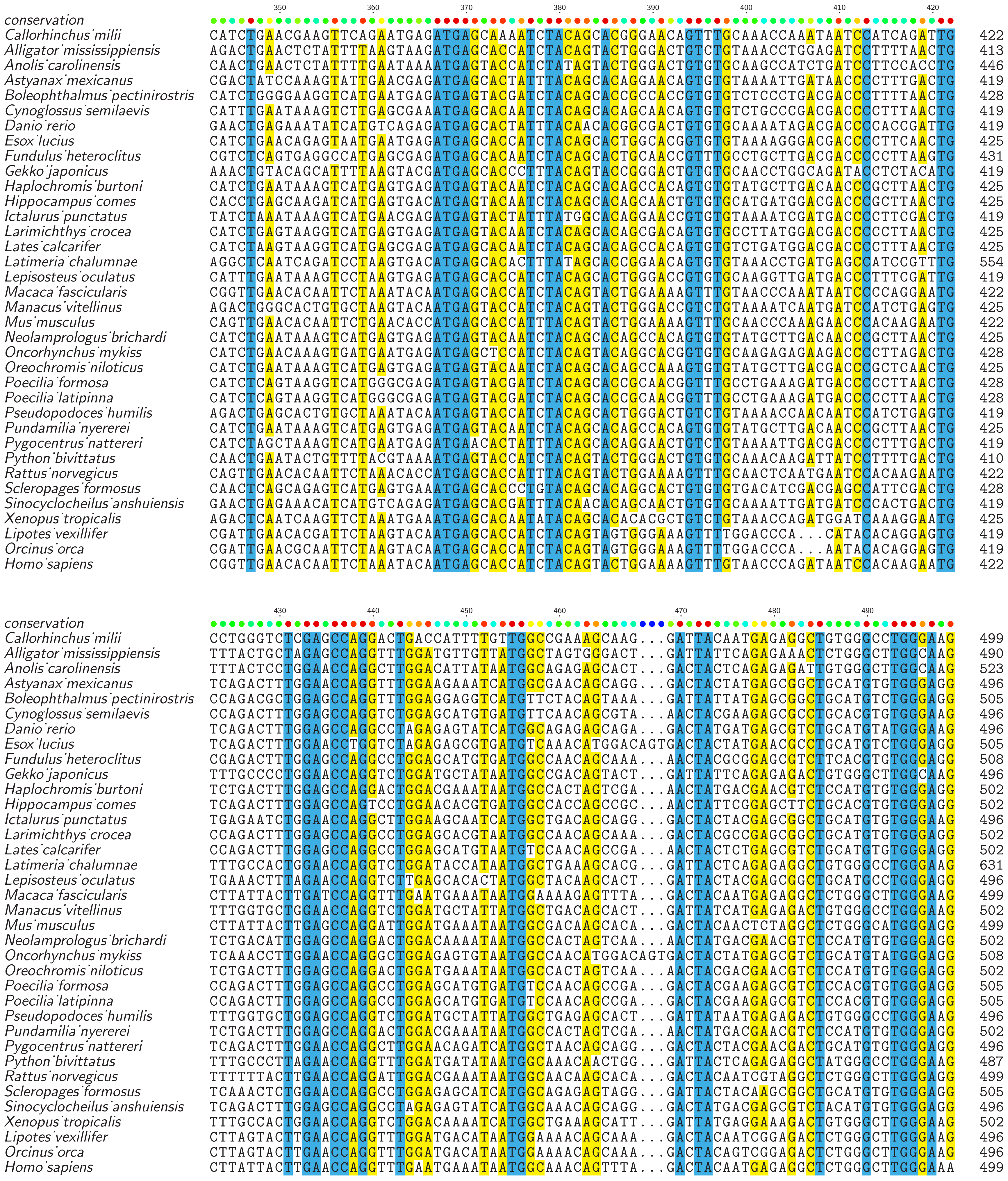
350 360 370 380 390 400 410 420
conservation
Callorhinchus milii CATCTGAACGAAGTTCAGAATGAGATGAGCAAAATCTACAGCACGGAACAGTTTGCAAACCAAATAATCCATCAGATTG 422
Alligator mississippiensis AGACTGAACTCTATTTTAAGTAAGATGAGCACCATCTACAGTACTGGGACTGTCTGTAAACCTGGAGATCCTTTTAACTG 413
Anolis carolinensis CAACTGAACTCTATTTTGAATAAAATGAGTACCATCTATAGTACTGGGACTGTGTGCAAGCCATCTGATCCTTCCACCTG 446
Astyanax mexicanus CGACTATCCAAAGTATTGAACGAGATGAGCACTATTTACAGCACAGGAACAGTGTGTAAAATTGATAACCCCTTTGACTG 419
Boleophthalmus pectinirostris CATCTGGGGAAGGTCATGAATGAGATGAGTACGATCTACAGCACCGCCACCGTGTGTCTCCCTGACGACCCTTTTAACTG 428
Cynoglossus semilaevis CATTTGAATAAAGTCTTGAGCGAAATGAGCACAATCTACAGCACAGCAACAGTGTGTCTGCCCGACGACCCCTTTAACTG 419
Danio rerio GAACTGAGAAATATCATGTCAGAGATGAGCACTATTTACAACACGGCGACTGTGTGCAAAATAGACGACCCCACCGATTG 419
Esox lucius CATCTGAACAGAGTAATGAATGAGATGAGCACCATCTACAGCACTGGCACGGTGTGTAAAAGGGACGACCCCTTCAACTG 425
Fundulus heteroclitus CGTCTCAGTGAGGCCATGAGCGAGATGAGCACAATCTACAGCACTGCAACCGTTTGCCTGCTTGACGACCCCCTTAAGTG 431
Gekko japonicus AAACTGTACAGCATTTTAAGTACGATGAGCACCCTTTACAGTACCGGGACTGTGTGCAACCTGGCAGATACCTCTACATG 419
Haplochromis burtoni CATCTGAATAAAGTCATGAGTGAGATGAGTACAATCTACAGCACAGCCACAGTGTGTATGCTTGACAACCCGCTTAACTG 425
Hippocampus comes CACCTGAGCAAGATCATGAGTGACATGAGTACAATCTACAGCACAGCAACTGTGTGCATGATGGACGACCCGCTTAACTG 425
Ictalurus punctatus TATCTAAATAAAGTCATGAACGAGATGAGTACTATTTATGGCACAGGAACCGTGTGTAAAATCGATGACCCCTTCGACTG 419
Larimichthys crocea CATCTGAGTAAGGTCATGAGTGAGATGAGTACAATCTACAGCACAGCGACAGTGTGCCTTATGGACGACCCCCTTAACTG 425
Lates calcarifer CATCTAAGTAAGGTCATGAGCGAGATGAGCACAATCTACAGCACAGCCACAGTGTGTCTGATGGACGACCCCTTTAACTG 425
Latimeria chalumnae AGGCTCAATCAGATCCTAAGTGACATGAGCACACTTTATAGCACCGGAACAGTGTGTAAACCTGATGAGCCATCCGTTTG 554
Lepisosteus oculatus CATTTGAATAAAGTCCTAAGTGAGATGAGCACCATCTACAGCACTGGGACCGTGTGCAAGGTTGATGACCCTTTCGATTG 419
Macaca fascicularis CGGTTGAACACAATTCTAAATACAATGAGCACCATCTACAGTACTGGAAAAGTTTGTAACCCAAATAATCCCCAGGAATG 422
Manacus vitellinus AGACTGGGCACTGTGCTAAGTACAATGAGTACCATCTACAGTACTGGGACCGTCTGTAAAATCAATGATCCATCTGAGTG 425
Mus musculus CAGTTGAACACAATTCTGAACACCATGAGCACCATTTACAGTACTGGAAAAGTTTGCAACCCAAAGAACCCACAAGAATG 422
Neolamprologus brichardi CATCTGAATAAAGTCATGAGTGAGATGAGTACAATCTACAGCACAGCCACAGTGTGTATGCTTGACAACCCGCTTAACTG 425
Oncorhynchus mykiss CATCTGAACAAAGTGATGAATGAGATGAGCTCCATCTACAGTACAGGCACGGTGTGCAAGAGAGAAGACCCCTTAGACTG 428
Oreochromis niloticus CATCTGAATAAAGTCATGAGTGAGATGAGTACAATCTACAGCACAGCCAAAGTGTGTATGCTTGACGACCCGCTCAACTG 425
Poecilia formosa CATCTCAGTAAGGTCATGGGCGAGATGAGTACGATCTACAGCACCGCAACGGTTTGCCTGAAAGATGACCCCCTTAACTG 428
Poecilia latipinna CATCTCAGTAAGGTCATGGGCGAGATGAGTACGATCTACAGCACCGCAACGGTTTGCCTGAAAGATGACCCCCTTAACTG 428
Pseudopodoces humilis AGACTGAGCACTGTGCTAAATACAATGAGTACCATCTACAGCACTGGGACTGTCTGTAAAACCAACAATCCATCTGAGTG 419
Pundamilia nyererei CATCTGAATAAAGTCATGAGTGAGATGAGTACAATCTACAGCACAGCCACAGTGTGTATGCTTGACAACCCGCTTAACTG 425
Pygocentrus nattereri CATCTAGCTAAAGTCATGAATGAGATGAACACTATTTACAGCACAGGAACTGTCTGTAAAATTGACGACCCCTTTGACTG 419
Python bivittatus CAACTGAATACTGTTTTACGTAAAATGAGTACCATCTACAGTACTGGGACTGTGTGCAAACAAGATTATCCTTTTGACTG 410
Rattus norvegicus CAGTTGAACACAATTCTAAACACCATGAGCACCATTTACAGTACTGGAAAAGTTTGCAACTCAATGAATCCACAAGAATG 422
Scleropages formosus CAACTCAGCAGAGTCATGAGTGAAATGAGCACCCTGTACAGCACAGGCACTGTGTGTGACATCGACGAGCCATTCGACTG 428
Sinocyclocheilus anshuiensis GAACTGAGAAACATCATGTCAGAGATGAGCACGATTTACAACACAGCAACTGTGTGCAAAATTGATGATCCCACTGACTG 419
Xenopus tropicalis AGACTCAATCAAGTTCTAAATGAAATGAGCACAATATACAGCACACACGCTGTCTGTAAACCAGATGGATCAAAGGAATG 425
Lipotes vexillifer CGATTGAACACGATTCTAAGTACAATGAGCACCATCTACAGTAGTGGGAAAGTTTTGGACCCA...CATACACAGGAGTG 419
Orcinus orca CGATTGAACGCAATTCTAAGTACAATGAGCACCATCTACAGTAGTGGGAAAGTTTTGGACCCA...AATACACAGGAGTG 419
Homo sapiens CGGTTGAACACAATTCTAAATACAATGAGCACCATCTACAGTACTGGAAAAGTTTGTAACCCAGATAATCCACAAGAATG 422
430 440 450 460 470 480 490
conservation
Callorhinchus milii CCTGGGTCTCGAGCCAGGACTGACCATTTTGTTGGCCGAAAGCAAG...GATTACAATGAGAGGCTGTGGCCTGGGAAG 499
Alligator mississippiensis TTTACTGCTAGAGCCAGGTTTGGATGTTGTTATGGCTAGTGGGACT...GATTATTCAGAGAAACTCTGGGCTTGGCAAG 490
Anolis carolinensis TTTACTCCTGGAACCAGGCTTGGACATTATAATGGCAGAGAGCACT...GACTACTCAGAGAGATTGTGGGCTTGGCAAG 523
Astyanax mexicanus TCAGACTTTGGAACCAGGTTTGGAAGAAATCATGGCGAACAGCAGG...GACTACTATGAGCGGCTGCATGTGTGGGAGG 496
Boleophthalmus pectinirostris CCAGACGCTGGAGCCAGGTTTGGAGGAGGTCATGTTCTACAGTAAA...GATTATTATGAGCGGCTGCATGTCTGGGAGG 505
Cynoglossus semilaevis CCAGACTTTGGAGCCAGGTCTGGAGCATGTGATGTTCAACAGCGTA...AACTACGAAGAGCGCCTGCACGTGTGGGAAG 496
Danio rerio TCAGACTTTGGAGCCAGGCCTAGAGAGTATCATGGCAGAGAGCAGA...GACTATGATGAGCGTCTGCATGTATGGGAAG 496
Esox lucius TCAGACTTTGGAACCTGGTCTAGAGAGCGTGATGTCAAACATGGACAGTGACTACTATGAACGCCTGCATGTCTGGGAGG 505
Fundulus heteroclitus CGAGACTTTGGAGCCAGGCCTGGAGCATGTGATGGCCAACAGCAAA...AACTACGCGGAGCGTCTTCACGTGTGGGAGG 508
Gekko japonicus TTTGCCCCTGGAACCAGGTCTGGATGCTATAATGGCCGACAGTACT...GATTATTCAGAGAGACTGTGGGCTTGGCAAG 496
Haplochromis burtoni TCTGACTTTGGAGCCAGGACTGGACGAAATAATGGCCACTAGTCGA...AACTATGACGAACGTCTCCATGTGTGGGAGG 502
Hippocampus comes TCAGACTTTGGAGCCAGTCCTGGAACACGTGATGGCCACCAGCCGC...AACTATTCGGAGCTTCTGCACGTGTGGGAGG 502
Ictalurus punctatus TGAGAATCTGGAACCAGGCTTGGAAGCAATCATGGCTGACAGCAGG...GACTACTACGAGCGGCTGCATGTGTGGGAAG 496
Larimichthys crocea CCAGACTTTGGAGCCAGGCCTGGAGCACGTAATGGCCAACAGCAAA...GACTACGCCGAGCGGCTGCATGTGTGGGAGG 502
Lates calcarifer CCAGACTTTGGAGCCAGGCCTGGAGCATGTAATGTCCAACAGCCGA...AACTACTCTGAGCGTCTGCATGTGTGGGAGG 502
Latimeria chalumnae TTTGCCACTGGAACCAGGTCTGGATACCATAATGGCTGAAAGCACG...GATTACTCAGAGAGGCTGTGGCCTGGGAAG 631
Lepisosteus oculatus TGAAACTTTAGAACCAGGTCTTGAGCACACTATGGCTACAAGCACT...GATTACTACGAGCGGCTGCATGCCTGGGAGG 496
Macaca fascicularis CTTATTACTTGATCCAGGTTTGAATGAAATAATGGAAAAGAGTTTA...GACTACAATGAGAGGCTCTGGGCTTGGGAAG 499
Manacus vitellinus TTTGGTGCTGGAACCAGGTCTGGATGCTATTATGGCTGACAGCACT...GATTATCATGAGAGACTGTGGGCCTGGGAAG 502
Mus musculus CTTATTACTTGAGCCAGGATTGGATGAAATAATGGCGACAAGCACA...GACTACAACTCTAGGCTCTGGGCATGGGAGG 499
Neolamprologus brichardi TCTGACATTGGAGCCAGGACTGGACAAAATAATGGCCACTAGTCAA...AACTATGACGAACGTCTCCATGTGTGGGAGG 502
Oncorhynchus mykiss TCAAACCTTGGAACCAGGGCTGGAGAGTGTAATGGCCAACATGGACAGTGACTACTATGAGCGTCTGCATGTATGGGAGG 508
Oreochromis niloticus TCTGACTTTGGAGCCAGGACTGGATGAAATAATGGCCACTAGTCAA...AACTACGACGAACGTCTCCATGTGTGGGAGG 502
Poecilia formosa CCAGACTTTGGAGCCAGGCCTGGAGCATGTGATGTCCAACAGCCGA...GACTACGAAGAGCGTCTCCACGTGTGGGAGG 505
Poecilia latipinna CCAGACTTTGGAGCCAGGCCTGGAGCATGTGATGTCCAACAGCCGA...GACTACGAAGAGCGTCTCCACGTGTGGGAGG 505
Pseudopodoces humilis TTTGGTGCTGGAGCCAGGTCTGGATGCTATTATGGCTGAGAGCACT...GATTATAATGAGAGACTGTGGGCCTGGGAAG 496
Pundamilia nyererei TCTGACTTTGGAGCCAGGACTGGACGAAATAATGGCCACTAGTCGA...AACTATGACGAACGTCTCCATGTGTGGGAGG 502
Pygocentrus nattereri TCAGACTTTGGAACCAGGCTTGGAACAGATCATGGCTAACAGCAGG...GACTACTACGAACGACTGCATGTGTGGGAGG 496
Python bivittatus TTTGCCCTTAGAACCAGGTTTGGATGATATAATGGCAAACAACTGG...GATTACTCAGAGAGGCTATGGGCCTGGGAAG 487
Rattus norvegicus TTTTTTACTTGAACCAGGATTGGACGAAATAATGGCAACAAGCACA...GACTACAATCGTAGGCTCTGGGCTTGGGAGG 499
Scleropages formosus TCAAACTCTGGAACCAGGCCTGGAGAGCATCATGGCAGAGAGTAGG...GATTACTACAAGCGGCTGCATGTGTGGGAGG 505
Sinocyclocheilus anshuiensis TCAGACTTTGGAGCCAGGCCTAGAGAGTATCATGGCAAACAGCAGG...GACTATGACGAGCGTCTACATGTGTGGGAGG 496
Xenopus tropicalis TTTGCCACTGGAACCAGGTCTGGACAAAATCATGGCTGAAAGCATT...GATTATGAGGAAAGACTGTGGGCTTGGGAAG 502
Lipotes vexillifer CTTAGTACTTGAACCAGGTTTGGATGACATAATGGAAAACAGCAAA...GACTACAATCGGAGACTCTGGGCTTGGGAAG 496
Orcinus orca CTTAGTACTTGAACCAGGTTTGGATGACATAATGGAAAACAGCAAA...GACTACAGTCGGAGACTCTGGGCTTGGGAAG 496
Homo sapiens CTTATTACTTGAACCAGGTTTGAATGAAATAATGGCAAACAGTTTA...GACTACAATGAGAGGCTCTGGGCTTGGGAAA 499

```
                                500       510       520       530       540       550       560       570
conservation
Callorhinchus milii             GCTGGAGACATAATGTGGGAAGGCGCTTAGACCACTTTACGAAGATTATGCAGATCTGAAGAATAAGGCAGCAAAGCTG   579
Alligator mississippiensis      GTTGGAGAGCAGACATTGGCAAGAAGATGAGGCCACTGTATGAAGAATATGTTGAACTGAAAAATGAAGCTGCAAGGAGT   570
Anolis carolinensis             GTTGGAGAGCTGATGTGGGAAAGAAAATGAGGCCTTTGTATGAAGGTTATGTTGAATTGAACAATGAGGCTGCAAGAAGT   603
Astyanax mexicanus              GCTGGAGGGTTCAAGTGGGAAGAAGATGAGGAAGCTGTATGAAGAGTACGCAGACCTGAAGAACGAAGCAGCCAAACTC   576
Boleophthalmus pectinirostris   GCTGGAGACAGGAAGTGGGTAAAAGGATGAGGACGCTGTATGAAGACTACGTAGATCTGAAGAACGAAGCAGCCAAACTC   585
Cynoglossus semilaevis          GCTGGAGGAAGGAAGTGGGGAGGAGAATGAGGCCTCTGTATGAGGACTACGCCGATCTGAAGAACGAAGCTGCCAAACTC   576
Danio rerio                     GCTGGAGAGTGGCCACAGGGATGAAGATGAGACCCTTGTATGAAAAATACGTCGACCTGAAGAATGAAGCTGCCAAGCTC   576
Esox lucius                     GCTGGAGAGTGGAGGTGGGCAAGAAGATGAGGCCTCTCTATGAAGATTACGTGGACCTGAAGAACGAAGCTGCCAAACTC   585
Fundulus heteroclitus           GCTGGAGGAAAGAGGTGGGGAAGAAGATGAGGCCTTTGTACGAAGACTATGTGGACCTGAAGAATGAAGCTGCCAAACTT   588
Gekko japonicus                 GCTGGAGAGCTGACATTGGCAAGAAAATGAGGCCTTTATATGAAGGTTATGTTGAACTGAAGAATGAGATTGCAAGGGGG   576
Haplochromis burtoni            GCTGGAGGAAAGAAGTAGGAAAGAGGATGAGACCTCTCTACGAAGACTACGTGGATCTGAAGAATGAAGCTGCCAAGCTA   582
Hippocampus comes               GTTGGAGGAGGGAGGTGGGGAAGAGGATGAGGCCGCTCTATGAAGACTATGTGCTTCTGAAGAACGAAGCTGCAAAACTG   582
Ictalurus punctatus             GCTGGCGGGTGCAGGTGGGCAAGAAGATGAGGCGACTCTATGAAGACTATGTGGACTTGAAGAACGAAGCTGCCAGGCTG   576
Larimichthys crocea             GCTGGAGGAGAGAGGTGGGGAAGAGGATGAGGCCCCTGTATGAAGACTATGTGGATCTGAAGAATGAAGCTTCCAAATTA   582
Lates calcarifer                GCTGGAGGAAAGAGGTGGGGAAGAGGATGAGGCCTCTGTACGAAGATTATGTGGATCTGAAGAATGAAGCTGCCATACTA   582
Latimeria chalumnae             GCTGGAGAGTGGAGGTTGGCAAGAAGATGAGGCCACTTTATGAAGAATATGTAGAACTGAAAAACACTGCAGCTAAAATG   711
Lepisosteus oculatus            GCTGGAGGGTTGCAGTGGGGAAGAAAATGAGACCACTCTACGAAGACTATGTGGACTTGAAAAACGAAGCGGCAAAACTC   576
Macaca fascicularis             GCTGGAGATCTGAGGTCGGCAAGCAGCTGAGGCCATTATATGAAGAGTATGTGGTCTTGAAAAATGAGATGGCAAGAGCA   579
Manacus vitellinus              GCTGGAGAGCTGATGTTGGCAGGATGATGAGACCATTATACGAAGAGTATGTTGAGCTTGAAAACGAGGTTGCGAAGCTT   582
Mus musculus                    GCTGGAGGGCTGAGGTTGGCAAGCAGCTGAGGCCGTTGTATGAAGAGTATGTGGTCCTGAAAAACGAGATGGCAAGAGCA   579
Neolamprologus brichardi        GCTGGAGGAAAGAAGCAGGAAAGAGGATGAGACCTCTCTACGAAGACTACGTGGATCTGAAGAATGAAGCTGCCAAGCTA   582
Oncorhynchus mykiss             GCTGGAGAGTGGAGGTAGGGAAGAAGATGAGGCCTCTGTATGAAGACTATGTGGACCTTAAGAACGAAGCTGCCAAACTC   588
Oreochromis niloticus           GCTGGAGGAAAGAAGTAGGAAAGAGGATGAGACCTCTCTACGAAGACTACGTGGATCTGAAGAATGAAGCTGCCAAGCTA   582
Poecilia formosa                GCTGGAGGAGAGAGGTGGGGAAAAGGATGAGGCCTCTGTACGAAGACTATGTGGATCTGAAGAATGAAGCTGCCAAACTG   585
Poecilia latipinna              GCTGGAGGAGAGAGGTGGGGAAAAGGATGAGGCCTCTGTACGAAGACTATGTGGATCTGAAGAATGAAGCTGCCAAACTG   585
Pseudopodoces humilis           GCTGGAGGGCTGGTGTTGGCAGGATGATGAGACCATTATATGAAGAGTATGTTGAACTTGAAAATGAGGTTGCAAGGCTT   576
Pundamilia nyererei             GCTGGAGGAAAGAAGTAGGAAAGAGGATGAGACCTCTCTACGAAGACTACGTGGATCTGAAGAATGAAGCTGCCAAGCTA   582
Pygocentrus nattereri           GCTGGAGAGTTCAAGTGGGAAGAAAATGAGGTGGCTATATGAAGATTACGTCGACCTGAAGAATGAAGCAGCCAAACTC   576
Python bivittatus               GCTGGAGAGCTGATGTTGGCAAGAAGATGAGGCCTTTATATGAAAGTTATGTTGAACTGAAAAATAAGTATGCAAGATTG   567
Rattus norvegicus               GCTGGAGGGCTGAGGTCGGCAAGCAGCTGAGGCCGTTATATGAAGAGTATGTGGTCCTGAAAAATGAGATGGCAAGAGCA   579
Scleropages formosus            GCTGGAGGGTGAATGTGGGAAGGAGATGAGGCCCCTCTATGAAGTCTACGTGGACCTAAAGAACGAAGCTGCCAGACTG   585
Sinocyclocheilus anshuiensis    GATGGAGAGTGGCCGCAGGGATGAAGATGAGACCCCTTTATGAGAAATATGTGGACCTGAAGAATGAAGCCGCCAAACTC   576
Xenopus tropicalis              GTTGGAGAGCTGGAGCTGGAAAGAAAATGAGATCGTTGTATGAGGAATATGTTGATCTGGAAAATGAGGCTGCCCGTCTT   582
Lipotes vexillifer              GCTGGAGGGCTGAGGTCGGCAAGCAGCTGAGGCCATTTTATGAAGAGTATGTGGTCCTGGAAAATGAGATGGCCAGAGCC   576
Orcinus orca                    GCTGGAGGGCTGAGGTCGGCAAGCAGCTGAGGCCATTATATGAAGAGTATGTGGTCCTGGAAAACGAGATGGCCAGAGCC   576
Homo sapiens                    GCTGGAGATCTGAGGTCGGCAAGCAGCTGAGGCCATTATATGAAGAGTATGTGGTCTTGAAAAATGAGATGGCAAGAGCA   579
```

```
                                580       590       600       610       620       630       640       650
conservation
Callorhinchus milii             AATGGTTATCAAGACTATGGGGATTACTGGCGGGGAAATTATGAGACGAAAGAC...ATAGGCGAATATGCATACAGTCG   656
Alligator mississippiensis      AATAATTATAATGACTATGGAGATTACTGGAGAGGAAATTATGAAACAGATTTT...CCTCCAGAATATAGATACAGTCG   647
Anolis carolinensis             AATGGTTATGCTGACTATGGAGATTATTGGAGAGCCGATTATGAAGTAGATTTC...CCTGAGGAGTATCGATACCCACG   680
Astyanax mexicanus              AACAATTATAAAGACTATGGAGATTACTGGAGAGCCAATTATGAAACAGAAGAT...GAGCCCAAATACAGCTACTCTCG   653
Boleophthalmus pectinirostris   AACGGTTTGAGGACTATGGGGCGTACTGGAGATATAACTATGAAACCATCGAAGAGCAGCCTCCCTACACTTACACCCG   665
Cynoglossus semilaevis          AATAAGTTAAAGACTATGGAGACTACTGGAGGTCCAACTATGAAACCGTTGAGGAGGTTGATAAGTACAAATACACCGG   656
Danio rerio                     AACAACTACGAAGACCATGGAGATTATTGGAGGGGAGATTATGAAACTATTGAT...GATCCCAAATACAGTTACTCCCG   653
Esox lucius                     AATGGTTATAAGGACCATGGAGATTATTGGAGGTCCAACTACGAAACCAACGAC...GACAGCCCCTACAAGTACACCAG   662
Fundulus heteroclitus           AACGGCTTTGAAGACTATGGAGCTTATTGGAGATACAATTATGAGACCATTGAGGAAGACGTTCAGTACAAGTATACCAG   668
Gekko japonicus                 ACTAATTATTCTGACTATGGAGATTACTGGAGAGGAAATTATGAAACAGATTAT...CCCGAGGATTATCGATACAGACG   653
Haplochromis burtoni            AATGGTTTTGAAGACTATGGCGCTTACTGGAGATACAACTATGAGACTCTTGAGGACGACATCATGTACCATTACACAGG   662
Hippocampus comes               AATGGGTTTGAGGACTATGGCGCTTACTGGAGATATAACTATGAGACTATTGATGAGGAAAGTCCATACGATTATACCAG   662
Ictalurus punctatus             AACAATTTTAAAGACCATGGAGACTACTGGAGATCTAATTATGAAACAATTGAT...GAGCCACAATACAGCTACACTCG   653
Larimichthys crocea             AACAACTTTGAAGACTATGGAGCTTACTGGAGATATAACTATGAGACCATTGAGGAGGATGCTCAGTTCAACTACACCAG   662
Lates calcarifer                AATGGTTTTGAAGACTATGGGGCTTATTGGAGATATAACTATGAGACCATTGATGAGGACGTTCAGTACAAATATACCAG   662
Latimeria chalumnae             AATGGTTATTCTGATTATGGAGATTACTGGAGATCAAACTACAAAACACAGGATACTACTGGAAAATATGCCTACACTGG   791
Lepisosteus oculatus            AACAACTACAAAGATTATGGGGACTATTGGAGAGGAAACTATGAAGCTACAGAT...GATGCAGTTTACGCTTACACTCG   653
Macaca fascicularis             AATCATTATAAGGACTATGGGGATTATTGGAGAGGAAACTATGAAGTAAACGGG...GTAGATGGCTATGACTACAACCG   656
Manacus vitellinus              AATAATTATTCTGACTATGGAGATTACTGGAGAGCAAATTATGAAGCCAGTTTT...CCAAGAGGTTATGAATACAGCCG   659
Mus musculus                    AACAATTATAACGACTATGGGGATTATTGGAGAGGGGACTATGAAGCAGAGGGA...GCAGATGGCTACAACTATAACCG   656
Neolamprologus brichardi        AATGGTTTTGAAGACTATGGCGCTTACTGGAGATACAACTATGAGACTCTTGAGGACGACATCATGTACCATTACACAGG   662
Oncorhynchus mykiss             AATGATTATGAGGACTACGGAGATTATTGGCGGTCCAACTATGAAACTATCGAT...GACAGCCCATACAACTACGCCAG   665
Oreochromis niloticus           AATGGCTTTGAGGACTATGGCGCTTACTGGAGATACAACTATGAGACTCTTGAGGACGACGTCATGTACCATTACACAGG   662
Poecilia formosa                AATGGTTTTCAAGACTACGGAGCTTATTGGAGATACAATTATGAGACCCTCGATGAAGACGTTCCTTACAAGTATACCAG   665
Poecilia latipinna              AATGGTTTTCAAGACTACGGAGCTTATTGGAGATACAATTATGAGACCCTCGATGAAGACGTTCCTTACAAGTATACCAG   665
Pseudopodoces humilis           AATGGTTATTCTGACTATGGAGACTACTGGAGAGCAAATTATGAAGCAAAGTCT...CCAGAGAAGTACAAATACAGCCG   653
Pundamilia nyererei             AATGGTTTTGAAGACTATGGCGCTTACTGGAGATACAACTATGAGACTCTTGAGGACGACATCATGTACCATTACACAGG   662
Pygocentrus nattereri           AATAATTATAAAGACTATGGGGACTACTGGAGAGCCAATTATGAGACCATAGAT...GAGCCCATTTATAGCTACACCCG   653
Python bivittatus               AGGGGCTATGCTGACTATGGAGATTATTGGAGAGCAAATTATGAAGTAGATTCT...CCACCAGAGTATCAATACAAACG   644
Rattus norvegicus               AACAATTATGAAGACTATGGGGATTATTGGCGAGGGGATTATGAAGCAGAGGGA...GTAGAAGGTTACAACTACAACCG   656
Scleropages formosus            AATGGTTATGCAGACTACGGGGACTACTGGAGGGCCAACTACGAGACCATCGAT...GAGGGAAACTACAGCTATACCCG   662
Sinocyclocheilus anshuiensis    AACAATTATGAAGACCATGGAGATTACTGGAGGGGGGATTATGAAACCATCGAT...GAACCCAAATACAGTTACTCCCG   653
Xenopus tropicalis              AATGGTTATAATGACTATGGAGATTATTGGAGAGGAAACTATGAAACTCTGGCA...ACAGACATGTATGCCTACAGCCG   659
Lipotes vexillifer              AACAATTATGAGGACTATGGGGACTATTGGAGAGGGGATTATGAAGTGATGGGG...GCAGGTGACTATGACTACAGCCG   653
Orcinus orca                    AACAATTATGAGGACTATGGGGACTATTGGAGAGGGGATTATGAAGTGACGGGG...GCAGGTGACTATGACTACAGCCG   653
Homo sapiens                    AATCATTATGAGGACTATGGGGATTATTGGAGAGGAGACTATGAAGTAAATGGG...GTAGATGGCTATGACTACAGCCG   656
```

```
                              660           670           680           690           700           710           720           730
conservation
Callorhinchus milii           AGACGATCTTGTGAAAGATGTGGAAAGCCTGTTTGAAGAGGTGAAGCCTCTGTATAGAGAACTCCATGCCTATGTGAGAG  736
Alligator mississippiensis    TAACCAGCTCATTGATGATGTAGAACGGACATTTGAGCAGATAAAACCTTTATATCAGCAACTGCATGCTTACGTGAGAT  727
Anolis carolinensis           TGCAAAGCTGATTACTGATGTGGAAAACACATTTAAACAAATAGAACCCTTGTACAGGCAGCTGCATGCTTATGTGAGGC  760
Astyanax mexicanus            AGACGAACTGATGAGAGATGTGAGAAGCATATACAGTGAGATCATGCCTCTGTACAAAGAGCTTCATGCTTATGTAAGAG  733
Boleophthalmus pectinirostris AGATGAGCTCATGCAAGACGTCCGCAAAATATACCACCAGATCATGCCTCTGTACAAAGAGCTCCATGCTTACGTCAGGG  745
Cynoglossus semilaevis        AGACCAGCTCATGGAAGATGTCCGTTCAATATACAAAGAGATCCTGCCTCTGTATAAGGAGCTTCATGCCTATGTGAGGG  736
Danio rerio                   AGACCAAGTTATAGAAGACGCCAGGCGCATTTACAAAGAGATCTTACCACTGTACAAAGAGCTTCATGCTTACGTGAGAG  733
Esox lucius                   GGAACAACTGATCACTGATGTGAGGCGCATTTACAAAGAGATTCTCCCCCTCTACAAAGAGCTGCATGCCTATGTGAGGA  742
Fundulus heteroclitus         AGATCAGCTGATGGAAGACGTTCGTTCAATATACAAAGAGATCATGCCTCTATACAAGGAGCTGCATGCCTATGTGAGAG  748
Gekko japonicus               CGAGCAGCTGATCACCGATGTGGAAGAAACATTTCAACAAATAAAACCTTTGTATCAGCAGCTACATGCTTATGTGAGGA  733
Haplochromis burtoni          CGATGAGTTAATGGAAGATGTCCGTGTAATATACAAACAGATCCTGCCTTTATACAAGGAGCTGCATGCCTATGTGAGAG  742
Hippocampus comes             GGATCAGCTGATGGAAGATGTACGCTCCATCTATAAACAGATCCTGCCCTTATACAAGGAGCTACATGCCTACGTGAGGG  742
Ictalurus punctatus           AGATGAACTGATGACAGATGTGAGACGCATATATAATGAGATTCTGCCTTTGTACAAAGAGCTTCATGCATATGTGAGAG  733
Larimichthys crocea           AAATGAGCTCATGGAAGACGTCCGCTCTATATACAAAGAGATCCTGCCTTTGTACAAGGAGCTGCATGCCTATGTGAGAG  742
Lates calcarifer              AAATCAGCTGATGCAAGATGTCCGTTCAATATACAAAGAGATCCTGCCATTATACCAGGATCTGCATGCCTATGTGAGAG  742
Latimeria chalumnae           GGATGATCTCATCAAAGATGTTCACAAAATATTTGATGAGGTGAAGCCTCTGTACAAAGAACTACATGCCTATGTGAGAG  871
Lepisosteus oculatus          AGACCAGCTTATGGAAGATGTCCGACGCTTATATCGGGAGATAATGCCTTTGTATAAAGAGCTCCATGCTTATGTGAGAG  733
Macaca fascicularis           CGACCAGTTGATTGAAGATGTGGAACGTACCTTTGAAGAGATTAAACCATTATATGAACATCTTCATGCCTATGTGAGGG  736
Manacus vitellinus            TGACCAGCTGATAGAAGATGTGGAGAAGACATTTGAGCAGATAAAACCTCTGTACCAGCAGCTGCATGCCTACGTGAGAC  739
Mus musculus                  TAACCAGTTGATTGAAGATGTAGAACGTACCTTCGCAGAGATCAAGCCATTGTATGAGCATCTTCATGCCTATGTGAGGA  736
Neolamprologus brichardi      CGATGAGTTAATGGAAGATGTCCGTGTAATATACAAACAGATCCTGCCTTTATACAAGGAGCTGCATGCCTATGTGAGAG  742
Oncorhynchus mykiss           GGGCCAACTGATGACCGATGTGAGGCGCATTTACAAAGAGATCCTCCCCCTCTACAAAGAGCTGCATGCCTATGTGAGGT  745
Oreochromis niloticus         CAATGAGTTAATGGAAGATGTCCGTGTAATATACAAACAGATCCTGCCTTTATACAAGGAGCTGCATGCTTATGTGAGAG  742
Poecilia formosa              AGATCAGCTGATGGAAGATGTTCGCTCAATATACAAAGAGATCATGCCTTTATACAAAGAGCTGCATGCCTATGTAAGAT  745
Poecilia latipinna            AGATCAGCTGATGGAAGATGTTCGCTCAATATACAAAGAGATCATGCCTTTATACAAAGAGCTGCATGCCTATGTAAGAT  745
Pseudopodoces humilis         TGACCAGCTGATTGAAGACGTGGAGAAAACATTTGAGCAGATAAAACCTCTGTATGAGCAGCTGCATGCCTATGTGAGAC  733
Pundamilia nyererei           CGATGAGTTAATGGAAGATGTCCGTGTAATATACAAACAGATCCTGCCTTTATACAAGGAGCTGCATGCCTATGTGAGAG  742
Pygocentrus nattereri         GGATGAACTGATGACGGATGTGAGAAGCATATACCACGAGATCATGCCTTTGTACAAAGAGCTTCACGCTTACGTGAGGG  733
Python bivittatus             TGCAGAGCTGATGACTGATGTGGAAAACACATTTCAAGAAATAAAGCCTTTGTATGAGCATCTACATGCTTACGTACGGC  724
Rattus norvegicus             AAACCAGTTGATCGAAGACGTAGAAAATACCTTCAAAGAGATCAAACCGTTGTATGAGCAACTTCATGCCTATGTGAGAA  736
Scleropages formosus          CGATGATCTTATGGCAGATGTTCGGCGCATATATAAAGAGATCATGCCATTGTACAAAGAGCTGCATGCTTATGTGCGGA  742
Sinocyclocheilus anshuiensis  AGACCAAGTTATGGAAGATGCCAGGCGCATTTACCAGGAGATCTTGCCTTTGTACAAAGAGCTTCATGCTTATGTGAGAG  733
Xenopus tropicalis            TGATGATCTTATAAAGGATGTGGAAAGAACATACCAAGAGATACTACCACTCTACAAGGAACTACATGCATTTGTAAGAG  739
Lipotes vexillifer            CGACCAGCTGATCACAGATGTGGAACGTACCTTTGCAGAGATTAAACCATTGTATGAACAACTTCACGCTTATGTGAGGG  733
Orcinus orca                  CGACCAGTTGATCAGAGATGTGGAACGTACCTTTGCAGAGATTAAACCATTATATGAACAACTTCACGCTTTTGTGAGGG  733
Homo sapiens                  CGGCCAGTTGATTGAAGATGTGGAACATACCTTTGAAGAGATTAAACCATTATATGAACATCTTCATGCCTATGTGAGGG  736

                              740           750           760           770           780           790           800           810
conservation
Callorhinchus milii           CAAAACTGATGGAAACTTTTGGATCAGAACATATCAGCCGGACTGGAGGCCTGCCTGCTCATTTACTGGGTGACATGTGG  816
Alligator mississippiensis    ACAAGTTGGAACAGGTCTATGGCTCAGATCGCATCAGCAAAACAGGATGTCTTCCTGCCCATTTACTTGGTGACATGTGG  807
Anolis carolinensis           ACAATTTATGGAAGGTCTATGGCTCAACGAACATTGACCCCAACGGATGTCTTCCTGCTCATTTACTTGGTGACATGTGG  840
Astyanax mexicanus            CCAAACTGCAGAATACCTAT...CCTGGTCATATTGCTTCTAATGGAGGCCTTCCAGCTCATTTACTTGGTGACATGTGG  810
Boleophthalmus pectinirostris CCAAACTGATGGACGTCTAC...CCCGGACACATCCACCCTCATAGCCCTCTGCCCGCCCATCTGCTCGGTGACATGTGG  822
Cynoglossus semilaevis        CCAGTCTGATGAAGGTCTAC...CCGGGCCACATTGATCCGAAAGGACCCCTGCCAGCTCACCTGCTGGGCGACATGTGG  813
Danio rerio                   CAAAACTGCAAGACGTCTAT...CCTGGTCACATTGGCTCTGATGCCTGTCTTCCAGCACATTTGCTTGGTGATATGTGG  810
Esox lucius                   CTAAGCTTCAGGCTAAACAT...CCTGAGCACATTCACCCGGAGGGAGCGCTGCCTGCCCATCTGCTGGGCGACATGTGG  819
Fundulus heteroclitus         CCAAGCTTATGGAGGTCTAC...CCAGGACACATTGAATCAGATGGACCACTGCCTGCCCACCTTCTTGGTGACATGTGG  825
Gekko japonicus               GTAATCTCCAGAAAATATATGGCTCACATCGCATTGACTCAAAAGGATGTCTTCCTGCTCATTTGCTTGGTGACATGTGG  813
Haplochromis burtoni          CCAAGCTTATGGAGGTCTAT...CCAGGGCATATTGATTCAGATGGATTTCTCCCAGCCCACTTGCTGGGTGACATGTGG  819
Hippocampus comes             CTAAGCTGATGAAAGTACAC...AAAGGGCATATTGATGAACAAGGACCTCTGCCAGCGCATCTCTTGGGCGACATGTGG  819
Ictalurus punctatus           CCAAATTGCAGGACGCCTAT...CCTGGCCACATCGTCTCTAACGGAGGTCTTCCAGCCCATTTGCTTGGTGATATGTGG  810
Larimichthys crocea           CCAAGCTCATGGAGGTCTAC...AAAGAAGGCATTGATTCAGAAGGACCTCTGCCAGCCCACCTGCTGGGTGACATGTGG  819
Lates calcarifer              CCAGGCTTATGGAGGTCTAC...CCAGGACATATTGATTCACAAGGACCTCTTCCAGCCCACCTGCTGGGTGACATGTGG  819
Latimeria chalumnae           CAAAGCTGTATGAGAAGTACGGCCCCCAACATATTAACCTGATGGGGGTCTTCCTGCTCATTTACTTGGTGACATGTGG  951
Lepisosteus oculatus          ATAAACTACGTGAAGTATAT...CCAGGACACATCAGCCCAAATGGTGGTCTTCCAGCTCATTTACTGGGTGATATGTGG  810
Macaca fascicularis           CAAAGTTGATGAATGCCTAC...CCTTCCTATATTAGTCCAACTGGATGCCTTCCTGCTCATTTGCTTGGTGATATGTGG  813
Manacus vitellinus            ACCACCTGGAGCGAGTGTACGGCCCCAAGCTCATCAGCTCCACAGGATGCCTCCCTGCTCACTTGCTTGGTGATATGTGG  819
Mus musculus                  GGAAGTTGATGGATACCTAC...CCTTCCTACATCAGCCCCACTGGATGCCTCCCTGCCCATTTGCTTGGTGATATGTGG  813
Neolamprologus brichardi      CCAAGCTTATGGAGGTCTAT...CCAGGGCATATTGATTCAGATGGATTTCTCCCAGCCCACTTGCTGGGTGACATGTGG  819
Oncorhynchus mykiss           CTAAGCTGCAGGCCAAACAC...CCTGAACACATCCACCCAGAGGGGGGCCTACCTGCACATCTACTGGGTGACATGTGG  822
Oreochromis niloticus         CCAAGCTTATGGAGGTCTAT...CCAGGGCATATTGATTCAGATGGATTTCTCCCAGCCCACTTGCTCGGTGACATGTGG  819
Poecilia formosa              CCAGGCTTATGGAGGTCTAC...CCAGGATACATTGACTCACAGGGACCCCTGCCCGCCCACCTCCTTGGTGACATGTGG  822
Poecilia latipinna            CCAGGCTTATGGAGGTCTAC...CCAGGATACATTGACTCACAGGGACCCCTGCCCGCCCACCTCCTTGGTGACATGTGG  822
Pseudopodoces humilis         ACAAGCTGGGGCAAGTGTATGGCCCTGAGCTCATCAGCACCACAGGAGGCCTCCCTGCTCACTTGCTGGGTGATATGTGG  813
Pundamilia nyererei           CCAAGCTTATGGAGGTCTAT...CCAGGGCATATTGATTCAGATGGATTTCTCCCAGCCCACTTGCTGGGTGACATGTGG  819
Pygocentrus nattereri         CCAAACTACAGGAAACCTAC...CCTGGGCACATCGCTTCTAATGGAGGCCTTCCAGCCCATTTACTAGGTGACATGTGG  810
Python bivittatus             GCCATCTGTACAAACGCTATGGCCCAGGGCTCATTAACCCCAAAGGATCTCTTCCTGCTCATTTACTTGGTGACATGTGG  804
Rattus norvegicus             CGAAGTTGATGGAAGTGTAC...CCTTCTTACATCAGCCCTACTGGATGCCTCCCTGCTCATTTGCTTGGTGATATGTGG  813
Scleropages formosus          GCAAACTTCAAGAGACTTAC...CCTGGACACATAAGTGCAACAGGAGGTCTTCCTGCACATCTGCTGGGTGATATGTGG  819
Sinocyclocheilus anshuiensis  CAAAACTGCAAGATGTCTAT...CCTGGTCACATCGCCTCTGACGCTTGCCTTCCAGTACACCTGCTTGGTGATATGTGG  810
Xenopus tropicalis            GAAATCTGCAGCAAGTCTATGGTTCTCGGTACATCAGTGACTCTGGATGCCTTCCTGCTCATTTGCTTGGTGATATGTGG  819
Lipotes vexillifer            CAAAGTTGATGGATGCCTAC...CCTTCCCGTATCAGTCCGACTGGATGCCTCCCTGCCCATTTGCTTGGTGATATGTGG  810
Orcinus orca                  CAAAGTTGATGGATGCCTAC...CCTTCCCGTATCAGTCCGACTGGATGCCTCCCTGCCCATTTGCTTGGTGATATGTGG  810
Homo sapiens                  CAAAGTTGATGAATGCCTAT...CCTTCCTATATCAGTCCAATTGGATGCCTCCCTGCTCATTTGCTTGGTGATATGTGG  813
```

```
                             820       830       840       850       860       870       880       890
conservation
Callorhinchus milii          GGAAGGTTTTGGGCAAACCTGTATCCTTGGTCAATTCCGTATCCGTCAGAGGAAGACATCGATGTGACACAAGCAATGGT   896
Alligator mississippiensis   GGGAGATTCTGGACAAATTTGTATCCCTTGACAGTTCCCTATCCAGACAAACCAAACATTGATGTAACTAATACAATGGT   887
Anolis carolinensis          GGAAGGTTTTGGACAAATTTATACCCATTAATGATTCCCTATCCAGCTAAACCAAATATTGATGTCTCTTCTGCAATGGT   920
Astyanax mexicanus           GGAAGGTTCTGGACCAACCTTTACCCACTAGCTGTTCCTTATCCTGACAAGCCTGATATCGACGTGAGCCCTGCAATGGT   890
Boleophthalmus pectinirostris GGACGATTCTGGACCAACCTGTATCCTCTGTCCATCCCCTTCCCCGACAGACCAGATATCGACGTCAGCAAAACCATGGT  902
Cynoglossus semilaevis       GGGCGATTCTGGACAAACCTGTACCGTCACAGTATCCCATATCCAGAAAAAACAGATATTGATGTCAGCAACACCATGGT   893
Danio rerio                  GGACGGTTTTGGACCAACTTGTATCCTCTCATGATCCCATATCCAGACAGGCCTGATATTGATGTGAGCTCCGCAATGGT   890
Esox lucius                  GGAAGGTTTTGGACAGGGCTGTACCCTATCTCAACCCCCTTCCCAGAGAAAACGGACATTGATGTGACCAGTGCTATGAT   899
Fundulus heteroclitus        GGAAGATTCTGGACCAACCTGTATCCTCTGTCGATCCCTTACCCTGACAAACCAGACATTGATGTCAGCAGCACCATGGT   905
Gekko japonicus              GGTAGATTTTGGACAAATTTATATCCTTTAATGATTCCCTATCCAAATAAACCAAATATTGATGTGTCTTCTGCAATGGT   893
Haplochromis burtoni         GGAAGATTCTGGACCAACCTTTATTCTCTAACAGTGCCCTATCCTGACAAACCAGATATCGATGTCAGTCAGTCTATGGT   899
Hippocampus comes            GGAAGATTCTGGACTAACTTGTATCCTCTGTCAATGCCCTACCAGGGT......GAGATTGATGTCAGCAAAACAATGGT   893
Ictalurus punctatus          GGAAGGTTTTGGACCAACCTTTACTCACTCTGTGTCCCTTATCAAGACAAGCCTGATATCGACGTGACCTCTGCGATGAT   890
Larimichthys crocea          GGAAGATTCTGGACTAACCTGTACCCTCTATCAATTCCCTACCCCGGAAAAAAAGATATTGATGTCAGCGATGAAATGGT   899
Lates calcarifer             GGAAGATTCTGGACCAACCTGTACCCTCTGTCTGTCCCCTACCCTGAAAAACCAGATATTGATGTCAGCAAAACTATGGT   899
Latimeria chalumnae          GGAAGATTTTGGACAAACTTGTACCCCTTGGCTGTCCCATACCCACATAAGACAAGCATAGATGTGACAAAACCTATGGT  1031
Lepisosteus oculatus         GGCCGATTTTGGACAAACCTATACCCTTTATCAATCCCCTATAAAAACAAAGAGGATATTGATGTGACATCTGCCATGGT   890
Macaca fascicularis          GGTAGATTTTGGACAAATCTGTACTCTTTGACAGTTCCCTTTGGACAGAAACCAAACATAGATGTTACTGATGCAATGGT   893
Manacus vitellinus           GGTAGGTTTTGGACAAATCTGTATGCCTTGACTGTTCCCTATCCAGCCAAACCCAACATTGACGTAACTGATGCAATGGT   899
Mus musculus                 GGTAGATTTTGGACAAATCTGTACCCTTTGACTGTTCCCTTTGCACAGAAACCAAACATAGATGTTACTGATGCAATGAT   893
Neolamprologus brichardi     GGAAGATTCTGGACCAACCTTTATTCTCTAACAGTGCCATATCCTGACAAACCGGATATCGATGTCAGTCAGTCTATGGT   899
Oncorhynchus mykiss          GGAAGGTTTTGGACAGGTCTTTACCCCATCTCAACCCCTTTTCCAGAGAAAACCGACATTGATGTGACGGAAGCTATGAT   902
Oreochromis niloticus        GGAAGATTCTGGACCAACCTTTATTCTCTATCAGTGCCCTATCCTGACAAACCGGATATCGATGTCAGCCAGTCTATGGT   899
Poecilia formosa             GGAAGATTCTGGACCAACCTGTATCCTCTGTCAGTCCCCTACCCCGACAAACCAGACATTGACGTCAGCAACACTATGGT   902
Poecilia latipinna           GGAAGATTCTGGACCAACCTGTATCCTCTGTCAGTCCCCTACCCTGACAAACCAGACATCGACGTCAGCAACACTATGGT   902
Pseudopodoces humilis        GGTAGATTCTGGACAAATCTCTATGCCTTGACTGTTCCCTATCCAGCCAAACCAAACATTGATGTAACTTCTGCAATGGT   893
Pundamilia nyererei          GGAAGATTCTGGACCAACCTTTATTCTCTAACAGTGCCCTATCCTGACAAACCAGATATCGATGTCAGTCAGTCTATGGT   899
Pygocentrus nattereri        GGAAGGTTTTGGACCAACCTTTACTCATTATCTGTCCCTTATCCAGACAAGCCTGATATTGACGTGAGCTCAGCAATGGT   890
Python bivittatus            GGTAGATTTTGGACAAATTTATATCCTTTAATGGTGCCCTATCCAAATAAACCAAACATTGACGTAACCTCTGCAATGGT   884
Rattus norvegicus            GGTAGGTTTTGGACAAATCTGTACCCTTTGACTACTCCCTTTCTTCAGAAACCAAACATAGATGTTACTGATGCAATGGT   893
Scleropages formosus         GGAAGGTTCTGGACCAACCTGTACCCGCTATCCATCCCCTACCCCAACAAAGAAGACATTGATGTAACGTCTGCTATGAT   899
Sinocyclocheilus anshuiensis GGAAGGTTTTGGACCAACTTGTATCCTCTCATGATCCCGTATCCAGAAAAGCCTGATATCGATGTGAGCTCTGAAATGGT   890
Xenopus tropicalis           GGAAGATTTTGGACAAATTTGTATCCTCTGATGGTCCCCTACCCAAACAAAGAAAGCATTGATGTGACTCCTACCATGGT   899
Lipotes vexillifer           GGGAGATTTTGGACAAATCTGTACCCTTTGACAGTCCCCTTTGGAGAGAGACCAAGCATAGATGTTACCAAAGAAATGCA   890
Orcinus orca                 GGGAGATTTTGGACAAATCTGTACCCTTTGACAGTCCCCTTTGGAGAGAGACCGAGCATAGATGTTACCAAAGAAATGCA   890
Homo sapiens                 GGTAGATTTTGGACAAATCTGTACTCTTTGACAGTTCCCTTTGGACAGAAACCAAACATAGATGTTACTGATGCAATGGT   893

                             900       910       920       930       940       950       960       970
conservation
Callorhinchus milii          AGAACAGGGCTGGACTGCAAAAAGAATGTTTGAATCAGCAGATAAATTCTTTCAGTCTGTGGGCCTTCAGCCGATGAATG   976
Alligator mississippiensis   CGAAAAGAACTGGAATGCTTCAAAAATATTTAAAGCAGCTGAGAACTTTTTTGTTTCCATTGGCCTCCCTCCAATGACTC   967
Anolis carolinensis          AAAAAAGAACTGGGATGTGACCACTATATTTAAGGCTGCTGAGACCTTTTTTGCCTCTGTCGGACTCTATGAAATGACTG  1000
Astyanax mexicanus           AGCACAGGGTTGGGATGAAGAACGGTTGTTCAAAGAAGCAGAGAAGTTCTTTGTGTCTGTGAACATGTCGGCCATGTTTC   970
Boleophthalmus pectinirostris CGAGCAGGGTTGGAAGGAGCAGCAGTTCTTTCAGGAGGCAGAGAAGTTCTTCATGTCTGTGGGCCTGTACAAAATGTTTG  982
Cynoglossus semilaevis       GGCGAAGGGCTGGACCGAGACTCGACTCTTTAAAGAAGCAGAGAAGTTTTTCATGTCCGTAGGTATGGAGAAGATGTTTG   973
Danio rerio                  GGAGCAGGGTTGGGATGAAATCCGTCTGTTTAAAGAGGCAGAAAAATTCTTCATGTCTGTAAACATGCCTGCAATGTTTG   970
Esox lucius                  CGAACAGGGTTGGCCTAAAAAGAGACTATTCCAGGAGGCAGAGAAGTTTTTCATGTCAGTGGGTCTTTACGAAATGTTCG   979
Fundulus heteroclitus        GGCGCAGGGCTGGAATGAAACACTTTTTTTTAAAGAAGCAGAGAAGTTCTTTATGTCTGTGGGGTTATACGAGATGTTTG   985
Gekko japonicus              TGAGAAGAACTGGAATGTAACAGCTATATTTAAAGCTGCTGAGAAGTTTTTTGTCTCAGTTGGCCTCTACAACATGACAG   973
Haplochromis burtoni         GGACAAGGGTTGGACCGAGCTTCAGCTTTTCCGAGAAGCTGAGAAGTTCTTCATGTCTGTGGGCCTTTACAAGATGTTTG   979
Hippocampus comes            GGAAAAGGGCTGGACTGAGCGCCGACTATTTGAAGAGGCGGAGAAGTTTTTTGAATCGGTGGGCTTGTATAAAATGTTTG   973
Ictalurus punctatus          AGCACAGGGCTGGAAAGAACAGCGGCTGTTCGAGGAAGCAGAAAAGTTCTTCGTATCCGTGAACATGTCGGCCATGTTTC   970
Larimichthys crocea          GAAGCAGGGTTGGACTGAGATTCGGCTTTTTGAAGAAGCAGAGAAGTTCTTCATGTCTGTGGGTCTTTATGAGATGTTCC   979
Lates calcarifer             GGAGAAGGGTTGGAATGAACTTCGGCTTTTTAAAGAAGCAGAGACGTTCTTCATGTCTGTGGGCCTGTATGAGATGTTTG   979
Latimeria chalumnae          GGATCAGGACTGGAATCCAAAGAGAATGTTTGAGGAAGCAGAAAAGTTTTTCCAATCAGTTGGCCTCCCGGGCATGAATG  1111
Lepisosteus oculatus         CAATCAGGGCTGGACGGTGCTGAGAATGTTTAGAGAAGCAGAAAAATTCTTTATGTCTGTTGGCCTGTATAAAATGTATG   970
Macaca fascicularis          GAACCAGGCCTGGAATGCACAGAGAATATTCAAGGAGGCCGAGAAGTTCTTTGTATCTGTTGGTCTTCCTAATATGACTC   973
Manacus vitellinus           TCAAAAGGGATGGAATGCAAATAAAATATTCCAAGCCGCTGAGGCCTTCTTCACCTCCGTTGGCCTTTATAACATGACTG   979
Mus musculus                 GAATCAGGGCTGGGATGCAGAAAGGATATTTCAAGAGGCAGAGAAATTCTTTGTTTCTGTTGGCCTTCCTCATATGACTC   973
Neolamprologus brichardi     GGACAAGGGTTGGACCAAGCTTCAGCTTTTCCAAGAAGCTGAGAAGTTCTTCATGTCTGTGGGCCTCTACAAGATGTTTG   979
Oncorhynchus mykiss          CGCACAGAAATGGCCTAAGGACAGACTGTTCCAAGAAGCAGAGAAGTTTTTCATGTCGGTGGGTCTATACAAGATGTTCG   982
Oreochromis niloticus        GGACAAGGGTTGGACCAAGATTCAGCTTTTCCAAGAAGCTGAGAAGTTCTTCATGTCTGTGGGCCTCTACAAGATGTTTG   979
Poecilia formosa             GCAGCAGGGCTGGGATGAAATGCGTTTCTTTAAGGAAGCAGAGAAGTTCTTTATGTCTGTGGGACTATACGAGATGTTTG   982
Poecilia latipinna           GCAGCAGGGCTGGGATGAAATGCGTTTCTTTAAGGAAGCAGAGAAGTTCTTTATGTCTGTGGGACTATACGAGATGTTTG   982
Pseudopodoces humilis        TGAAAAGAAATGGGATGCAATTAAAATATTCAAATCTGCTGAGGCCTTCTTTGTTTCCATTGGCCTTGATCCCATGACTG   973
Pundamilia nyererei          GGACAAGGGTTGGACCGAGCTTCAGCTTTTCCAAGAAGCTGAGAAGTTCTTCATGTCTGTGGGCCTTTACAAGATGTTTG   979
Pygocentrus nattereri        AGCACAGGGCTGGAAAGAACGGCAGCTGTTTGAAGAAGCAGAGAAGTTCTTTGTGTCTGTGAACATGTCGGCCATGTTTC   970
Python bivittatus            GGAGAAGAAATGGACGGTAGACTCAATATTTAAAGCTGCTGAACATTTTTTTGTCTCAATTGATCTCTTCAATATGACTG   964
Rattus norvegicus            GAATCAGAGCTGGGATGCAGAAAGAATATTTAAAGAGGCAGAGAAGTTCTTCGTTTCTGTTGGCCTTCCTCAAATGACTC   973
Scleropages formosus         CAGTCAGGGCTGGACTGTGAACCAAATGTTCAAAGAAGCAGAGAAGTTTTTCATGTCTGTGAACCTGTACAAGATGTTTG   979
Sinocyclocheilus anshuiensis GGCCCAGGGCTGGGATGAAATCCGCCTGTTTAAAGAGGCAGAAAAATTCTTTATGTTTGTAAACATGTCTGCAATGTTTG   970
Xenopus tropicalis           AGCTCAGGGATGGACGATAGAAAGAATGTTCAAGGAAGCAGAAATTTTCTTCAAGTCTGTTGACCTTTTTGCACTTAATG   979
Lipotes vexillifer           GAACCAGTCGTGGGATGCAAAGAGGATATTCAAGGAGGCTGAGAAGTTCTTTGTGTCTATTGGCCTTCCTAATATGACTC   970
Orcinus orca                 GAACCAGTCCTGGGATGCAAAGAGGATATTCAAGGAGGCTGAGAAGTTCTTTGTGTCTATTGGCCTTCCTAATATGACTC   970
Homo sapiens                 GGACCAGGCCTGGGATGCACAGAGAATATTCAAGGAGGCCGAGAAGTTCTTTGTATCTGTTGGTCTTCCTAATATGACTC   973
```

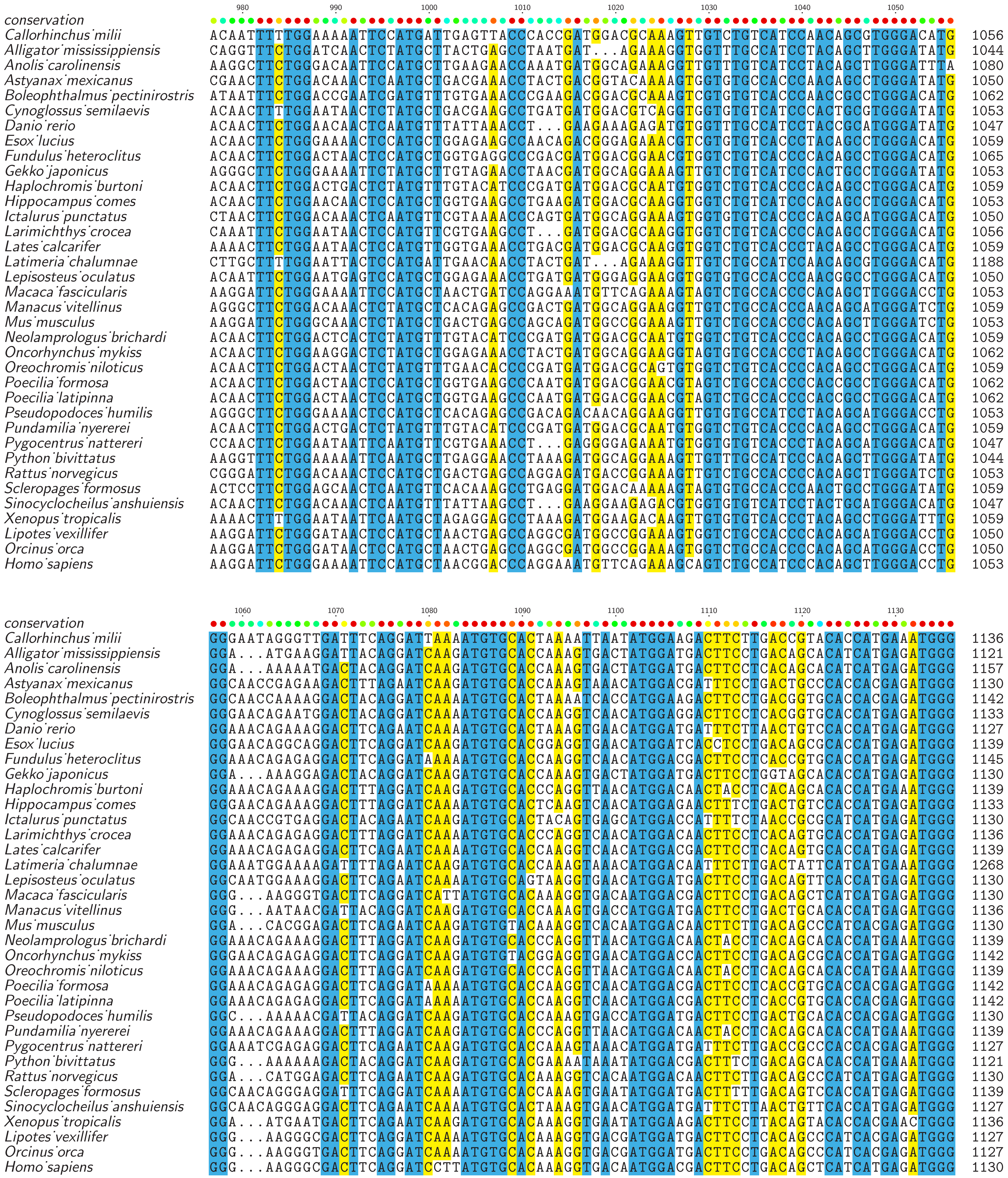

980
990
1000
1010
1020
1030
1040
1050
conservation
Callorhinchus milii 1056
Alligator mississippiensis 1044
Anolis carolinensis 1080
Astyanax mexicanus 1050
Boleophthalmus pectinirostris 1062
Cynoglossus semilaevis 1053
Danio rerio 1047
Esox lucius 1059
Fundulus heteroclitus 1065
Gekko japonicus 1053
Haplochromis burtoni 1059
Hippocampus comes 1053
Ictalurus punctatus 1050
Larimichthys crocea 1056
Lates calcarifer 1059
Latimeria chalumnae 1188
Lepisosteus oculatus 1050
Macaca fascicularis 1053
Manacus vitellinus 1059
Mus musculus 1053
Neolamprologus brichardi 1059
Oncorhynchus mykiss 1062
Oreochromis niloticus 1059
Poecilia formosa 1062
Poecilia latipinna 1062
Pseudopodoces humilis 1053
Pundamilia nyererei 1059
Pygocentrus nattereri 1047
Python bivittatus 1044
Rattus norvegicus 1053
Scleropages formosus 1059
Sinocyclocheilus anshuiensis 1047
Xenopus tropicalis 1059
Lipotes vexillifer 1050
Orcinus orca 1050
Homo sapiens 1053
1060
1070
1080
1090
1100
1110
1120
1130
conservation
Callorhinchus milii 1136
Alligator mississippiensis 1121
Anolis carolinensis 1157
Astyanax mexicanus 1130
Boleophthalmus pectinirostris 1142
Cynoglossus semilaevis 1133
Danio rerio 1127
Esox lucius 1139
Fundulus heteroclitus 1145
Gekko japonicus 1130
Haplochromis burtoni 1139
Hippocampus comes 1133
Ictalurus punctatus 1130
Larimichthys crocea 1136
Lates calcarifer 1139
Latimeria chalumnae 1268
Lepisosteus oculatus 1130
Macaca fascicularis 1130
Manacus vitellinus 1136
Mus musculus 1130
Neolamprologus brichardi 1139
Oncorhynchus mykiss 1142
Oreochromis niloticus 1139
Poecilia formosa 1142
Poecilia latipinna 1142
Pseudopodoces humilis 1130
Pundamilia nyererei 1139
Pygocentrus nattereri 1127
Python bivittatus 1121
Rattus norvegicus 1130
Scleropages formosus 1139
Sinocyclocheilus anshuiensis 1127
Xenopus tropicalis 1136
Lipotes vexillifer 1127
Orcinus orca 1127
Homo sapiens 1130

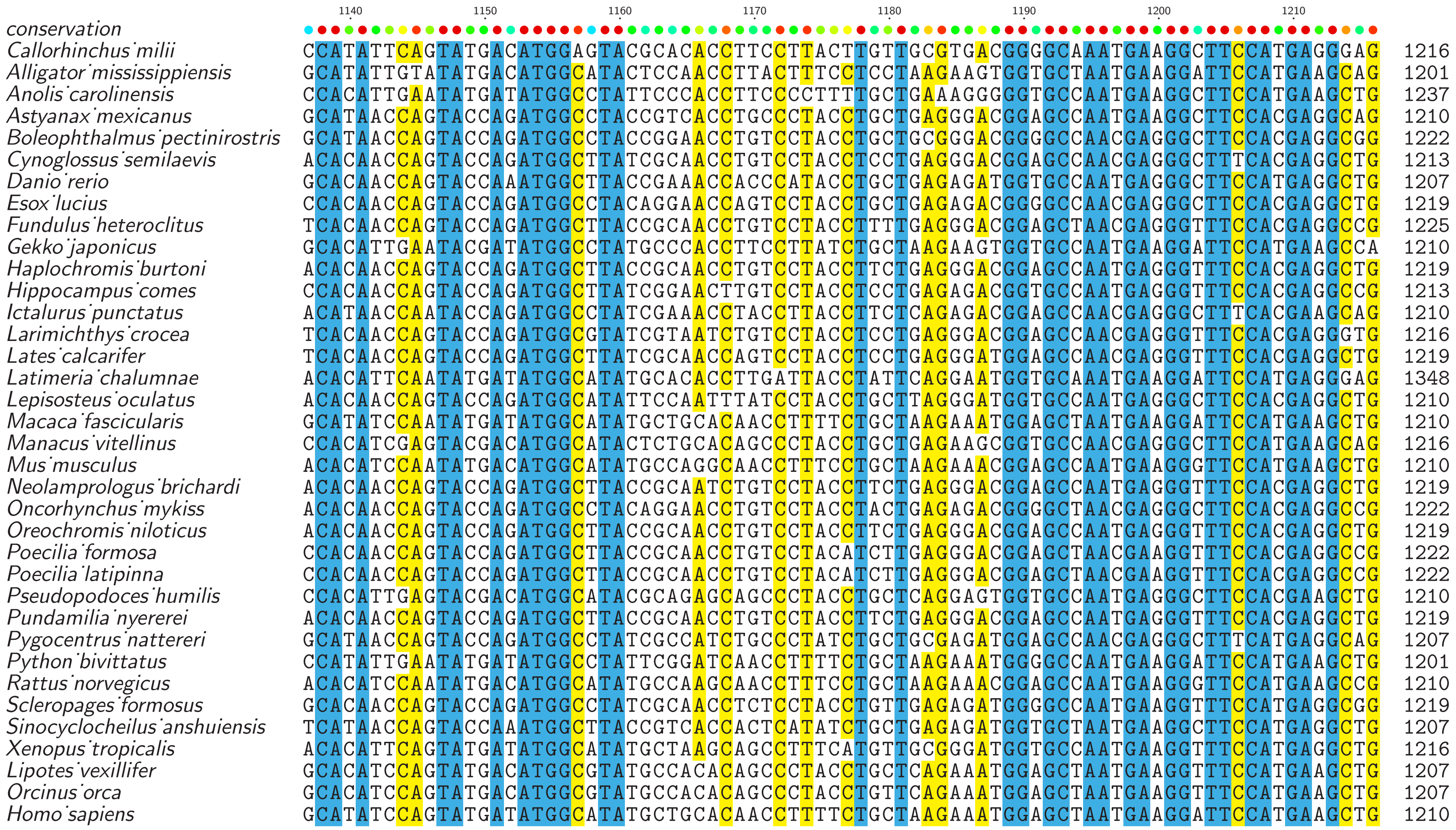
1140
1150
1160
1170
1180
1190
1200
1210
conservation
Callorhinchus milii 1216
Alligator mississippiensis 1201
Anolis carolinensis 1237
Astyanax mexicanus 1210
Boleophthalmus pectinirostris 1222
Cynoglossus semilaevis 1213
Danio rerio 1207
Esox lucius 1219
Fundulus heteroclitus 1225
Gekko japonicus 1210
Haplochromis burtoni 1219
Hippocampus comes 1213
Ictalurus punctatus 1210
Larimichthys crocea 1216
Lates calcarifer 1219
Latimeria chalumnae 1348
Lepisosteus oculatus 1210
Macaca fascicularis 1210
Manacus vitellinus 1216
Mus musculus 1210
Neolamprologus brichardi 1219
Oncorhynchus mykiss 1222
Oreochromis niloticus 1219
Poecilia formosa 1222
Poecilia latipinna 1222
Pseudopodoces humilis 1210
Pundamilia nyererei 1219
Pygocentrus nattereri 1207
Python bivittatus 1201
Rattus norvegicus 1210
Scleropages formosus 1219
Sinocyclocheilus anshuiensis 1207
Xenopus tropicalis 1216
Lipotes vexillifer 1207
Orcinus orca 1207
Homo sapiens 1210

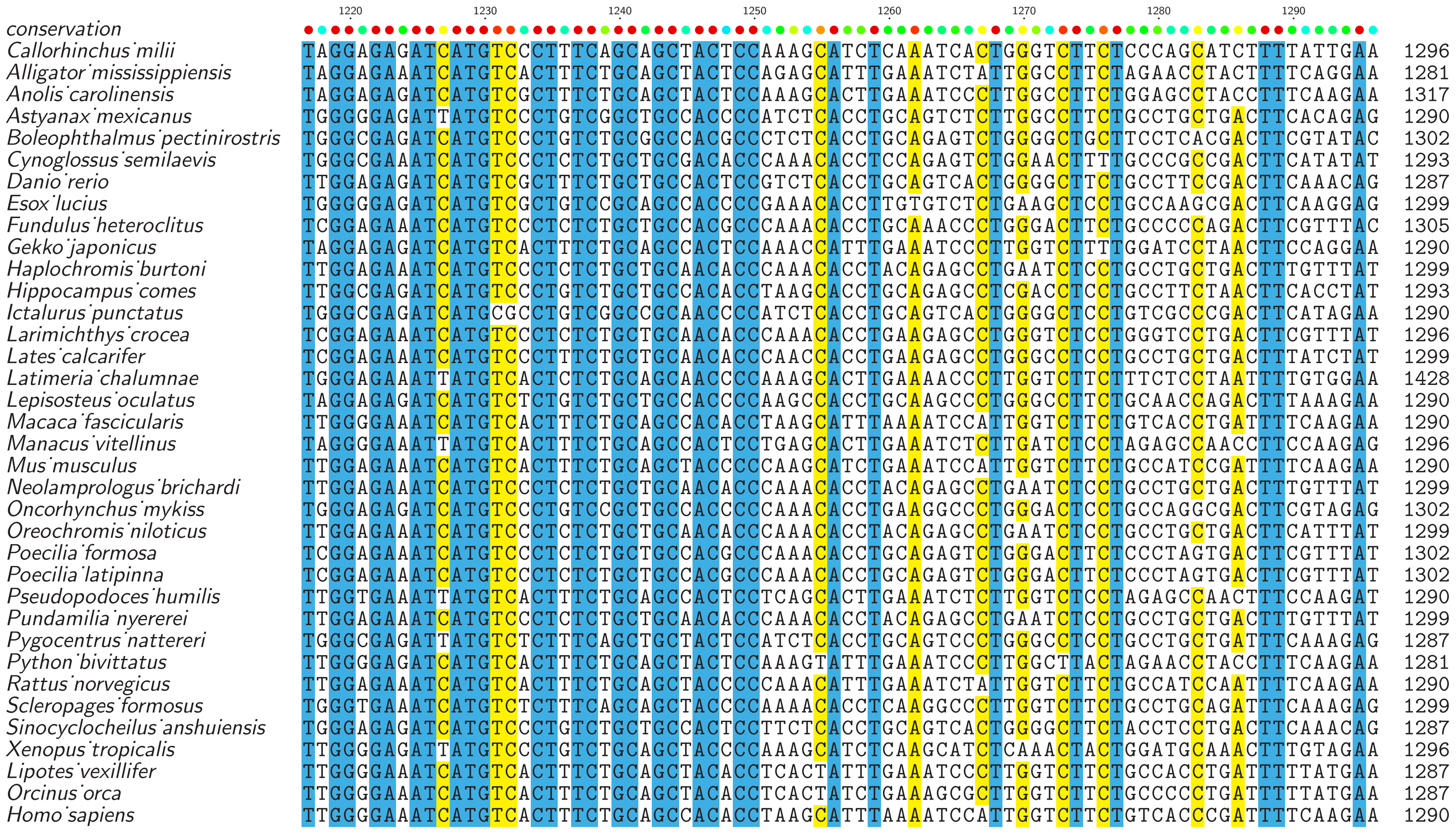
1220
1230
1240
1250
1260
1270
1280
1290
conservation
Callorhinchus milii 1296
Alligator mississippiensis 1281
Anolis carolinensis 1317
Astyanax mexicanus 1290
Boleophthalmus pectinirostris 1302
Cynoglossus semilaevis 1293
Danio rerio 1287
Esox lucius 1299
Fundulus heteroclitus 1305
Gekko japonicus 1290
Haplochromis burtoni 1299
Hippocampus comes 1293
Ictalurus punctatus 1290
Larimichthys crocea 1296
Lates calcarifer 1299
Latimeria chalumnae 1428
Lepisosteus oculatus 1290
Macaca fascicularis 1290
Manacus vitellinus 1296
Mus musculus 1290
Neolamprologus brichardi 1299
Oncorhynchus mykiss 1302
Oreochromis niloticus 1299
Poecilia formosa 1302
Poecilia latipinna 1302
Pseudopodoces humilis 1290
Pundamilia nyererei 1299
Pygocentrus nattereri 1287
Python bivittatus 1281
Rattus norvegicus 1290
Scleropages formosus 1299
Sinocyclocheilus anshuiensis 1287
Xenopus tropicalis 1296
Lipotes vexillifer 1287
Orcinus orca 1287
Homo sapiens 1290

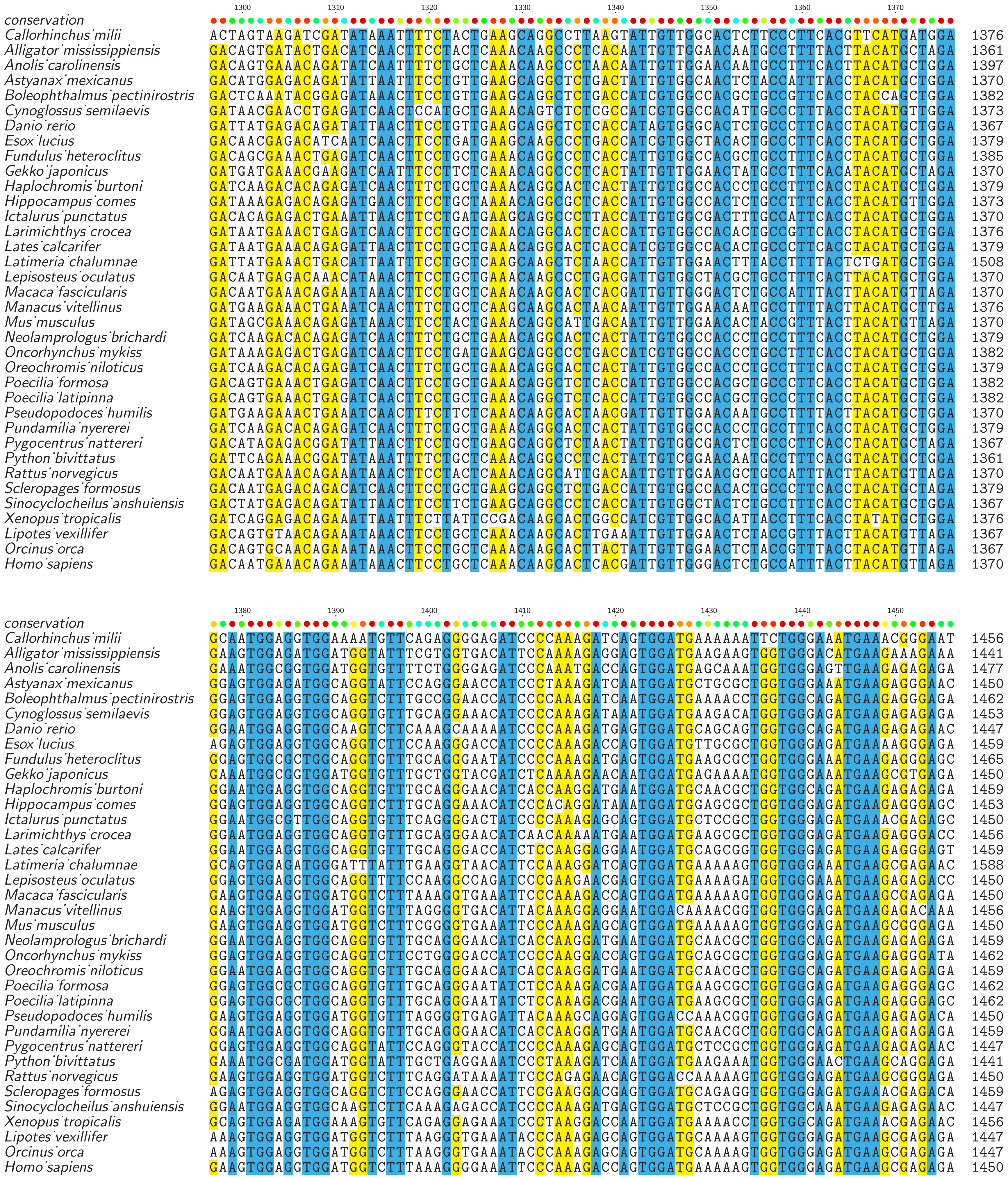

1300 1310 1320 1330 1340 1350 1360 1370
conservation
Callorhinchus milii 1376
Alligator mississippiensis 1361
Anolis carolinensis 1397
Astyanax mexicanus 1370
Boleophthalmus pectinirostris 1382
Cynoglossus semilaevis 1373
Danio rerio 1367
Esox lucius 1379
Fundulus heteroclitus 1385
Gekko japonicus 1370
Haplochromis burtoni 1379
Hippocampus comes 1373
Ictalurus punctatus 1370
Larimichthys crocea 1376
Lates calcarifer 1379
Latimeria chalumnae 1508
Lepisosteus oculatus 1370
Macaca fascicularis 1370
Manacus vitellinus 1376
Mus musculus 1370
Neolamprologus brichardi 1379
Oncorhynchus mykiss 1382
Oreochromis niloticus 1379
Poecilia formosa 1382
Poecilia latipinna 1382
Pseudopodoces humilis 1370
Pundamilia nyererei 1379
Pygocentrus nattereri 1367
Python bivittatus 1361
Rattus norvegicus 1370
Scleropages formosus 1379
Sinocyclocheilus anshuiensis 1367
Xenopus tropicalis 1376
Lipotes vexillifer 1367
Orcinus orca 1367
Homo sapiens 1370
1380 1390 1400 1410 1420 1430 1440 1450
conservation
Callorhinchus milii 1456
Alligator mississippiensis 1441
Anolis carolinensis 1477
Astyanax mexicanus 1450
Boleophthalmus pectinirostris 1462
Cynoglossus semilaevis 1453
Danio rerio 1447
Esox lucius 1459
Fundulus heteroclitus 1465
Gekko japonicus 1450
Haplochromis burtoni 1459
Hippocampus comes 1453
Ictalurus punctatus 1450
Larimichthys crocea 1456
Lates calcarifer 1459
Latimeria chalumnae 1588
Lepisosteus oculatus 1450
Macaca fascicularis 1450
Manacus vitellinus 1456
Mus musculus 1450
Neolamprologus brichardi 1459
Oncorhynchus mykiss 1462
Oreochromis niloticus 1459
Poecilia formosa 1462
Poecilia latipinna 1462
Pseudopodoces humilis 1450
Pundamilia nyererei 1459
Pygocentrus nattereri 1447
Python bivittatus 1441
Rattus norvegicus 1450
Scleropages formosus 1459
Sinocyclocheilus anshuiensis 1447
Xenopus tropicalis 1456
Lipotes vexillifer 1447
Orcinus orca 1447
Homo sapiens 1450

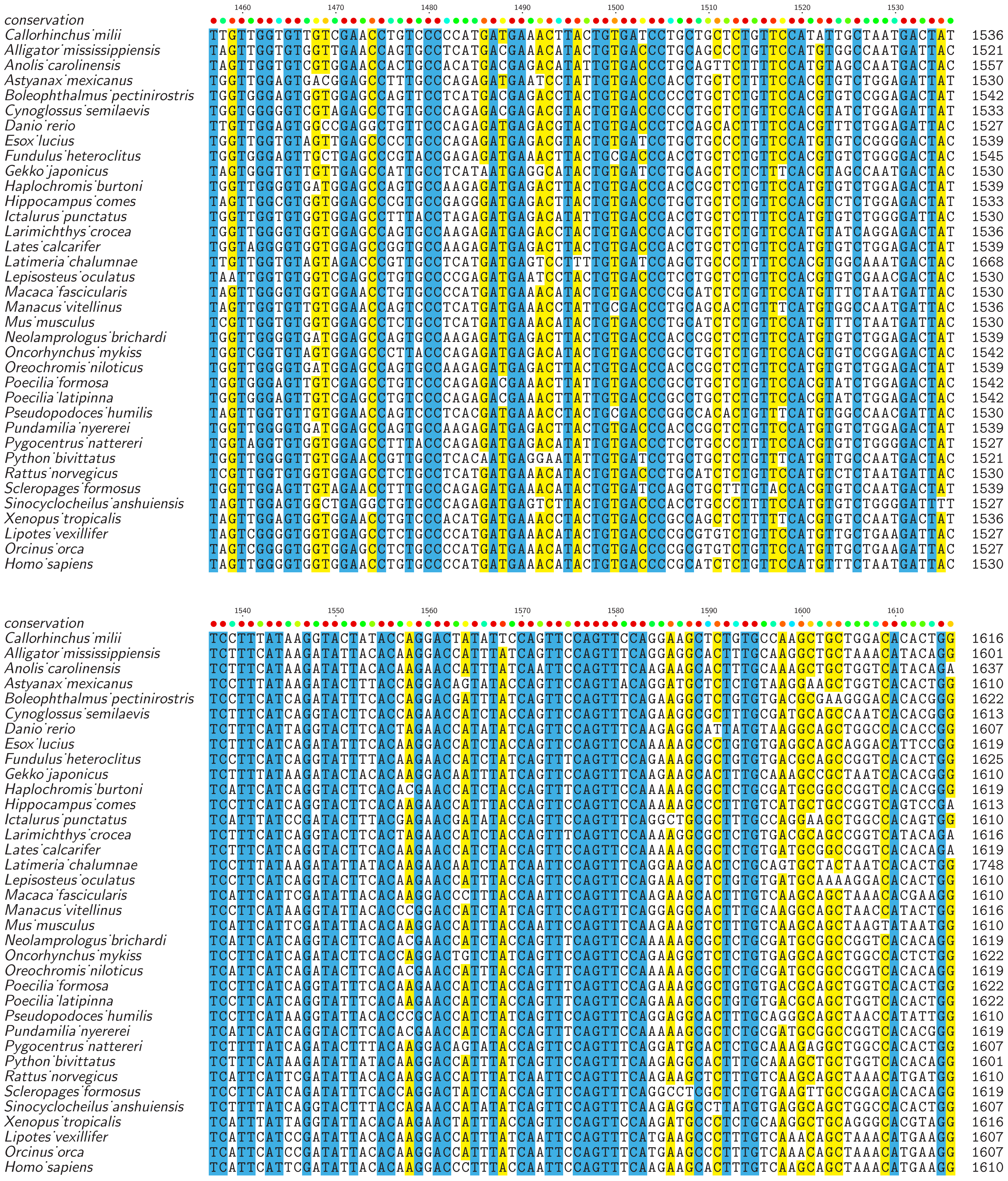

conservation
1460 1470 1480 1490 1500 1510 1520 1530
Callorhinchus milii 1536
Alligator mississippiensis 1521
Anolis carolinensis 1557
Astyanax mexicanus 1530
Boleophthalmus pectinirostris 1542
Cynoglossus semilaevis 1533
Danio rerio 1527
Esox lucius 1539
Fundulus heteroclitus 1545
Gekko japonicus 1530
Haplochromis burtoni 1539
Hippocampus comes 1533
Ictalurus punctatus 1530
Larimichthys crocea 1536
Lates calcarifer 1539
Latimeria chalumnae 1668
Lepisosteus oculatus 1530
Macaca fascicularis 1530
Manacus vitellinus 1536
Mus musculus 1530
Neolamprologus brichardi 1539
Oncorhynchus mykiss 1542
Oreochromis niloticus 1539
Poecilia formosa 1542
Poecilia latipinna 1542
Pseudopodoces humilis 1530
Pundamilia nyererei 1539
Pygocentrus nattereri 1527
Python bivittatus 1521
Rattus norvegicus 1530
Scleropages formosus 1539
Sinocyclocheilus anshuiensis 1527
Xenopus tropicalis 1536
Lipotes vexillifer 1527
Orcinus orca 1527
Homo sapiens 1530
conservation
1540 1550 1560 1570 1580 1590 1600 1610
Callorhinchus milii 1616
Alligator mississippiensis 1601
Anolis carolinensis 1637
Astyanax mexicanus 1610
Boleophthalmus pectinirostris 1622
Cynoglossus semilaevis 1613
Danio rerio 1607
Esox lucius 1619
Fundulus heteroclitus 1625
Gekko japonicus 1610
Haplochromis burtoni 1619
Hippocampus comes 1613
Ictalurus punctatus 1610
Larimichthys crocea 1616
Lates calcarifer 1619
Latimeria chalumnae 1748
Lepisosteus oculatus 1610
Macaca fascicularis 1610
Manacus vitellinus 1616
Mus musculus 1610
Neolamprologus brichardi 1619
Oncorhynchus mykiss 1622
Oreochromis niloticus 1619
Poecilia formosa 1622
Poecilia latipinna 1622
Pseudopodoces humilis 1610
Pundamilia nyererei 1619
Pygocentrus nattereri 1607
Python bivittatus 1601
Rattus norvegicus 1610
Scleropages formosus 1619
Sinocyclocheilus anshuiensis 1607
Xenopus tropicalis 1616
Lipotes vexillifer 1607
Orcinus orca 1607
Homo sapiens 1610

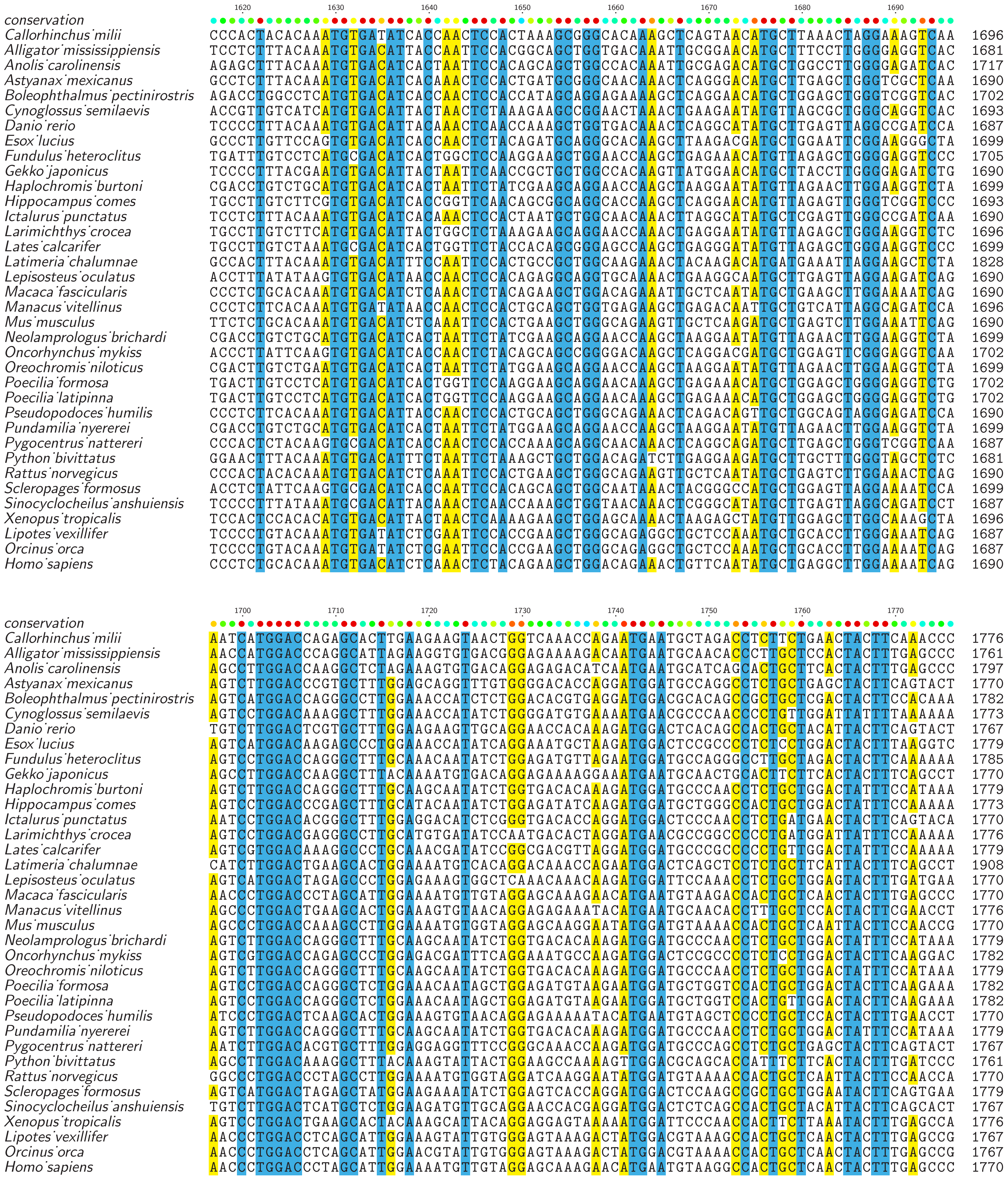
1620 1630 1640 1650 1660 1670 1680 1690
conservation
Callorhinchus milii CCCACTACACAAATGTGATATCACCAACTCCACTAAAGCGGGCACAAAGCTCAGTAACATGCTTAAACTAGGAAAGTCAA 1696
Alligator mississippiensis TCCTCTTTACAAATGTGACATTACCAATTCCACGGCAGCTGGTGACAAATTGCGGAACATGCTTTCCTTGGGGAGGTCAC 1681
Anolis carolinensis AGAGCTTTACAAATGTGACATCACTAATTCCACAGCAGCTGGCCACAAATTGCGAGACATGCTGGCCTTGGGGAGATCAC 1717
Astyanax mexicanus GCCTCTTTACAAATGTGACATCACAAACTCCACTGATGCGGGCAACAAACTCAGGGACATGCTTGAGCTGGGTCGCTCAA 1690
Boleophthalmus pectinirostris AGACCTGGCCTCATGTGACATCACCAACTCCACCATAGCAGGAGAAAAGCTCAGGAACATGCTGGAGCTGGGTCGGTCAC 1702
Cynoglossus semilaevis ACCGTTGTCATCATGTGACATTACTAACTCTAAAGAAGCCGGAACTAAACTGAAGAATATGTTAGCGCTGGGCAGGTCAC 1693
Danio rerio TCCCCTTTACAAATGTGACATTACAAACTCAACCAAAGCTGGTGACAAACTCAGGCATATGCTTGAGTTAGGCCGATCCA 1687
Esox lucius GCCCTTGTTCCAGTGTGACATCACCAACTCTACAGATGCAGGGCACAAGCTTAAGACGATGCTGGAATTCGGAAGGGCTA 1699
Fundulus heteroclitus TGATTTGTCCTCATGCGACATCACTGGCTCCAAGGAAGCTGGAACCAAGCTGAGAAACATGCTTAGAGCTGGGGAGGTCCC 1705
Gekko japonicus TCCCCTTTACGAATGTGACATTACTAATTCAACCGCTGCTGGCCACAAGTTATGGAACATGCTTACCTTGGGGAGATCTG 1690
Haplochromis burtoni CGACCTGTCTGCATGTGACATCACTAATTCTATCGAAGCAGGAACCAAGCTAAGGAATATGTTAGAACTTGGAAGGTCTA 1699
Hippocampus comes TGCCTTGTCTTCGTGTGACATCACCGGTTCAACAGCGGCAGGCACCAAGCTCAGGAACATGTTAGAGTTGGGTCGGTCCC 1693
Ictalurus punctatus TCCTCTTTACAAATGTGACATCACAAACTCCACTAATGCTGGCAACAAACTTAGGCATATGCTCGAGTTGGGCCGATCAA 1690
Larimichthys crocea TGCCTTGTCTTCATGTGACATTACTGGCTCTAAAGAAGCAGGAACCAAACTGAGGAATATGTTAGAGCTGGGAAGGTCTC 1696
Lates calcarifer TGCCTTGTCTAAATGCGACATCACTGGTTCTACCACAGCGGGAGCCAAGCTGAGGGATATGTTAGAGCTGGGAAGGTCCC 1699
Latimeria chalumnae GCCACTTTACAAATGTGACATTTCCAATTCCACTGCCGCTGGCAAGAAACTACAAGACATGATGAAATTAGGAAGCTCTA 1828
Lepisosteus oculatus ACCTTTATATAAGTGTGACATCATAACCAACTCCACAGAGGCAGGTGCAAAACTGAAGGCAATGCTTGAGTTAGGAAGATCAG 1690
Macaca fascicularis CCCTCTGCACAAATGTGACATCTCAAACTCTACAGAAGCTGGACAGAAATTGCTCAATATGCTGAAGCTTGGAAAATCAG 1690
Manacus vitellinus CCCTCTTCACAAATGTGATATAACCAACTCCACTGCAGCTGGTGAGAAGCTGAGACAATTGCTGTCATTAGGCAGATCCA 1696
Mus musculus TTCTCTGCACAAATGTGACATCTCAAATTCCACTGAAGCTGGGCAGAAGTTGCTCAAGATGCTGAGTCTTGGAAATTCAG 1690
Neolamprologus brichardi CGACCTGTCTGCATGTGACATCACTAATTCTATCGAAGCAGGAACCAAGCTAAGGAATATGTTAGAACTTGGAAGGTCTA 1699
Oncorhynchus mykiss ACCCTTATTCAAGTGTGACATCACCAACTCTACAGCAGCCGGGGACAAGCTCAGGACGATGCTGGAGTTCGGGAGGTCAA 1702
Oreochromis niloticus CGACTTGTCTGAATGTGACATCACTAATTCTATGGAAGCAGGAACCAAGCTAAGGAATATGTTAGAACTTGGAAGGTCTA 1699
Poecilia formosa TGACTTGTCCTCATGTGACATCACTGGTTCCAAGGAAGCAGGAACAAAGCTGAGAAACATGCTGGAGCTGGGGAGGTCTG 1702
Poecilia latipinna TGACTTGTCCTCATGTGACATCACTGGTTCCAAGGAAGCAGGAACAAAGCTGAGAAACATGCTGGAGCTGGGGAGGTCTG 1702
Pseudopodoces humilis CCCTCTTCACAAATGTGACATTACCAACTCCACTGCAGCTGGGCAGAAACTCAGACAGTTGCTGGCAGTAGGGAGATCCA 1690
Pundamilia nyererei CGACCTGTCTGCATGTGACATCACTAATTCTATGGAAGCAGGAACCAAGCTAAGGAATATGTTAGAACTTGGAAGGTCTA 1699
Pygocentrus nattereri CCCACTCTACAAGTGCGACATCACCAACTCCACCAAAGCAGGCAACAAACTCAGGCAGATGCTTGAGCTGGGTCGGTCAA 1687
Python bivittatus GGAACTTTACAAATGTGACATTTCTAATTCTAAAGCTGCTGGACAGATCTTGAGGAAGATGCTTGCTTTGGGTAGCTCTC 1681
Rattus norvegicus CCCACTACACAAATGTGACATCTCAAATTCCACTGAAGCTGGGCAGAAGTTGCTCAATATGCTGAGTCTTGGAAACTCAG 1690
Scleropages formosus ACCTCTATTCAAGTGCGACATCACCAATTCCACAGCAGCTGGCAATAAACTACGGGCCATGCTGGAGTTGGAAAATCCA 1699
Sinocyclocheilus anshuiensis TCCCCTTTATAAATGCGACATTACAAACTCAACCAAAGCTGGTAACAAACTCGGGCATATGCTTGAGTTAGGCAGATCCT 1687
Xenopus tropicalis TCCACTCCACACATGTGACATTACTAACTCAAAAGAAGCTGGAGCAAAACTAAGAGCTATGTTGGAGCTTGGCAAAGCTA 1696
Lipotes vexillifer TCCCCTGTACAAATGTGATATCTCGAATTCCACCGAAGCTGGGCAGAGGCTGCTCCAAATGCTGCACCTTGGGAAATCAG 1687
Orcinus orca TCCCCTGTACAAATGTGATATCTCGAATTCCACCGAAGCTGGGCAGAGGCTGCTCCAAATGCTGCACCTTGGAAAATCAG 1687
Homo sapiens CCCTCTGCACAAATGTGACATCTCAAACTCTACAGAAGCTGGACAGAAACTGTTCAATATGCTGAGGCTTGGAAAATCAG 1690
1700 1710 1720 1730 1740 1750 1760 1770
conservation
Callorhinchus milii AATCATGGACCAGAGCACTTGAAGAAGTAACTGGTCAAACCAGAATGAATGCTAGACCTCTTCTGAACTACTTCAAACCC 1776
Alligator mississippiensis AACCATGGACCCAGGCATTAGAAGGTGTGACGGGAGAAAAGACAATGAATGCAACACCCTTGCTCCACTACTTTGAGCCC 1761
Anolis carolinensis AGCCTTGGACCAAGGCTCTAGAAAGTGTGACAGGAGAGACATCAATGAATGCATCAGCACTGCTTCACTACTTTGAGCCC 1797
Astyanax mexicanus AGTCTTGGACCCGTGCTTTGGAGCAGGTTGTGGGGACACCAGGATGGATGCCAGGCCTCTGCTGAGCTACTTCAGTACT 1770
Boleophthalmus pectinirostris AGTCATGGACCAGGGCCTTGGAAACCATCTCTGGACACGTGAGGATGGACGCACAGCCGCTGCTCGACTACTTCCACAAA 1782
Cynoglossus semilaevis AGTCCTGGACAAAGGCTTTGGAAACCATATCTGGGATGTGAAAATGAACGCCCAACCCCTGTTGGATTATTTAAAAAA 1773
Danio rerio TGTCTTGGACTCGTGCTTTGGAAGAAGTTGCAGGAACCACAAAGATGGACTCACAGCCACTGCTACATTACTTCAGTACT 1767
Esox lucius AGTCATGGACAAGAGCCCTGGAAACCATATCAGGAAATGCTAAGATGGACTCCGCCCCTCTCCTGGACTACTTTAAGGTC 1779
Fundulus heteroclitus AGTCCTGGACCAGGGCTTTGCAAACAATATCTGGAGATGTTAGAATGGATGCCAGGGCCTTGCTAGACTACTTCAAAAAA 1785
Gekko japonicus AGCCTTGGACCAAGGCTTTACAAAATGTGACAGGAGAAAAGGAAATGAATGCAACTGCACTTCTTCACTACTTTCAGCCT 1770
Haplochromis burtoni AGTCTTGGACCAGGGCTTTGCAAGCAATATCTGGTGACACAAAGATGGATGCCCAACCTCTGCTGGACTATTTCCATAAA 1779
Hippocampus comes AGTCCTGGACCCGAGCTTTGCATACAATATCTGGAGATATCAAGATGGATGCTGGGCCACTGCTGGACTATTTCCAAAAA 1773
Ictalurus punctatus AATCCTGGACACGGGCTTTGGAGGACATCTCGGGTGACACCAGGATGGACTCCCAACCTCTGATGAACTACTTCAGTACA 1770
Larimichthys crocea AGTCCTGGACGAGGGCCTTGCATGTGATATCCAATGACACTAGGATGAACGCCGGCCCCCTGATGGATTATTTCCAAAAA 1776
Lates calcarifer AGTCGTGGACAAAGGCCCTGCAAACGATATCCGGCGACGTTAGGATGGATGCCCGCCCCCTGTTGGACTATTTCCAAAAA 1779
Latimeria chalumnae CATCTTGGACTGAAGCACTGGAAAATGTCACAGGACAAACCAGAATGGACTCAGCTCCTCTGCTTCATTACTTTCAGCCT 1908
Lepisosteus oculatus AGTCATGGACTAGAGCCCTGGAGAAAGTGGCTCAAACAAACAAGATGGATTCCAAACCTCTGCTGGAGTACTTTGATGAA 1770
Macaca fascicularis AACCCTGGACCCTAGCATTGGAAAATGTTGTAGGAGCAAAGAACATGAATGTAAGACCACTGCTCAACTACTTTGAGCCC 1770
Manacus vitellinus AGCCCTGGACTGAAGCACTGGAAAGTGTAACAGGAGAGAAATACATGAATGCAACACCCTTTGCTCCACTACTTCGAACCT 1776
Mus musculus AGCCCTGGACCAAAGCCTTGGAAAATGTGGTAGGAGCAAGGAATATGGATGTAAAACCACTGCTCAATTACTTCCAACCG 1770
Neolamprologus brichardi AGTCTTGGACCAGGGCTTTGCAAGCAATATCTGGTGACACAAAGATGGATGCCCAACCTCTGCTGGACTATTTCCATAAA 1779
Oncorhynchus mykiss AGTCGTGGACCAGAGCCCTGGAGACGATTTCAGGAAATGCCAAGATGGACTCCGCCCCTCTCCTGGACTACTTCAAGGAC 1782
Oreochromis niloticus AGTCTTGGACCAGGGCTTTGCAAGCAATATCTGGTGACACAAAGATGGATGCCCAACCTCTGCTGGACTATTTCCATAAA 1779
Poecilia formosa AGTCCTGGACCAGGGCTCTGGAAACAATAGCTGGAGATGTAAGAATGGATGCTGGTCCACTGCTGGACTACTTCAAGAAA 1782
Poecilia latipinna AGTCCTGGACCAGGGCTCTGGAAACAATAGCTGGAGATGTAAGAATGGATGCTGGTCCACTGTTGGACTACTTCAAGAAA 1782
Pseudopodoces humilis ATCCCTGGACTCAAGCACTGGAAAGTGTAACAGGAGAAAAAATACATGAATGTAGCTCCCCTGCTCCACTACTTTGAACCT 1770
Pundamilia nyererei AGTCTTGGACCAGGGCTTTGCAAGCAATATCTGGTGACACAAAGATGGATGCCCAACCTCTGCTGGACTATTTCCATAAA 1779
Pygocentrus nattereri AATCTTGGACACGTGCTTTGGAGGAGGTTCCGGGCAAACCAAGATGGATGCCCAGCCTCTGCTGAGCTACTTCAGTACT 1767
Python bivittatus AGCCTTGGACAAAGGCTTTACAAAGTATTACTGGAAGCCAAAAGTTGGACGCAGCACCATTTCTTCACTACTTTGATCCC 1761
Rattus norvegicus GGCCCTGGACCCTAGCCTTGGAAAATGTGGTAGGATCAAGGAATATGGATGTAAAACCACTGCTCAATTACTTCCAACCA 1770
Scleropages formosus AGTCATGGACTAGAGCTATGGAAGAAATATCTGGAGTCACCAGGATGGACTCCAAGCCGCTGCTGGAATACTTCAGTGAA 1779
Sinocyclocheilus anshuiensis TGTCTTGGACTCATGCTCTGGAAGATGTTGCAGGAACCACGAGGATGGACTCTCAGCCACTGCTACATTACTTCAGCACT 1767
Xenopus tropicalis AGTCCTGGACTGAAGCACTACAAAGCATTACAGGAGGAGTAAAAATGGATTCCCAACCACTTCTTAAATACTTTGAGCCA 1776
Lipotes vexillifer AACCCTGGACCTCAGCATTGGAAAGTATTGTGGGAGTAAAGACTATGGACGTAAAGCCACTGCTCAACTACTTTGAGCCG 1767
Orcinus orca AACCCTGGACCTCAGCATTGGAACGTATTGTGGGAGTAAAGACTATGGACGTAAAACCACTGCTCAACTACTTTGAGCCG 1767
Homo sapiens AACCCTGGACCCTAGCATTGGAAAATGTTGTAGGAGCAAAGAACATGAATGTAAGGCCACTGCTCAACTACTTTGAGCCC 1770

```
                              1780          1790          1800          1810          1820          1830          1840
conservation
Callorhinchus milii           TTGTATGAGTGGCTGAAGAAAGATAACCAGGACAAAGGCAGACATGTTGGTTGGGACCCCACCTGGACTCCA........ 1848
Alligator mississippiensis    TTATATCAGTGGCTGCAAAAGAATAACACCAAT......AGGCGCATTGGCTGGAATACAACCTGGACACCA........ 1827
Anolis carolinensis           TTGTATCAGTGGCTAATAAAGAACAACACTGGC......AGATCTCTTGAGTGGGATACAGCCTGGCGTCCA........ 1863
Astyanax mexicanus            CTGTATGAATGGCTTAAGGAGGAAAATCAGAAGAACAACAGGGCAAGTGGGTGGAGTCTGAGTGATGATCCA........ 1842
Boleophthalmus pectinirostris CTCTTTGTGTGGCTCCAAGAGGACAACAAGAAACATGGCCGTATTGTGGGCTGGAACCCCACCATAGACCCT........ 1854
Cynoglossus semilaevis        CTGTACGAATGGCTGAAGGAAGAAAATAGGAAACACGGCAGGACTGTAGGCTGGGACCCAAGCATTGACCCA........ 1845
Danio rerio                   CTCATGGAGTGGTTGAAGGAGGAAAACCAGAAGAACAACAGAGTTCCTGGCTGGAACGTCAATGTTAACCCTGGTGTACT 1847
Esox lucius                   CTGTATGATTGGCTAATAAAAGAAAACAGGAAGAACAATAGGAAGCCAGGATGGAAAGCAACTGAGGACCCA........ 1851
Fundulus heteroclitus         CTTTACGACTGGTTGAAGCAGGATAACCTGAAACACAACAGGGCTGTAGGCTGGAGGACAACAGCGGATCCA........ 1857
Gekko japonicus               TTATACGAGTGGCTGATAGAGAACAATGCCAAC......AGATTTGTTGGTTGGGATACAGCCTGGTCTCCA........ 1836
Haplochromis burtoni          CTTTATGAGTGGTTGCAGGCAGATAACAAGAAACGCAACAGGGTGGTAGGCTGGAGTGAA......GATCAA........ 1845
Hippocampus comes             CTTTACGTTTGGCTGAAGGAGGACAACATGAAACATAACCGCATTGTTGGCTGGGATGCAGCAGTAGATCCA........ 1845
Ictalurus punctatus           CTGTATGACTGGCTTAAGGCTGAAAACCAGAAAAACAACAGACAACCAGGGTGGGATGCTGCTATCGATCCA........ 1842
Larimichthys crocea           CTTTATGACTGGCTGAAGGTAGAGAATAAGAAACACAATCGGACGGTAGGCTGGAAAACAAACATAGATCCA........ 1848
Lates calcarifer              CTTCATGACTGGCTGAAGGCAGAGAACAAGAAACACAATAGAACTGTAGGCTGGAAGACAGCAGTAGATCCA........ 1851
Latimeria chalumnae           TTGTATGAGTGGCTGAAAAAGAACAATGCGGAAAACGGGAGAGTTGTTGGCTGGAATCAATTCTGCACTCCA........ 1980
Lepisosteus oculatus          CTATACAGATGGTTACAAAATCAGAATCAAGAGAACAACAGGAATATTGGCTGGACAAAGCATGGGACCCA........ 1842
Macaca fascicularis           TTGTTTACCTGGCTGAAAGACCAGAACAAGAAT......TCTTTTGTGGGATGGAGTACCGACTGGAGTCCG........ 1836
Manacus vitellinus            TTATATGAATGGCTGACAAAAAACAATTCTGGA......AGATACATTGGTTGGAAAACAGACTGGGCTCCA........ 1842
Mus musculus                  TTGTTTGACTGGCTGAAAGAGCAGAACAGAAAT......TCTTTTGTGGGGTGGAACACTGAATGGAGCCCA........ 1836
Neolamprologus brichardi      CTTTATGAGTGGTTGCAGGCACATAACAAGAAACACAACCTGGTTGTAGGCTGGAGTGAA......GATCCA........ 1845
Oncorhynchus mykiss           CTCCACGTTTGGCTCATAGAAGAAAACAGGAAGAACAACAGGAAGCCAGGATGGAGAGCAGCTGAGGACCCA........ 1854
Oreochromis niloticus         CTTTATGAGTGGTTGCAGGCAGATAACAAGAAACACAACAGGGTGGTAGGCTGGAGTGAA......GATCCA........ 1845
Poecilia formosa              CTGTATGACTGGCTGAAAGAGAATAACCAGAAACACAGCAGAGCTGTTGGCTGGAAGACAACAGTGGATCCA........ 1854
Poecilia latipinna            CTGTATGACTGGCTGAAAGAGAATAACCAGAAACACAGCAGAGCTGTTGGCTGGAAGACAACAGTGGATCCA........ 1854
Pseudopodoces humilis         TTATATGAGTGGCTGAAAAGGAACAATTCTGGC......AGATTCATTGGCTGGAAAACAGACTGGGCTCCA........ 1836
Pundamilia nyererei           CTTTATGAGTGGTTGCAGGCAGATAACAAGAAACACAACAGGGTGGTAGGCTGGAGTGAA......GCTCAA........ 1845
Pygocentrus nattereri         CTGTACACCTGGCTCAAGGATGAAAATCAGAAGAACAACAGGCAGCCAGGATGGAGCGTGGCTGTTGATCCA........ 1839
Python bivittatus             TTATTAAAGTGGCTGGAGAAAAACAACAGTGAT......GAAAGTGTTGGTTGGAATACAAACTGGACTCCA........ 1827
Rattus norvegicus             TTGTTTGTCTGGCTGAAAGAGCAGAACAGGAAT......TCGACTGTGGGGTGGAGCACTGACTGGAGCCCA........ 1836
Scleropages formosus          CTCTACAGTTGGTTACAAGAAGACAATCGAAAGAACAACAGGCAACCAGGGTGGAAGACCACGGAGGATCCA........ 1851
Sinocyclocheilus anshuiensis  CTCATGGACTGGCTAAAGGAGGAGAACCAGAAACAACAACAGAGTGCCTGGCTGGGATGTCAATATTAACCCA........ 1839
Xenopus tropicalis            TTATTTGTATGGCTACAGAAGAATAATGAGGAAAATAGAAGACAGAGTACCTGGAACACAGCATGGTCTCCA........ 1848
Lipotes vexillifer            TTGTTGACCTGGCTGAAAGACCAGAACAGGAAT......TCGTTTGTGGGATGGAGCACAGACTGGACTCCA........ 1833
Orcinus orca                  TTGTTGACCTGGCTGAAAGAGCAGAACAGGAAT......TCATTTGTGGGATGGAGAACAGACTGGACTCCA........ 1833
Homo sapiens                  TTATTTACCTGGCTGAAAGACCAGAACAAGAAT......TCTTTTGTGGGATGGAGTACCGACTGGAGTCCA........ 1836

                                                          1850          1860          1870          1880          1890          1900
conservation
Callorhinchus milii           ............................TACGCAGATAGACACCACGTTGACCTAATCAGTAAATCAGATGAAGAGCAAT 1900
Alligator mississippiensis    ...............................TATGCTGCC........................................ 1836
Anolis carolinensis           ...............................TATGCTGAT........................................ 1872
Astyanax mexicanus            ............................TACTCCAATGATGCC..................................... 1857
Boleophthalmus pectinirostris ............................TTTTCGGAAAATGGC..................................... 1869
Cynoglossus semilaevis        ............................TACTCAGAGAATGCA..................................... 1860
Danio rerio                   TACATCATCTTTTATAAATGATGCAGAAATTTCAGAAAATGCC..................................... 1890
Esox lucius                   ............................TACTCAAATGATGCC..................................... 1866
Fundulus heteroclitus         ............................AATTCAGAACATGCC..................................... 1872
Gekko japonicus               ...............................TATGTCAAC........................................ 1845
Haplochromis burtoni          ............................TATCCAGAATATTCA..................................... 1860
Hippocampus comes             ............................TATTCTGATTATGGC..................................... 1860
Ictalurus punctatus           ............................TACTCTGAAAATGCT..................................... 1857
Larimichthys crocea           ............................TATTCAAAGTATGCC..................................... 1863
Lates calcarifer              ............................TATTCACAGTATGCC..................................... 1866
Latimeria chalumnae           ............................TATAAAAGGAAGAAT..................................... 1995
Lepisosteus oculatus          ............................TATTCAGAGAATGCA..................................... 1857
Macaca fascicularis           ...............................TATGCTGAC........................................ 1845
Manacus vitellinus            ...............................TATTCCAGT........................................ 1851
Mus musculus                  ...............................TATGCCGAC........................................ 1845
Neolamprologus brichardi      ............................TATCCAGAATATTCA..................................... 1860
Oncorhynchus mykiss           ............................TTCTCAGCGAATGCC..................................... 1869
Oreochromis niloticus         ............................TATCCAGAATATTCA..................................... 1860
Poecilia formosa              ............................TATTCACAATATGCC..................................... 1869
Poecilia latipinna            ............................TATTCACAATATGCC..................................... 1869
Pseudopodoces humilis         ...............................TATTATAGC........................................ 1845
Pundamilia nyererei           ............................TATCCAGAATATTCA..................................... 1860
Pygocentrus nattereri         ............................TACTCCAACGATGCC..................................... 1854
Python bivittatus             ...............................TATTCTAAG........................................ 1836
Rattus norvegicus             ...............................TATGCCGAC........................................ 1845
Scleropages formosus          ............................TATGCCAATGATGCC..................................... 1866
Sinocyclocheilus anshuiensis  .ACATCCTCTGTAAATAAAGAT.........GATTCAAATGCA..................................... 1872
Xenopus tropicalis            ...............................TTCTCAGAGGCT..................................... 1860
Lipotes vexillifer            ...............................TATTCTGAC........................................ 1842
Orcinus orca                  ...............................TATTCTAAC........................................ 1842
Homo sapiens                  ...............................TATGCAGAC........................................ 1845
```

```
                                          1910      1920      1930      1940      1950      1960      1970      1980
conservation
Callorhinchus milii              TTGACCAGAAGTCTGTCTCAGAAGCTGCAGAGGCATTCAAAGTACGGATCAGCTTGAAGACTGCCCTTGGTGAGAAAGCA  1980
Alligator mississippiensis       .............................AATGCAATCAAAGTACGGATCAGTCTGAAATCAGCACTCGGAGACAAGGCA  1887
Anolis carolinensis              .............................GATGAAATCAAAGTGAGGATCAGTCTGAAGGCAGCAATGGGAAACAGTGCA  1923
Astyanax mexicanus               ...................................TTTAAAGTGAGACTTAGTCTGAAGACTGCCATGGGAGACAATGCA  1902
Boleophthalmus pectinirostris    ...................................GTCAAAGTGAGGATCAGTTTGAAAGCTGCAATGGGCGACAATGCC  1914
Cynoglossus semilaevis           ...................................ATCAAAGTGAGAATAAGTTTGAAGGCTGCTATGGGCGACGGTGCT  1905
Danio rerio                      ...................................TTCAAAGTCAGAATAAGCCTTAAATCTGCCCTGGGAAATGAAGCA  1935
Esox lucius                      ...................................TACAAAGTGAGGTTGAGCCTGAAAAAAGCTATGGGTGACAAAGCA  1911
Fundulus heteroclitus            ...................................ATTAAAGTGAGAATAAGTTTGATATCTGCCATGGGGGACGATGCT  1917
Gekko japonicus                  .............................GATACAATCAAAGTGAGGATCAGCCTGAAGGCAGCACTGGGAGATAACGCG  1896
Haplochromis burtoni             ...................................ATTAAAGTGAGACTAAGTTTAAATTCTGCAATGGGCGATGATGCT  1905
Hippocampus comes                ...................................ATCAAAGTGAGGCTCAGTTTAAAAATGGCCATGGGTGATGAAGCT  1905
Ictalurus punctatus              ...................................ATTAAAGTGAGAGTGAGTCTAAAAGCTGCCCTGGGAGATAATGCA  1902
Larimichthys crocea              ...................................ATTAAAGTGAGAATAAGTTTGAAGGCTGCCATGGGTGAAAACGCT  1908
Lates calcarifer                 ...................................ATTAAAGTGAGACTAAGTCTGAAGGCTGCCATGGGTGATAATGCT  1911
Latimeria chalumnae              ................................TCTATAAAAGTGCGGATCAGCCTGAAAAGTGCCCTTGGAGACAAGGCT  2043
Lepisosteus oculatus             ...................................ATAAAAGTTAGAATCAGCCTGAAGACAGCATTGGGTGATGAAGCA  1902
Macaca fascicularis              .............................CAAAGCATCAAAGTGAGGATAAGCTTAAAATCAGCTCTTGGAGATAAAGCA  1896
Manacus vitellinus               .............................GATGCCATCAAAGTGCGCATCAGCCTGAAGTCAGCGCTGGGGGACCAGGCG  1902
Mus musculus                     .............................CAAAGCATTAAAGTGAGGATAAGCCTAAAATCAGCTCTTGGAGCTAATGCA  1896
Neolamprologus brichardi         ...................................ATTAAAGTGAGACTAAGTTTAAATTCTGCAATGGGCGATGATGCT  1905
Oncorhynchus mykiss              ...................................TACAAAGTGAGGTTGAGTCTGAAAGCAGCTATGGGTGACAAGGCA  1914
Oreochromis niloticus            ...................................GTTAAAGTGAGACTAAGTTTAAATTCTGCAATGGGCGATGATGCT  1905
Poecilia formosa                 ...................................ACTAAAGTGAGAATAAGTCTGAAAGCTGCTATGGGAGACGATGCT  1914
Poecilia latipinna               ...................................ACTAAAGTGAGAATAAGTCTGAAAGCTGCTATGGGAGACGATGCT  1914
Pseudopodoces humilis            .............................AATGCCATCAAAGTGCGCATCAGCCTGAAGTCAGCTCTGGGGGACCAGGCG  1896
Pundamilia nyererei              ...................................ATTAAAGTGAGACTAAGTTTAAATTCTGCAATGGGTGATGATGCT  1905
Pygocentrus nattereri            ...................................AGCAAAGTGAGACTGAGTCTAAAAGCTGCCATGGGGGAAAAGGCA  1899
Python bivittatus                .............................GATGCCATCCAAGTGAGGATCAGCTTGAAAACAGCACTGGGTGATGATGCA  1887
Rattus norvegicus                .............................CAAAGCATTAAAGTGAGGATAAGCCTAAAATCAGCTCTTGGAAAAATGCG  1896
Scleropages formosus             ...................................TTCAAAGTCAGACTGAGTCTAAAGGCTGCAATGGGCGATGAAGCT  1911
Sinocyclocheilus anshuiensis     ...................................TTCAAAGTCAGAATACGCCTAAACTCTGCCATGGGAGATGATGCA  1917
Xenopus tropicalis               .............................GAGTCCATTAAAGTCAGGATCAGTCTAATATCTGGCCTAGGAGACAATGCG  1911
Lipotes vexillifer               .............................CAAAGCATCAAAGTGAGGATAAGCCTAAAATCAGCTCTTGGAGAAAAAGCT  1893
Orcinus orca                     .............................CAAAGCATCAAAGTGAGGATAAGCCTCAAATCAGCTCTTGGAGAAAAAGCT  1893
Homo sapiens                     .............................CAAAGCATCAAAGTGAGGATAAGCCTAAAATCAGCTCTTGGAGATAAAGCA  1896

                                          1990      2000      2010      2020      2030      2040      2050      2060
conservation
Callorhinchus milii              TATGAATGGAATGCAAACGAAGAATATTTTTTCCAAGCAACTGTAGCTTATTCCATGAGGAAATACTGGGCAGAGGTTAA  2060
Alligator mississippiensis       TACATATGGGATAAAAGTGAGCTTTTTTTGTTCAAGTCATCAATTGCCTATGCCATGAGAAAGTATTTCTTAGAGGTCAA  1967
Anolis carolinensis              TACACATGGAATGATAATGAAATGTATTTGTTCAAGTCCACAATTGCCTACGCCATGAGACAATACTTTTTAATCATAAA  2003
Astyanax mexicanus               TACGCTTGGAATAGCAATGAAATGTATCTGTTTAGAGCCTCCATGGCCTACGCTATGCGACAGTACTATTTAGAGGAAAA  1982
Boleophthalmus pectinirostris    TATCCTTGGAATGACAATGAGTTGTTCCTGTTTAAATCCAACATCGCCTACGCCCTGAGACAGTACTACCAGCTCAAGAA  1994
Cynoglossus semilaevis           TACCCGTGGAACAGTAATGAGATGTACCTGTTTAAGACCAACATGGCATATGCTCTGAGGCAGTACTACAGCCAG...AA  1982
Danio rerio                      TACACCTGGAACGCAAATGACATTTATCTCTTTAAGTCCACTATGGCCTTTGCCATGCGCCAGTACTACTTGAAAGAAAA  2015
Esox lucius                      TACAGCTGGAATGCCAATGAGATTTACCTGTTCAAAGCCAACATAGCCTACGCCATGAGACAGTATTACCTCAAGGTCAA  1991
Fundulus heteroclitus            TATCCCTGGAATGTCAACGAGATGTTCCTCTTCAAGGCCAGCATTGCTTACGCCCTCAGGCAGTACTACAGCCAA...AA  1994
Gekko japonicus                  TACAAATGGAATGAAAATGAAATGTATTTATTCACGGCAACAATTGCCTATGCCATGAGAAAGTACTTTTCGGAGAAGAA  1976
Haplochromis burtoni             TACCCCTGGAATGACAATGAGCTTTACCTGTTCCGGGCCAACATTGCTTATGCCTTGAGGCAGTACTACAGCCAG...AA  1982
Hippocampus comes                TATTCCTGGAACTCCAACGAGCAGTACCTGTTCAGGGCCAGCGTCGCTTATGCCCTGCGACAGTACTACAGCCAA...AA  1982
Ictalurus punctatus              TACCAGTGGAATGGCAATGAGATGTACCTCTTCAAGGCCTCCATGGCCTTTGCCATGCGACAATACTATTTAGAGACCAA  1982
Larimichthys crocea              TATCCCTGGAACAACAATGAGCTGTACCTGTTCAAGACCAACATTGCGTATGCTTTGAGGCAGTACTACAGCCAAATGAA  1988
Lates calcarifer                 TATTCCTGGAATGCCAATGAGCTGTACCTGTTCAAGGCCAATATTGCGTACGCTCTGAGGCAGTACTACAGCCAG...AG  1988
Latimeria chalumnae              TACACCTGGGATGAAAGTGAAATGTACTATTTCAGAGCAACAGTGGCCTTTGCCATGCAGAAGTACTTTAAAGAGGTTAA  2123
Lepisosteus oculatus             TACTCGTGGAATGAAAATGAAATGTATCTCTTCAAAGCAACAATGGCTTTTGCCATGCGTCAGTATTACCTGCAA...AA  1979
Macaca fascicularis              TATGAATGGAACGACAATGAAATGTACCTGTTCCGATCATCTGTTGCATATGCCATGAGGACGTACTTTTTAGAAATCAA  1976
Manacus vitellinus               TATGAATGGGATGACAACGAGCTCTTCCTGTTCAAGTCATCCATCGCCTATGCCATGAGAAAATACTATGCAGATGTGAT  1982
Mus musculus                     TATGAATGGACCAACAACGAAATGTTCCTGTTCCGATCATCTGTTGCATATGCCATGAGAAAGTATTTTTCAATAATCAA  1976
Neolamprologus brichardi         TACCCCTGGAATGACAATGAGCTTTACCTGTTCCGGGCCAACATTGCTTATGCCTTGAGGCAGTACTACAGCCAG...AA  1982
Oncorhynchus mykiss              TACATGTGGAACGCCAACGAGATGTACCTGTTCAAAGCCAACATGGCCTACGCCATGAGACAGTATTACCTTGAGGTCAA  1994
Oreochromis niloticus            TACCCCTGGAATGCCAATGAGCTTTACCTGTTCCGGGCCAACATTGCTTATGCCTTGAGGCAGTACTACAGCCAG...AA  1982
Poecilia formosa                 TATTCCTGGAACGCCAATGAGATGTACCTCTTCAAGGCCAGCATTGCCTACGCCCTGAGGCAGTACTACAGCCAA...AA  1991
Poecilia latipinna               TATTCCTGGAACGCCAATGAGATGTACCTCTTCAAGGCCAGCATTGCCTACGCCCTGAGGCAGTACTACAGCCAA...AA  1991
Pseudopodoces humilis            TATGAATGGGATGAGAACGAGCTCTTCTTGTTCAAGTCATCTGTCGCCTATGCCATGAGAAAATACTTTGCAAAGGTGAA  1976
Pundamilia nyererei              TACCCCTGGAATGCCAATGAGCTTTACCTGTTCCGGGCCAACATTGCTTATGCCTTGAGGCAGTACTACAGCCAG...AA  1982
Pygocentrus nattereri            TACACTTGGAACGAAAATGAGATGTACCTCTTCAAAGCCTCCATGGCCTATGCCATGCGACAGTACTATTTAGAAACGAA  1979
Python bivittatus                TACAAATGGGACGAGAGTGAAATGTATTTATTCAAATCAACAATCGCCTATGCTATGCAGAAGTACTTTTTAGACATCAA  1967
Rattus norvegicus                TATGAATGGACCGACAACGAAATGTACCTATTCCGATCATCTGTTGCCTATGCCATGAGAGAGTATTTTTCAAGGGAAAA  1976
Scleropages formosus             TACCCCTGGAATGACAATGAGATGTACCTTTTCAAATCCACCATTGCCTTTGCCATGCGCCAGTATTATAATCTGGAAAA  1991
Sinocyclocheilus anshuiensis     TACACTTGGAATGCAAATGAAATGTATCTCTTTAAGTCCACTATGGGCTTTGCCATGCGCCAGTACTACTTGGAAGAAAA  1997
Xenopus tropicalis               TATAAATGGGATGCAAGTGAAATGTACCTGTTCAGGTCATCTATTGCATACGCCATGTCAAAATATTACTCTGATGTAAT  1991
Lipotes vexillifer               TATGAATGGAATGACAATGAAATGTACTTGTTCCGGTCGTCTGTTGCATACGCCATGAGAGAGTATTTTTCAAAAGTCAG  1973
Orcinus orca                     TATGAATGGAATGACAATGAAATGTACTTGTTCCGGTCATCTGTTGCATACGCCATGAGAGAGTATTTTTCAAAAGTCAG  1973
Homo sapiens                     TATGAATGGAACGACAATGAAATGTACCTGTTCCGATCATCTGTTGCATATGCTATGAGGCAGTACTTTTTAAAAGTAAA  1976
```

```
                                      2070      2080      2090      2100      2110      2120      2130      2140
conservation
Callorhinchus milii              AAGTGAAACTTTAAATTTTGAGATAACTCATGTGCACATGTCCAATTCTACTCAAAGGATTTCATTTTATTTTATTGTTA  2140
Alligator mississippiensis       GAATGAGAACATTGACTTCCAGAGCGAAGATATCCATGTAGATTCTGTGACACAGAGAATCTCCTTCTCTTTCATTGTCA  2047
Anolis carolinensis              GAAAGAAACAATAGAATTTCGATCCACCGACATCCGTGTAAAAAATGTGACGCCAAGAATCTCTTTCTCCTTTACTGTCA  2083
Astyanax mexicanus               GAATCAGGAAGTCCTTTTCATGTCAGAGAACATCCACACATATAAACTGACCCCGAGGGTTTCCTTTTACTTTGTGGTGA  2062
Boleophthalmus pectinirostris    GAACACCACTGTTATGCTCACATCAGACAATGTCATAGTTTATGACCAGACTCCAAGAATCTCCTTCTACGTCGTGGTGA  2074
Cynoglossus semilaevis           GAACCAGACTCTTCCCTTCACATCAGCGAACATTCACACCTACAACGAAACTCCCAGGATCTCCTTTTACATGGCCGCCA  2062
Danio rerio                      GAACACAGATGTAAATTTCACGCCAGAGAATATCCACACGTACAATGAAACCGCCAGAATATCCTTTAAATTTGCTGTGA  2095
Esox lucius                      CAAGACAAACGTTTCTTTCACGATAGACAATATTCACATATACAAGGAGACACCTAGAATATCATTCTACTTTGTGGTGA  2071
Fundulus heteroclitus            GAACCAGAACCTTAATTTCACAACAGAGCACATTATCACTTATGAGGAAACGCCCAGGATCTCCTTCTACATGATGGCCA  2074
Gekko japonicus                  AAATGAAACAGTAGAATTTCGAAGTGTAAATATTCATGTCGGTGATGTGACACCAAGAATCTCCTTCCAGTTTATTGTCA  2056
Haplochromis burtoni             GAACAAGACTCTTCCTTTCACAACAACTAATGTCATCACCTATAAGGAGACTCCCAGAATCTCTTTCTACGTTGTCGCCA  2062
Hippocampus comes                GAACAACACTCTTCTCTTCACAGCAGACAACGTCATAACTTACAAACAGACGCCCAGGATTTCCTTCTACATTGTGGTTA  2062
Ictalurus punctatus              GAGTGAAGTTGTTGATTTCATGGCAGAGAATATCTACACATACAAAGAAACTCCAAGGATCTCTTTTTACCTGGTGGTGA  2062
Larimichthys crocea              GAGAGAGACCCTTCTCTTCACATCAGAGGACATCCACGCATATAATGACACTCCAAGAATCTCCTTCTACATTGCGGTGA  2068
Lates calcarifer                 GAACCAGGCCCTTCTCTTCACATCAGAAAACGTCCTCACCTACAAGGAGACTCCCAGAATCTCCTTCTACATGGTGGTCA  2068
Latimeria chalumnae              AAACCAGAATGATAAATTTGTTGCTGAAAATGTTTGTACATATAACGAGAAACCAAGAATTTCCTTCTATTTTTACATCG  2203
Lepisosteus oculatus             GGGGCAGGCACTCAACTTTGTGTTGGAAAATGTCTACACATACAACGAAAAGCCAAGAGTCTCCTTCTACTTTATTGTCA  2059
Macaca fascicularis              ACATCAGACGATTCTTTTTGGGGAGGAGGATGTGCGAGTTGCTGATTTGAAACCAAGAATCTCCTTAATTTCTATGTCA   2056
Manacus vitellinus               GAAAGAGAACGTTAACTTCCAGATTACAGACGTTCATGTTGGGGAACAAACACAAAGGATCTCTTTCTATCTGACTGTCA  2062
Mus musculus                     AAACCAGACAGTTCCTTTTCTAGAGGAAGATGTACGAGTGAGTGATTTGAAACCAAGAGTCTCCTTCTACTTCTTTGTCA  2056
Neolamprologus brichardi         GAACAAGACTCTTCCTTTCACAATAACTAATGTCATCACCTATAAGGAGACTCCCAGAATCTCTTTCTACGTTGTCGCCA  2062
Oncorhynchus mykiss              CAAGACTGCAGCTCTTTTCACGACAGAGAACATCCACACCTACAAGGAGACAGCCAGAATCTCTTTCTACTTTGTGGTGA  2074
Oreochromis niloticus            GAACAAGACTCTTCCTTTCACAACAACTGATGTCATCACCTATATGGAGACTCCCAGAGTCTCCTTCTACGTTGTCGCCA  2062
Poecilia formosa                 GGACCAGAACCTTCCTTTCACAGCAGAGAACATTCTCACTTATGAAGAAACGCCCAGGATCTCCTTCTACATGGTGGCCA  2071
Poecilia latipinna               GGACCAGAACCTTCCTTTCACAGCAGAGAACATTCTCACTTATGAAGAAACGCCCAGGATCTCCTTCTACATGGTGGCCA  2071
Pseudopodoces humilis            GAAACAGGAAGTTGACTTCCAAATTGCAGACGTTCATGTCAGGGAGCAGACACAAAGGATCTCCTTCTACATTACTGTCA  2056
Pundamilia nyererei              GAACAAGACTCTTCCTTTCACAACAACTAATGTCATCACCTATAAGGAGACTCCCAGAATCTCTTTCTACGTTGTCGCCA  2062
Pygocentrus nattereri            GGGAGAGGAAGTCAATTTCTTGTCAGAGAATATCTATACATATAAAGAAACTCCAAGGATCTCCTTTTACTTTGTGGTGA  2059
Python bivittatus                GAATCAAACCGTACTATTTCAAACAGACGATGTTCATGTGAGTGATGTGACACAGAGGATCTCCTTTTATTTTACAGTCA  2047
Rattus norvegicus                GAACCAGACAGTTCCTTTTGGGGAGGCAGACGTATGGGTGAGTGATTTGAAACCAAGAGTCTCCTTCAACTTCTTTGTCA  2056
Scleropages formosus             AGGAAAACAAATTAACTTCATGCCAGAGAATATCTACACATACAAAATAACCCCAAGGATATCATTCTTCTTTGTTGTTA  2071
Sinocyclocheilus anshuiensis     GGGAATAGAAATGAATTTCATGCCAGAGAATATCAACACATACAATGAAACGGCCAGAATTTCCTTTTATTTCGTTGTGA  2077
Xenopus tropicalis               GAAACAAGAAGTATCTTTTACATTTGAAAATGTACATGTATATGACGAGACCCCAAGAGTTTCCTTTATGTTTTATGTCA  2071
Lipotes vexillifer               AAACGAGACCATTCCTTTTGGGGAGGAGGACGTGTGGGTGAGTGACTTGAAACCAAGAATCTCCTTCAACTTCTTTGTCA  2053
Orcinus orca                     AAACAAGACCATTCCTTTTGGGGAGAAGGACGTGTGGGTGAGTGACTTGAAGCCAAGAATCTCCTTCAACTTCTTTGTCA  2053
Homo sapiens                     AAATCAGATGATTCTTTTTGGGGAGGAGGATGTGCGAGTGGCTAATTTGAAACCAAGAATCTCCTTTAATTTCTTTGTCA  2056

                                      2150                2160      2170      2180      2190      2200      2210
conservation
Callorhinchus milii              AGAACCCTAAG......GACAACACGACTATTCCAAAAGCAGACGTGGAGCAAGCCATCAGGATGAATAAACATCGGTTC  2214
Alligator mississippiensis       GCATGCCTGGAAAT...ACCAGTAATGTTGTTCCCAAGTCTGATGTGGAACAAGCTATCAGAATGTCTCGTGGAAGAATC  2124
Anolis carolinensis              GCATGCCTAATGAC...AGCGCCAGTTTGGTTCCTAGAGCTGAAGTGGAAAAAGCAATCAGTATGTCTCGGGGTCGCATC  2160
Astyanax mexicanus               CTGATCCTGCCAAC...CCGAGCACTATCATTCCTAAAGCAGAAGTGGAGGCAGCAATACGGCTGTCCAGAGAACGCATT  2139
Boleophthalmus pectinirostris    AAAACCCAGCCGGA...ATGAACCAGTTTTTCCCCAAAGAAGACATAGAGGAAGCTATCAGACTGTCTCGTGGGAGAATC  2151
Cynoglossus semilaevis           GCCGCCCATCAAGT......TCAGAGTACATCCCGAAGAGTGATTTGGAGGCTGCCATCAGGTTATCCCGAGGTAGAATC  2136
Danio rerio                      TGGATCCAACCAAA...ACAGGCACAGTTATTCCAAAGGCAGAAGTAGAGAATGCTATCTGGCAGGAGAGAGATCGCATC  2172
Esox lucius                      CTGACCCTGCCAAC...CCAACCATGGTCATCCCCAAAGCCAAAGTAGAGGCAGCTATCAGGATGTCCAGAGGTCGCATC  2148
Fundulus heteroclitus            CCCATCCAGAAAAC...CCCTCTGCATACATTCCCAAGTCTGATATGGAAGCGGCCATCAGGTTATCTCGAGGTCGAATC  2151
Gekko japonicus                  CCATGCCTAATAAT...GACTCAGACATTGTTCCCAGATCTGAAGTGGAGGAAGCAATCAGAATATCACGCGGCCGCATC  2133
Haplochromis burtoni             AAAACCCAAATACA...CCCACTGAATTCATCCCAAAGAATGAGCTGAAGGAAGCCATCAGGTTAGCTCGAGGCCGGATC  2139
Hippocampus comes                CCAACCCAAATAAT...CCATCCGTATACGTGCCAAGAGAAGATTTGAAGGCAGCCATACGACTATCCCGAGGCCGAATC  2139
Ictalurus punctatus              CTGATCCTACTAAC...GCAACTAGGGTTATTCCCAAAGCAGACGTGGAGGCCGCTATCTGGCTCTCCAGAGGTCGCATC  2139
Larimichthys crocea              CCAAACCTGGGAAT...CCGTCCATATATATCCCAAAGGGTGATGTAGAAGCTGCCATAAGGTTATGTCGAGGCCGGATC  2145
Lates calcarifer                 CCAACCCTGCAAGT...CCCTCCAACTATATCCCAAAGAGTGATGTGGAGGCTGCCATCCGGTTATCCCGCGGCCGAATC  2145
Latimeria chalumnae              TTGATCCCTTGAATATCTCTAACAGCAGCATTCCAAAGGCAGAAGTGGCAGAAGCTATTAGGAAGTACAGAGAACGATTC  2283
Lepisosteus oculatus             CTGCTCCTGACAAT...AATTCAACAATAATTCCCAAAGGAGATGTGGAGGCGGCAGTGCGGCTGTCCAGGAACCGCATC  2136
Macaca fascicularis              CTGCACCTAAAAAT...GTGTCTGACATCATTCCTAGAACTGAAGTTGAAGAGGCCATCAGGATCTCCAGGAGCCGTATC  2133
Manacus vitellinus               GCATGCCAGGTAAC...ATCAGTGATACTGTGCCCAAAGCTGACGTGGAAAATGCCATCAGGATGTCTCGGGGACGAATC  2139
Mus musculus                     CCTCACCCCAAAAT...GTGTCTGATGTCATTCCTAGAAGTGAAGTTGAAGATGCCATCAGGATGTCTCGGGGCCGCATC  2133
Neolamprologus brichardi         AAAACCCAAATACA...CCCACTGAATTCATCCCAAAGAATGAGCTGAAGGAAGCCATCAGGTTAGCTCGAGGCCGGATC  2139
Oncorhynchus mykiss              CTGACCCTGCCAAC...TCTGCCGTGGTCATTCCCAAAGCTGAGGTAGAGGCTGCTATCAGGATGTCCAGAGGTCGAATC  2151
Oreochromis niloticus            AAAACCCAAATGCA...ACCACTGAATTCATCCCAAAGAATGAGCTGAAGGAAGCTATCAGGTTAGCTCGAGGCCGGATC  2139
Poecilia formosa                 CCCACCCAGGAAAC...CCCTCTACATACATTCACAAGTCTGACATGGAGGCAGCTGTCAGGTTATATCGGGGTCGAATC  2148
Poecilia latipinna               CCCACCCAGGAAAC...CCCTCTACATACATTCACAAGTCTGACATGGAGGCAGCTGTCAGGTTATATCGGGGTCGAATC  2148
Pseudopodoces humilis            GCATGCCAGGTGAT...ATCGGTGATCTTGTGCCCAAAGCTGATGTGGAAAATGCCATCAGTATGTCTCGAGGACGAATC  2133
Pundamilia nyererei              AAAACCCAAATACA...CCCACTGAATTCATCCCAAAGAATGAGCTGAAGGAAGCCATCAGGTTAGCTCGAGGCCGGATC  2139
Pygocentrus nattereri            CTGATCCTACCAAT...GCAAGCAGGATTATTCCTAAAGCTGAAGTGGAGGCAGCTTTCTGGATGTCCAGAGGACGCCTT  2136
Python bivittatus                GCATGCCAAACAAT...GTCTCAGATTTGGTTCCTAAATCTGAAGTGGAAGATGCAATCAGACTGTCTCGGGGACGTATC  2124
Rattus norvegicus                CTTCACCCAAAAAT...GTGTCTGACATCATTCCCAGAAGTGAAGTTGAAGAGGCCATCAGGATGTCTCGGGGCCGTATC  2133
Scleropages formosus             CTGACCCCACCAAC...CCAATGAAGATCATCCCAAAGGCAGATGTGGAGGTTGCTATTCGGCTTTCCAGGGATCGAATC  2148
Sinocyclocheilus anshuiensis     TGGATCCAACCAAA...CCAGACACAGTCATTCCCAAAGCAGATGTAGAGGCAGCTATCTGGTTGTCCAGAGAACGCATC  2154
Xenopus tropicalis               CCATGCCAAATAAT.........GCTGTGGTTTCGAAACTGGAAGTGGAAAAGGCTGTGAAGAAGTCTAAAGACCGGATC  2142
Lipotes vexillifer               CTTCACCTAAAAAT...GTGTCTGACATCATTCCTAGAACTGAAGTTGAAGAGGCCATCAGGATGTCCCGGGGCCGTATC  2130
Orcinus orca                     CTTCACCTAAAAAT...ATGTCTGACATCATTCCTAGAACTGAAGTTGAAGAGGCCATCAGGATGTCCCGGGGCCGTATC  2130
Homo sapiens                     CTGCACCTAAAAAT...GTGTCTGATATCATTCCTAGAACTGAAGTTGAAAAGGCCATCAGGATGTCCCGGAGCCGTATC  2133
```

```
                              2220      2230      2240      2250      2260      2270      2280      2290
conservation
Callorhinchus milii           AACAGTGCATTTTTACTGGATGATAAAACGCTGGAATTTGTGGGCATTCCCCCCACTCTTGCGCCTCAGAGTAAATCCTC  2294
Alligator mississippiensis    AGTGAGGCTTTCAAACTGGATGACAAGACGCTTGAGTTTGAGGGCATTCTTCCAACACTGACCCCACCATTTGAACCACC  2204
Anolis carolinensis           AATGAAGCATTCAAACTGGATGATGAAACTTTGGAATTTGTCGGAATCTATCCCACTTTGGCACCACCTTATGAACCCCC  2240
Astyanax mexicanus            AATAGTGTTTTCCACCTGAACGATGACACGCTGGAGTTCGAGGGACTGGTGGCCACCTTGGCTCCACCTCCAGAGCAACC  2219
Boleophthalmus pectinirostris AACGATGCCTTCCAGCTCAGTGACAGCACTCTGGAGTTTGTGGGCGTGCCTCCGACTTTATCTCCGCCTGCTGAGCAGCC  2231
Cynoglossus semilaevis        AATGATGCCTTTCAACTGGATGACTGGACGCTTGAGTTCTTAGGCATCCCAGCAACACTGGCGCCCCCTGTAGAGCAGCC  2216
Danio rerio                   AATGGTGCATTTCTACTAAGTGACGAAACTCTGGAATTTGTTGGTCTGATGGCAACATTAGCACCACCAAAAGAGGAAAA  2252
Esox lucius                   AATGATGCTTTCCAACTGGACGATAATACTCTGGAGTTTGAGGGTCTCCTAGCAACACTGGCCCCGCCCGTGGAGCAGCC  2228
Fundulus heteroclitus         AACGAAGCCTTCCAATTAGATGACCAGACGCTCGAGTTTGTAGGTATCCAGCCTACGCTGGCCCCGCCCATAAAGCAGCC  2231
Gekko japonicus               AATGAAATTTTCCAACTGGATGACCAAACTCTAGAGTTTATTGGCATTGCTCCAACACTGGAACCACCTTACACACCTCC  2213
Haplochromis burtoni          AACGACGCCTTCAACTTAGGTGATAATACTCTGGAGTTTGCCGGTATCCTACCAACACTGGCCTCACCCACTGAGCAACC  2219
Hippocampus comes             AACGACGCTTTCCAGCTGGATGACCACACACTAGAGTTTGACGGTATCCCGGCAACGCTTTCACCTCCTGTGGAGCAACC  2219
Ictalurus punctatus           AATGGGGTTTTCAGCTGACAGACGACACACTGGAGTTTGAAGGCCTGCGTGCCACATTGGCTCCTCCCACAGAGCCAAA  2219
Larimichthys crocea           AACAATGCCTTCCAATTGGATGACAGGACGCTGGAGTTTGAGGGTATTATACCAACACTGGCCACTCCCACGCAGCAGCC  2225
Lates calcarifer              AACGATGCCTTCCAGTTGGATGACAGGACACTGGAGTTTGTGGGTATCCCAGCAACACTGGCCCCTCCTGTGGAGCAGCC  2225
Latimeria chalumnae           AACAGTGCTTTTATGCTGGATGATAAGACTCTTCAATTTGTGGGTATTCCTCCAACACTGGCACCCCAGTATAAGCCACC  2363
Lepisosteus oculatus          AATAGTGTGTTTCTACTAGATGACAAAACTCTAGAGTTTGAGGGTCTCCCAGCAACATTAACACCACCTCAAGAGCAGCC  2216
Macaca fascicularis           AATGATGCTTTCCGTCTGAATGACAACAGCCTGGAGTTTCTGGGGATACAGACAACACTTGCACCTCCTTACCAGTCCCC  2213
Manacus vitellinus            AATGAGGCTTTCAGGTTGGATGATAACACGCTGGAGTTTGTGGGCATACTTCCAACCCTGGCCGCACCTTATGAACCACC  2219
Mus musculus                  AATGATGTCTTTGGCCTGAATGATAACAGCCTGGAGTTTCTGGGGATTCACCCAACACTTGAGCCACCTTACCAGCCTCC  2213
Neolamprologus brichardi      AACGACGCCTTCAAGTTAGGTGATAATACTCTGGAGTTTGCCGGTATCCTACCAACACTGGCCTCACCCACTGAGCAACC  2219
Oncorhynchus mykiss           AATGACGCATTCAAACTGGACGATAAAACTCTGGAGTTCGAGGGTCTCCTAGCAACGCTGGCCCCGCCCGTGGAGCAGCC  2231
Oreochromis niloticus         AATGACGCCTTCAAGTTAGATGATAATACTCTGGAGTTTGCCGGTATCCTACCAACACTGGCCTCACCCACTGAGCAACC  2219
Poecilia formosa              AACGAAGCCTTCCAATTGGACGACCACACACTCGAGTTCGTAGGTATCGTACCGACGCTGGCCCCGCCTGTAAAGCAGCC  2228
Poecilia latipinna            AACGAAGCCTTCCAATTGGACGACCACACACTCGAGTTCGTAGGTATCGTACCGACGCTGGCCCCGCCTGTAAAGCAGCC  2228
Pseudopodoces humilis         AATGAGGCTTTCAGACTGGATGATAATACACTGGAGTTTGTGGGAATACTTCCAACCCTGGCTGCACCCTATGAACCACC  2213
Pundamilia nyererei           AACGACGCCTTCAACTTAGGTGATAATACTCTGGAGTTTGCCGGTATCCTACCAACACTGGCCTCACCCACTGAGCAACC  2219
Pygocentrus nattereri         AATGGAATTTTCCAGCTGAGTGACGACACATTCGAGTTTGAAGGTCTGCAGGTCACCTTGGCTCCCCCTGCTGAGCCGCC  2216
Python bivittatus             AATGAAGCTTTCAAACTGAATGATCAAACTTTGGAATTTATTGACATCTTACCAACATTGGCACCACCTTATGAATCACC  2204
Rattus norvegicus             AATGATATTTTTGGTCTGAATGATAACAGCCTGGAGTTTCTGGGGATCTACCCAACACTTAAGCCACCTTACGAGCCTCC  2213
Scleropages formosus          AATGGTGTTTTCAAGCTTGATGATACAACACTGGAGTTTGAGGGCATCCTCTCTACACTGTCTCCACCTGTGGAACCACC  2228
Sinocyclocheilus anshuiensis  AATGGTGCATTTTTACTCAATGATGAAACGCTGGAATTTGTTGGGCTGCAGGCAACATTAGCTCCACCCAAAGAGGAAAA  2234
Xenopus tropicalis            AACAGCATATTTAAACTAGATGACAATGCTCTAGAGTTTATAGGTATTCCTCCTACTCTGGCAGCCCCAGTAGAACAGCC  2222
Lipotes vexillifer            AATGATGCTTTCCGCCTGGATGACAGCAGCCTGGAGTTTCTGGGGGTTCAGCCGACGCTGGCACCCCCTTATAAGCCACC  2210
Orcinus orca                  AATGATGCTTTCCGCCTGGATGACAGCAGCCTGGAGTTTCTGGGGGTTCAGCCGACGCTGGCACCCCCTTACGAGCCACC  2210
Homo sapiens                  AATGATGCTTTCCGTCTGAATGACAACAGCCTAGAGTTTCTGGGGATACAGCCAACACTTGGACCTCCTAACCAGCCCCC  2213

                              2300      2310      2320      2330      2340      2350      2360      2370
conservation
Callorhinchus milii           GGTGACAGTCTGGCTCATCCTGTTTGGAGTGGTCATGGGCATGGTTTGCATCGCGCTAGCTTTGCTGATTATTACCGGAC  2374
Alligator mississippiensis    TGTCACCATATGGCTAATAGTGTTTGGAGTAGTCATAGGCGTTGTAGTAGTTGGAATCATTGTCTTGATCTTCACTGGGC  2284
Anolis carolinensis           AGTCACTGTGTGGGTTATAGTATTTGGAATTGTCGTAGGTATTGTAGTGATCGCATCCATTGCCTTGATCATCATGGGAG  2320
Astyanax mexicanus            CGTGACCATTTGGCTGGTGGTGTTTGGAGTGGTGATGGCCGTGGTTGTTTGTGCGGGGGTTTACCTAGTTGTCATGGGTG  2299
Boleophthalmus pectinirostris TGTGGAGGTGTGGCTTGTCATATTTGGGGTGGTCATGGGACTTGTAGTCCTCACCGGCATCTATATGGTGTTCTCCGGTA  2311
Cynoglossus semilaevis        AGTGGAGGTGTGGCTGGTTGTATTCGGAGTTGTCATGGGAGTGGTGGTCCTAATGGGCATGTTTCTCATCATCTCTGGTG  2296
Danio rerio                   AATCACAATTTGGCTGGTGGTGTTTGGGGTAGTAATGGGAGTGACTGTCCTTGCGGGGATCTACCTCGTTACCACAGGGA  2332
Esox lucius                   GGTAACTGTCTGGCTGGTGGTGTTTGGTGTGGTGATGGGATTGGTGGTCTGCATGGGGTCCTACCTGGTAATCTCCGGAC  2308
Fundulus heteroclitus         CGTGGAGGTGTGGCTGGTGGTATTTGGTGTTGTGATGGGCATCGTGGTGGCACTGGGTCTCTACCTCATCATAACTGGTG  2311
Gekko japonicus               AGTCACTGTCTGGCTGATATTATTTGGAATTGTCATGGGCATTGTCGTAGTTGCGATGATTGTCTTGATCATCACTGGAC  2293
Haplochromis burtoni          TGTGCAGGTGTGGCTGGTGGTATTTGGTGTGGTTATGGGCATTGTGGTGGTAGCCGGCGTCTACCTTGTCGTCTCTGGTG  2299
Hippocampus comes             GGTGGAGGTGTGGCTGGTTGTGTTTGGAGTGGTTATGGGAGTGGTGGTGCTAATGGGGCTCCTCCTCATCATCTCTGGCA  2299
Ictalurus punctatus           GTTTGAAGTGTGGCTGGTTGTTTTTGGCGTGGTGATGGCCTTGGTGGTTTGTATTGGCGTTTACCTCGTTGTCATGGGCA  2299
Larimichthys crocea           GGTGGAGGTGTGGCTGGTGGTATTTGGGGTGGTCATGGGAGTTGTGGTGTTGGCAGGCGTTTACCTCGTCATCACTGGTG  2305
Lates calcarifer              AGTGGAGGTTTGGCTGGTGGTGTTTGGGGTGGTCATGGGAGTCGTGGTGCTAATGGGCATCTACCTCATCGTGTCTGGCG  2305
Latimeria chalumnae           TGTTACAGTGTGGCTTATAGTCTTTGGAGTTGTCATCGGTGTAGTTGTGATTGGCATTATTATCCTTGTTGTCAGTGGGA  2443
Lepisosteus oculatus          TGTGACTGTGTGGCTTGTTGTGTTTGGGGTGATCATGGGTGTGGTTGTGGCTGCTGCAGCATACCTGATTATATCTGGAT  2296
Macaca fascicularis           CGTTACCACATGGCTAATTGTTTTTGGAGTTGTGATGGGAGTGATAGTGGCTGGCATTGTCGTCCTGATCTTCACTGGGA  2293
Manacus vitellinus            AGTCACCATCTGGTTAATTGTATTTGGGGTGGTCATTGGTCTGGTTGTCATTGGAGTTATTGCCTTGATCATCACTGGCC  2299
Mus musculus                  TGTCACCATATGGCTGATTATTTTTGGTGTTGTGATGGCACTGGTAGTGGTTGGCATCATCATCCTGATTGTCACTGGGA  2293
Neolamprologus brichardi      TGTGCAGGTGTGGCTGGTGGTATTTGGTGTGCTTATGAGCATTGTGGTGGTAGCCGGCGTCTACCTTGTCGTCTCTGGTG  2299
Oncorhynchus mykiss           GGTCACCGTCTGGCTGGTGGTGTTTGGGGTGGTCATGGGACTGGTGGTCTGCATGGGCTGTTACCTCATCATCTCTGGCT  2311
Oreochromis niloticus         TGTGCAGGTGTGGCTGGTGGTATTTGGTGTGGTTATGGGCATTGTGGTGGTAGCTGGCGTCTACCTTGTCGTCTCCGGTG  2299
Poecilia formosa              AGTGCAGGTGTGGCTGGTGCTGTTCGGTGTCGTCATGGGACTCACGGTGTTGCTGGGCGCCTACCTCGTCATATCTGGTG  2308
Poecilia latipinna            AGTGCAGGTGTGGCTGGTGCTGTTCGGTGTCGTCATGGGACTCACGGTGTTGCTGGGCGCCTACCTCGTCATATCTGGTG  2308
Pseudopodoces humilis         AGTAACCATCTGGTTAATTGTATTTGGGGTGGTCATGGGTCTGATTGTCGTTGGAGTTATTGCCTTGATCATCACTGGGC  2293
Pundamilia nyererei           TGTGCAGGTGTGGCTGGTGGTATTTGGTGTGGTTATGGGCATTGTGGTGGTAGCCGGCGTCTACCTTGTCGTCTCTGGTG  2299
Pygocentrus nattereri         AGTCACCGTTTGGCTGGTTGTGTTTGGAGTGGTCATGGCCTTGGCGGTGTGTGTGGGAGTTTACCTCGTGGTCATGGGGG  2296
Python bivittatus             CATCACTGTGTGGCTTGTAGTATTTGGAGTTGTGATTGGTATTGTAGTGATTGGAATCGTCACCTTGATCATTATTGGAC  2284
Rattus norvegicus             TGTCACCATATGGCTGATTATTTTTGGTGTCGTGATGGGAACGGTAGTGGTTGGCATTGTTATCCTGATCGTCACTGGGA  2293
Scleropages formosus          TGTGACAGTGTGGCTGATAGCATTTGGGGTTGTCATGGGTCTGGTGATTTTGGTTGGCATGTACCTTGTTTTCTCTGGCT  2308
Sinocyclocheilus anshuiensis  AATCACAGTTTGGCTGGTGGTATTTGGAGTCGTAATGGGTGTAACTGTGTTTGGGGGGATCTACCTCATTACCACAGGGA  2314
Xenopus tropicalis            CTTCAGTATATGGCTTGTGGTATTTGGTGCCGTAGCTGCCATAATAGTTGTTGCTTTCATTGTTCTGATCGTTCTTGGTT  2302
Lipotes vexillifer            TGTCACCGTATGGCTGATTATCTTTGGGGTCGTGATGGGAGTGGTAGTGATTGGTATTGTTGTGCTCATCTTCACTGGCA  2290
Orcinus orca                  TGTCACCGTATGGCTGATTATCTTTGGGGTCGTGATGGGAGTGGTAGTGATTGGTATTGTTGTGCTCATCTTCACTGGCA  2290
Homo sapiens                  TGTTTCCATATGGCTGATTGTTTTTGGAGTTGTGATGGGAGTGATAGTGGTTGGCATTGTCATCCTGATCTTCACTGGGA  2293
```

```
                                   2380          2390           2400          2410
conservation
Callorhinchus milii                AGAGAGCTAAAAAACAG...AAGGCGAAGGAAACAGATGTATAT....................................  2415
Alligator mississippiensis         AGAGAGACAAAAAAAAGAAAAAAAAA...GCAAAAGCAAATGAACTGGTAAAATGTGACCTGAGT...............  2346
Anolis carolinensis                TGAGAGACCGAAAAAGGAGAAGAAATGCAGCACAGAATGACTTAGTCCGAACC...........................  2373
Astyanax mexicanus                 CGATTACCAGAAAGAGGAAAGCCAAGAAACTGGAGGAACCT.......................................  2340
Boleophthalmus pectinirostris      TCAGGGAACGCAGGAGGAAAGTCGTTAAAGATGAAGTTTTG.......................................  2352
Cynoglossus semilaevis             TCAGAGAGCGCAAGAAGAGATCTGCCAGGCCATTGGTG..........................................  2334
Danio rerio                        TATTAAATAGGAAGAAGAAAGCTAAGAAGGCTAAGGAGGCCTCAGTT.................................  2379
Esox lucius                        TCAGAGATAGGAAAAGAAAATCTGCAGCTGATGCTGAAGTGAATGCA.................................  2355
Fundulus heteroclitus              TCAGGGAGCGTAAGAAGAAACCTTCAAAGTCAACTGTG..........................................  2349
Gekko japonicus                    AAAGAAACCGGAAAAAGAAAAGAAAGGCAACTGAAAATGAGTTATCCCAAACAACAATAGCGAACTGTCATGAC......  2367
Haplochromis burtoni               TCAAGGAGCGCAGGAAGAAA......AAGCCTGGTGTG..........................................  2331
Hippocampus comes                  TCAGAGAGCGTAGGAAGAAGTCGAAAAACATGGGGGTG..........................................  2337
Ictalurus punctatus                CCGTGAACAGGAAGAGGAAAGCCAAGAAAGCGGACAAGCCT.......................................  2340
Larimichthys crocea                TAAGAGACCGCAGAAAGAAACCTGAAGATCCAGTTGTG..........................................  2343
Lates calcarifer                   TCAGAGAGCGCAAGAAGAAATCTGCAAAGACAGGCATG..........................................  2343
Latimeria chalumnae                TTAAAACCAGAAAGAAGAGAAAACATGCAAGAGAGAGTGCATTG....................................  2487
Lepisosteus oculatus               TCCTGAACAGGAAGAGAAATTCGAAGATCCACAAAGAAAGAGAGGCA.................................  2343
Macaca fascicularis                TCAGAGATCGAAAGAAGAAAAATCAAGCAAGAAGTGAAGAAAATCCTTATGCC...........................  2346
Manacus vitellinus                 AAAGAGATCGCAAAAAGAGAGCAAGAGAAAGTAGAGTTGAAGCAGGATCAAACCCTGAAGCAGTG...............  2364
Mus musculus                       TCAAAGGTCGAAAGAAGAAAAATGAAACAAAAAGAGAAGAGAACCCTTATGAC...........................  2346
Neolamprologus brichardi           TCAAGGAGCGCAGGAAGAAA......AAGCCTGGTGTG..........................................  2331
Oncorhynchus mykiss                TCAGAGACAGGAAGAAGAAATCTGCAGCGAAAGCTGAAGAGAATGCA.................................  2358
Oreochromis niloticus              TCAAGGAGCGCAGGAAGAAA......AAGCCTGGTGTG..........................................  2331
Poecilia formosa                   TCAAGGAGCGCAAGAAGAAATCGTCAAAGCCGCCTGAG..........................................  2346
Poecilia latipinna                 TCAAGGAGCGCAAGAAGAAATCGTCAAAGCCGCCTGAG..........................................  2346
Pseudopodoces humilis              AAAGAGATCGCAAAAAGAGAGCAAGAGAAAGCAGAGGTGAAGCAGGATCAAATCATGAAGAGGTG...............  2358
Pundamilia nyererei                TCAAGGAGCGCAGGAAGAAA......AAGCCTGGTGTG..........................................  2331
Pygocentrus nattereri              CAGTGAATAGAAAGAGGCAAGCCAAGAAACTTGAAGAGCCT.......................................  2337
Python bivittatus                  AGAAAGATCGAAAAAAGAAAAAACGGACAGCACAAACTGAGGCCATTGAAACT...........................  2337
Rattus norvegicus                  TCAAAGGTCGAAAGAAGAAAAATGAAACAAAAAGAGAAGAGAATCCTTATGAC...........................  2346
Scleropages formosus               TCCGTGAAAGAAAAAGAAAATCTAAG...GCACAGGAATCC.......................................  2346
Sinocyclocheilus anshuiensis       TATTAAACAGGAAGAAGAAAGCTAAGAAGGCTGACGAGACT.......................................  2355
Xenopus tropicalis                 ACAAAGAAAGGAAAACGAAACAAAAATCTCAAGGAGACAAGGAAGCTGTAGAGCTCAGCCAAAACGCTAATAACCCTGAA  2382
Lipotes vexillifer                 TCAGAGATCGAAGAAAGAAAAATCAAGCAAGCAGTGAAGAAAATCCTTATGGC...........................  2343
Orcinus orca                       TCAGAGATCGAAGAAAGAAAAATCAAGCAAGCAGTGAAGAAAATCCTTATGGC...........................  2343
Homo sapiens                       TCAGAGATCGGAAGAAGAAAAATAAAGCAAGAAGTGGAGAAAATCCTTATGCC...........................  2346

conservation
Callorhinchus milii                ................................................................................  2415
Alligator mississippiensis         ................................................................................  2346
Anolis carolinensis                ................................................................................  2373
Astyanax mexicanus                 ................................................................................  2340
Boleophthalmus pectinirostris      ................................................................................  2352
Cynoglossus semilaevis             ................................................................................  2334
Danio rerio                        ................................................................................  2379
Esox lucius                        ................................................................................  2355
Fundulus heteroclitus              ................................................................................  2349
Gekko japonicus                    ................................................................................  2367
Haplochromis burtoni               ................................................................................  2331
Hippocampus comes                  ................................................................................  2337
Ictalurus punctatus                ................................................................................  2340
Larimichthys crocea                ................................................................................  2343
Lates calcarifer                   ................................................................................  2343
Latimeria chalumnae                ................................................................................  2487
Lepisosteus oculatus               ................................................................................  2343
Macaca fascicularis                ................................................................................  2346
Manacus vitellinus                 ................................................................................  2364
Mus musculus                       ................................................................................  2346
Neolamprologus brichardi           ................................................................................  2331
Oncorhynchus mykiss                ................................................................................  2358
Oreochromis niloticus              ................................................................................  2331
Poecilia formosa                   ................................................................................  2346
Poecilia latipinna                 ................................................................................  2346
Pseudopodoces humilis              ................................................................................  2358
Pundamilia nyererei                ................................................................................  2331
Pygocentrus nattereri              ................................................................................  2337
Python bivittatus                  ................................................................................  2337
Rattus norvegicus                  ................................................................................  2346
Scleropages formosus               ................................................................................  2346
Sinocyclocheilus anshuiensis       ................................................................................  2355
Xenopus tropicalis                 CCTGCTACATTGGAAATTACTCAAGAAAATACAGGGCAAATGAATAAAGCATTTGATGGAAATGATAATAAAGCATTTGA  2462
Lipotes vexillifer                 ................................................................................  2343
Orcinus orca                       ................................................................................  2343
Homo sapiens                       ................................................................................  2346
```

| Species | Sequence (alignment columns 2412–2461) | Position |
|---|---|---|
| Callorhinchus milii | .............GAAAATCCTAGTTCAATCGAAGAACCTGATTTTGACAAA.....................GGGGTAA | 2461 |
| Alligator mississippiensis | ................CATCTTTCCTCTGTTGAT.......................................GGAGAAA | 2371 |
| Anolis carolinensis | ................GACCCTTATTCAAACTAT.......................................GGAGAAA | 2398 |
| Astyanax mexicanus | .............GAAAACCCATATGAT......GAACCATCTGAA...........................GGAATAG | 2374 |
| Boleophthalmus pectinirostris | .............GAAAACCCATATGACTTAGAC.......................................GGACACT | 2380 |
| Cynoglossus semilaevis | .............AATAATCCTTATGAAACTGAA.......................................GGGCAAA | 2362 |
| Danio rerio | .............GAGAACCCATACGAT......GGATCAGATGAT...........................GGTGAAG | 2413 |
| Esox lucius | .............GAGAACCCCTATGAAAATGCGACAATT......GAA........................GGGAAGA | 2392 |
| Fundulus heteroclitus | .............GAGAATCCTTACAACATAGACACTGAC.................................GGGATTA | 2383 |
| Gekko japonicus | ....AACAGTGGAGCAAAGGATGATGACAACGGT.......................................GGACAAA | 2404 |
| Haplochromis burtoni | .............GAGAACCCCTATGAGTCATCCACTGAT.................................GGACAAA | 2365 |
| Hippocampus comes | .............GAAAATCCTTACGAGCCTGAGCCGGAG.................................GGACAGA | 2371 |
| Ictalurus punctatus | .............GAGAACCCGTACGAG......GAACATACTAAT...........................GGGACGG | 2374 |
| Larimichthys crocea | .............GAAAATCCCTACGATGAAGTCGATGAAATGTCTAAT........................GGACAAG | 2386 |
| Lates calcarifer | .............GAGAATCCCTATGATACGACCATTGAT.................................GGACAAT | 2377 |
| Latimeria chalumnae | .............GAACATCCTCAGAACCCATATGAAGAAGATACTGATAAT.....................GGAATTG | 2533 |
| Lepisosteus oculatus | ..........CCAGAAAACCCCTACGGAAGTGTCAATCAGACCAAT...........................GGAGTGA | 2386 |
| Macaca fascicularis | ................TCCATCGATATTAACAAA.......................................GGAGAAA | 2371 |
| Manacus vitellinus | ................AACCCTTATGCTGAAGAG.......................................GGAAAAA | 2389 |
| Mus musculus | ................TCGATGGACATTGGAAAA.......................................GGAGAAA | 2371 |
| Neolamprologus brichardi | .............GAGAACCCCTATGAGTCATCCACTGAT.................................GGACAAA | 2365 |
| Oncorhynchus mykiss | .............GAGAACCCCTATGGC................................................GTGA | 2377 |
| Oreochromis niloticus | .............GAGAACCCCTATGAGTCATCCACTGAT.................................GGACAAA | 2365 |
| Poecilia formosa | .............GAGAATCCTTACAGCATAGATGTCGAT.................................GGGATCA | 2380 |
| Poecilia latipinna | .............GAGAATCCTTACAGCATAGATGTCGAT.................................GGGATCA | 2380 |
| Pseudopodoces humilis | ................AACCCTTACGGAGAAGAG.......................................GGAAGAA | 2383 |
| Pundamilia nyererei | .............GAGAACCCCTATGAGTCATCCACTGAT.................................GGACAAA | 2365 |
| Pygocentrus nattereri | .............GAAAACCCATATGAT......GAACCACCTGAA...........................GGAATAC | 2371 |
| Python bivittatus | ................GCTGTTAATGAGGAATGT.......................................GGAGAAA | 2362 |
| Rattus norvegicus | ................TCCATGGACATTGGCAAA.......................................GGAGAAA | 2371 |
| Scleropages formosus | .............GAAAACCCATATGATGCCGAAATGAAAATTGAA...........................GGAGTGA | 2386 |
| Sinocyclocheilus anshuiensis | .............GTGAACCCATATGAG......AATTCAGAT..............................GGAGAAG | 2386 |
| Xenopus tropicalis | TGGAAATGATAATAAAGCGATTGACGGAAATGACAATAAAGGGATTGATGGAAAGGATAATAAAGCATTCAGTGGAAATG | 2542 |
| Lipotes vexillifer | ................TCCGTGGGCTTGAGTAAA.......................................GGAGACA | 2368 |
| Orcinus orca | ................TCCGTGGGCTTGAGTAAA.......................................GGAGAAA | 2368 |
| Homo sapiens | ................TCCATCGATATTAGCAAA.......................................GGAGAAA | 2371 |

| Species | Sequence (alignment columns 2462–2511) | Position |
|---|---|---|
| Callorhinchus milii | AGAATTCAGCTTTTACCATTGAAGAATCATTGAATAATACAGCAATGTAA | 2511 |
| Alligator mississippiensis | GCAATGCTGGGTTTGAACCAAGCGAAGATAGACAAACATCATTTTAG... | 2418 |
| Anolis carolinensis | ACAAGTTAATGTCCCAAGAAGGAATTAACAACTCAACGACATTCTAA... | 2445 |
| Astyanax mexicanus | CAAACAAAGCTTTTGAG...AGTGAGAATGAACAAACAGGATTTTAA... | 2418 |
| Boleophthalmus pectinirostris | CCAACAAAGGTTTTGAG...AGCGAGAATGAACAAACTGGATTTTAG... | 2424 |
| Cynoglossus semilaevis | CCAACAAGGCTTTCGAGGACAACGAAGGTGAACAAACTGGATTCTGA... | 2409 |
| Danio rerio | TGAACAAAGCCTTTGAG...GAAGACATTGAACAAACAGGACTGTAA... | 2457 |
| Esox lucius | CCAACAAAATCTTTGATAGTGAAGAAGATCAACAGACAGGATTCTGA... | 2439 |
| Fundulus heteroclitus | GTAACGCCGCCTATGATGACTTTAACAATGAACAAACTGGACTTTAA... | 2430 |
| Gekko japonicus | ATAATCTAAATTATCTGCAAGATGATGAGTGCTCATCAGCATTCTAA... | 2451 |
| Haplochromis burtoni | GCAACAAAGCCTATGATGACAGTGACAATGAGCAAACTGGATTCTGA... | 2412 |
| Hippocampus comes | CCAACAGAGCCTATGATGACACGAATGATGAACAAACCGGTCTCTGA... | 2418 |
| Ictalurus punctatus | TGAACAAGGCTTATGAG...AGCGACAGTGAGCAAACGGGGTTTTAA... | 2418 |
| Larimichthys crocea | TTAACAAAGCCTATGATGACAGTGGCCATGAACAAACTGGATTCTAA... | 2433 |
| Lates calcarifer | CTAACAAAGCCTTTGAGGATGGTGATGATGAACAGACTGGATTCTGA... | 2424 |
| Latimeria chalumnae | AAAATGAAGCTTTCAGCAAAGAGGGAGAATTGCAAACAGATTTCTAA... | 2580 |
| Lepisosteus oculatus | CAAATGTAGCGTATTCT...GACGAAGATAATCACACGGGATTGTAA... | 2430 |
| Macaca fascicularis | ATAATCCAGGATTCCAAAACACTGATGATGTTCAGACCTCCTTTTAG... | 2418 |
| Manacus vitellinus | GCAACCTGGGCTTTGAGCCATCTGAAGAGACACAAACATCATTTTGA... | 2436 |
| Mus musculus | GCAATGCAGGATTCCAAAACAGTGATGATGCTCAGACTTCCTTTTAG... | 2418 |
| Neolamprologus brichardi | GCAACAAAGCCTATGATGACAGTGACAATGAGCAAACTGGATTCTGA... | 2412 |
| Oncorhynchus mykiss | CCAACAAAACATTTGAGAGGGAAGAGGATGAACAGACAGGGTTCTGA... | 2424 |
| Oreochromis niloticus | GCAACAAAGCCTATGAC...AGTGACAATGAGCAAACTGGATTCTGA... | 2409 |
| Poecilia formosa | GTAACGCTGCCTTTGAGGACAATAAGGACGAACAAACTGGACTTTAA... | 2427 |
| Poecilia latipinna | GTAACGCTGCCTATGAGGACAATAAGGACGAACAAACTGGACTTTAA... | 2427 |
| Pseudopodoces humilis | GCAACCTGGGCTTTGAGCCAGCTGAAGAGACACAAACATCATTTTAA... | 2430 |
| Pundamilia nyererei | GCAACAAAGCCTATGATGACAGTGACAATGAGCAAACTGGATTCTGA... | 2412 |
| Pygocentrus nattereri | CTAACAAAGCCTTTGAG...AGCGACAGTGAGCAAACAGGATTTTAA... | 2415 |
| Python bivittatus | GCAATTCAGCATTTCAACAGGATGAAGCTGCCTTAACAACTTTATAA... | 2409 |
| Rattus norvegicus | GTAACGCAGGATTCCAAAACAGTGATGATGCTCAAACTTCATTCTAA... | 2418 |
| Scleropages formosus | CCAACAAAAGCTTTGAA...CGTGATGATGAACAAACAGGATTTTGA... | 2430 |
| Sinocyclocheilus anshuiensis | AGAACAAAGCCTTTGAG...GAAGACATTGAACAAACAGGACTATAA... | 2430 |
| Xenopus tropicalis | ATAATAAAGCATATGAAGAAGTAGAGACTATGAATACAACATTTTAA... | 2589 |
| Lipotes vexillifer | ATAATTCAGGATTCCAAAATAGTGATGATGTTCAGACTTCGTTTTAG... | 2415 |
| Orcinus orca | ATAATCCAGGATTCCAAAATAGTGATGATGTTCAGACTTCGTTTTAG... | 2415 |
| Homo sapiens | ATAATCCAGGATTCCAAAACACTGATGATGTTCAGACCTCCTTTTAG... | 2418 |

X non conserved
X $\geq$ 60% conserved
X $\geq$ 85% conserved
