# Supplementary material for: A Genomic Survey of Angiotensin-Converting Enzymes Provides Novel Insights into Their Molecular Evolution in Vertebrates
Source: Molecules. 2018 Nov 9;23(11):2923. doi: 10.3390/molecules23112923 (PMC6278350; doi:10.3390/molecules23112923)
Supplement: Supplementary file 1 [file molecules-23-02923-s001.zip › Figure S6.pdf]

```
                                          10        20        30        40        50        60        70
conservation
ace_Alligator_mississipp.Block2        NMWAQSWSAIFDLLTPFPSATKVDATPAMKSQNWTPKRMFQESDNFFTSLDLIPMPQEFWDKSMIEKPSDGR   72
ace_Alligator_mississipp.Block4        NMWAQQWNNIYDMMIPYPNKPNLDVTNAMNQKNWNATHMFRVSEEFFTSLGLLGMPPEFWAESMLEKPTDGR   72
ace_Danio_rerio.Block2                 NMWAQTWSGIMDLVNPYPDATQVDATPAMIAQGWTPKRMFEESDRFFTSLGLLPMPPEFWNKSMLEKPTDGR   72
ace_Danio_rerio.Block4                 NMWSQTWNNIYNMMIPFPNRPNVDVTNTMIAKGYNATHMFRVAEEFFTSLGLLEMPPEFWDKSMLEKPTDGR   72
ace_Homo_sapiens.Block2                NMWAQTWSNIYDLVVPFPSAPSMDTTEAMLKQGWTPRRMFKEADDFFTSLGLLPVPPEFWNKSMLEKPTDGR   72
ace_Homo_sapiens.Block4                DMWAQSWENIYDMVVPFPDKPNLDVTSTMLQQGWNATHMFRVAEEFFTSLELSPMPPEFWEGSMLEKPADGR   72
ace_Lepisosteus_oculatus.Block2        NMWAQSWSNIYDLVTPFPDAGQVDATPAMVAKGWDARKMFEQSDHFFQSLGLIPMPPEFWSKSMLEKPSDGR   72
ace_Lepisosteus_oculatus.Block4        NMWAQQWNNIYDMMVPFPHKPNLDVTQTMVNLKWNATHMFRVSEEFFTSLGLIAMPQEFWDHSMLEKPTDGR   72
ace_Mus_musculus.Block2                NMWAQTWSNIYDLVAPFPSAPNIDATEAMIKQGWTPRRIFKEADNFFTSLGLLPVPPEFWNKSMLEKPTDGR   72
ace_Mus_musculus.Block4                DMWAQSWENIYDMVVPFPDKPNLDVTSTMVQKGWNATHMFRVSEEFFTSLGLSPMPPEFWAESMLEKPTDGR   72
ace_Pseudopodoces_humili.Block2        NMWAQSWSNIFDLVIPFPDATKVDATPAMKKQGWTPKKMFEESDRFFTSLGLIPMPQEFWDKSMIEKPSDGR   72
ace_Pseudopodoces_humili.Block4        NMWAQQWNNIYDMLIPYPEKPNLDVTSSMVQQGWNATHMFRVSEEFFTSLGLLEMPTEFWKKSMLEKPTDGR   72
ace_Scleropages_formosus.Block2        NMWAQSWAAIMDLAVPFPDATQVDATPAMIAKKWDARKMFNESDRFFTSLGLIPMPPEFWDKSMLEKPTDGR   72
ace_Scleropages_formosus.Block4        NMWAQTWNNIYDMMIPFPGKPNVDVTDAMIAQDWTTTKMFRVSDEFFTSLGLEKMPDEFWNMSMLEKPNDGR   72
ace2_Alligator_mississipp.Block5       DMWGRFWTNLYPLTVPYPDKPNIDVTNTMVEKNWNASKIFKAAENFFVSIGLPPMTPGFWINSMLTEPND.R   71
ace2_Danio_rerio.Block5                DMWGRFWTNLYPLMIPYPDRPDIDVSSAMVEQGWDEIRLFKEAEKFFMSVNMPAMFDNFWNNSMFIKPEE.R   71
ace2_Homo_sapiens.Block5               DMWGRFWTNLYSLTVPFGQKPNIDVTDAMVDQAWDAQRIFKEAEKFFVSVGLPNMTQGFWENSMLTDPGNVQ   72
ace2_Lepisosteus_oculatus.Block5       DMWGRFWTNLYPLSIPYKNKEDIDVTSAMVNQGWTVLRMFREAEKFFMSVGLYKMYDNFWNESMLEKPDDGR   72
ace2_Mus_musculus.Block5               DMWGRFWTNLYPLTVPFAQKPNIDVTDAMMNQGWDAERIFQEAEKFFVSVGLPHMTQGFWANSMLTEPADGR   72
ace2_Pseudopodoces_humili.Block5       DMWGRFWTNLYALTVPYPAKPNIDVTSAMVEKKWDAIKIFKSAEAFFVSIGLDPMTEGFWENSMLTEPTDNR   72
ace2_Scleropages_formosus.Block5       DMWGRFWTNLYPLSIPYPNKEDIDVTSAMISQGWTVNQMFKEAEKFFMSVNLYKMFDSFWSNSMFTKPEDGQ   72
```

```
                                          80        90        100       110       120       130       140
conservation
ace_Alligator_mississipp.Block2        EVVCHASAWDFYNRKDFRIKQCTVVNMDDLITVHHEMGHVQYFLQYKDQPISFRDGANPGFHEAIGDVMALS  144
ace_Alligator_mississipp.Block4        EVVCHASAWDFYNRKDFRIKQCTTVTMEQLFTVHHEMGHVEYYLQYKDQPVTFRSGANPGFHEAIGDVMGLS  144
ace_Danio_rerio.Block2                 EVVCHASAWDFYNRKDFRIKQCTVVTMDDLITVHHEMGHVQYFLQYKDQPISFRDGANPGFHEAIGDVLALS  144
ace_Danio_rerio.Block4                 EVVCHASAWDFYNRKDFRIKQCTTVTMEQLFTVHHEMGHVEYYLQYKDQPVSFRRGANPGFHEAIGDVLSLS  144
ace_Homo_sapiens.Block2                EVVCHASAWDFYNGKDFRIKQCTTVNLEDLVVAHHEMGHIQYFMQYKDLPVALREGANPGFHEAIGDVLALS  144
ace_Homo_sapiens.Block4                EVVCHASAWDFYNRKDFRIKQCTRVTMDQLSTVHHEMGHIQYYLQYKDLPVSLRRGANPGFHEAIGDVLALS  144
ace_Lepisosteus_oculatus.Block2        SVVCHASAWDFYNRKDFRIKQCTVVTMDDLITVHHEMGHVQYFLQYKDQPVPFRDGANPGFHEAIGDVLALS  144
ace_Lepisosteus_oculatus.Block4        QVVCHASAWDFYNRKDFRIKQCTTVTMEQLFTVHHEMGHVEYYLQYKDQPVGFRSGANPGFHEAVGDVLSLS  144
ace_Mus_musculus.Block2                EVVCHPSAWDFYNGKDFRIKQCTSVNMEDLVIAHHEMGHIQYFMQYKDLPVTFREGANPGFHEAIGDIMALS  144
ace_Mus_musculus.Block4                EVVCHASAWDFYNRKDFRIKQCTRVTMEQLATVHHEMGHVQYYLQYKDLHVSLRRGANPGFHEAIGDVLALS  144
ace_Pseudopodoces_humili.Block2        EVVCHASAWDFYNRKDFRIKQCTVVNMDDLITVHHEMGHVQYFLQYKDQPVSFRDGANPGFHEAVGDVMALS  144
ace_Pseudopodoces_humili.Block4        EVVCHASAWDFYNRKDFRIKQCTSVTMEQLFTVHHEMGHIQYYLQYKDQPVSFRSGANPGFHEAVGDVLSLS  144
ace_Scleropages_formosus.Block2        NVVCHASAWDFYNRKDFRVKQCTVVTMDDLITVHHEMGHVQYFLQYKEQPISFREGANPGFHEAIGDVMALS  144
ace_Scleropages_formosus.Block4        EVVCHASAWDFYNRKDFRIKQCTTVTMEQLFTVHHEMGHIEYYLQYKEQPVSFRGGANPGFHEAIGDVLSLS  144
ace2_Alligator_mississipp.Block5       KVVCHPTAWDMG.MKDYRIKMCTKVTMDDFLTAHHEMGHIVYDMAYSNLTFLLRSGANEGFHEAVGEIMSLS  142
ace2_Danio_rerio.Block5                DVVCHPTAWDMGNRKDFRIKMCTKVNMDDFLTVHHEMGHNQYQMAYRNHPYLLRDGANEGFHEAVGEIMSLS  143
ace2_Homo_sapiens.Block5               KAVCHPTAWDLGKG.DFRILMCTKVTMDDFLTAHHEMGHIQYDMAYAAQPFLLRNGANEGFHEAVGEIMSLS  143
ace2_Lepisosteus_oculatus.Block5       KVVCHPTAWDMGNGKDFRIKMCSKVNMDDFLTVHHEMGHNQYQMAYSNLSYLLRDGANEGFHEAVGEIMSLS  144
ace2_Mus_musculus.Block5               KVVCHPTAWDLGHG.DFRIKMCTKVTMDNFLTAHHEMGHIQYDMAYARQPFLLRNGANEGFHEAVGEIMSLS  143
ace2_Pseudopodoces_humili.Block5       KVVCHPTAWDLG.KNDYRIKMCTKVTMDDFLTAHHEMGHIEYDMAYAEQPYLLRSGANEGFHEAVGEIMSLS  143
ace2_Scleropages_formosus.Block5       KVVCHPTAWDMGNREDFRIKMCTKVNMDDFLTVHHEMGHNQYQMAYRNLSYLLRDGANEGFHEAVGEIMSLS  144
```

```
                                          150       160       170       180       190       200       210
conservation
ace_Alligator_mississipp.Block2        VSTPKHLHSINLLP.QVEDNMESDINYLMSIALDKIAFVPFGYLMDQWRWKVFDGRIKEDEYNQQWWNLRLK  215
ace_Alligator_mississipp.Block4        VSTPSHLKKIGLLD.QVVQDTENDINYLLKMALEKIAFLPFGYLIDQWRWNVFSGHTSPNRYNYDWWYLRTK  215
ace_Danio_rerio.Block2                 VATPKHLQSIGLLD.KVEDNAESTINFLMSIALDKIAFLPFGYLMDQWRWKVFDGRISSSEYNKEWWNLRMK  215
ace_Danio_rerio.Block4                 VSTPKHLHSIDLLD.QLTDDAESDINYLLKMALEKIAFLPFGYLIDQWRWSVFSGETPPDRYNADWWYLRTK  215
ace_Homo_sapiens.Block2                VSTPKHLHSLNLLS.SEGGSDEHDINFLMKMALDKIAFIPFSYLVDQWRWRVFDGSITKENYNQEWWSLRLK  215
ace_Homo_sapiens.Block4                VSTPEHLHKIGLLD.RVTNDTESDINYLLKMALEKIAFLPFGYLVDQWRWGVFSGRTPPSRYNFDWWYLRTK  215
ace_Lepisosteus_oculatus.Block2        VSTPKHLHSIGLLD.KVENNYDSDINYLMSIALDKIAFLPFGYLMDQWRWKVFDGRISEQEYNKEWWNLRLK  215
ace_Lepisosteus_oculatus.Block4        VSTPKHLKEIGLLK.SFVPDNESDINYLLKMALEKIAFLPFGYLIDQWRWGVFSGRTPPERYNADWWYLRTK  215
ace_Mus_musculus.Block2                VSTPKHLYSLNLLS.TEGSGYEYDINFLMKMALDKIAFIPFSYLIDQWRWRVFDGSITKENYNQEWWSLRLK  215
ace_Mus_musculus.Block4                VSTPAHLHKIGLLD.HVTNDIESDINYLLKMALEKIAFLPFGYLVDQWRWGVFSGRTPPSRYNFDWWYLRTK  215
ace_Pseudopodoces_humili.Block2        VSTPKHLHSINLLD.QVMENEESDINYLMSIALDKIAFLPFGYLMDQWRWKVFDGRIKDDEYNKEWWNLRMK  215
ace_Pseudopodoces_humili.Block4        VSTPSHLKKIGLLS.SAAEDEESNINYLLKMALEKIAFLPFGYLIDQWRWNVFSGHTPPSRYNYDWWYLRTK  215
ace_Scleropages_formosus.Block2        VSTPMHLHSIGLLD.NAESSYESDINYLMSIALDKIAFLPFGYLMDQWRWKVFDGRISEKDYNKEWWNLRLK  215
ace_Scleropages_formosus.Block4        VSTPKHLKKIGLLE.NLVEDHESDLNYLLKMALEKIAFLPFGFLIDQWRWGVFSGKTPPERYNADWWYLRTK  215
ace2_Alligator_mississipp.Block5       AATPEHLKSIGLLEPTFQEDSDTDINFLLKQALTIVGTMPFTYMLEKWRWMVFRGDIPKEEWMKKWWDMKKE  214
ace2_Danio_rerio.Block5                AATPSHLQSLGLLPSDFKQDYETDINFLLKQALTIVGTLPFTYMLEEWRWQVFKAKIPKDEWMQQWWQMKRE  215
ace2_Homo_sapiens.Block5               AATPKHLKSIGLLSPDFQEDNETEINFLLKQALTIVGTLPFTYMLEKWRWMVFKGEIPKDQWMKKWWEMKRE  215
ace2_Lepisosteus_oculatus.Block5       AATPSHLQALGLLQPDFKEDNETNINFLLKQALTIVATLPFTYMLEEWRWQVFQGQIPKNEWMKRWWEMKRD  216
ace2_Mus_musculus.Block5               AATPKHLKSIGLLPSDFQEDSETEINFLLKQALTIVGTLPFTYMLEKWRWMVFRGEIPKEQWMKKWWEMKRE  215
ace2_Pseudopodoces_humili.Block5       AATPQHLKSLGLLEPTFQDDEETEINFLLKQALTIVGTMPFTYMLEKWRWMVFRGEITKQEWTKRWWEMKRD  215
ace2_Scleropages_formosus.Block5       AATPKHLKALGLLPADFKEDNETDINFLLKQALTIVATLPFTYMLEEWRWQVFQGTIPKDEWMQRWWEMKRD  216
```

```
                                          220       230       240       250       260       270       280
conservation
ace_Alligator_mississipp.Block2        YQGLCPPVPRSEADFDPGAKFHIPANVPYVRYFVSFVIQFQFHQALCKAAGHTGPLYKCDIYKSKEAGTLL  286
ace_Alligator_mississipp.Block4        YQGICPPIARNETNFDPGAKYHIPANTPYIRYFVSFVLQFQFHKALCQAANHTGPLHTCDIYQSKEAGTKL  286
ace_Danio_rerio.Block2                 YQGLCPPVPRTEKDFDPGAKFHIPANVPYIRYFVSFVIQFQFHKGLCDAAGHKGPLHNCDIYQSKEAGKLL  286
ace_Danio_rerio.Block4                 YQGICPPTRRTEEHFDAGGKYHIPGNTPYIRYFVSFILQFQFHEKLCKEAGHTGPLHKCDIYKSREAGAVL  286
ace_Homo_sapiens.Block2                YQGLCPPVPRTQGDFDPGAKFHIPSSVPYIRYFVSFIIQFQFHEALCQAAGHTGPLHKCDIYQSKEAGQRL  286
ace_Homo_sapiens.Block4                YQGICPPVTRNETHFDAGAKFHVPNVTPYIRYFVSFVLQFQFHEALCKEAGYEGPLHQCDIYRSTKAGAKL  286
ace_Lepisosteus_oculatus.Block2        YQGLCPPVPRSEEDFDPGAKFHIPANVPYVRYFVSFVIQFQFHEKLCKAAKHTGPLHMCDIYNSREAGKLL  286
ace_Lepisosteus_oculatus.Block4        YQGICPPIERSEEHFDAGAKFHIPGNTPYIRYFVSFILQFQFHEKLCEAAKHVGPLHQCDIYRSKEAGAIL  286
ace_Mus_musculus.Block2                YQGLCPPVPRSQGDFDPGSKFHVPANVPYVRYFVSFIIQFQFHEALCRAAGHTGPLHKCDIYQSKEAGKLL  286
ace_Mus_musculus.Block4                YQGICPPVARNETHFDAGAKFHIPNVTPYIRYFVSFVLQFQFHQALCKEAGHQGPLHQCDIYQSAQAGAKL  286
ace_Pseudopodoces_humili.Block2        YQGLCPPALRSEDDFDPGAKFHIPANVPYIRYFVSFVIQFQFHQALCDAAGHKGPLHTCDIYQSQEAGKIL  286
ace_Pseudopodoces_humili.Block4        YQGICAPVPRNESNFDPGAKYHIPGNTPYIRYFVSFILQFQFHKALCKAANHNGSLHTCDIYRSKEAGAKL  286
ace_Scleropages_formosus.Block2        YQGLCPPVARSEEDFDPGSKFHIPANVPYVRYFVSFVIQFQFHQALCDAAGHVGPLHTCDIYQSKAAGKLL  286
ace_Scleropages_formosus.Block4        YQGICPPTKRTEEHFDAAAKYHIPGNTPYIRYFVSFILQFQFHERLCQAANHTGPLHQCDIFQSTEAGKIL  286
ace2_Alligator_mississipp.Block5       IVGVVEPVPHDETYCDPAALFHVANDYSFIRYYTRTIYQFQFQEALCKAAKHTGPLYKCDITNSTAAGDKL  285
ace2_Danio_rerio.Block5                LVGVAEAVPRDETYCDPPALFHVSGDYSFIRYFTRTIYQFQFQEALCKAAGHTGPLYKCDITNSTKAGDKL  286
ace2_Homo_sapiens.Block5               IVGVVEPVPHDETYCDPASLFHVSNDYSFIRYYTRTLYQFQFQEALCQAAKHEGPLHKCDISNSTEAGQKL  286
ace2_Lepisosteus_oculatus.Block5       LIGVVEPVPRDESYCDPPALFHVSNDYSFIRYFTRTIYQFQFQKALCDAKGHTGPLYKCDITNSTEAGAKL  287
ace2_Mus_musculus.Block5               IVGVVEPLPHDETYCDPASLFHVSNDYSFIRYYTRTIYQFQFQEALCQAAKYNGSLHKCDISNSTEAGQKL  286
ace2_Pseudopodoces_humili.Block5       IVGVVEPVPHDETYCDPATLFHVANDYSFIRYYTRTIYQFQFQEALCRAANHIGPLHKCDITNSTAAGQKL  286
ace2_Scleropages_formosus.Block5       MVGVVEPLPRDETYCDPAALYHVSNDYSFIRYFTRTIYQFQFQASLCEVAGHTGPLFKCDITNSTAAGNKL  287
```
