# Supplementary figures and images for: A Genomic Survey of Angiotensin-Converting Enzymes Provides Novel Insights into Their Molecular Evolution in Vertebrates
Source: Molecules. 2018 Nov 9;23(11):2923. doi: 10.3390/molecules23112923 (PMC6278350; doi:10.3390/molecules23112923)

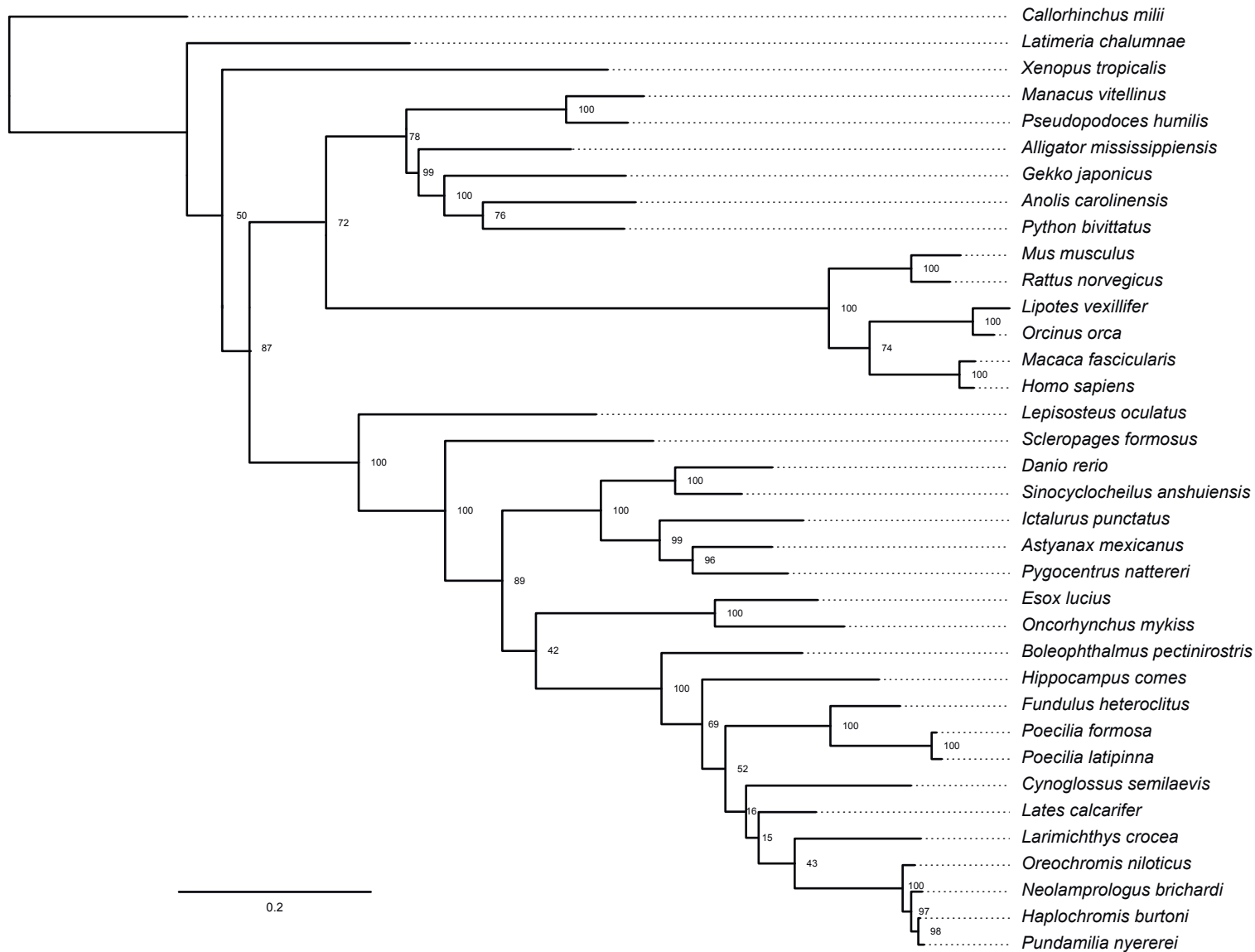

Supplement: Supplementary file 1 [file molecules-23-02923-s001.zip › Figure S4.pdf]

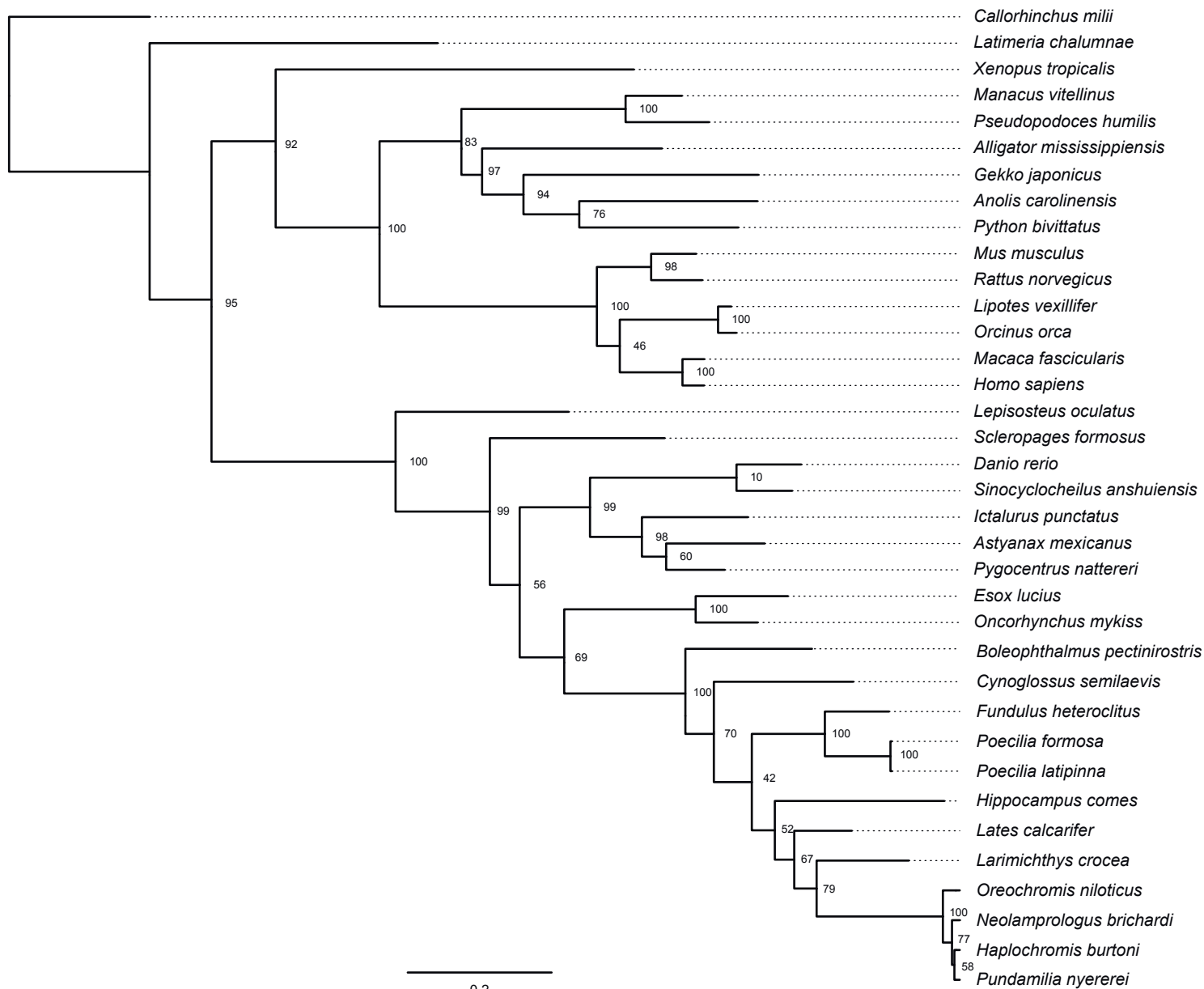

Supplement: Supplementary file 1 [file molecules-23-02923-s001.zip › Figure S5.pdf]

(a) Block 1 compares Block 3

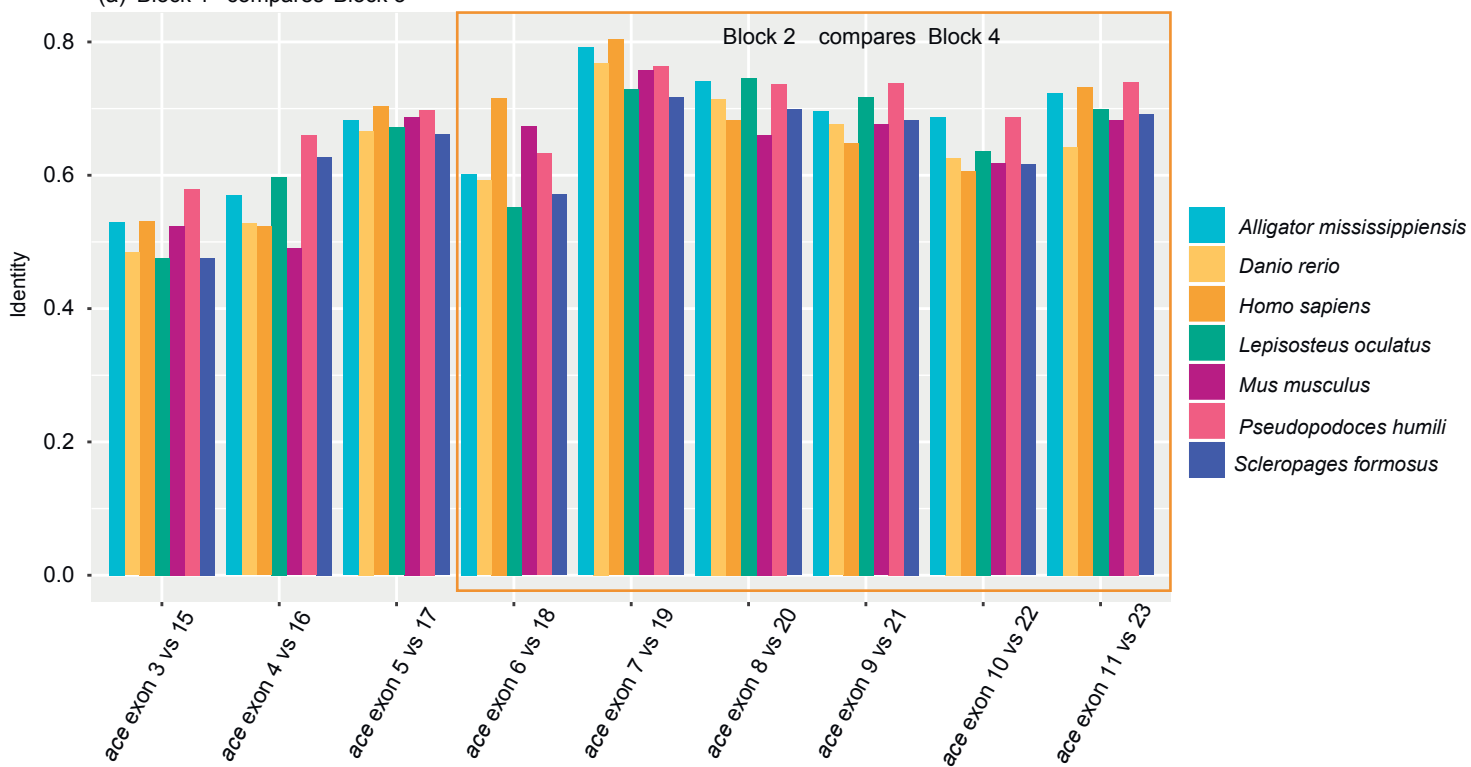

(b) Block 2 compares Block 5

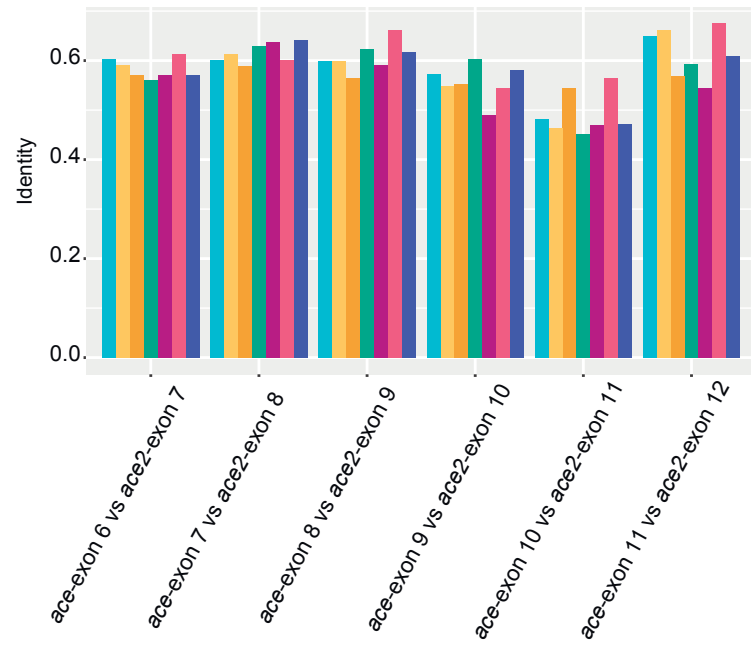

(b) Block 4 compares Block 5

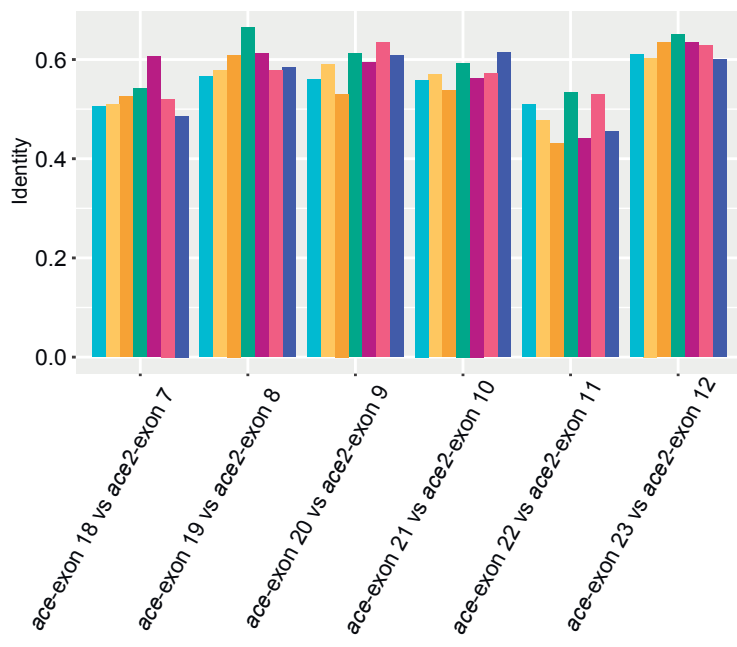

Supplement: Supplementary file 1 [file molecules-23-02923-s001.zip › Figure S7.pdf]
